# Gene modification by fast‐track recombineering for cellular localization and isolation of components of plant protein complexes

**DOI:** 10.1111/tpj.14450

**Published:** 2019-07-26

**Authors:** Zhoubo Hu, Ajit Ghosh, Sara C. Stolze, Mihály Horváth, Bing Bai, Sabine Schaefer, Simone Zündorf, Shanda Liu, Anne Harzen, Mohsen Hajheidari, Tomasz J. Sarnowski, Hirofumi Nakagami, Zsuzsa Koncz, Csaba Koncz

**Affiliations:** ^1^ Max‐Planck Institute for Plant Breeding Research Carl‐von‐Linné‐Weg 10 D‐50829 Cologne Germany; ^2^ Department of Biochemistry and Molecular Biology Shahjalal University of Science and Technology Sylhet 3114, Bangladesh; ^3^ Botanical Institute Cologne Biocenter, Cluster of Excellence on Plant Sciences, University of Cologne D‐50674 Cologne Germany; ^4^ Institute of Biochemistry and Biophysics Polish Academy of Sciences Pawińskiego 5A 02‐106 Warsaw Poland; ^5^ Institute of Plant Biology Biological Research Center of Hungarian Academy of Sciences Temesvári krt. 62 H‐6726 Szeged Hungary

**Keywords:** recombineering, site‐directed gene modification, fluorescent reporters, affinity purification, TFIIH protein kinases, DNA replication‐dependent HISTONE H3.1, technical advance

## Abstract

To accelerate the isolation of plant protein complexes and study cellular localization and interaction of their components, an improved recombineering protocol is described for simple and fast site‐directed modification of plant genes in bacterial artificial chromosomes (BACs). Coding sequences of fluorescent and affinity tags were inserted into genes and transferred together with flanking genomic sequences of desired size by recombination into *Agrobacterium* plant transformation vectors using three steps of *E. coli* transformation with PCR‐amplified DNA fragments. Application of fast‐track recombineering is illustrated by the simultaneous labelling of CYCLIN‐DEPENDENT KINASE D (CDKD) and CYCLIN H (CYCH) subunits of kinase module of TFIIH general transcription factor and the CDKD‐activating CDKF;1 kinase with green fluorescent protein (GFP) and mCherry (green and red fluorescent protein) tags, and a PIPL (His_18_‐StrepII‐HA) epitope. Functionality of modified *CDKF;1* gene constructs is verified by complementation of corresponding T‐DNA insertion mutation. Interaction of CYCH with all three known CDKD homologues is confirmed by their co‐localization and co‐immunoprecipitation. Affinity purification and mass spectrometry analyses of CDKD;2, CYCH, and DNA‐replication‐coupled HISTONE H3.1 validate their association with conserved TFIIH subunits and components of CHROMATIN ASSEMBLY FACTOR 1, respectively. The results document that simple modification of plant gene products with suitable tags by fast‐track recombineering is well suited to promote a wide range of protein interaction and proteomics studies.

## Significance Statement

A PCR‐based BAC‐recombineering protocol accelerating site‐specific modification of plant genes with suitable tags is described and exploited for purification, cellular localization, co‐immunoprecipitation, and mass spectrometry analysis of CDKD;2 and CYCLIN H associated components of TFIIH general transcription factor.

## Introduction

The term recombineering refers to cloning technologies that employ phage‐encoded recombination enzymes, such as Exo, Beta, and Gam of lambda phage Red system, to achieve *in vivo* site‐specific integration of foreign DNA sequences into genes carried by bacterial chromosomes or plasmids (Thomason *et al*., 2014). The λRed system mediates recombination between 50 nucleotide arms of a PCR‐amplified DNA fragment and corresponding homologous sequences flanking the target site, which may be represented by a single nucleotide, a codon triplet, or a longer DNA sequence for generating point mutations, codon exchanges, deletions, and in‐frame insertions of suitable tags, respectively. First, a positive‐negative selectable marker cassette is inserted into the target gene, and then this cassette is replaced by a desired tag, or with a DNA fragment carrying a nucleotide exchange or deletion. The phage genes coding for recombination enzymes are either harboured by a plasmid or stably integrated into the chromosome of bacterial host for recombineering. In the λRed *E. coli* host SW102 (Warming *et al*., 2005), the *exo*,* beta*, and *gam* genes of a defective prophage are expressed by the *pL* promoter, which is induced by temporal inactivation of thermosensitive cI857^ts^ repressor. One of the most popular positive−negative selectable markers is the galactokinase (*galK*) gene, the integration of which into the target site is selected for on minimal medium in a *galK* ^*−*^ host. Subsequently, the exchange of *galK* marker with desired sequences is achieved by counter‐selection on deoxygalactose‐containing medium.

Bacterial artificial chromosome (BAC) clones generated during the genome‐sequencing projects carry 100 kb or larger genomic DNA segments with multiple genes and can be readily recombined into chromosomes of transformed mammalian embryonic stem cells. The modification of mammalian genes by BACs recombineering became a routine high‐throughput approach for the generation of knockout and knock‐in lines, especially in transgenic mice (Sharan *et al*., 2009; Ciotta *et al*., 2011; Narayanan and Chen, 2011). As homologous recombination by BAC DNA transformation did not prove to be feasible in plants, the application of recombineering was coupled to *Agrobacterium*‐mediated gene transfer. This was achieved either by recombineering of plant genes cloned in TACs (transformation competent BAC *Agrobacterium* vectors, Shibata and Liu, 2000; Zhou *et al*., 2011; Alonso and Stepanova, 2015) or by moving the modified plant genes from BACs into *Agrobacterium* binary vectors by gap‐repair recombination (Bitrián *et al*., 2011). Both plant BAC‐recombineering approaches are, however, relatively slow because direct and counter‐selection of the *galK* exchange marker on minimal medium requires weeks, and the first approach is also affected by low frequency of plant transformation with large chromosomal segments of TACs.

To improve the efficacy of plant BAC recombineering, we replaced the *galK* marker with antibiotic resistance genes that are either flanked by cleavage sites of the I‐*Sce*I homing endonuclease and therefore excisable, or linked to an arabinose‐inducible *ccdB* gyrase‐inhibitor killer gene as counterselectable marker. Inhibition of gyrase by ccdB results in the accumulation of DNA double‐stranded breaks, causing ultimate cell death. To avoid unnecessary cloning steps, the target plant genes modified by recombineering were moved from the BACs into PCR‐amplified binary vectors by recombination, and then transferred by *Agrobacterium* into transgenic plants. The improved recombineering tools were used for exploring *in vivo* interactions of Arabidopsis CDKD (CYCLIN‐DEPENDENT KINASE D) homologues of human CDK7 with the CDKD‐activating kinase CDKF;1, CYCLIN H (CYCH) and core components of the RNA polymerase II (RNAPII) general transcription factor TFIIH. The kinase module (TFIIK) of human TFIIH is composed of CDK7, CYCH and MAT1 (Menage a trois 1) assembly factor subunits. TFIIK plays a key role in the activation of cell cycle kinases and, when bound to TFIIH it phosphorylates serine 5 residues of heptapeptide repeats of RNAPII C‐terminal domain promoting transcription initiation. Furthermore, TFIIK is targeted to DNA damage sites by TFIIH and modulates both transcription‐coupled and general genome repair (Fisher, 2012, 2019). Whereas the Arabidopsis CDK7 homologues CDKD;1, CDKD;2 and CDKD;3 are activated by CDKF;1‐mediated T‐loop phosphorylation and phosphorylate serine 5 residues of RNAPII CTD *in vitro* (Hajheidari *et al*., 2012, 2013), their interactions with CYCH and CDKF;1 are not confirmed *in vivo*. Compared with yeast and animal MAT1 homologues, which mediate interaction of TFIIK with the XPB and XPD helicase subunits of TFIIH, the N‐terminal RING domain is missing in the putative Arabidopsis MAT1 (At4 g30820) TFIIK subunit (Umeda *et al*., 2005). As TFIIH was not yet purified in association with TFIIK from plants, it is unknown whether Arabidopsis TFIIK‐TFIIH carries conserved homologues of all TFIIH subunits that were recently characterized by structural studies of human TFIIH (Greber *et al*., 2019). Therefore, we isolated TFIIH complexes from Arabidopsis using the CDKD;2 and CYCH TFIIK subunits modified by recombineering and analyzed their subunit composition by LC‐MS/MS mass spectrometry. In these experiments, we used as nuclear control the DNA replication‐dependent HISTONE H3.1 protein, which is located in silent regions of the genome and incorporated into chromatin during heterochromatin replication (Jacob *et al*., 2014; Otero *et al*., 2016). CDKD and CYCH subunits of the TFIIH kinase module were labelled by recombineering with a PIPL (His_18_‐StrepII‐HA) epitope, and GFP and mCherry (green and red fluorescent protein) tags. The CDKD‐activating kinase CDKF;1 was similarly labelled with GFP and PIPL tags and expressed in the *cdkf;1* T‐DNA insertion mutant to validate functionality of modified gene constructs by genetic complementation. Co‐immunoprecipitation and cellular co‐localization data indicated that CYCLIN H (CYCH) is associated with the Arabidopsis CDKD;1, CDKD;2 and CDKD;3 kinases, but not with their upstream activating kinase CDKF;1. In contrast, co‐immunoprecipitation data confirmed interaction of CDKF;1 with CDKD kinases. Affinity purification and mass spectrometry analysis of CYCH:mCherry and CDKD;2:GFP verified their association with Arabidopsis homologues of conserved core subunits of TFIIH except XPB. Histone H3.1–mCherry purified as nuclear control in the same experiments was identified in complex with three subunits of CAF1 (CHROMATIN ASSEMBLY FACTOR 1, Tagami *et al*., 2004; Jiang and Berger, 2017) and ASF1A/B (ANTI‐SILENCING FUNCTION 1, Lario *et al*., 2013). In summary, the results documented that fast‐track recombineering is well suited to assist the isolation and characterization of components of plant protein complexes.

## Results

### Recombineering using *ccdB* gene cassettes

In recombineering experiments, selectable markers are inserted into desired positions of genes and then replaced with DNA fragments that either code for suitable tags or carry nucleotide exchanges or deletions. Linear DNA fragments of selectable markers and tags used for their replacement are PCR amplified with primers that carry 50 nucleotides (nt) flanks of targeted gene positions. These fragments are transformed into *E. coli* hosts and following pulse‐induction of phage‐encoded enzymes are integrated by recombination aided by the 50‐nt flanks into the designated target sites. The time requirement of recombineering experiments, which replace traditional cloning with simple *E. coli* transformation is primarily determined by the selection conditions applied. *E. coli* hosts with heat‐inducible λRed genes need to be cultured at 32°C to avoid constitutive expression of recombination enzymes (Warming *et al*., 2005). Transformation of such strains with BACs carrying the target plant genes is achieved in a day by selecting for the BAC‐encoded antibiotic resistance markers in complete medium. However, subsequent selection for insertion and replacement of popular *galK* marker on minimal medium requires considerably longer time, hence represents a major bottleneck of recombineering experiments. To accelerate the recombineering procedure, we replaced *galK* with new exchange markers by linking chloramphenicol (*CmR*), kanamycin (*KmR*) and spectinomycin (*SpR*) resistance genes to a *ccdB* DNA‐gyrase (gyrA) inhibitor suicide gene, which is transcribed by an arabinose‐inducible pBAD promoter and controlled by an adjacent *araC* repressor gene (Le Roux *et al*., 2007; see Experimental Procedures, Supporting Information Figure S1).

**Figure 1:**
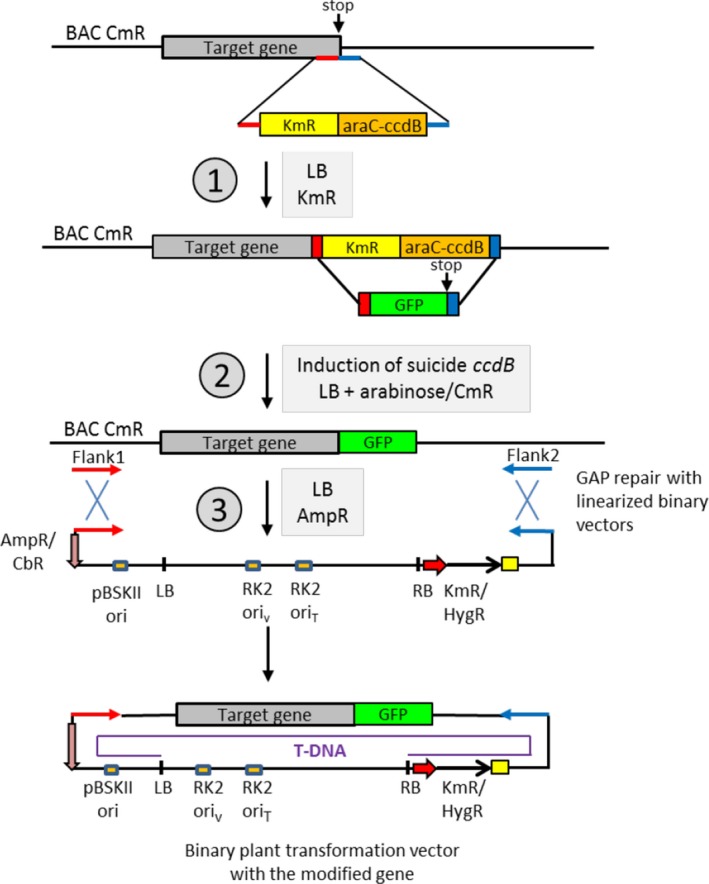
Recombineering with *ccdB* gene cassettes. The work flow of recombineering with the *ccdB* exchange cassettes is illustrated in the example of replacement of the stop codon of *CDKD;3* (At1g18040) gene by the GFP coding sequences (Figure S2e). The *CDKD;3* BAC clone (T10F20) carrying a CmR marker is introduced into the recombineering host *E. coli* SW102 and the presence of the target gene is verified by PCR amplification with gene‐specific primers (green arrows) flanking its stop codon. In the first step of recombineering (1), the SW102 (BAC T10F20) strain is transformed with the DNA fragment of KmR‐araC‐ccdB cassette (2.7 kb), which is PCR amplified with primers carrying 50 nt flanks of the target stop codon (blue and red bars). KmR transformants are selected and regrown in LB‐Km−0.5% glucose medium without selecting for the BAC CmR marker, to enhance the loss of BACs lacking the *ccdB* insertion. Colonies carrying only BACs with the *ccdB* insertion are identified by PCR (2.7 kb + space between the gene‐specific primers). In the second step (2), the obtained SW102 (BAC:*ccdB*) strain is transformed with a DNA fragment of GFP coding region, which is PCR amplified with primers carrying the 50 nt flanks of the stop codon (0.82 kb). Transformants are selected and enriched for the BAC CmR marker in LB medium containing 0.2% arabinose to induce the suicide *ccdB* gene expression. Exchange of the *ccdB* marker with the GFP cassette is monitored by colony PCR (0.72 kb + space between the gene‐specific primers). In the third step (3), the modified plant gene is moved by gap‐repair into an *Agrobacterium* binary vector. When using pGAPKm or pGAPHyg (Bitrián *et al*., 2011; Figure S2a), two BAC segments flanking the modified gene (usually located upstream and downstream of neighbouring genes) are PCR amplified as *Eco*RI‐*Sal*I and *Sal*I‐*Bam*HI fragments and inserted into *Eco*RI−*Bam*HI sites of pGAPs. Subsequently, the vectors are linearized by *Sal*I, phosphatase treated and transformed into SW102 (BAC:GFP). Following selection of AmpR transformants, plasmid DNA is prepared and transformed into *E. coli* DH5α or DH10B. The presence of modified plant gene is verified by restriction enzyme fingerprinting and sequencing with the gene‐specific primers. The verified clone is transformed to the *E. coli* donor stain MFDpir ΔTIV lacIq and the conjugated into *Agrobacterium* GV3101 (pMP90RK) for plant transformation. To save time, the gap‐repair step (3) is performed with PCR‐amplifiable pGAPBRKm and pGAPBRHyg vectors as shown in Figure 2b. BACs carrying a KmR marker are similarly modified using either the SpR‐ccdB or CmR‐ccdB cassette. The latter was used for modification of *CDKF;1*,* CDKD;1* and *CDKD;2* genes (Figure S2b–d). The *ccdB* exchange cassettes can be similarly inserted into any position of a target gene and replaced with DNA fragments carrying point mutations, codon exchanges or deletions.

For recombineering, BAC DNAs carrying the genes of TFIIH‐associated kinases CDKD;1 (At1g73690 BAC F25P22 KmR), CDKD;2 (At1g66750 BACF4N21 KmR) and CDKD;3 (At1g18040 BAC T10F20, CmR) and their upstream activating kinase CDKF;1 (At4g28980, BAC F25O24 KmR) were verified for the presence of target genes by PCR using primers flanking their stop codons (Figure S2 and Table S1). The BACs were transformed into *E. coli* SW102 by selecting for their KmR or CmR markers. In the first step of recombineering (Figure 1), the stop codons of *CDKF;1*,* CDKD;1* and *CDKD;2* genes were replaced by the *CmR‐ccdB* marker, and that of the *CDKD;3* gene with the *KmR‐ccdB* cassette. DNA fragments of *ccdB* cassettes were PCR amplified with primers including 50nt flanks of target gene stop codons (Figure S2 and Table S1) and transformed into the BAC‐containing host strains. Transformants selected for the CmR or KmR markers of *ccdB* cassettes were subsequently grown without selecting for the BAC‐encoded resistance markers to enhance the loss of empty BAC copies lacking *ccdB* insertions, which was monitored by colony PCR with flanking gene‐specific primers.

In the second step, the *ccdB* cassettes were replaced with coding sequences of PIPL tag, which is composed of 18 His residues from the Co^2+^/Ni^2+^‐binding domain of Arabidopsis CobW‐like protein (At1g15730) linked to StrepII and HA (hemagglutinin) epitopes (Figure S3), or with those of the GFP, or a combination of both. These tags were also adapted to generation of N‐terminal fusions for replacing translational start codons (Figure S3), and designed to assist purification of modified plant proteins on Ni^2+^‐agarose, Strep‐Tactin, anti‐HA affinity, and GFP‐Trap resins. Cells carrying only BACs with *ccdB* cassette insertions were transformed with PCR‐amplified fragments of the tags, and then transformants were selected for the BAC‐encoded resistance marker in the presence of 0.2% arabinose to induce the suicide *ccdB* gene. The exchange events were confirmed by screening for the loss of CmR or KmR markers of *ccdB* cassettes and colony PCR with primers flanking the target sites.

In the third step, the tagged genes and neighbouring genomic sequences securing their native transcriptional regulation (i.e. including usually two flanking genes) were transferred by gap‐repair into pGAP binary vectors (Bitrián *et al*., 2011; Table S1, Figures 1 and S2). Two flanks, defining the boundaries of modified genes were cloned into pGAP vectors, which were then linearized between the flanks, dephosphorylated and transformed into SW102 cells carrying the BACs. Gap‐repair recombination between homologous sequences of flanks of linear vectors and BACs resulted in the integration of modified genes into the binary vectors. Following selection for ampicillin resistant (AmpR) transformants, the resulting pGAP clones were fingerprinted by restriction enzyme digestions, and the junctions of inserted tags were confirmed by sequencing using the flanking gene‐specific primers.

Whereas the *ccdB* cassettes could be similarly inserted into any position of a target gene and replaced also by DNA fragments carrying codon exchanges or deletions, the need for finding transformants with homogeneous BAC populations carrying only the *ccdB* insertions delimited the speed of the first step in the procedure. The requirement for cloning of homology arms into the binary vectors for gap‐repair recombination in the third step represented another bottleneck.

### Recombineering with I‐*Sce*I insertion cassettes

**Figure 2:**
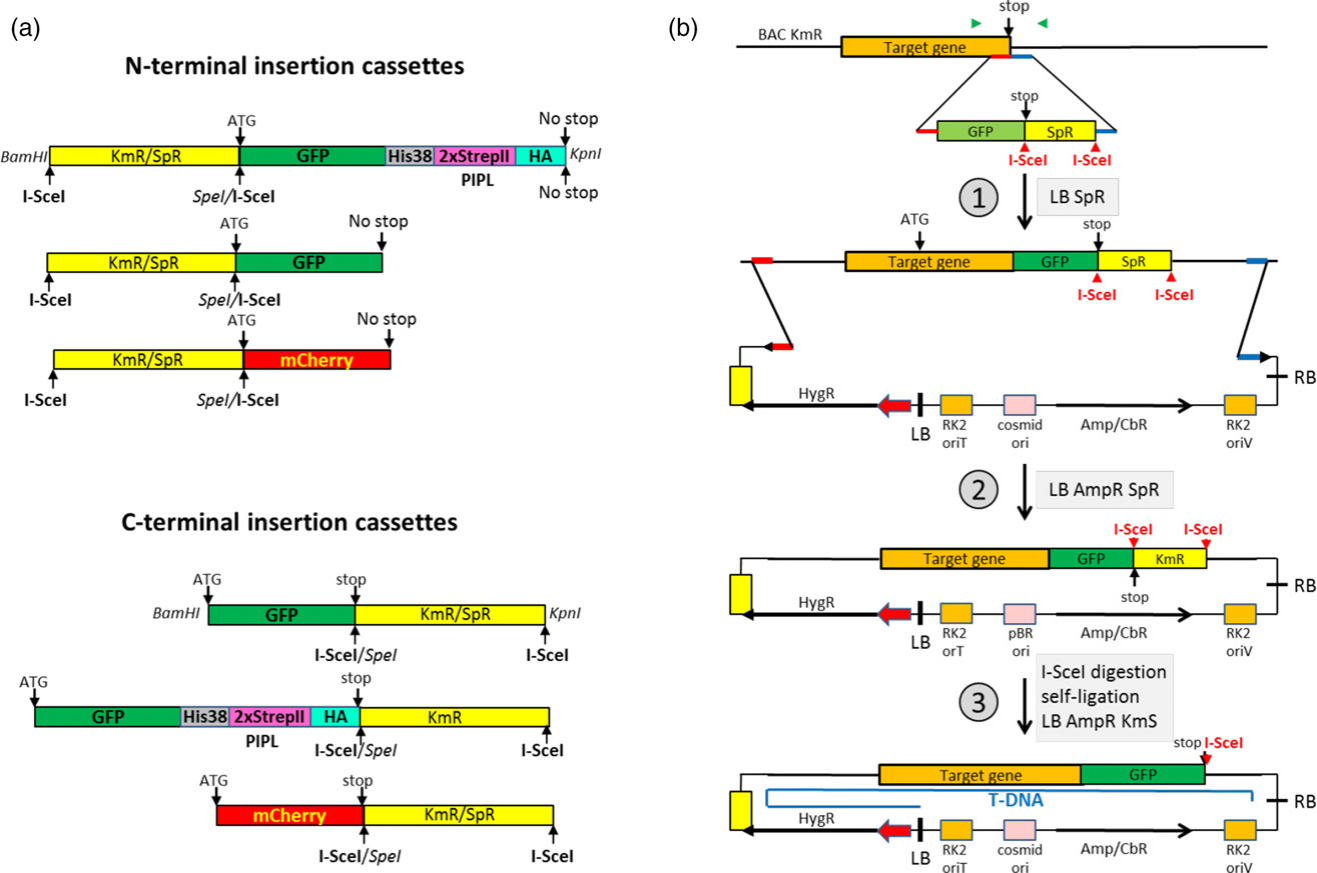
Fast‐track recombineering using I‐*Sce*I insertion cassettes. (a) Schematic presentation of N‐ and C‐terminal KmR and SpR gene‐linked I‐*Sce*I cassettes (Figure S4) designed for replacement of start and stop codons of target genes with coding regions of GFP, mCherry and PIPL (His_18_StrepII‐HA) epitope. (b) The work flow of fast‐track recombineering is illustrated schematically by the replacement of stop codons of *CYCH* and *H3.1* genes (Figure S2f,g), which are carried by BACs with KmR markers. The BAC harbouring the target gene is transformed into the recombineering host SW102 and verified by PCR amplification of a segment of target gene with primers flanking its stop codon (green arrowheads). In the first step of recombineering (1), the C–GFPstop‐SpR I‐*Sce*I cassette (Figure S4) is PCR amplified with primers carrying 50‐nt flanks of the stop codon (red and blue bars) and the cassette DNA fragment (2.07 kb) is transformed into SW102 harbouring the target BAC. Transformants are selected for the SpR marker of the I‐*Sce*I cassette and verified by colony PCR with the gene‐specific primers. The PCR will detect BACs both with and without cassette insertions (2.07 kb + space between the primers versus distance between the gene‐specific primers). In the second step (2), the target gene carrying the I‐*Sce*I cassette insertion replacing its stop codon is moved by gap‐repair into the pGAPBRHyg (or pGAPBRKm, Figure S5) binary vector. pGAPBRHyg is linearized with *Bam*HI, phosphatase treated (see Experimental Procedures for necessary control step), and PCR amplified with primers that carry 50 nt flanks of BAC sequences designed for transfer into plants linked to the modified target gene (Figure S2f,g). The purified linear pGAPBRHyg is transformed into SW102 (BAC:GFPstop‐SpR). Following selection of AmpR transformants, plasmid DNA is prepared and transformed into *E. coli* DH10B to purify the pGAPBRHyg clones from the resident BACs. In the third step (3), the pGAPBRHyg clone is fingerprinted with restriction enzymes, cleaved by I‐*Sce*I, self‐ligated and transformed into *E. coli* DH10B. AmpR transformants are screened for the loss of SpR marker and subjected to verification by sequencing the junction of modified plant gene in pGAPBRHyg using the gene‐specific primers. Finally, the construct is transferred by conjugation from *E. coli* into *Agrobacterium* for plant transformation as described in Figure 1.

When designing a fast‐track version of recombineering, it was considered that the majority of plant gene modifications aims at labelling the gene products with N‐ and C‐terminal fusions to fluorescent or affinity tags. Therefore, we constructed a set of N‐ and C‐terminal insertion cassettes, in which the KmR and SpR genes flanked by recognition sites of the homing endonuclease I‐*Sce*I were fused to coding sequences of GFP, mCherry and GFP–PIPL tags (Figures 2a and S4). To avoid unnecessary cloning steps, PCR‐amplifiable binary vectors (6.5–6.7 kb, Figure S5) with a cosmid replicon, bacterial ampicillin (AmpR)/carbenicillin (CbR) resistance marker, and conditional RK2 conjugational transfer and replication origins (ori_T_ and ori_V_) were constructed (pGAPBRKm and pGAPBRHyg; Experimental Procedures). Studies of the T‐DNA integration mechanism and sequencing plant DNA junctions of T‐DNA insertions indicated that, compared with the left T‐DNA border, the right border is less prone to deletions as it is protected by a covalently linked VirD2 protein during T‐DNA transfer from *Agrobacterium* into plants (Gelvin, 2017). To select for the integration of full‐length T‐DNA inserts into plant chromosomes, therefore, the plant selectable markers were placed into the vicinity of T‐DNA left border, whereas the site used for linearization and PCR amplification of vectors, and integration of tagged plant genes by homologous recombination was positioned close to the right T‐DNA border.

We used the I‐*Sce*I insertion cassettes for in‐frame replacement of stop codons of *CYCLIN H* (*CYCH* At5g27620; BACF15A18 KmR) and *HISTONE H3;1* (AT5G65360; BAC MNA5 KmR) genes with coding sequences of GFP and mCherry (Figure S2). In the first step (Figure 2b), PCR‐amplified C–mCherrystop‐SpR and C‐GPFstop‐SpR cassette fragments (Figure S4 and Table S1) with corresponding flanks were transformed into *E. coli* SW102 carrying the verified BACs, and then transformants were selected for the SpR marker of I‐*Sce*I cassette insertions.

To move the insertion cassette‐containing genes into *Agrobacterium* vectors in the second step, the pGAPBR vectors were linearized by *Bam*HI digestion and PCR amplified with primers that carried 50 nt flanks marking the boundaries of plant genomic regions of BACs destined for transfer into plants (Figures S2 and S5). From AmpR SpR colonies obtained by transformation, plasmid DNA was isolated and transformed into a regular *E. coli* host, such as DH5α or DH10B. The selection for gap‐repair of target plant genes that carried the insertion cassettes with the SpR marker made it unnecessary to remove the untagged BACs before performing the gap‐repair.

Finally, the SpR selectable marker was removed in the third step from the modified plant genes by I‐*Sce*I digestion and transformation of self‐ligated recombinant binary vectors into *E. coli* followed by screening for AmpR and Sp‐sensitive colonies. Analogously to the Gateway and Cre/*Lox* site‐specific recombination techniques, the digestion left an I‐*Sce*I footprint of 27 bp after the integrated GFP and mCherry tags in the modified plant genes. The resulting recombinant vectors were verified by endonuclease fingerprinting and sequencing. The RK2 ori_T_ function aided easy conjugation of binary vectors from *E. coli* into *Agrobacterium* GV3101 (pMP90RK, Koncz and Schell, 1986), whereas the RK2 ori_V_ replication origin secured their maintenance in the latter host, which provided the *trans*‐acting *trfA* replication helper function on the disarmed Ti‐plasmid pMP90RK. RK2 conjugation helper functions of pMP90RK also assisted back‐conjugation of the vectors from *Agrobacterium* to *E. coli*, in order to test their integrity before plant transformation. Detailed protocols of recombineering with the *ccdB* exchange marker and I‐*Sce*I insertion cassettes are provided in the Experimental Procedures.

### Expression and cellular localization of proteins labelled by recombineering in Arabidopsis

**Figure 3:**
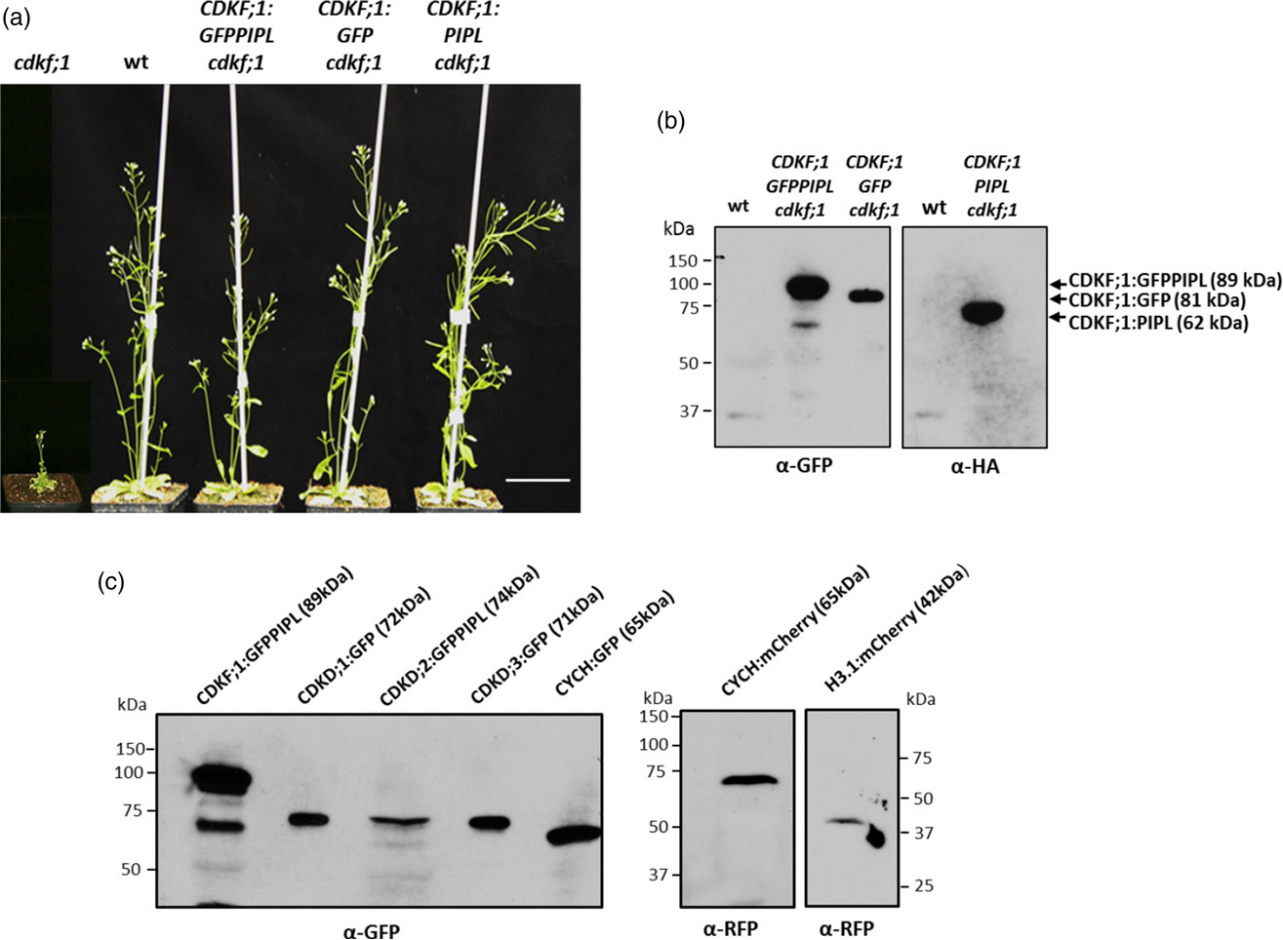
Genetic complementation of *cdkf;1* mutation with modified native *CDKF;1* gene constructs and confirmation of expression of CDKF;1, CDKD, CYCH and HISTONE H3.1 proteins labelled by recombineering in transgenic plants. (a) Comparison of phenotypes of *cdkf;1* mutant and genetically complemented mutant lines carrying the *CDKF;1:GFP–PIPL, CDKF;1–GFP* and *CDKF;1:PIPL* constructs. Bar, 7 cm. (b) Comparison of expression levels of CDKF;1:GFP–PIPL, CDKF;1:GFP and CDKF;1:PIPL proteins in the genetically complemented *cdkf;1* mutant by western blotting with anti‐GFP and anti‐HA (PIPL cross‐reacting) antibodies. (c) Confirmation of expression of CDKF;1, CDKD, CYCH and H3.1 proteins labelled by GFP/PIPL and mCherry tags using recombineering in wild type transgenic plants by western blotting with anti‐GFP and anti‐RFP antibodies. Except for CDKD;1:GFP and CDKD3:GFP, equal aliquots (25 μg) of total protein extracts from 15‐day‐old seedlings were used for western blotting. CDKD;1:GFP and CDKD;3:GFP were isolated by affinity purification on GFP‐Trap from 20 mg protein prepared in parallel from seedlings grown under identical conditions.

All genes modified by recombineering were transformed in *Agrobacterium* binary vectors into wild type Arabidopsis plants. Although the insert size in the pGAPBR vectors varied from 4.7 kb (HISTONE H3.1 clones) to 14.2 kb (CDKD;2 constructs; Table S1), the transformation efficiencies were similar to those obtained with the empty pGAP and related pPCV binary vectors (the transformation frequencies ranged between 0.5% and 1.2% of T1 seed obtained by infiltration of inflorescences; Koncz *et al*., 1994; Ríos *et al*., 2002). Transformants showing 3:1 segregation of single T‐DNA insertions were propagated to isolate homozygous T3 lines. *CDKF;1* gene constructs carrying the GFP–PIPL, GFP and PIPL tags were also introduced into the *cdkf;1‐2/+* (GABI_315A10, Hajheidari *et al*., 2012) T‐DNA insertion mutant. T2 lines carrying single T‐DNA inserts of pGAP vectors were screened for homozygous status of sulfadiazine resistance marker of *cdkf;1‐2* mutation, and then at least three independent T3 offspring harbouring the kanamycin or hygromycin resistance markers of complementing *CDKF;1:GFP–PIPL*,* CDKF;1:GFP* and *CDKF;1:PIPL* constructs in homozygous form were identified. Compared with an extreme dwarf phenotype of *cdkf;1‐2* mutant, all selected T3 lines were wild type indicating genetic complementation of the mutation and verifying full functionality of tagged *CDKF;1* gene constructs (Figure 3a). Western blotting of equal aliquots of total protein extracts from randomly chosen T3 lines confirmed comparable expression levels of tagged CDKF;1 kinase proteins in the complemented *cdkf;1‐2* mutant (Figure 3b). Following microscopic inspection of GFP and mCherry expression in roots and hypocotyls of wild type T3 seedlings, the expression of CDKF;1:GFP–PIPL; CDKD;2:GFP–PIPL, CDKD;3:GFP, CYCH:GFP, CYCH:mCherrry and Histone H3;1:mCherry proteins of expected molecular mass was analogously confirmed by western blotting of total protein extracts with anti‐GFP and anti‐RFP antibodies. As expression levels of CDKD;1:GFP and CDKD;3:GFP proteins were low, their detection required previous enrichment by affinity purification on GFP‐Trap resin (Figure 3c).

Examination of expression patterns of these fusion proteins in 15‐day‐old seedlings by confocal microscopy revealed that CDKF;1, CYCH, CDKDs and the control DNA replication‐dependent histone H3.1 showed the highest levels in root tip, division and elongation zones, but lower expression in differentiated cells of lateral roots and hypocotyls. Except for CDKF;1 and CDKD;2, the amounts of other examined TFIIH components and histone H3.1 were particularly low in leaves. Compared with CDKD;2, CDKD;1 and CDKD;3 showed low expression in all organs except primary roots (Figure S6). All examined proteins were also detected in the regions of sperm and vegetative cells in pollen grains, various cell types of pistils, and epidermal cells of immature seeds, in which CDKD;1 levels were the lowest (Figure S7). Compared with CDKF;1 and CYCH, CDKDs and histone H3.1 displayed higher level of nuclear localization in the examined cell types.

**Figure 4:**
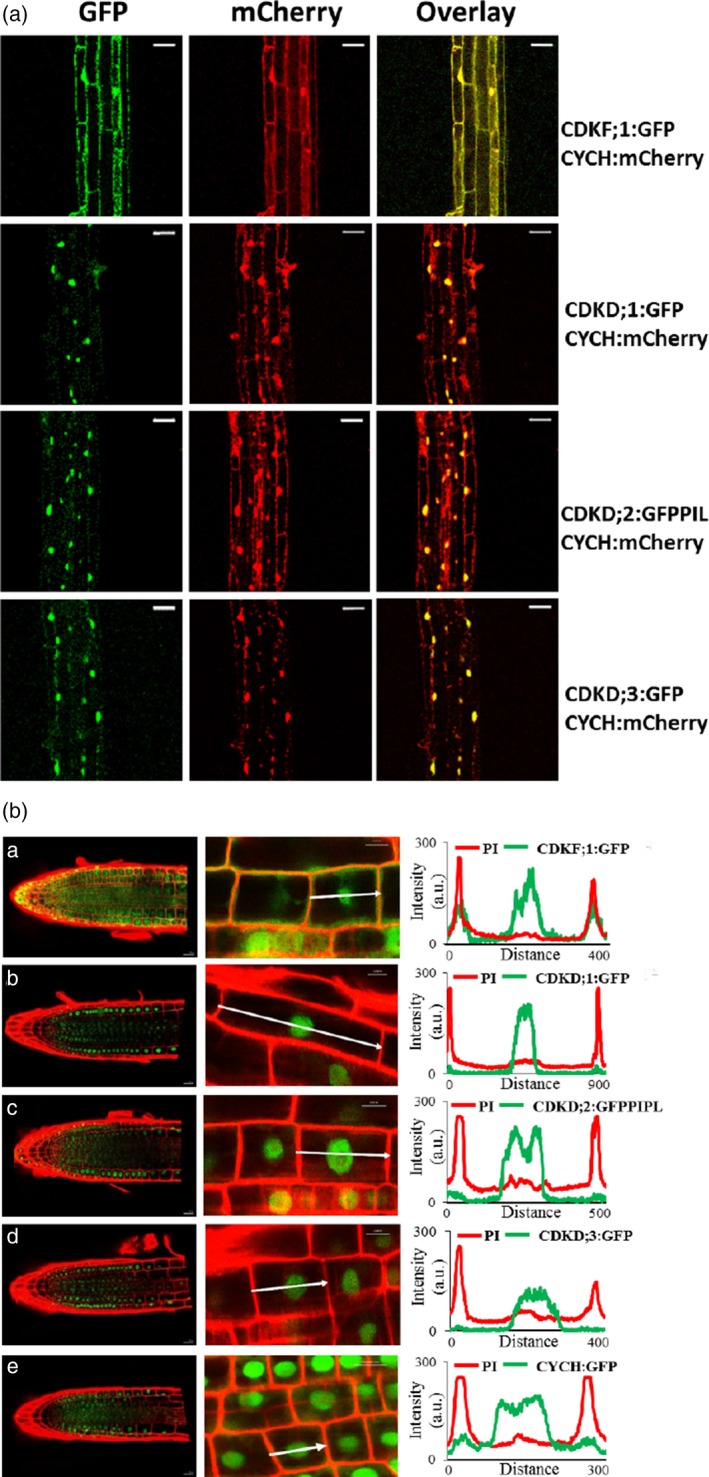
Co‐localization of CYCH:mCherry with CDKF;1:GFP, CDKD;1:GFP, CDKD:2:GFP–PIPL and CDKD;3:GFP in hypocotyl cells and line intensity profile analysis of subcellular distribution GFP/PIPL‐labelled CDKF;1, CDKD and CYCH proteins in propidium iodine‐stained roots cells. (a) Confocal images and overlay of GFP‐labelled CDKF;1 and CDKDs and mCherry‐labelled CYCH in hypocotyl cells indicate overlapping localization of CDKF;1 with CYCH in the cytoplasm and nuclei. In contrast, CDKDs show nuclear co‐localization with CYCH. Bars: 20 μm. (b) Scanning of green GFP and PI‐stained red cell wall fluorescence through individual root cells detects CDKF;1:GFP in both cytoplasm and nuclei. In comparison, CDKD;2:GFP and CYCH:GFP show lower accumulation in the cytoplasm, whereas the CDKD;1:GFP and CDKD;2:GFP–PIPL signals are confined to nuclei. White arrows: 10 μm.

To compare subcellular co‐localization of CYCH to those of CDKF;1 and CDKDs, the CYCH:mCherry construct linked to a plant hygromycin resistance marker was transformed into lines carrying the *CDKF;1:GFP*,* CDKD;1:GFP* and *CDKD;3:GFP* genes linked to KmR gene, and crossed with the CDKD;2–GFP–PIPL line, which harboured a HygR marker. In hypocotyl cells of subsequently isolated double homozygous seedlings, subcellular localization of CDKF;1:GFP and CYCH:mCherry overlapped yielding orange fluorescence in the cytoplasm around the plasma membrane and nuclei. In contrast, signals of CDKD:GFP fusion proteins showed an overlap with CYCH:mCherry only in cell nuclei (Figure 4a). These results were corroborated by line intensity profile analysis of fluorescence signals of GFP‐labelled CDKF;1, CDKD and CYCH proteins through the medians of propidium iodine (PI, red) counter‐stained single root cells. High cytoplasmic GFP signals were detected close to the peaks of PI‐stained red cell wall positions in CDKF;1:GFP expressing cells, and at lower levels in cells expressing the CYCH:GFP and CDKD;2:GFP–PIPL proteins, respectively. In contrast, the GFP signals of CDKD;1 and CDKD;3 kinases were confined to the area of root cell nuclei (Figure 4b).

### Differential interaction of CDKD kinases with CYCH and CDKF;1

**Figure 5:**
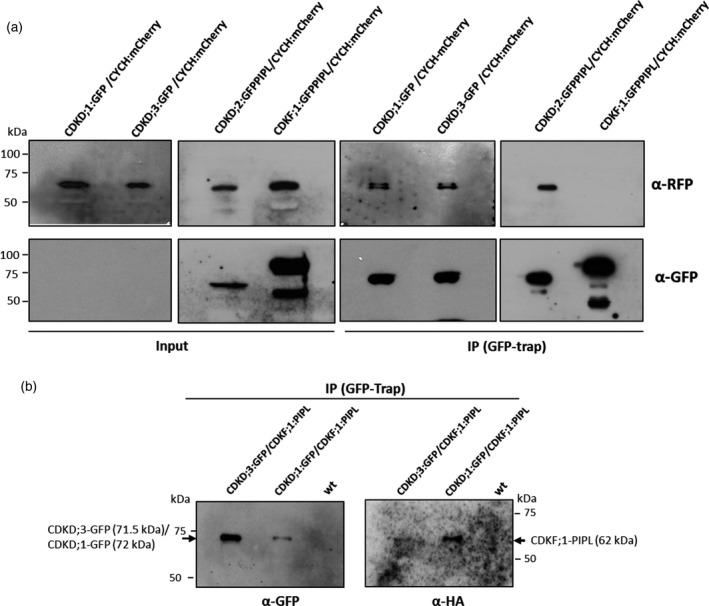
Differential interaction of CDKF;1 with CDKDs and CYCH. (a) CDKD;1:GFP, CDKD;2:GFP–PIPL, and CDKD;3:GFP were affinity purified on GFP‐Trap using 20 mg protein extracts prepared from 15‐day‐old seedlings co‐expressing CYCH:mCherry. Western blotting of proteins eluted from the GFP‐Trap by anti‐RFP and anti‐GFP antibodies indicates that CYCH:mCherry is immunoprecipitated by all three CDKDs, but not by CDKF;1. Compared with CDKD;2:GFP–PIPL, CDKD;1:GFP and CDKD;3:GFP are expressed at lower levels and therefore were not detected in the input protein (50 μg) fractions. (b) CDKD;1:GFP and CDKD;3:GFP were affinity purified on GFP‐Trap as in (a) from 15‐day‐old seedlings co‐expressing CDKF;1–PIPL. Western blotting of proteins eluted from the GFP‐Trap with anti‐HA antibody detecting the HA epitope of PIPL tag indicates association of both CDKD;1:GFP and CDKD:3:GFP with CDKF;1–PIPL. Co‐immunoprecipitation of CDKF;1 with CDKD;2 was previously shown by Shimotohno *et al*. (2004), and is confirmed by identification of CDKF;1 in complex with GFP‐Trap purified CDKD;2–GFP–PIPL by LC‐MS/MS mass spectrometry (Table S2).

It is still an open question whether all three Arabidopsis CDKD kinases are found in similar complexes with CYCH and function as TFIIH‐associated kinases phosphorylating the RNAPII CTD. According to Shimotohno *et al*. (2006), CDKD;1 cannot phosphorylate the RNAP II CTD, and CDKD;3 shows only very weak interaction with CYCH, suggesting that the main CYCH‐associated TFIIH kinase is CDKD;2. In contrast, Hajheidari *et al*. (2012) showed that all three Arabidopsis CDKD kinases were active, phosphorylate serine 5 residues of RNAP II CTD and their activities were increased by CYCH binding and CDKF;1‐mediated phosphorylation of their conserved T‐loop threonine residues. To examine *in vivo* interaction of CYCH with CDKDs and CDKF;1 in Arabidopsis, protein extracts from seedlings co‐expressing CYCH:mCherry with CDKF;1:GFP–PIPL, CDKD;1:GFP, CDKD;2:GFP–PIPL and CDKD;3:GFP were affinity purified on GFP‐Trap and subjected to western blotting with anti‐RFP and anti‐GFP antibodies. Although CDKD;1:GFP and CDKD;3:GFP were not detectable in the input fractions because their levels were much lower in seedlings compared with CDKD;2 and CDKF;1, the association of CYCH:mCherry with all three CDKD kinases was clearly demonstrated after GFP‐Trap purification by western blotting with anti‐RFP antibody (Figure 5a). In contrast, CDKF;1: GFP–PIPL failed to pull‐down CYCH:mCherry. To examine the interaction of CDKF;1 with CDKDs, the CDKF;1:PIPL construct was introduced into CDKD;1:GFP and CDKD;3:GFP expressing plants to isolate subsequently double homozygous lines. Protein extracts from these lines were purified by GFP‐Trap and subjected to western blotting with anti‐HA antibody detecting the HA epitope of CDKF;1‐fused PIPL tag, as well as with anti‐GFP antibody to monitor the CDKD baits. The results confirmed that both CDKD;1 and CDKD;3 were associated with their activating CDKF;1 kinase (Figure 5b).

### Identification of CDKD;2:GFP and CYCH:mCherry associated proteins and interacting partners of HISTONE H3.1 by mass spectrometry

**Figure 6:**
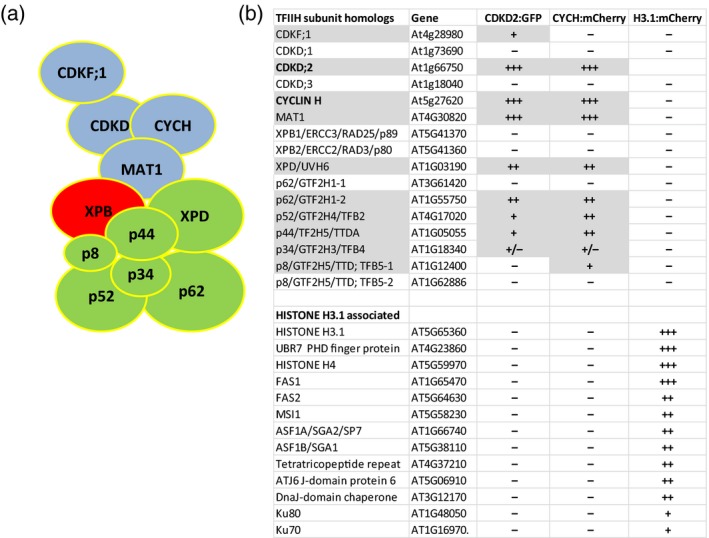
Summary of results of LC‐MS/MS analyses. (a) Schematic presentation of arrangement of TFIIH subunits based on cryo‐electron microscope study of TFIIH structure (Greber *et al*., 2019). Components of the kinase module (blue) interact through MAT1 with the XPB and XPD helicases of human TFIIH core. However, in Arabidopsis, TFIIH subcomplexes purified by CDKD;2 and CYCH the XPB subunit is absent and the representation of p34 is largely reduced. Compared with XPD and p62, all remaining TFIIH subunits show lower abundance in purified CDKD;2 and CYCH complexes. (b) List of proteins identified in the purified CDKD;2:GFP, CYCH:mCherry and HISTONE H3.1:mCherry complexes. Representation of peptide peaks of different subunits measured by LC‐MS/MS (Tables S2 and S3) is indicated schematically (+ signs).

Expression of modified TFIIH components in transgenic wild type (wt) and mutant Arabidopsis lines provided suitable starting materials for affinity purification of corresponding proteins and their associated factors. CDKD;2–GFP–PIPL was purified using standardized amounts of protein extracts from three biological replicates of 4‐week‐old rosette plants in parallel with similar samples from control wild type and YFP−HA (yellow fluorescent protein−hemagglutinin epitope) expressing plants by affinity binding to GFP‐Trap. CYCH–mCherry was analogously isolated on RFP‐Trap using control extracts from wild type and HISTONE H3.1:mCherry expressing plants. Comparison of common components of CDKD;2–GFP–PIPL and CYCH–mCherry complexes identified by mass spectrometry (Tables S2 and S3) indicated that CDKD;2 and CYCH were associated with each other, as well as with Arabidopsis homologues of MAT1 (cyclin‐dependent kinase‐activating kinase assembly factor‐related AT4G30820), XPD (5′ to 3′ helicase XERODERMA PIGMENTOSUM GROUP D, AT1G03190), and p62 (GTF2H1‐2, AT1G55750) TFIIH subunits. In addition, different amounts of p52 (GTF2H4/TFB2, AT4G17020) were pulled down by CDKD;2 and CYCH, which were also associated with comparably lower amounts of p44 (GTF2H2/TF2H5/TTDA, AT1G05055) and p34 (GTF2H3/TFB4, AT1G18340) TFIIH subunits. The smallest TFIIH subunit p8 was detected only in the CYCH:mCherry pull‐down, while corroborating the co‐immunoprecipitation data the CDKD‐activating kinase CDKF;1 was only found in a complex with CDKD;2–GFP. Intriguingly, both CDKD;2 and CYCH complexes lacked however the 3′ to 5′ helicase XPB subunit of TFIIH (Figure 6). These data confirmed the results of previous mass spectrometry studies, which identified CDKD;2 in complex with MAT1, CYCH. XPD, p62 and p52 in Arabidopsis cell suspension (Van Leene *et al*., 2010) and extended them by showing that CYCH can be isolated in association with all known conserved TFIIH subunits except for XPB (Fan and DuPrez, 2015; Rimel and Taatjes, 2018; Greber *et al*., 2019).

Simultaneous identification of unique proteins showing co‐purification only with H3.1:mCherry, used as nuclear control in the analysis of CYCH‐associated factors, confirmed interaction of HISTONE 3.1 with HISTONE 4 (AT5G59970), as well as with conserved FAS1 (FASCIATA 1, AT1G65470), FAS2 (FASCIATA 2, AT5G64630) and MSI1 (MULTICOPY SUPRESSOR OF IRA1, AT5G58230) subunits of CAF‐1 (CHROMATIN ASSEMBLY FACTOR 1; Serra‐Cardona and Zhang, 2018) and their interacting ASF1 partners (ANTI‐ SILENCING FUNCTION 1A, AT1G66740 and 1B, AT5G38110; Lario *et al*., 2013). CAF1 and ASF1 play a pivotal role in the deposition of histone H3/4 core during DNA replication and are involved in the regulation of DNA repair, recombination, endoreduplication, epigenetic imprinting, heterochromatin silencing and cell fate determination (Cheloufi and Hochedlinger, 2017; Jiang and Berger, 2017). Among other candidates of H3.1‐interactors (Figure 6, Table S3), the PHD finger protein (AT4G23860) was identified as a homologue of human histone H3‐binding UBR7 E3 ubiquitin ligase, which mediates K120 ubiquitination of histone H2B and acts as breast cancer tumour suppressor (Kleiner *et al*., 2018; Adhikary *et al*., 2019). The tetratricopeptide repeat protein NASP (AT4G37210) was identified as a member of conserved H3‐binding SHNi‐TRP domain factors that mediate deposition of H3‐variants in yeast and Arabidopsis (Dunleavy *et al*., 2007; Maksimov *et al*., 2016), while the DNA‐J chaperones ATJ6 and AT3G12170 were found to be closely related to the human nuclear H3‐binding factor DNAJC9 (Campos *et al*., 2015; Lambert *et al*., 2015). Finally, Ku70 (AT1G16970) and Ku80 (AT1G48050) in the list of putative H3.1‐interactors represented key factors that bind to DNA ends at double‐stranded breaks and interact with components of the nonhomologous end‐joining (NHEJ) DNA repair pathway, including the human CAF1 complex (Hoek *et al*., 2011). Although these protein interactions remained to be confirmed by subsequent studies, the results of mass spectrometry analyses illustrated that fast‐track recombineering can be applied as a useful tool to assist in the isolation and identification of components of plant protein complexes.

## Discussion

### Unique advantages of recombineering

Compared with Gateway and Gibson assembly of gene constructs, recombineering offers the advantage that it circumvents PCR amplification of target genes and provides a means for their seamless site‐specific modification by homologous recombination in BACs. As it is difficult to predict *a priori* the localization of full‐length functional promoter sequences, larger genomic regions including at least two genes flanking the target are transferred into an *Agrobacterium* binary vector and then to the plant for maintaining native transcriptional regulation of the studied plant gene. It is particularly important when, for example, a bidirectional promoter region controls expression of the target and its upstream neighbouring gene (see for example *miR159a* and *CDKD;1* genes in Figure S2), or when 3′‐UTRs of target and downstream neighbouring gene overlap, suggesting potential generation of natural antisense natsi‐RNAs (as is the case for the *CDKD;3* and AT1G18030 *PP2C* genes in Figure S2). Replacement of the *galK* exchange marker by antibiotic resistance gene‐linked cassettes and the use of PCR‐amplifiable *Agrobacterium* binary vectors for gap‐repair cloning of modified target genes removed two major bottlenecks from the recombineering procedure reducing its total time requirement to 12–14 days, including three *E. coli* transformation steps with PCR‐amplified DNA fragments. From the two protocols described, the application of *ccdB* cassettes (Figures 1 and S1) in combination with PCR‐amplifiable pGAPBR gap‐repair vectors (Figure S5) is necessary when internal nucleotide or codon exchanges are generated in the target gene. The use of I‐*Sce*I insertion cassettes (Figures 2 and S4) simplifies the replacement of translational start or stop codons of target genes with coding sequences of desired tags. N‐terminal and C‐terminal I‐*Sce*I insertion cassettes were constructed such that their GFP/PIPL and mCherry sequences can be replaced with any other desired tag. As the most laborious step in recombineering is the preparation of heat‐induced electrocompetent cells, its application is particularly effective when all genes in a single BAC are simultaneously modified. This approach could facilitate systematic genome‐wide labelling of plant gene products with desired tags. In the examples described above, we illustrated that labelling of interacting proteins with GFP and mCherry fluorescent protein tags provides a simple means for their cellular co‐localization during different developmental stages and in various cell types. The same fluorescent tags are also amenable to support more detailed fluorescence resonance energy transfer (FRET) interaction studies (Albertazzi *et al*., 2009; Liao *et al*., 2019). In addition, the availability of immobilized alpaca and llama nanobodies against GFP and RFP facilitate simple affinity purification and subsequent mass spectrometry analysis of plant gene products labelled by these tags using recombineering. To assist multistep affinity purification of protein complexes, we also created a combined affinity tag, PIPL, which is applicable for pre‐enrichment of tagged proteins e.g., on Ni^2+^‐agarose or on anti‐HA matrix following elution by HA‐peptide (Farrás *et al*., 2001). Fast‐track recombineering could particularly accelerate ‘proteomics walking’ by confirmation of interactions between newly identified components of protein complexes and identification of their novel interacting partners. This application is illustrated in the model study of CDKF;1 interaction with CDKDs and CYCH followed by purification of CDKD;2 and CYCH complexes and identification of their common components.

### Interaction of CDKD TFIIH kinases with CDKF;1 and CYCH

Arabidopsis CDKD;1–3 are closely related homologues of budding yeast Kin28, fission yeast Msc6 and metazoan CDK7 protein kinases, which together with cyclin H and the MAT1 (Menage a trois 1) assembly factor form the trimeric kinase module (TFIIK) of general transcription factor TFIIH (Rimel and Taatjes, 2018; Kolesnikova *et al*., 2019). When unbound of TFIIH, TFIIK plays a pivotal role in cell cycle control through activation of cell cycle kinases by T‐loop phosphorylation. Cell cycle kinase‐activating kinase (CAK) activity of human CDK7 is mediated by autophosphorylation and CYCH binding (Martinez *et al*., 1997; Fisher, 2012, 2019), whereas for binding of Msc2/cyclin H Msc6 requires its T‐loop phosphorylation by the Csk1 CAK‐kinase (CAKAK), which has no CAK activity (Hermand *et al*., 2001; Devos *et al*., 2015). In contrast, budding yeast Kin28 has no CAK activity (Cismowski *et al*., 1995) and its activating T‐loop phosphorylation is mediated by the CDC28 cell cycle kinase‐activating kinase Cak1/Cvi1 (Kaldis *et al*., 1996). Arabidopsis CDKF;1 identified as a suppressor of yeast *cak1* and *csk1* mutations (Umeda *et al*., 1998; Shimotohno *et al*., 2004) phosphorylates the T‐loops of CYCH‐dependent CDKD TFIIH kinases (Hajheidari *et al*., 2012), but does not affect the activities of CDKA;1 and CDKB;1/2 cell cycle kinases (Takatsuka *et al*., 2009). Based on genetic analysis of double and triple mutants, Hajheidari *et al*. (2012) found that the three Arabidopsis CDKD homologues perform overlapping functions. In contrast, Shimotohno *et al*. (2004) reported that CDKD;1 is an inactive enzyme, which is not phosphorylated by CDKF;1, and found that CDKD;3 has higher CDKA;1 phosphorylating activity and lower CYCH‐binding capability compared with CDKD;2 (Shimotohno *et al*., 2003, 2006). In addition, Takatsuka *et al*. (2009) observed that the lack of T‐loop phosphorylation in the *cdkf;1* mutant leads to selective transcriptional downregulation and degradation of CDKD;2 but does not affect CDKD;3 levels (Takatsuka *et al*., 2009). To support the conclusion that CDKDs perform distinct functions, Takatsuka *et al*. (2015) also reported that a *cdkd;1/+cdkd;3/cdkd;3* double mutant segregated progeny with both female and male gametophytic lethality, and even managed to complement these segregating defects with the *CDKD;3* gene.

Our recurrent analysis of expression patterns of native *CDKD* genes labelled by recombineering indicated that both *CDKD;1* and *CDKD;3* are expressed at notably lower levels compared with *CDKD;2* in most organs, except roots. In total protein extracts of seedlings, CDKD;1 and CDKD;3 can only be detected after enrichment on GFP‐Trap. All three CDKDs showed nuclear localization, although the most abundant CDKD;2 protein is also detectable at a low level in the cytoplasm. All three CDKDs showed co‐immunoprecipitation and nuclear co‐localization with CYCH. In comparison, the CDKD‐activating CDKF;1 kinase, as well as CYCH are detectable in both the cytoplasm and the nucleus, and their cellular localization largely overlaps. Nonetheless, CDKF;1 did not co‐immunoprecipitate with CYCH, but showed similar association with all three CDKD kinases. These results are consistent with the observation that CDKF;1, like yeast Cak1, preferentially binds to and phosphorylates the T‐loops of its cyclin‐free substrates, which then enhances further interaction of activated kinases with their cyclin partners (Tsakraklides and Solomon, 2002). Although binding to the RING domain of MAT1 can also mediate complex formation of unphosphorylated KIN28 and CDK7 with Ccl1 and CYCH, respectively, T‐loop phosphorylation of Kin28 and CDK7 is necessary for stabilization of trimeric CAK complexes in budding yeast and Drosophila (Larochelle *et al*., 2001; Keogh *et al*., 2002). This is probably the case for Arabidopsis CDKD;2 (Takatsuka *et al*., 2009), but it remains to be clarified how the degradation of CDKDs is regulated during the cell cycle and in different cell types.

### TFIIH components isolated by the CDKD;2 and CYCH baits

According to mass spectrometry data, CDKD;2, CYCH and MAT1 components of TFIIH kinase module have the highest abundance in the purified CDKD;2–GFP and CYCH–mCherry complexes. The fourth most abundant component in the CDKD;2–GFP complex is the Arabidopsis homologue of XPD DNA helicase (Table S2), which carries a redox‐sensitive 4Fe4S cluster. *Drosophila* XPD was identified to interact with the RING and α‐helical domains of MAT1 in a cytoplasmic complex (Abdulrahman *et al*., 2013; Greber *et al*., 2019), and to inhibit cell division promoting activity of the CDK7‐CYCH‐MAT1 TFIIH kinase module (Chen *et al*., 2003; Li *et al*., 2010). This interaction is disrupted by components of the cytosolic MMXD iron−sulfur assembly complex that recruit XPD from CAK to mitotic spindles regulating chromosome segregation (Ito *et al*., 2010; Houten *et al*., 2016; Nag *et al*., 2018). It is still an open question whether homologues of iron−sulfur assembly factors (Yuan *et al*., 2010; Han *et al*., 2015) would analogously regulate CAK activity of CDKD−CYCH−MAT1 complexes in Arabidopsis. Although synthesized and partly assembled in the cytoplasm, the kinase module and remaining core components of *Drosophila* TFIIH form a common complex only in the nucleus (Aguilar‐Fuentes *et al*., 2006). When targeted to the RNAPII pre‐initiation complex (PIC) by TFIIH in the nucleus, the kinase module mediates phosphorylation of Ser5 and Ser7 residues of RNAPII CTD stimulating transcription initiation. The kinase module is also directed by TFIIH to DNA damage sites. During transcription‐coupled and general genome repair, the XPD‐inhibitor kinase module is evicted from the TFIIH complex by the repair factor XPA (Coin *et al*., 2008; Compe and Egly, 2016).

Although we did not detect CDKF;1 in the complex with CYCH–mCherry, Hajheidari *et al*. (2012) demonstrated that CDKF;1 is required for *in vivo* phosphorylation of RNAPII CTD Ser7 residues, as well as for maintenance of CDKD‐dependent Ser5 CTD phosphorylation. CDKF;1 is co‐immunoprecipitated with CDKDs but not with CYCH. It is therefore possible that either targeting the C‐terminus of CYCH for pull‐down or CYCH binding to CDKDs disrupts or weakens, respectively, the interaction of CDKF;1 with the CDKD kinase module of TFIIH. In budding yeast, Cak1 is not part of the TFIIH (Kaldis *et al*., 1996) but nonetheless remains associated with RNAPII and mediates T‐loop phosphorylation and activation of Kin28, Bur1 and Ctk1 RNAPII CTD kinases (Espinoza *et al*., 1998; Kimmelman *et al*., 1999; Yao and Prelich, 2002; Ostapenko and Solomon, 2005). During PIC formation, TFIIH mediates ATP hydrolysis‐dependent opening of promoter region around the transcription start site (TSS) (Rimel and Taatjes, 2018; Kolesnikova *et al*., 2019). In the horseshoe‐like structure of TFIIH (Figure 6), which is recruited by the Mediator to PIC, the terminal XPB and XPD DNA helicases interact with each other and the MAT1 subunit of kinase module (Greber *et al*., 2019). Interaction of XPD with p62 and p44 forms one arm of the horseshoe structure whereas, in the other arm, XPB is bound to the p52, p8 and p34 TFIIH subunits. The central p44 and p34 subunits play a major role in stabilization of the complex by multiple interactions with the p62, p54 and p8 subunits (Greber *et al*., 2019). In the TFIIH holocomplex, MAT1 interacts with XPD and its activator p44, and inhibits the helicase activity of XPD (Sandrock and Egly, 2001). Upon entry into the PIC, the interaction of XPB with XPD is interrupted by TFIIE‐binding to XPB, which abolishes its contact with MAT1 and positions of the XPB‐p52‐p8 helicase module towards opening the promoter DNA. At the same time, the kinase module is displaced between the hook and shoulder positions of the Mediator close to the RNAPII CTD domain (Schilbach *et al*., 2017). This prevents inhibition of CDK7 kinase through phosphorylation of its CYCH subunit by the Mediator‐associated cyclin C‐dependent CDK8 kinase (Akoulitchev *et al*., 2000).

Comparison of representation of TFIIH subunits isolated by the help of CDKD;2–GFP and CYCH–mCherry baits (Tables S2 and S3) indicates a tight association of kinase module with XPD and its immediate binding partner p62, which mediates TFIIH interactions with numerous transcription factors and the XPC sensor of DNA lesions (Okuda *et al*., 2016). The representation of TFIIH stabilizing p44, and especially p34 subunit is surprisingly lower, similarly to their p52 and p8 interacting partners. The low abundance of latter TFIIH subunits might reflect their proximity order compared with the CDKD;2 and CYCH baits used for purification. However, the complete absence of XPB from both CDKD;2 and CYCH complexes contradicts this argumentation, as XPB is an immediate binding partner of MAT1 and XPD. From the two Arabidopsis XPB homologues, XPB2 is known to complement the yeast *rad25* DNA repair deficiency (Morgante *et al*., 2005). However, participation of XPBs in transcription‐committed TFIIH complexes is not yet confirmed experimentally. Although ATP‐dependent helicase activity of XPB is essential for transcription, recently Alekseev *et al*. (2017) demonstrated that chemical inhibition and induced degradation of XPB does not prevent RNAPII transcription, whereas XPB functions as transcription inhibitor in the absence of ATP. Further labelling of other TFIIH subunits, including XPBs by recombineering should be helpful to determine whether displacement of the XPB‐p52‐p8 arm from the kinase module can be prevented by modification of TFIIH isolation conditions. Combination of this approach with induction of DNA lesions might also be helpful to identify components of plant TFIIH‐associated DNA repair factors.

## Experimental procedures

### Plant materials, transformation and growth conditions

Wild type (Col‐0) and *cdkf;1/+* (GABI_315A10, Hajheidari *et al*., 2012) Arabidopsis plants were grown in MSAR medium (Koncz *et al*., 1994) in a controlled culture room at 22°C with 120 mol m^−2^ sec^−1^ light intensity and a photoperiod of 8 h light and 16 h darkness. Seedlings from *in vitro* cultures were transferred into soil and grown under standard greenhouse conditions (12 h light/12 h of dark period; 22–24°C day temperature and 18°C night temperature, 200 μEinstein m^−2^ sec^−1^ irradiance). For crosses and *Agrobacterium*‐mediated transformation by vacuum infiltration (Bechtold *et al*., 1993), Arabidopsis seedlings planted into soil were grown under short‐day conditions (8 h light/16 h dark) for 14–16 days and then transferred to long‐day conditions to induce flowering. T1 *cdkf;1/+* plants transformed with *CDKF;1* gene constructs carrying the GFP–PIPL, GFP and PIPL tags were self‐pollinated and the resulting T2 lines were screened for homozygous status of the SuR (sulfadiazine resistance) marker of *cdkf;1* mutation. Subsequently, the progeny of derived T3 lines were screened for homozygous status of the KmR or HygR markers of complementing *CDKF;1* gene constructs. The *CYCH–mCherry* (HygR) construct was transformed to homozygous lines carrying the *CDKF;1:GFP*,* CDKD;1:GFP* and *CDKD;3:GFP* genes linked to KmR selectable marker to isolate homozygous HygR and KmR T2 progeny. Homozygous *CYCH–mCherry* (HygR) and *CDKD;2–GFP–PIPL* (HygR) plants were crossed to identify homozygous T2 *CYCH–mCherry* lines showing 100% mCherry and segregating GFP expression in their roots, and then screened for T3 progeny carrying both markers in homozygous form.

### Construction of *ccdB* exchange and I‐*Sce*I insertion cassettes and PCR‐amplifiable binary vectors for recombineering

To construct *ccdB* cassettes, first a CmR gene linked to an I‐*Sce* I site was PCR amplified from plasmid pEL04 (Lee *et al*., 2001; primers CmF and CmR‐I‐*Sce*I, Table S1) and inserted as *Nae*I fragment into the *Nco*I site of pGEM‐T Easy (Promega). Next, the *araC*‐pBAD‐*ccdB* cassette was amplified from pSW8197 (Le Roux *et al*., 2007; primers *Spe*I‐araC and *Xba*I‐ccdB) and inserted as an *Spe*I−*Sac*I fragment into the adjacent *Xba*I−*Sac*I sites resulting in pGEM‐CmR‐araC‐ccdB (Figure S1). The CmR gene of the latter vector was removed by *Nco*I cleavage and replaced by an amplified *Bsp*HI fragment pACYC177 *KmR* gene linked to an I‐*Sce*I site (primers KmF and KmR‐I‐*Sce*I) to yield pGEM‐KmR‐araC‐ccdB. A *Sph*I−*Eco*RI fragment of the latter plasmid carrying the *KmR* gene was replaced by the *SpR* gene of pER8 linked to an I‐*Sce*I site (Zuo *et al*., 2000; primers SpF and SpR‐I‐*Sce*I) to construct pGEM‐SpR‐araC‐ccdB (Figure S1).

To assemble the coding region of PIPL tag, the Co^2+^/Ni^2+^‐binding domain of Arabidopsis CobW‐like protein (At1g15730) carrying 18 His residues was linked to two copies of the StrepII epitope separated by a Gly‐rich linker by annealing partially complementary primers P1 and P2 (Table S1). After filling in the ends with T4 DNA polymerase, the resulting fragment was extended and PCR amplified using P1 and a third partially overlapping primer P3, and cloned into the *Eco*RV site of pUC57 (Genscript). A GFP–PIPL cassette for generation of C‐terminal fusions was constructed by simultaneous insertion of the PCR‐amplified *Eco*RI−*Sal*I fragment of the GFP coding region without the stop codon (primers GFPc1F/R) and an amplified *Sal*I−*Sac*I fragment of PIPL coding region (primers PIPLc1F/R) with start and stop codons into *Eco*RI−*Sac*I sites of pBSKII. A PIPL–GFP cassette for generation of N‐terminal fusions was created by insertion an amplified (primers PIPLc2F/R) *Eco*RI−*Sal*I fragment of the PIPL coding region without a stop codon and a *Sal*I−*Sac*I fragment of GFP coding region with start but with no stop codon into *Eco*RI−*Sac*I sites of pBSKII (Figure S3).

DNA fragments amplified from pGAPHyg and pGAPKm (Bitrián *et al*., 2011) with linker primers for joining the adjacent segments (Table S1) were assembled into the binary vectors pGAPHyg2 and pGAPKm2 (Figure S5) using a Gibson assembly master mix (NEB). Upon *Sal*I cleavage, T4 DNA polymerase fill in and *Eco*RI digestion, the multicopy pUC9 replicons of the latter vectors were exchanged for a pHC79 cosmid replicon, which was isolated by *Bgl*II cleavage, fill in and *Eco*RI digestion from pPCV6NFHyg (Koncz *et al*., 1989) to construct the more stable and lower copy number binary vectors pGAPBRHyg and pGAPBRKm (Figure S5).

To construct the N‐terminal KmR–GFP–PIPL I‐*Sce*I insertion cassette, the KmR gene amplified (primers BamSceKm5 and Km3Spe; Table S1) as the *Bam*HI−I‐*Sce*I/*Spe*I fragment from pGEM‐KmR‐araC‐ccdB and the GFP–PIPL coding region amplified without a stop codon as *Spe*I−I‐*Sce*I/*Kpn*I fragment from pBSK–GFP–PIPL (primers GFPPIPL5SpeSce and GFPPIPL3Kpn) were cloned into *Bam*HI−*Kpn*I sites of pBSKII yielding pNKmR–GFP–PIPL. The *Spe*I−*Kpn*I GFP–PIPL fragment of the latter vector was replaced with the mCherry coding region amplified without stop codon as *Spe*I−I‐*Sce*I/*Kpn*I fragment from pPC‐GW–mCherry (Dalal *et al*., 2015; primers mCher5SpeISceI and mCher3KpnI) to create the pN‐KmR–mCherry cassette plasmid. The *Not*I−*Spe*I fragments of *KmR* genes in the GFP–PIPL and mCherry cassettes were replaced by a *Not*I−*Spe*I fragment of the *SpR* gene amplified from pER8 (primers NotBamHI‐SceSpF and SpectR3) to construct the cassette plasmids pN‐SpR–GFP–PIPL and N‐SpR–mCherry. The C‐terminal GFP–PIPL‐KmR and GFP‐KmR cassettes were assembled by pairwise cloning of *Bam*HI/I‐*Sce*I−*Spe*I fragments of GFP–PIPL and GFP coding regions amplified with stop codons from pBSK–GFP–PIPL (primers GFPPIPL5Bam, GFPPIPL3stopSceSpe, and GFP3stopSceSpe) with a *Spe*I/I‐*Sce*I−*Kpn*I fragment of *KmR* gene amplified from pGEM‐KmR‐araC‐ccdB (primers Km5Spe and Km3SceKpnI) into *Bam*HI−*Kpn*I sites of pBSKII. The *GFP* gene of GFP‐KmR cassette was replaced by a *Bam*HI/I‐*Sce*I−*Spe*I fragment of mCherry coding region amplified with a stop codon (primers mCher5BamHI and mCher3StopSceSpe) from pPC‐GW–mCherry yielding the C–mCherry‐KmR cassette in pBSKII. Finally, the *KmR* gene of C–GFP‐KmR and C–mCherry‐KmR cassettes was replaced by a *Spe*I/I‐*Sce*I−*Kpn*I fragment of the *SpR* gene amplified from pER8 (primers SpRSpe5 and SpRSceKpnI) to construct the C–GFP‐SpR and C–mCherry‐SpR cassettes in pBSKII. Schematic maps of insertion cassettes and their sequences are depicted, respectively, in Figure 2a and Figure S4).

### Preparatory steps of recombineering

*E. coli* strains with BAC clones carrying the studied Arabidopsis genes were obtained from the Arabidopsis Biological Research Center (ABRC) and maintained by selecting for the BAC‐encoded antibiotic resistance marker, if not stated otherwise. BAC DNA was isolated by the alkaline lysis procedure (Sambrook and Russell, 2000) following incubation of cells in buffer I (50 mm Tris–HCl (pH 8.0), 50 mm glucose, and 20 mm EDTA and 1 mg mL^−1^ lysozyme) for 30 min at 20°C. After RNase A (250 mg mL^−1^, 2 h at 37°C) and proteinase K (0.4 mg mL^−1^ overnight at 37°C) treatments, BAC DNA was subjected to phenol−chloroform extraction followed by 2‐propanol precipitation. Each BAC was verified by PCR amplification using *Taq* DNA polymerase (New England Biolabs, NEB) with two primers flanking the position of target site (i.e. stop codons) in the studied plant gene (Table S1 and Figure S2). Before *E. coli* transformation by electroporation (Dower *et al*., 1988), the BACs and PCR‐amplified DNA fragments and binary vectors were drop‐dialyzed on Millipore membrane filters (MFTM 0.025 μm VSWP) floating on sterile H_2_O. The recombineering *E. coli* host SW102 was always cultured at 32°C. After transforming with a BAC, SW102 was grown by selecting for the BAC‐encoded antibiotic resistance marker in 20 mL LB medium to OD_600_ 0.4‐0.6. For recombineering, 4 mL culture was pelleted by 8000 rpm for 1 min in a tabletop centrifuge, resuspended in 1 mL LB medium and incubated by shaking at 42°C for 15 min to induce the expression of λ*Red* genes. After centrifugation, the cells were resuspended in 1 mL ice‐cold sterile H_2_O and pelleted three times, then resuspended in 100 μl H_2_O and electroporated with linear cassette fragments or pGAPBR vectors (0.2–4 μg in 20 μl H_2_O) in a pre‐chilled electroporation cuvette (BioBudget, 2 mm) using an electropulse (at 2.5 kV [i.e., 12.5 kV cm^−1^ cuvette width], 25 μF, and 200 Ω) for 4–5 msec in a Bio‐Rad Gene Pulser.

Before starting the recombineering experiments, all necessary plasmid PCR templates were purified by caesium chloride−ethidium bromide gradient centrifugation (Sambrook and Russell, 2000). The KmR/SpR‐*ccdB* cassettes were PCR amplified using high fidelity Q5 DNA polymerase (NEB) with the corresponding forward and reverse primers (Table S1 and Figures S1 and S2), digested by *Dpn*I to cleave the contaminating template plasmid, and isolated from agarose gels after size separation. Similarly, all I‐*Sce*I insertion cassettes (Figure S4) were excised from the corresponding pBSK cassette vectors as *Bam*HI−*Kpn*I fragments and gel purified. The pGAPBR binary vectors (Figure S5) were linearized by *Bam*HI. All purified *ccdB* and I‐*Sce*I insertion cassettes and linear pGAPBR vectors were treated with alkaline phosphatase, purified with phenol‐chloroform extraction and then aliquots from them were self‐ligated and electroporated into BAC‐free SW102 to exclude the presence of contaminating cassette‐source vectors and circularized pGAPBR plasmids. The cassette fragments and *Bam*HI‐cleaved pGAPBR vectors were then used as PCR templates in the recombineering experiments.

### Recombineering with the *ccdB* exchange markers

In the first step of recombineering with the *ccdB* exchange markers (Figure 1), the KmR/SpR‐*ccdB* cassettes were PCR amplified with forward and reverse primers carrying 50‐nt flanks of the target site of plant gene using Q5 DNA polymerase (NEB) and a PCR program (98°C 30 sec, 36 cycles of 98°C 10 sec, 60°C 30 sec, and 72°C 3 min). After electroporation into heat‐induced SW102, the cells were supplemented in 1 mL LB‐0.5% glucose, incubated for 2 h at 32°C, and then plated onto LB agar containing 0.5% glucose and antibiotics to select for the resistance marker of the *ccdB* cassette. A transformant was regrown in 25 mL LB−0.5% glucose medium and plated to single colonies by selecting only for the resistance marker of the *ccdB* cassette. Using a primer pair flanking the target site, next colony PCR was performed to screen for transformants that harboured only BACs with the *ccdB* cassette but lost unmodified BACs carrying empty target sites. For colony PCR, 6–12 transformants were grown at 32°C in LB‐0.5% glucose by selecting for the antibiotics resistance marker of *ccdB* cassettes and 1 μl aliquots of these cultures were used as templates in PCR reactions with *Taq* polymerase (1 U, NEB) in 20 μl buffer (1× PCR buffer (NEB), 0.25 mm dNTP, 1 μm gene‐specific flanking primers) and a PCR program (95°C 30 sec, 35 cycles of 95°C 15 sec, 60°C 30 sec and 68°C 2 min followed by 68°C 2 min).

In the second step of recombineering, the *ccdB* cassette was replaced with GFP‐(PIPL) tags (Figures S2 and S3). The PCR amplified tag fragments were electroporated into heat‐induced SW102 that carried only BACs with the confirmed *ccdB* cassette. Following incubation in 1 mL LB medium for 2 h at 32°C, half of the cells were plated on LB−0.2% arabinose plates and the other half was inoculated in 20 mL LB‐0.2% arabinose liquid culture and plated next day by selecting for the antibiotic resistance marker of BAC in both cases. The loss of antibiotic resistance marker of *ccdB* cassette was confirmed by replica plating of colonies and subsequent colony PCR either with primers flanking the target sites or with tag‐specific primers or combinations of both.

The third gap‐repair step of recombineering was performed either as outlined below for the I‐*Sce*I insertion cassettes, or using the pGAP vectors as described (Bitrián *et al*., 2011). In the latter case, two segments of BACs flanking the modified plant genes were PCR amplified as *Eco*RI−*Bam*HI and *Bam*HI−*Sal*I fragments and after digestion with the corresponding enzymes and gel purification were cloned into *Eco*RI−*Sal*I sites of the binary vectors. Before the gap‐repair step, these vectors were linearized with *Sal*I, treated with alkaline phosphatase and tested for the absence of self‐ligation. After transforming the linear pGAP or pGAPBR vectors into the SW102 host harbouring a BAC with a tag replacing the *ccdB* markers, the clones in the gap‐repair step were selected for their AmpR marker.

Sequences of pGAP vectors and modified versions of *CDKF;1*,* CDKD;1*,* CDKD;2* and *CDKD;3* genes cloned by gap‐repair between *Eco*RI and *Bam*HI sites of pGAPs are depicted in Figure S2, which indicates the positions of flanks and all primers used for recombineering with the *ccdB* cassettes. The corresponding PCR primers are listed in Table S1.

### Recombineering with the I‐*Sce*I insertion cassettes

In the first step of recombineering purified *Bam*HI−*Kpn*I fragments (2 ng in 50 μl) of the I‐*Sce*I insertion cassettes were PCR amplified with the corresponding forward and reverse primers carrying 50‐nt flanks of translation start or stop codons of target plant genes (Figure S4 and Table S1) using Q5 *Taq* polymerase (NEB) and a program (98°C 30 sec, 36 cycles of 98°C 10 sec, 71°C 30 sec and 72°C 1 min, followed by 72°C 3 min). After testing an aliquot of PCR products by electrophoresis, the amplified DNA fragments were desalted and electroporated into SW102 (BAC) cells. Following incubation in 1 mL LB at 32°C for 2 h, the cells were plated on LB agar by selecting only for the antibiotic resistance marker of the I‐*Sce*I insertion cassette. Two to three transformants were tested by colony PCR with gene‐specific primers flanking the target site, which detected BACs with the insertion cassettes, as well as unmodified empty BACs.

In the second step, linearized pGAPBR vectors were PCR amplified in a 50‐μl volume with forward and reverse primers carrying 50‐nt flanks (1 μM) marking the boundaries of BAC region, which was destined to be transferred into plants. The pGAPBR reverse primer carrying the *Spe*I site (Figures S2, S5 and Table S1) was always linked to reverse complement of the flank sequence. The pGAPBR vectors were amplified using Q5 DNA polymerase with enhancer‐containing NEB buffer and the following program: 98°C 30 sec, 35 cycles of 98°C 10 sec; 60°C 30 sec; 72°C 10 min followed by 72°C for 5 min. The amplified pGAPBR vectors were size separated, gel isolated and transformed into SW102, which was grown by selecting for the antibiotics marker of the I‐*Sce*I cassette insertion in the BAC. Following incubation of electroporated cells in 0.5 mL LB at 32°C for 2 h, AmpR KmR or AmpR SpR (ampicillin 100 mg L^−1^, kanamycin 25 mg L^−1^, and spectinomycin 50 mg L^−1^) transformants were selected on LB medium at 32°C and grown up for plasmid DNA isolation using a NucleoSpin Plasmid kit (Macherey‐Nagel GmbH, Düren, Germany). The resulting plasmid DNA was transformed into *E. coli* DH5α or DH10B followed by selecting for AmpR KmR or AmpR SpR colonies. From one to three transformants plasmid DNA was recurrently isolated and fingerprinted with restriction enzymes to confirm the expected structure of recombinant pGAPBR carrying the designed plant DNA region.

In the third step, the pGAPBR plasmid carrying the target plant gene with the I‐*Sce*I insertion cassette was digested with I‐*Sce*I (NEB) to remove the antibiotic resistance gene, and then self‐ligated and transformed into *E. coli* DH10B followed by selection for AmpR transformants and screening for the loss of the antibiotic resistance gene of the insertion cassette (i.e., kanamycin or spectinomycin sensitivity). The junctions of tags placed by recombineering into the plant genes were verified by sequencing of the pGAPBP clones by gene‐specific primer pairs flanking the tags in the target sites. Sequences of modified *CYCLIN H* (*CYCH*) and *HISTONE H3;1* genes are depicted by marking the positions of all primers used for recombineering with the I‐*Sce*I cassettes in Figure S2, and the primers are listed in Table S1.

The verified pGAP clones were then transformed into the RK2 conjugation helper *E. coli* donor strain MFDpir ΔTIV lacIq (JKE201, kindly provided by C. Dehio, Biozentrum, University of Basel) and conjugated into *Agrobacterium* recipient GV3101 (pMP90RK) at 28°C for 36 h by placing drops of a 1:1 mix of donor and recipient onto LB agar plates containing 0.3 mm DAP (2,6‐diaminopimelic acid). A loop of conjugation mix was streaked out on YEB‐agar containing 100 mg L^−1^ rifampicin (Rif100) and 100 mg L^−1^ carbenicillin (Cb100) to isolate GV3101 (pMP90RK) transconjugants carrying the binary vectors. For testing the integrity of pGAP(BR) clones in *Agrobacterium*, the plasmids were back‐conjugated into *E. coli*. The *Agrobacterium* donor was grown in LB‐Cb100 medium, whereas the *E. coli* DH5α recipient in LB at 28°C. After mixing the donor and recipient, drops of the conjugation mix were incubated on an LB agar plate for 36 h at 28°C. A loop from the conjugation spots was streaked out onto LB‐Cb100 plates to grow up single *E. coli* colonies at 37°C, where *Agrobacterium* growth was inhibited.

All recombineering tools described in this paper are submitted to and will be distributed by the Arabidopsis Biological Resource Center.

### Confocal laser‐scanning microscopy

The localization of GFP‐ and mCherry‐labelled proteins in fresh tissue samples was captured by a Leica TCS SP8 confocal microscope (Leica, Bensheim, Germany). GFP was excited with Argon laser at 488 nm and the emitted fluorescence was detected between 493 and 550 nm. mCherry was excited at 561 nm and the emitted fluorescence was detected between 576 and 632 nm. Homozygous transgenic plants carrying the *CDKF;1–GFP–PIPL*,* CDKD;1–GFP*,* CDKD;2–GFP–PIPL*,* CDKD;3–GFP* and *CYCH–GFP* constructs were germinated on vertical MSAR agar plates and grown for 10 days in a controlled culture room. The roots of seedlings were stained for 30 sec with 0. 01% PI (propidium iodide, Sigma‐Aldrich) followed by washing several times with water. GFP and PI fluorescence of root tips was captured by a Leica TCS SP8 confocal microscope. PI was excited at 488 and the emitted fluorescence was detected between 600 and 657 nm. Merging of images was performed using the Leica LAS X software. Line intensity profiles of selected regions of interest (ROI) were generated using the Image J2 software (Rueden *et al*., 2017) and the data were exported to Excel.

### Immunoblotting and co‐immunoprecipitation of GFP‐ and mCherry‐labelled proteins

Total protein extracts from 14‐day‐old short‐day grown seedlings were prepared in extraction buffer (50 mm Tris–HCl (pH 7.5), 10% glycerol, 1 mm EDTA, 150 mm NaCl, 1 mm PMSF and 20 μm Sigma plant protease inhibitor cocktail). Following measurement of protein concentrations of tissue extracts (Bradford, 1976), 25 μg aliquots of protein samples were subjected to size separation by SDS‐PAGE electrophoresis and electro‐transferred onto PVDF membranes (Merck [Millipore] Darmstadt, Germany). The GFP‐ and mCherry‐labelled proteins were detected by ChromoTek rat monoclonal primary 3H9 anti‐GFP (dilution 1:1000) and mouse monoclonal 6G6 anti‐RFP (dilution 1:1000) antibodies in TBST buffer (25 mm Tris–HCl [pH7.5], 0.15 m NaCl, 0.05% and 5% milk powder). After washing with TBST, proteins were visualized by incubation with horseradish peroxidase conjugated goat anti‐rabbit (1:20 000; Vector PI‐1000) and anti‐mouse (1:10 000; Thermo Fisher Scientific GmbH, Dreieich, Germany) secondary antibodies followed detection by enhanced chemoluminescence (ECL) and autoradiography.

For immunoprecipitation, 5 g of 14‐day‐old seedlings grown in MSAR medium in Petri dishes under short‐day condition were harvested and ground to a fine power in liquid nitrogen using 10 mL of EBW extraction buffer (50 mm Tris–HCl (pH 7.5), 10% glycerol, 1 mm EDTA, 150 mm NaCl, 5 mm dithiothreitol (DTT), 20 μm plant protease inhibitor cocktail, 1 mm PMSF). After thawing on ice, the crude extract was subjected to centrifugation in a Beckmann JA20 rotor at 12 000 rpm (17 000 ***g***) for 20 min at 4°C. The supernatant was moved into a 15‐mL Falcon tube and protein concentration was measured by Bradford assay (Bradford, 1976). 50 μl GFP‐Trap Agarose (ChromoTek GmbH, Planegg‐Martinsried, Germany) were added to aliquots of 20 mg total protein and incubated for 2 h at 4°C in the cold room. Next, the GFP‐Trap resin was pelleted by centrifugation (at 500 rpm, Heraeus centrifuge) at 4°C and washed three times with 10 mL washing buffer (50 mm Tris–HCl (pH 7.5), 300 mm NaCl) followed each time by centrifugation under the latter condition. Finally, the GFP‐Trap resin was suspended in 1 mL washing buffer and transferred into 1.5‐mL Eppendorf tubes. The beads were boiled for 5 min in 30 μl 1× Laemmli‐buffer (63 mm Tris–HCl (pH 6.8), 10% glycerol, 2% SDS, 0.1% 2‐mercaptoethanol, 0.0005% bromophenol blue) and 20 μl aliquots of supernatant samples were subjected to western blotting as described above.

### Isolation of CDKD;2:GFP, CYCH:mCherry and HISTONE H3.1:mCherry complexes

Protein extracts from 15 g rosette materials of 3‐week‐old greenhouse grown seedlings ground in liquid nitrogen were prepared using 30 mL of EBW extraction buffer (see above). After centrifugation in a Beckmann JA20 rotor at 12 000 rpm (about 17 000 ***g***) for 20 min at 4°C, 10 mL supernatants of three equal biological replicates, each containing 20 mg protein, were incubated for 2 h at 4°C with 50 μl GFP‐Trap agarose (ChromoTek) or RFP‐Trap agarose (ChromoTek) resin, which was previously washed three times with 1.5 mL extraction buffer and added in 100 μl EBW to the protein extracts in Falcon tubes. Subsequently, the GFP‐Trap or RFP‐Trap resins were pelleted (500 rpm, Heraeus centrifuge) at 4°C and washed three times with 10 mL washing buffer (50 mm Tris–HCl (pH 7.5), 300 mm NaCl). Finally, the GFP‐Trap beads were suspended in 1 mL washing buffer, transferred into a 1.5‐mL Eppendorf tube, pelleted by centrifugation, and the bound proteins were eluted by 50 μl 0.1% trifluoroacetic acid (TFA) followed by immediate neutralization of the solution by addition of 8 μl 1 m Tris base. The RFP‐Trap beads were similarly treated but the bound proteins were directly subjected to on‐bead trypsin digestion prior LC‐MS/MS analysis. As controls, three biological replicates of protein extracts were prepared from wild type (Col‐0) and 35S:StrepII‐3xHA‐YFP (Lapin *et al*., 2019) expressing plants of the same age, harvested and processed in parallel with the CDKD;2–GFP‐, CYCH–mCherry‐ and HISTONE H3.1‐expressing plants.

### Sample preparation for mass spectrometry and LC‐MS/MS data acquisition

Proteins eluted from GFP‐Trap resins were reduced with dithiothreitol (DTT), alkylated with chloroacetamide (CAA), and digested with trypsin. Next, the samples were desalted using StageTips with C18 Empore disc membranes (3M; Rappsilber *et al*., 2003), dried in a vacuum evaporator, and dissolved in 2% ACN (acetonitrile), 0.1% TFA (trifluoroacetic acid). The RFP‐Trap‐bound proteins associated with CYCH:mCherry and HISTONE H3.1:mCherry were subjected to an on‐bead digestion. In brief, dry beads were resuspended in 25 μl digestion buffer 1 (50 mm Tris–HCl (pH 7.5), 2 m urea, 1 mm DTT, 5 μg μl^−1^ trypsin), incubated for 30 min at 30°C in a thermomixer with 400 rpm, and then pelleted and the supernatant was transferred to a fresh tube. Digestion buffer 2 (50 mm Tris–HCl (pH 7.5), 2 m urea, 5 mm CAA) was added. After mixing the beads were pelleted and the supernatant was collected and combined with the previous one. The combined supernatants were then incubated overnight at 32°C in a thermomixer at 400 rpm and protecting the samples from light. The digestion was stopped by adding 1 μl TFA followed by desalting the samples with C18 Empore disc membranes according to the StageTip protocol (Rappsilber *et al*., 2003).

Dried peptides were re‐dissolved in 10 μl of 2% ACN, 0.1% TFA and adjusted to a final concentration of 0.1 μg μl^−1^, or measured without dilution for the on‐bead digested samples. Samples were analyzed using an EASY‐nLC 1200 system (ThermoFisher) coupled to a Q Exactive Plus mass spectrometer (ThermoFisher). Peptides were separated on 16‐cm frit‐less silica emitters (New Objective, 0.75 μm inner diameter), packed in‐house with reversed‐phase ReproSil‐Pur C18 AQ 1.9 μm resin (Dr. Maisch GmbH, Ammerbuch‐Entringen, Germany). Peptides (0.5 μg) were loaded on the column and eluted for 115 min using a segmented linear gradient of 5–95% solvent B (0 min: 5%B; 0–5 min: 5%B; 5–65 min: 20%B; 65–90 min: 35%B; 90–100 min: 55%; 100–105 min: 95%, 105–115 min: 95%) (solvent A 0% ACN, 0.1% FA; solvent B 80% ACN, 0.1%FA) at a flow rate of 300 nl min^−1^. Mass spectra were acquired in data‐dependent acquisition mode with a TOP15 method. MS spectra were acquired in the Orbitrap analyzer with a mass range of 300–1750 m/z at a resolution of 70 000 FWHM and a target value of 3 × 10^6^ ions. Precursors were selected with an isolation window of 1.3 m/z. Higher‐energy collisional dissociation (HCD) fragmentation was performed at normalized collision energy of 25. MS/MS spectra were acquired with a target value of 10^5^ ions at a resolution of 17 500 FWHM, a maximum injection time (max.) of 55 msec and a fixed first mass of m/z 100. Peptides with a charge of +1, greater than 6, or with unassigned charge state were excluded from fragmentation for MS2, dynamic exclusion for 30 sec prevented repeated selection of precursors.

### LC‐MS/MS data analysis

Raw data were processed using MaxQuant software (version 1.5.7.4, http://www.maxquant.org/, Cox and Mann, 2008) with label‐free quantification (LFQ) and iBAQ enabled (Tyanova *et al*., 2016). MS/MS spectra were searched by the Andromeda search engine against a combined database containing the sequences from *A. thaliana* (TAIR10_pep_20101214; ftp://ftp.arabidopsis.org/home/tair/Proteins/TAIR10_protein_lists/) and sequences of 248 common contaminant proteins and decoy sequences. Trypsin specificity was required and a maximum of two missed cleavages allowed. Minimal peptide length was set to seven amino acids. Carbamidomethylation of cysteine residues was set as fixed, oxidation of methionine and protein N‐terminal acetylation as variable modifications. Peptide‐spectrum‐matches and proteins were retained if they were below a false discovery rate of 1%. Statistical analysis of the MaxLFQ values was carried out using Perseus (version 1.5.8.5, http://www.maxquant.org/). Quantified proteins were filtered for reverse hits and hits ‘identified by site’ and MaxLFQ values were log2 transformed. After grouping samples by condition only those proteins were retained for the subsequent analysis that had two valid values in one of the conditions. Two‐sample *t*‐tests were performed with a *P*‐value cutoff of 5%. Alternatively, quantified proteins were grouped by condition and only those hits were retained that had three valid values in one of the conditions. Missing values were imputed from a normal distribution, using the default settings in Perseus (1.8 downshift, separately for each column). Volcano plots were generated in Perseus using an FDR of 5% and an S_0_ = 1. The Perseus output was exported and further processed using Excel. Data from the LC‐MS/MS analysis obtained for the CDKD;2:GFP (Acc. No. 171005), CYCH:mCherry and HISTONE H3.1:mCherry (Acc. No. 190227) samples were deposited in the PRIDE archive (https://www.ebi.ac.uk/pride/archive/) under the project accession number PXD013637.

## Accession numbers

CDKF;1 (At4g28980), CDKD;1 (At1g73690), CDKD;2 (At1g66750), CDKD;3 (At1g18040), CYCH (At5g27620), MAT1 (AT4G30820), XPB1 (AT5G41370), XPB2 (AT5G41360), XPD (AT1G03190), p62‐1 (AT3G61420), p62‐2 (AT1G55750), p52 (AT4G17020), p44 (AT1G05055), p34 (AT1G18340), p8‐1 (AT1G12400), p8‐2 (AT1G62886), HISTONE H3.1 (AT5G65360), HISTONE H4 (AT5G59970), UBR7 PHD finger (AT4G23860), FAS1 (AT1G65470), FAS2 (AT5G64630), MSI (AT5G58230), ASF1A (AT1G66740), ASF1B (AT5G38110), NASP (AT4G37210), ATJ6 (AT5G06910), DnaJ domain (AT3G12170), Ku70 (AT1G16970), and Ku80 (AT1G48050).

## Conflict Of Interest

The authors have no conflicts of interest to declare.

## Authors’ Contributions

ZH, AG, MHo, SchS, BB and SZ performed recombineering experiments planned by CK, ZH carried out western blot and co‐immunoprecipitation studies and purified the protein complexes, SCS, AH and HN accomplished the LC‐MS/MS mass spectrometry measurements and data analyses, ZH and SL contributed to the confocal microscopy studies, MHo, MHa and TJS prepared the PIPL epitope tag, CK, ZK and ZH wrote the manuscript, which was corrected and amended by all authors.

## Supporting information


**Figure S1.** Arrangement of antibiotic resistance, *araC* and *ccdB* gene sequences in the *ccdB* cassettes.

**Figure S2.** Example templates for planning the recombineering experiments and sequences of modified plant genes in pGAP and pGAPBR vectors constructed in this work.

**Figure S3.** Nucleotide and amino acid sequences of GFP–PIPL and PIPL–GFP tags.

**Figure S4.** Nucleotide sequences of N‐terminal and C‐terminal I‐*Sce*I insertion cassettes.

**Figure S5.** Gibson assembly and sequences of pGAPBRHyg and pGAPBRKm binary vectors.

**Figure S6.** Localization of CDKF;1:GFP, CDKD;1:GFP, CDKD2:GFP–PIPL, CDKD;3:GFP, CYCH:GFP; CYCH:mCherry and HISTONE H3.1:mCherry proteins in roots, lateral roots, hypocotyls and primary leaves of 7‐day‐old seedlings by confocal microscopy.

**Figure S7.** Localization of CDKF;1:GFP, CDKD;1:GFP, CDKD2:GFP–PIPL, CDKD;3:GFP, CYCH:GFP; CYCH:mCherry and HISTONE H3.1:mCherry proteins in pollens, pistil tissues and seed coat.

**Table S1.** List of oligonucleotide PCR primers.

**Table S2.** Perseus output of MaxQuant analysis data of TFIIH subunits detected in association with CDKD;2:GFP compared with YFP expressing and wild type controls (PRIDE PXD013637_171005).

**Table S3.** Perseus output of MaxQuant analysis data of TFIIH subunits detected in association with CYCH:mCherry compared with H3:1:mCherry expressing and wild type controls (PRIDE PXD013637_190227).
