# Supplementary material for: Gene modification by fast‐track recombineering for cellular localization and isolation of components of plant protein complexes
Source: Plant J. 2019 Jul 26;100(2):411–29. doi: 10.1111/tpj.14450 (PMC6852550; doi:10.1111/tpj.14450)
Supplement: Supplementary file 1 — Figure S1. Arrangement of antibiotic resistance, araC and ccdB gene sequences in the ccdB cassettes. [file TPJ-100-411-s001.docx]

**(a)**


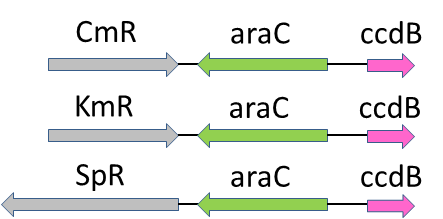


**(b)**

gggcgaattgggcccgacgtcgcatgctcccggccgccatgggcc**ctgtgacggaagatc** 60 **CmRF**

**acttcgcaga**ataaataaatcctggtgtccctgttgataccgggaagccctgggccaact 120

tttggcgaaaatgagacgttgatcggcacgtaagaggttccaactttcaccataatgaaa 180 CmseqR2

taagatcactaccgggcgtattttttgagttatcgagattttcaggagctaaggaagcta 240

aa**ATG**GAGAAAAAAATCACTGGATATACCACCGTTGATATATCCCAATGGCATCGTAAAG 300

AACATTTTGAGGCATTTCAGTCAGTTGCTCAATGT**ACCTATAACCAGACCGTTCAGCTGG** 360 CmseqR1

**AT**ATTACGGCCTTTTTAAAGACCGTAAAGAAAAATAAGCACAAGTTTTATCCGGCCTTTA 420

TTCACATTCTTGCCCGCCTGATGAATGCTCATCCGGAATTCCGTATGGCAATGAAAGACG 480 **CmR**

GTGAGCTGGTGATATGGGATAGTGTTCACCCTTGTTACACCGTTTTCCATGAGCAAACTG 540

AAACGTTTTCATCGCTCTGGAGTGAATACCACGACGATTTCCGGCAGTTTCTACACATAT 600

ATTCGCAAGATGTGGCGTGTTACGGTGAAAACCTGGCCTATTTCCCTAAAGGGTTTATTG 660

AGAATATGTTTTTCGTCTCAGCCAATCCCTGGGTGAGTTTCACCAGTTTTGATTTAAACG 720

TGGCCAATATGGACAACTTCTTCGCCCCCGTTTTCACCATGGGCAAATATTATACGCAAG 780

GCGACAAGGTGCTGATGCCGCTGGCGATTCAGGTTCATCATGCCGTTTGTGATGGCTTCC 840

ATGTCGGCAGAATGCTTAATGAATTACAACAGTACTGCGATGAGTGGCAGGGCGGGGCG**T** 900

**AA**tttttttaaggcagttattggtgcccttaaacgcctggttgctacgcctgaataagtg 960

ataataagcggatgaatggcagaaattcgaaa**CTATATTACCCTGTTATCCCTAGCGTA**a 1020 **I-SceI**

ctgcccatggcggccgcgggaattcgatatcactagagccgtcaattgtctgattcgtta 1080

ccaa**TTA**TGACAACTTGACGGCTACATCATTCACTTTTTCTTCACAACCGGCACGAAACT 1140

CGCTCGGGCTGGCCCCGGTGCATTTTTTAAATACTCGCGAGAAATAGAGTTGATCGTCAA 1200

AACCAACATTGCGACCGACGGTGGCGATAGGCATCCGGGTAGTGCTCAAAAGCAGCTTCG 1260

CCTGACTAATGCGTTGGTCCTCGCGCCAGCTTAAGACGCTAATCCCTAACTGCTGGCGGA 1320

AAAGATGTGACAGACGCGACGGCGACAAGCAAACATGCTGTGCGACGCTGGCGATATCAA 1380

AATTGCTGTCTGCCAGGTGATCGCTGATGTACTGACAAGCCTCGCGTACCCGATTATCCA 1440

TCGGTGGATGGAGCGACTCGTTAATCGCTTCCATGCGCCGCAGTAACAATTGCTCAAGCA 1500 **araC**

GATTTATCGCCAGCAGCTCCGAATAGCGCCCTTCCCCTTGCCCGGCGTTAATGATTTGCC 1560

CAAACAGGTCGCTGAAATGCGGCTGGTGCGCTTCATCCGGGCGAAAGAAACCCGTATTGG 1620

CAAATATTGACGGCCAGTTAAGCCATTCATGCCAGTAGGCGCGCGGACGAAAGTAAACCC 1680

ACTGGTGATACCATTCGCGAGCCTCCGGATGACGACCGTAGTGATGAATCTCTCCTGGCG 1740

GGAACAGCAAAATATCACCCGGTCGGCAGACAAATTCTCGTCCCTGATTTTTCACCACCC 1800

CCTGACCGCGAATGGTGAGATTGAGAATATAACCTTTCATTCCCAGCGGTCGGTCGATAA 1860

AAAAATCGAGATAACCGTTGGCCTCAATCGGCGTTAAACCCGCCACCAGATGGGCGTTAA 1920

ACGAGTATCCCGGCAGCAGGGGATCATTTTGCGCTTCAGC**CAT**acttttcatactcccac 1980

cattcagagaagaaaccaattgtccatattgcatcagacattgccgtcactgcgtctttt 2040

actggctcttctcgctaacccaaccggtaaccccgcttattaaaagcattctgtaacaaa 2100

gcgggaccaaagccatgacaaaaacgcgtaacaaaagtgtctataatcacggcagaaaag 2160

tccacattgattatttgcacggcgtcacactttgctatgccatagcatttttatccataa 2220

gattagcGGATCCtacctgacgctttttatcgcaactctctactgtttctccatacccgt 2280

ttttttggatggagtgaaacg**ATG**CAGTTTAAGGTTTACACCTATAAAAGAGAGAGCCGT 2340

TATCGTCTGTTTGTGGATGTACAGAGTGATATTATTGACACGCCCGGGCGACGGATGGTG 2400 **ccdB**

ATCCCCCTGGCCAGTGCACGTCTGCTGTCAGATAAAGTCTCCCGTGAACTTTACCCGGTG 2460 ccdBseqF1

GTGCATATCGGGGATGAAAGCTGGCGCATGATGACCACCGATATGGCCAGTGTGCCGGTC 2520 ccdBseqF2

TCCGTTATCGGGGAAGAAGTGGCTGATCTCAGCCACCGCGAAAATGACATCAAAAACGCC 2580

ATTAA**CCTGATGTTCTGGGGAATATAAgagctc**ccaacgcgttggatgcatagcttgagt 2640 **ccdBR**

attctatagtgtcacctaaatagcttggcgtaatcatggtcatagctgtttcctgtgtga 2700

**(c)**

gggcgaattgggcccgacgtcgcatgctcccggccgc**catgggccacgttgtgtctcaa**a 60 **KmF**

atctctgatgttacattgcacaagataaaaatatatcatcatgaacaataaaactgtctg 120

cttacataaacagtaatacaaggggtgtt**ATG**AGCCATATTCAACGGGAAACGTCTTGCT 180 KmseqR2

CGAGGCCGCGATTAAATTCCAACATGGATGCTGATTTATATGGGTATAAATGGGCTCGCG 240

ATAATGTCGGGCAATCAGGTGCGACAATCTATCGATTGTATGGGAAGCCCGATGCGCCAG 300 KmseqR1

AGTTGTTTCTGAAACATGGCAAAGGTAGCGTTGCCAATGATGTTACAGATGAGATGGTCA 360

GACTAAACTGGCTGACGGAATTTATGCCTCTTCCGACCATCAAGCATTTTATCCGTACTC 420

CTGATGATGCATGGTTACTCACCACTGCGATCCCCGGGAAAACAGCATTCCAGGTATTAG 480

AAGAATATCCTGATTCAGGTGAAAATATTGTTGATGCGCTGGCAGTGTTCCTGCGCCGGT 540 **KmR**

TGCATTCGATTCCTGTTTGTAATTGTCCTTTTAACAGCGATCGCGTATTTCGTCTCGCTC 600

AGGCGCAATCACGAATGAATAACGGTTTGGTTGATGCGAGTGATTTTGATGACGAGCGTA 660

ATGGCTGGCCTGTTGAACAAGTCTGGAAAGAAATGCATAAGCTTTTGCCATTCTCACCGG 720

ATTCAGTCGTCACTCATGGTGATTTCTCACTTGATAACCTTATTTTTGACGAGGGGAAAT 780

TAATAGGTTGTATTGATGTTGGACGAGTCGGAATCGCAGACCGATACCAGGATCTTGCCA 840

TCCTATGGAACTGCCTCGGTGAGTTTTCTCCTTCATTACAGAAACGGCTTTTTCAAAAAT 900

ATGGTATTGATAATCCTGATATGAATAAATTGCAGTTTCATTTGATGCTCGATGAGTTTT 960

TC**TAA**tcagaattggttaattggttgtaacactggcagagcattacgctgacttgacggg 1020

acgg**CTATATTACCCTGTTATCCCTAGCGTA**ACTCcatggcggccgcgggaattcgatat 1080 **I-SceI**

cactagagccgtcaattgtctgattcgttaccaa**TTA**TGACAACTTGACGGCTACATCAT 1140

TCACTTTTTCTTCACAACCGGCACGAAACTCGCTCGGGCTGGCCCCGGTGCATTTTTTAA 1200

ATACTCGCGAGAAATAGAGTTGATCGTCAAAACCAACATTGCGACCGACGGTGGCGATAG 1260

GCATCCGGGTAGTGCTCAAAAGCAGCTTCGCCTGACTAATGCGTTGGTCCTCGCGCCAGC 1320

TTAAGACGCTAATCCCTAACTGCTGGCGGAAAAGATGTGACAGACGCGACGGCGACAAGC 1380

AAACATGCTGTGCGACGCTGGCGATATCAAAATTGCTGTCTGCCAGGTGATCGCTGATGT 1440

ACTGACAAGCCTCGCGTACCCGATTATCCATCGGTGGATGGAGCGACTCGTTAATCGCTT 1500 **araC**

CCATGCGCCGCAGTAACAATTGCTCAAGCAGATTTATCGCCAGCAGCTCCGAATAGCGCC 1560

CTTCCCCTTGCCCGGCGTTAATGATTTGCCCAAACAGGTCGCTGAAATGCGGCTGGTGCG 1620

CTTCATCCGGGCGAAAGAAACCCGTATTGGCAAATATTGACGGCCAGTTAAGCCATTCAT 1680

GCCAGTAGGCGCGCGGACGAAAGTAAACCCACTGGTGATACCATTCGCGAGCCTCCGGAT 1740

GACGACCGTAGTGATGAATCTCTCCTGGCGGGAACAGCAAAATATCACCCGGTCGGCAGA 1800

CAAATTCTCGTCCCTGATTTTTCACCACCCCCTGACCGCGAATGGTGAGATTGAGAATAT 1860

AACCTTTCATTCCCAGCGGTCGGTCGATAAAAAAATCGAGATAACCGTTGGCCTCAATCG 1920

GCGTTAAACCCGCCACCAGATGGGCGTTAAACGAGTATCCCGGCAGCAGGGGATCATTTT 1980

GCGCTTCAGC**CAT**acttttcatactcccaccattcagagaagaaaccaattgtccatatt 2040

gcatcagacattgccgtcactgcgtcttttactggctcttctcgctaacccaaccggtaa 2100

ccccgcttattaaaagcattctgtaacaaagcgggaccaaagccatgacaaaaacgcgta 2160

acaaaagtgtctataatcacggcagaaaagtccacattgattatttgcacggcgtcacac 2220

tttgctatgccatagcatttttatccataagattagcggatcctacctgacgctttttat 2280

cgcaactctctactgtttctccatacccgtttttttggatggagtgaaacg**ATG**CAGTTT 2340

AAGGTTTACACCTATAAAAGAGAGAGCCGTTATCGTCTGTTTGTGGATGTACAGAGTGAT 2400 **ccdB**

ATTATTGACACGCCCGGGCGACGGATGGTGATCCCCCTGGCCAGTGCACGTCTGCTGTCA 2460 ccdBseqF1

GATAAAGTCTCCCGTGAACTTTACCCGGTGGTGCATATCGGGGATGAAAGCTGGCGCATG 2520 ccdBseqF2

ATGACCACCGATATGGCCAGTGTGCCGGTCTCCGTTATCGGGGAAGAAGTGGCTGATCTC 2580

AGCCACCGCGAAAATGACATCAAAAACGCCATTAA**CCTGATGTTCTGGGGAATATAAgag** 2640 **ccdBR**

**ctc**ccaacgcgttggatgcatagcttgagtattctatagtgtcacctaaatagcttggcg 2700

**(d)**

gggcgaattgggcccgacgtcg**catgcCTCATGTTACCGATGCTATTC**GGAAGAACGGCA 60 **SpF**

ACTAAGCTGCCGGGTTTGAAACACGGATGATCTCGCGGAGGGTAGCATGTTGATTGTAAC 120

GATGACAGAGCGTTGCTGCCTGTGATCAATTCGGGCACGAACCCAGTGGACATAAGCCTC 180 SpseqR2

GTTCGGTTCGTAAGCTGTAATGCAAGTAGCGTAACTGCCGTCACGCAACTGGTCCAGAAC 240 SpseqR1

CTTGACCGAACGCAGCGGTGGTAACGGCGCAGTGGCGGTTTTCATGGCTTCTTGTT**ATG**A 300

CATGTTTTTTTGGGGTACAGTCTATGCCTCGGGCATCCAAGCAGCAAGCGCGTTACGCCG 360

TGGGTCGATGTTTGATGTTATGGAGCAGCAACGATGTTACGCAGCAGGGCAGTCGCCCTA 420

AAACAAAGTTAAACATCATGGGGGAAGCGGTGATCGCCGAAGTATCGACTCAACTATCAG 480

AGGTAGTTGGCGTCATCGAGCGCCATCTCGAACCGACGTTGCTGGCCGTACATTTGTACG 540

GCTCCGCAGTGGATGGCGGCCTGAAGCCACACAGTGATATTGATTTGCTGGTTACGGTGA 600

CCGTAAGGCTTGATGAAACAACGCGGCGAGCTTTGATCAACGACCTTTTGGAAACTTCGG 660 **SpR**

CTTCCCCTGGAGAGAGCGAGATTCTCCGCGCTGTAGAAGTCACCATTGTTGTGCACGACG 720

ACATCATTCCGTGGCGTTATCCAGCTAAGCGCGAACTGCAATTTGGAGAATGGCAGCGCA 780

ATGACATTCTTGCAGGTATCTTCGAGCCAGCCACGATCGACATTGATCTGGCTATCTTGC 840

TGACAAAAGCAAGAGAACATAGCGTTGCCTTGGTAGGTCCAGCGGCGGAGGAACTCTTTG 900

ATCCGGTTCCTGAACAGGATCTATTTGAGGCGCTAAATGAAACCTTAACGCTATGGAACT 960

CGCCGCCCGACTGGGCTGGCGATGAGCGAAATGTAGTGCTTACGTTGTCCCGCATTTGGT 1020

ACAGCGCAGTAACCGGCAAAATCGCGCCGAAGGATGTCGCTGCCGACTGGGCAATGGAGC 1080

GCCTGCCGGCCCAGTATCAGCCCGTCATACTTGAAGCTAGACAGGCTTATCTTGGACAAG 1140

AAGAAGATCGCTTGGCCTCGCGCGCAGATCAGTTGGAAGAATTTGTCCACTACGTGAAAG 1200

GCGAGATCACCAAGGTAGTCGGCAAA**TAA**tgtctagctagaaattcgttcaagccgacgc 1260

cgcttcgccgaagtg**CTATATTACCCTGTTATCCCTAGCGTA**gaattcgatatcactaga 1320

gccgtcaattgtctgattcgttaccaa**TTA**TGACAACTTGACGGCTACATCATTCACTTT 1380

TTCTTCACAACCGGCACGAAACTCGCTCGGGCTGGCCCCGGTGCATTTTTTAAATACTCG 1440

CGAGAAATAGAGTTGATCGTCAAAACCAACATTGCGACCGACGGTGGCGATAGGCATCCG 1500

GGTAGTGCTCAAAAGCAGCTTCGCCTGACTAATGCGTTGGTCCTCGCGCCAGCTTAAGAC 1560

GCTAATCCCTAACTGCTGGCGGAAAAGATGTGACAGACGCGACGGCGACAAGCAAACATG 1620

CTGTGCGACGCTGGCGATATCAAAATTGCTGTCTGCCAGGTGATCGCTGATGTACTGACA 1680 **araC**

AGCCTCGCGTACCCGATTATCCATCGGTGGATGGAGCGACTCGTTAATCGCTTCCATGCG 1740

CCGCAGTAACAATTGCTCAAGCAGATTTATCGCCAGCAGCTCCGAATAGCGCCCTTCCCC 1800

TTGCCCGGCGTTAATGATTTGCCCAAACAGGTCGCTGAAATGCGGCTGGTGCGCTTCATC 1860

CGGGCGAAAGAAACCCGTATTGGCAAATATTGACGGCCAGTTAAGCCATTCATGCCAGTA 1920

GGCGCGCGGACGAAAGTAAACCCACTGGTGATACCATTCGCGAGCCTCCGGATGACGACC 1980

GTAGTGATGAATCTCTCCTGGCGGGAACAGCAAAATATCACCCGGTCGGCAGACAAATTC 2040

TCGTCCCTGATTTTTCACCACCCCCTGACCGCGAATGGTGAGATTGAGAATATAACCTTT 2100

CATTCCCAGCGGTCGGTCGATAAAAAAATCGAGATAACCGTTGGCCTCAATCGGCGTTAA 2160

ACCCGCCACCAGATGGGCGTTAAACGAGTATCCCGGCAGCAGGGGATCATTTTGCGCTTC 2220

AGC**CAT**acttttcatactcccaccattcagagaagaaaccaattgtccatattgcatcag 2280

acattgccgtcactgcgtcttttactggctcttctcgctaacccaaccggtaaccccgct 2340

tattaaaagcattctgtaacaaagcgggaccaaagccatgacaaaaacgcgtaacaaaag 2400

tgtctataatcacggcagaaaagtccacattgattatttgcacggcgtcacactttgcta 2460

tgccatagcatttttatccataagattagcggatcctacctgacgctttttatcgcaact 2520

ctctactgtttctccatacccgtttttttggatggagtgaaacg**ATG**CAGTTTAAGGTTT 2580

ACACCTATAAAAGAGAGAGCCGTTATCGTCTGTTTGTGGATGTACAGAGTGATATTATTG 2640 **ccdB**

ACACGCCCGGGCGACGGATGGTGATCCCCCTGGCCAGTGCACGTCTGCTGTCAGATAAAG 2700 ccdBseqF1

TCTCCCGTGAACTTTACCCGGTGGTGCATATCGGGGATGAAAGCTGGCGCATGATGACCA 2760 ccdBseqF2

CCGATATGGCCAGTGTGCCGGTCTCCGTTATCGGGGAAGAAGTGGCTGATCTCAGCCACC 2820

GCGAAAATGACATCAAAAACGCCATTAACCTGATGTTCTGGGGAATA**TAA**gagctcccaa 2880 **ccdBR**

cgcgttggatgcatagcttgagtattctatagtgtcacctaaatagcttggcgtaatcat 2940

**Figure S1.** Arrangement of antibiotic resistance, *araC* and *ccdB* gene sequences in the *ccdB* cassettes.

(a) Schematic structure of *ccdB* gene cassettes. (b) to (d): Sequences of *CmR-araC-ccdB*, *KmR-araC-ccdB* and *SpR-araC-ccdB* cassettes. The position of forward (CmF, KmF and SpF in yellow) and reverse (ccdBR in blue) primers used for PCR amplification of cassettes is indicated. Gene specific primers CmseqR1/2, KmseqR1/2, SpseqR1/2 (blue) and ccdBseqF1/2 (yellow) are used for sequencing the junctions of cassette insertions. The I-SceI site (red) in the middle of cassettes provides an alternative mean for counter-selection in the recombineering *E. coli* host strain GS1783, which carries in addition to the heat-inducible *Red* recombinase genes a chromosomally integrated *araC-pBAD-I-SceI* gene that can be induced in parallel with the *araC-ccdB* lethal gene (Tischer et al., 2010).

**Tischer, B.K., Smith, G.A., and Osterrieder, N.** (2010). En passant mutagenesis: a two step markerless red recombination system. *Methods Mol. Biol.* **634**, 421-430.
