# Supplementary material for: Gene modification by fast‐track recombineering for cellular localization and isolation of components of plant protein complexes
Source: Plant J. 2019 Jul 26;100(2):411–29. doi: 10.1111/tpj.14450 (PMC6852550; doi:10.1111/tpj.14450)
Supplement: Supplementary file 2 — Figure S2. Example templates for planning the recombineering experiments and sequences of modified plant genes in pGAP and pGAPBR vectors constructed in this work. [file TPJ-100-411-s002.docx]

**(a)**

**EcoRI-BamHI cleaved pGAPKm and pGAPHyg vectors (NCBI Accession No: EU933992.1 and EU933993.1) used for gap-repair cloning of *CDKF;1*, *CDKD;1*, *CDKD;2* and *CDKD;3* gene constructs**

**pGAPKm**

**BamHI**

**GGATCC**gggttacccggccgccgtgctggaccgggttgaatggtgcccgtaactttcggt 60 pGAPKm/BamHI

agagcggacggccaatactcaacttcaaggaatctcacccatgcgcgccggcggggaacc 120

ggagttcccttcagtgaGcgttattagttcgccgctcggtgtgtcgtagatactagcccc 180

tggggcActtttgaaatttgaataagatttatgtaatcagtcttttaggtttgaccggtt 240

ctgccgctttttttaaattggatttgtaataataaaacgcaattgtttgttattgtggcg 300 OCSpA

ctctatcatagatgtcgctataaacctattcagcacaatatattgttttcattttaatat 360

tgtacatataagtagtagggtacaatcagtaaattgaacggagaatattattcataaaaa 420

tacgatagtaacgggtgatatattcattagaatgaaccgaaaccggcggtaaggatctga 480

gctacacatgctcaggttttttacaacgtgcacaacagaattgaaagcaaatatcatgcg 540

atcataggcgtctcgcatatctcattaaagcagggggtgggcgaagaactccagcatgag 600

atccccgcgctggaggatcatccagccggcgtcccggaaaacgattccgaagcccaacct 660

ttcatagaaggcggcggtggaatcgaaatctcgtgatggcaggttgggcgtcgcttggtc 720

ggtcatttcgaaccccagagtcccgc**TCA**GAAGAACTCGTCAAGAAAGCGATAGAAGGCG 780

ATGCGCTGCGAATCGGGAGCGGCGATACCGTAAAGCACGAGGAAGCGGTCAGCCCATTCG 840

CCGCCAAGCTCTTCAGCAATATCACGGGTAGCCAACGCTATGTCCTGATAGCGGTCCGCC 900

ACACCCAGCCGGCCACAGTCGATGAATCCAGAAAAGCGGCCATTTTCCACCATGATATTC 960

GGCAAGCAGGCATCGCCATGGGTCACGACGAGATCCTCGCCGTCGGGCATGCGCGCCTTG 1020

AGCCTGGCGAACAGTTCGGCTGGCGCGAGCCCCTGATGCTCTTCGTCCAGATCATCCTGA 1080

TCGACAAGACCGGCTTCCATCCGAGTACGTGCTCGCTCGATGCGATGTTTCGCTTGGTGG 1140 KmR/*aphII*

TCGAATGGGCAGGTAGCCGGATCAAGCGTATGCAGCCGCCGCATTGCATCAGCCATGATG 1200

GATACTTTCTCGGCAGGAGCAAGGTGAGATGACAGGAGATCCTGCCCCGGCACTTCGCCC 1260

AATAGCAGCCAGTCCCTTCCCGCTTCAGTGACAACGTCGAGCACAGCTGCGCAAGGAACG 1320

CCCGTCGTGGCCAGCCACGATAGCCGCGCTGCCTCGTCCTGCAGTTCATTCAGGGCACCG 1380

GACAGGTCGGTCTTGACAAAAAGAACCGGGCGCCCCTGCGCTGACAGCCGGAACACGGCG 1440

GCATCAGAGCAGCCGATTGTCTGTTGTGCCCAGTCATAGCCGAATAGCCTCTCCACCCAA 1500

GCGGCCGGAGAACCTGCGTGCAATCCATCTTGTTCAATCCA**CAT**gatcatgggccggatc 1560

tggattgagagtgaatatgagactctaattggataccgaggggaatttatggaacgtcag 1620

tggagcatttttgacaagaaatatttgctagctgatagtgaccttaggcgacttttgaac 1680 pNOS

gcgcaataatggtttctgacgtatgtgcttagctcattaaactccagaaacccgcggctc 1740

agtggctccttcaacgttgcggttctgtcagttccaaacgtaaaacggcttgtcccgcgt 1800

catcggcgggggtcataacgtgactcccttaattctccgctcatgatcagattgtcgttt 1860 **T-DNA**

cccgccttcggtttaaactatcagtgtt**TGACAGGATATATTGGCGGGTAAAC**ctaagag 1920 **RB**

aaaagagcgtttattagaataatcggatatttaaaagggcgtgaaaaggtttatccgttc 1980

gtccatttgtatgtgcatgccaaccacagggttcccctcgggagtgcttggcattccgtg 2040

cgataatgacttctgttcaaccacccaaacgtcggaaagcctgacgacggagcagcattc 2100

caaaaagatcccttggctcgtctgggtcggctagaaggtcgagtgggctgctgtggcttg 2160

atccctcaacgcggtcgcggacgtagcgcagcgccgaaaaatcctcgatcgcaaatccga 2220

cgctgtcgaaaagcgtgatctgcttgtcgctctttcggccgacgtcctggccagtcatca 2280 pTiC58

cgcgccaaagttccgtcacaggatgatctggcgcgagttgctggatctcgccttcaatcc 2340

gggtctgtggcgggaactccacgaaaatatccgaacgcagcaagatatcgcggtgcatct 2400

cggtcttgcctgggcagtcgccgccgacgccgttgatgtggacgccgggcccgatcatat 2460

tgtcgctcaggatcgtggcgttgtgcttgtcggccgttgctgtcgtaatgatatcggcac 2520

cttcgaccgcctgttccgcagaggtgcaggcctcgatctgaaacccgaaccgctggagat 2580

tgcgggagcagcgagcagtagcctcggggtcgatgtcgtaaagtcgtatccgatcgacgc 2640

cgatcagcgccttgaaggccaaagcctggaactcactttgggcaccgttgccgatcagcg 2700

ccatcgtgcgcgaatctttacgggccagatactttgccgcgatcgcggaggtcgcggccg 2760

ttcgcaaggccgtcaggattgtcatttccgacagcagcagcggatagccgctatcgacat 2820

cggagagcacgccgaacgcggttaccggcctcttcatcgggaatgcgcgcgaccttcagc 2880

atcgccggcatgtccccctggcggacgggaagtatccagctcgagatccggattttgtag 2940

ccctggccgacggccagcaggtaggccgacaggctcatgccggccgccgccgccttttcc 3000

tcaatcgctcttcgttcgtctggaaggcagtacaccttgataggtgggctgcccttcctg 3060

gttggcttggtttcatcagccatccgcttgccctcatctgttacgccggcggtagccggc 3120

cagcctcgcagagcaggattcccgttgagcaccgccaggtgcgaataagggacagtgaag 3180 **RK2 oriT**

aaggaacacccgctcgcgggtgggcctacttcacctatcctgcccggctgacgccgttgg 3240 nick site

atacaccaaggaaagtctacacgaaccctttggcaaaatcctgtatatcgtgcgaaaaag 3300

gatggatataccgaaaaaatcgctataatgaccccgaagcagggttatgcagcggaaaag 3360

cgctgcttccctgctgttttgtggaatatctaccgactggaaacaggcaaatgcaggaaa 3420

ttactgaactgaggggacaggcgagagacgatgccaaagagctacaccgacgagctggcc 3480

gagtgggttgaatcccgcgcggccaagaagcgccggcgtgatgaggctgcggttgcgttc 3540

ctggcggtgagggcggatgtcgaggcggcgttagcgtccggctatgcgctcgtcaccatt 3600

tgggagcacatgcgggaaacggggaaggtcaagttctcctacgagacgttccgctcgcac 3660

gccaggcggcacatcaaggccaagcccgccgatgtgcccgcaccgcaggccaaggctgcg 3720

gaacccgcgccggcacccaagacgccggagccacggcggccgaagcaggggggcaaggct 3780

gaaaagccggcccccgctgcggccccgaccggcttcaccttcaacccaacaccggacaaa 3840

aaggatctagcgtggactcaaggctctcgcgaatggctcgcgttggaaactttcattgac 3900

acttgaggggcaccgcagggaaattctcgtccttgcgagaaccggctatgtcgtgctgcg 3960

catcgagcctgcgcccttggcttgtctcgcccctctccgcgtcgctacggggcttccagc 4020

gcctttccgacgctcaccgggctggttgccctcgccgctgggctggcggccgtctatggc 4080

cctgcaaacgcgccagaaacgccgtcgaagccgtgtgcgagacaccgcggccgccggcgt 4140

tgtggatacctcgcggaaaacttggccctcactgacagatgaggggcggacgttgacact 4200

tgaggggccgactcacccggcgcggcgttgacagatgaggggcaggctcgatttcggccg 4260 **RK2 oriV**

gcgacgtggagctggccagcctcgcaaatcggcgaaaacgcctgattttacgcgagtttc 4320

ccacagatgatgtggacaagcctggggataagtgccctgcggtattgacacttgaggggc 4380

gcgactactgacagatgaggggcgcgatccttgacacttgaggggcagagtgctgacaga 4440

tgaggggcgcacctattgacatttgaggggctgtccacaggcagaaaatccagcatttgc 4500

aagggtttccgcccgtttttcggccaccgctaacctgtcttttaacctgcttttaaacca 4560

atatttataaaccttgtttttaaccagggctgcgccctgtgcgcgtgaccgcgcacgccg 4620

aaggggggtgcccccccttctcgaaccctcccggcccgctaacgcgggcctcccatcccc 4680

ccaggggctgcgcccctcggccgcgaacggcctcaccccaaaaatggcagcgctggcagt 4740

ccttgccattgccgggatcggggcagtaacgggatgggcgatcagcccgagcgcgacgcc 4800

cggaagcattgacgtgccgcaggtgctggcatcgacattcagcgaccaggtgccgggcag 4860 RK2

tgagggcggcggcctgggtggcggcctgcccttcacttcggccgtcggggcattcacgga 4920

cttcatggcggggccggcaatttttaccttgggcattcttggcatagtggtcgcgggtgc 4980

cgtgctcgtgttcgggggtgaattgcaagctagcttgcttggtcgttccggtaccgtgaa 5040

cgtcggctcgattgtacctgcgttcaaatactttgcgatcgtgttgcgcgcctgcccggt 5100 pTiAch5

gcgtcggctgatctcacggatcgactgcttctctcgcaacgccatccgacggatgatgtt 5160

taaaagtcccatgtggatcactccgttgccccgtcgctcaccgtgttggggggaaggtgc 5220

acatggctcagttctcaatggaaattatctgcctaaccggctcagttctgcgtagaaacc 5280

aacatgcaagctccaccgggtgcaaagcggcagcggc**GGCAGGATATATTCAATTGTAAA** 5340 **LB**

**T**ggcttcatgtccgggaaatctacatggatcagcaatgagtatgatggtcaatatggaga 5400 **T-DNA**

aaaagaaagagtaattaccaattttttttcaattcaaaaatgtagatgtccgcagcgtta 5460

ttataaaatgaaagtacattttgataaaacgacaaattacgatccgtcgtatttataggc 5520

gaaagcaataaacaaattattctaattcggaaatctttatttcgacgtgtctacattcac 5580

gtccaaatgggggcttagatgagaaacttcacgatcgatgggagaggcggtttgcgtatt 5640 pBSKII(-)

gggcgctcttccgcttcctcgctcactgactcgctgcgctcggtcgttcggctgcggcga 5700

gcggtatcagctcactcaaaggcggtaatacggttatccacagaatcaggggataacgca 5760

ggaaagaacatgtgagcaaaaggccagcaaaaggccaggaaccgtaaaaaggccgcgttg 5820

ctggcgtttttccataggctccgcccccctgacgagcatcacaaaaatcgacgctcaagt 5880 pBSK ori

cagaggtggcgaaacccgacaggactataaagataccaggcgtttccccctggaagctcc 5940

ctcgtgcgctctcctgttccgaccctgccgcttaccggatacctgtccgcctttctccct 6000

tcgggaagcgtggcgctttctcatagctcacgctgtaggtatctcagttcggtgtaggtc 6060

gttcgctccaagctgggctgtgtgcacgaaccccccgttcagcccgaccgctgcgcctta 6120

tccggtaactatcgtcttgagtccaacccggtaagacacgacttatcgccactggcagca 6180

gccactggtaacaggattagcagagcgaggtatgtaggcggtgctacagagttcttgaag 6240

tggtggcctaactacggctacactagaagaacagtatttggtatctgcgctctgctgaag 6300

ccagttaccttcggaaaaagagttggtagctcttgatccggcaaacaaaccaccgctggt 6360

agcggtggtttttttgtttgcaagcagcagattacgcgcagaaaaaaaggatctcaagaa 6420

gatcctttgatcttttctacggggtctgacgctcagtggaacgaaaactcacgttaaggg 6480

attttggtcatgagattatcaaaaaggatcttcacctagatccttttaaattaaaaatga 6540

agttttaaatcaatctaaagtatatatgagtaaacttggtctgacag**TTA**CCAATGCTTA 6600

ATCAGTGAGGCACCTATCTCAGCGATCTGTCTATTTCGTTCATCCATAGTTGCCTGACTC 6660

CCCGTCGTGTAGATAACTACGATACGGGAGGGCTTACCATCTGGCCCCAGTGCTGCAATG 6720

ATACCGCGAGACCCACGCTCACCGGCTCCAGATTTATCAGCAATAAACCAGCCAGCCGGA 6780

AGGGCCGAGCGCAGAAGTGGTCCTGCAACTTTATCCGCCTCCATCCAGTCTATTAATTGT 6840

TGCCGGGAAGCTAGAGTAAGTAGTTCGCCAGTTAATAGTTTGCGCAACGTTGTTTGCATT 6900

GCTACAGGCATCGTGGTGTCACGCTCGTCGTTTGGTATGGCTTCATTCAGCTCCGGTTCC 6960 AmpR/CbR

CAACGATCAAGGCGAGTTACATGATCCCCCATGTTGTGCAAAAAAGCGGTTAGCTCCTTC 7020

GGTCCTCCGATCGTTGTCAGAAGTAAGTTGGCCGCAGTGTTATCACTCATGGTTATGGCA 7080

GCACTGCATAATTCTCTTACTGTCATGCCATCCGTAAGATGCTTTTCTGTGACTGGTGAG 7140

TACTCAACCAAGTCATTCTGAGAATAGTGTATGCGGCGACCGAGTTGCTCTTGCCCGGCG 7200

TCAATACGGGATAATACCGCGCCACATAGCAGAACTTTAAAAGTGCTCATCATTGGAAAA 7260

CGTTCTTCGGGGCGAAAACTCTCAAGGATCTTACCGCTGTTGAGATCCAGTTCGATGTAA 7320

CCCACTCGTGCACCCAACTGATCTTCAGCATCTTTTACTTTCACCAGCGTTTCTGGGTGA 7380

GCAAAAACAGGAAGGCAAAATGCCGCAAAAAAGGGAATAAGGGCGACACGGAAATGTTGA 7440

ATACT**CAT**actcttcctttttcaatattattgaagcatttatcagggttattgtctcatg 7500

agcggatacatatttgaatgtatttagaaaaataaacaaataggggttccgcgcacattt 7560

ccccgaaaagtgccacctgacgcgccctgtagcggcgcattaagcgcggcgggtgtggtg 7620

gttacgcgcagcgtgaccgctacacttgccagcgccctagcgcccgctcctttcgctttc 7680

ttcccttcctttctcgccacgttcgccggctttccccgtcaagctctaaatcgggggctc 7740

cctttagggttccgatttagtgctttacggcacctcgaccccaaaaaacttgattagggt 7800

gatggttcacgtagtgggccatcgccctgatagacggtttttcgccc**GAATTC** 7853

**EcoRI**

**pGAPHyg**

**BamHI**

**GGATCC**tagaagctagcttcacgctgccgcaagcactcagggcgcaagggctgctaaagg 60

aagcggaacacgtagaaagccagtccgcagaaacggtgctgaccccggatgaatgtcagc 120

tactgggctatctggacaagggaaaacgcaagcgcaaagagaaagcaggtagcttgcagt 180 Tn5 seq

gggcttacatggcgatagctagactgggcggttttatggacagcaagcgaaccggaattg 240

ccagctggggcgccctctggtaaggttgggaagccctgcaaagtaaactggatggctttc 300

ttgccgccaaggatctgatggcgcaggggatcaagatcatgagcggagaattaagggagt 360

cacgttatgacccccgccgatgacgcgggacaagccgttttacgtttggaactgacagaa 420

ccgcaacgttgaaggagccactcagccgcgggtttctggagtttaatgagctaagcacat 480 pNOS

acgtcagaaaccattattgcgcgttcaaaagtcgcctaaggtcactatcagctagcaaat 540

atttcttgtcaaaaatgctccactgacgttccataaattcccctcggtatccaattagag 600

tctcatattcactctcaatccagatcggggggcaataagat**ATG**AAAAAGCCTGAACTCA 660

CCGCGACGTCTGTCGAGAAGTTTCTGATCGAAAAGTTCGACAGCGTCTCCGACCTGATGC 720

AGCTCTCGGAGGGCGAAGAATCTCGTGCTTTCAGCTTCGATGTAGGAGGGCGTGGATATG 780

TCCTGCGGGTAAATAGCTGCGCCGATGGTTTCTACAAAGATCGTTATGTTTATCGGCACT 840

TTGCATCGGCCGCGCTCCCGATTCCGGAAGTGCTTGACATTGGGGCATTCAGCGAGAGCC 900

TGACCTATTGCATCTCCCGCCGTGCACAGGGTGTCACGTTGCAAGACCTGCCTGAAACCG 960

AACTGCCCGCTGTTCTGCAGCCGGTCGCGGAGGCCATGGATGCGATCGCTGCGGCCGATC 1020

TTAGCCAGACGAGCGGGTTCGGCCCATTCGGACCGCAAGGAATCGGTCAATACACTACAT 1080 HygR/ *aph(IV)*

GGCGTGATTTCATATGCGCGATTGCTGATCCCCATGTGTATCACTGGCAAACTGTGATGG 1140

ACGACACCGTCAGTGCGTCCGTCGCGCAGGCTCTCGATGAGCTGATGCTTTGGGCCGAGG 1200

ACTGCCCCGAAGTCCGGCACCTCGTGCACGCGGATTTCGGCTCCAACAATGTCCTGACGG 1260

ACAATGGCCGCATAACAGCGGTCATTGACTGGAGCGAGGCGATGTTCGGGGATTCCCAAT 1320

ACGAGGTCGCCAACATCTTCTTCTGGAGGCCGTGGTTGGCTTGTATGGAGCAGCAGACGC 1380

GCTACTTCGAGCGGAGGCATCCGGAGCTTGCAGGATCGCCGCGGCTCCGGGCGTATATGC 1440

TCCGCATTGGTCTTGACCAACTCTATCAGAGCTTGGTTGACGGCAATTTCGATGATGCAG 1500

CTTGGGCGCAGGGTCGATGCGACGCAATCGTCCGATCCGGAGCCGGGACTGTCGGGCGTA 1560

CACAAATCGCCCGCAGAAGCGCGGCCGTCTGGACCGATGGCTGTGTAGAAGTACTCGCCG 1620

ATAGTGGAAACCGACGCCCCAGCACTCGTCCGAGGGCAAAGGAA**TAG**agtagatgccgac 1680

cgggatcttcgatccccgatcgttcaaacatttggcaataaagtttcttaagattgaatc 1740

ctgttgccggtcttgcgatgattatcatataatttctgttgaattacgttaagcatgtaa 1800 NOSpA

taattaacatgtaatgcatgacgttatttatgagatgggtttttatgattagagtcccgc 1860

aattatacatttaatacgcgatagaaaacaaaatatagcgcgcaaactaggataaattat 1920

cgcgcgcggtgtcatctatgttactagatcgggaattgccaagctgatcagattgtcgtt 1980 **T-DNA**

tcccgccttcggtttaaactatcagtgtt**TGACAGGATATATTGGCGGGTAAAC**ctaaga 2040 **RB**

GAaaagagcgtttattagaataaTcggatatttaaaagggcgtgaaaaggtttatccgtt 2100

cgtccatttgtatgtgcatgccaaccacagggttcccctcgggagtgcttggcattccgt 2160

gcgataatgacttctgttcaaccacccaaacgtcggaaagcctgacgacggagcagcatt 2220

ccaaaaagatcccttggctcgtctgggtcggctagaaggtcgagtgggctgctgtggctt 2280 pTiC58

gatccctcaacgcggtcgcggacgtagcgcagcgccgaaaaatcctcgatcgcaaatccg 2340

acgctgtcgaaaagcgtgatctgcttgtcgctctttcggccgacgtcctggccagtcatc 2400

acgcgccaaagttccgtcacaggatgatctggcgcgagttgctggatctcgccttcaatc 2460

cgggtctgtggcgggaactccacgaaaatatccgaacgcagcaagatatcgcggtgcatc 2520

tcggtcttgcctgggcagtcgccgccgacgccgttgatgtggacgccgggcccgatcata 2580

ttgtcgctcaggatcgtggcgttgtgcttgtcggccgttgctgtcgtaatgatatcggca 2640

ccttcgaccgcctgttccgcagaggtgcaggcctcgatctgaaacccgaaccgctggaga 2700

ttgcgggagcagcgagcagtagcctcggggtcgatgtcgtaaagtcgtatccgatcgacg 2760

ccgatcagcgccttgaaggccaaagcctggaactcactttgggcaccgttgccgatcagc 2820

gccatcgtgcgcgaatctttacgggccagatactttgccgcgatcgcggaggtcgcggcc 2880

gttcgcaaggccgtcaggattgtcatttccgacagcagcagcggatagccgctatcgaca 2940

tcggagagcactgatcagattgtcgtttcctactcgagatccggattttgtagccctggc 3000

cgacggccagcaggtaggccgacaggctcatgccggccgccgccgccttttcctcaatcg 3060

ctcttcgttcgtctggaaggcagtacaccttgataggtgggctgcccttcctggttggct 3120

tggtttcatcagccatccgcttgccctcatctgttacgccggcggtagccggccagcctc 3180

gcagagcaggattcccgttgagcaccgccaggtgcgaataagggacagtgaagaaggaac 3240 **RK2 oriT**

acccgctcgcgggtgggcctacttcacctatcctgcccggctgacgccgttggatacacc 3300 nick site

aaggaaagtctacacgaaccctttggcaaaatcctgtatatcgtgcgaaaaaggatggat 3360

ataccgaaaaaatcgctataatgaccccgaagcagggttatgcagcggaaaagcgctgct 3420

tccctgctgttttgtggaatatctaccgactggaaacaggcaaatgcaggaaattactga 3480

actgaggggacaggcgagagacgatgccaaagagctacaccgacgagctggccgagtggg 3540

ttgaatcccgcgcggccaagaagcgccggcgtgatgaggctgcggttgcgttcctggcgg 3600

tgagggcggatgtcgaggcggcgttagcgtccggctatgcgctcgtcaccatttgggagc 3660

acatgcgggaaacggggaaggtcaagttctcctacgagacgttccgctcgcacgccaggc 3720

ggcacatcaaggccaagcccgccgatgtgcccgcaccgcaggccaaggctgcggaacccg 3780

cgccggcacccaagacgccggagccacggcggccgaagcaggggggcaaggctgaaaagc 3840

cggcccccgctgcggccccgaccggcttcaccttcaacccaacaccggacaaaaaggatc 3900

tagcgtggactcaaggctctcgcgaatggctcgcgttggaaactttcattgacacttgag 3960

gggcaccgcagggaaattctcgtccttgcgagaaccggctatgtcgtgctgcgcatcgag 4020

cctgcgcccttggcttgtctcgcccctctccgcgtcgctacggggcttccagcgcctttc 4080

cgacgctcaccgggctggttgccctcgccgctgggctggcggccgtctatggccctgcaa 4140

acgcgccagaaacgccgtcgaagccgtgtgcgagacaccgcggccgccggcgttgtggat 4200

acctcgcggaaaacttggccctcactgacagatgaggggcggacgttgacacttgagggg 4260

ccgactcacccggcgcggcgttgatagatgaggggcaggctcgatttcggccggcgacgt 4320 **RK2 oriV**

ggagctggccagcctcgcaaatcggcgaaaacgcctgattttacgcgagtttcccacaga 4380

tgatgtggacaagcctggggataagtgccctgcggtattgacacttgaggggcgcgacta 4440

ctgacagatgaggggcgcgatccttgacacttgaggggcagagtgctgacagatgagggg 4500

cgcacctattgacatttgaggggctgtccacaggcagaaaatccagcatttgcaagggtt 4560

tccgcccgtttttcggccaccgctaacctgtcttttaacctgcttttaaaccaatattta 4620

taaaccttgtttttaaccagggctgcgccctgtgcgcgtgaccgcgcacgccgaaggggg 4680

gtgcccccccttctcgaaccctcccggcccgctaacgcgggcctcccatccccccagggg 4740

ctgcgcccctcggccgcgaacggcctcaccccaaaaatggcagcgctggcagtccttgcc 4800

attgccgggatcggggcagtaacgggatgggcgatcagcccgagcgcgacgcccggaagc 4860

attgacgtgccgcaggtgctggcatcgacattcagcgaccaggtgccgggcagtgagggc 4920 RK2

ggcggcctgggtggcggcctgcccttcacttcggccgtcggggcattcacggacttcatg 4980

gcggggccggcaatttttaccttgggcattcttggcatagtggtcgcgggtgccgtgctc 5040

gtgttcgggggtgaattgcaagctagcttgcttggtcgttccggtaccgtgaacgtcggc 5100 pTiAch5

tcgattgtacctgcgttcaaatactttgcgatcgtgttgcgcgcctgcccggtgcgtcgg 5160

ctgatctcacggatcgactgcttctctcgcaacgccatccgacggatgatgtttaaaagt 5220

cccatgtggatcactccgttgccccgtcgctcaccgtgttggggggaaggtgcacatggc 5280

tcagttctcaatggaaattatctgcctaaccggctcagttctgcgtagaaaccaacatgc 5340

aagctccaccgggtgcaaagcggcagcggc**GGCAGGATATATTCAATTGTAAAT**ggcttc 5400 **LB**

atgtccgggaaatctacatggatcagcaatgagtatgatggtcaatatggagaaaaagaa 5460 **T-DNA**

agagtaattaccaattttttttcaattcaaaaatgtagatgtccgcagcgttattataaa 5520

atgaaagtacattttgataaaacgacaaattacgatccgtcgtatttataggcgaaagca 5580

ataaacaaattattctaattcggaaatctttatttcgacgtgtctacattcacgtccaaa 5640

tgggggcttagatgagaaacttcacgatcgatgggagaggcggtttgcgtattgggcgct 5700 pBSKII(-)

cttccgcttcctcgctcactgactcgctgcgctcggtcgttcggctgcggcgagcggtat 5760

cagctcactcaaaggcggtaatacggttatccacagaatcaggggataacgcaggaaaga 5820

acatgtgagcaaaaggccagcaaaaggccaggaaccgtaaaaaggccgcgttgctggcgt 5880 pBSK ori

ttttccataggctccgcccccctgacgagcatcacaaaaatcgacgctcaagtcagaggt 5940

ggcgaaacccgacaggactataaagataccaggcgtttccccctggaagctccctcgtgc 6000

gctctcctgttccgaccctgccgcttaccggatacctgtccgcctttctcccttcgggaa 6060

gcgtggcgctttctcatagctcacgctgtaggtatctcagttcggtgtaggtcgttcgct 6120

ccaagctgggctgtgtgcacgaaccccccgttcagcccgaccgctgcgccttatccggta 6180

actatcgtcttgagtccaacccggtaagacacgacttatcgccactggcagcagccactg 6240

gtaacaggattagcagagcgaggtatgtaggcggtgctacagagttcttgaagtggtggc 6300

ctaactacggctacactagaagAacagtatttggtatctgcgctctgctgaagccagtta 6360

ccttcggaaaaagagttggtagctcttgatccggcaaacaaaccaccgctggtagcggtg 6420

gtttttttgtttgcaagcagcagattacgcgcagaaaaaaaggatctcaagaagatcctt 6480

tgatcttttctacggggtctgacgctcagtggaacgaaaactcacgttaagggattttgg 6540

tcatgagattatcaaaaaggatcttcacctagatccttttaaattaaaaatgaagtttta 6600

aatcaatctaaagtatatatgagtaaacttggtctgacag**TTA**CCAATGCTTAATCAGTG 6660

AGGCACCTATCTCAGCGATCTGTCTATTTCGTTCATCCATAGTTGCCTGACTCCCCGTCG 6720

TGTAGATAACTACGATACGGGAGGGCTTACCATCTGGCCCCAGTGCTGCAATGATACCGC 6780

GAGACCCACGCTCACCGGCTCCAGATTTATCAGCAATAAACCAGCCAGCCGGAAGGGCCG 6840

AGCGCAGAAGTGGTCCTGCAACTTTATCCGCCTCCATCCAGTCTATTAATTGTTGCCGGG 6900

AAGCTAGAGTAAGTAGTTCGCCAGTTAATAGTTTGCGCAACGTTGTTTGCATTGCTACAG 6960

GCATCGTGGTGTCACGCTCGTCGTTTGGTATGGCTTCATTCAGCTCCGGTTCCCAACGAT 7020

CAAGGCGAGTTACATGATCCCCCATGTTGTGCAAAAAAGCGGTTAGCTCCTTCGGTCCTC 7080 AmpR/CbR

CGATCGTTGTCAGAAGTAAGTTGGCCGCAGTGTTATCACTCATGGTTATGGCAGCACTGC 7140

ATAATTCTCTTACTGTCATGCCATCCGTAAGATGCTTTTCTGTGACTGGTGAGTACTCAA 7200

CCAAGTCATTCTGAGAATAGTGTATGCGGCGACCGAGTTGCTCTTGCCCGGCGTCAATAC 7260

GGGATAATACCGCGCCACATAGCAGAACTTTAAAAGTGCTCATCATTGGAAAACGTTCTT 7320

CGGGGCGAAAACTCTCAAGGATCTTACCGCTGTTGAGATCCAGTTCGATGTAACCCACTC 7380

GTGCACCCAACTGATCTTCAGCATCTTTTACTTTCACCAGCGTTTCTGGGTGAGCAAAAA 7440

CAGGAAGGCAAAATGCCGCAAAAAAGGGAATAAGGGCGACACGGAAATGTTGAATACT**CA** 7500

**T**actcttcctttttcaatattattgaagcatttatcagggttattgtctcatgagcggat 7560

acatatttgaatgtatttagaaaaataaacaaataggggttccgcgcacatttccccgaa 7620

aagtgccacctgacgcgccctgtagcggcgcattaagcgcggcgggtgtggtggttacgc 7680

gcagcgtgaccgctacacttgccagcgccctagcgcccgctcctttcgctttcttccctt 7740

cctttctcgccacgttcgccggctttccccgtcaagctctaaatcgggggctccctttag 7800

ggttccgatttagtgctttacggcacctcgaccccaaaaaacttgattagggtgatggtt 7860

cacgtagtgggccatcgccctgatagacggtttttcgcccGAATTC 7906

**EcoRI**

**(b)**

***CDKF;1*:*GFPPIPL* insertion in the EcoRI-BamHI sites of pGAPKm and pGAPHyg vectors, and the position of *cdkf;1* (GABI_315A10) T-DNA insertion mutation**

**EcoRI**

**GAATTC***ccgtgacccagcacctccaactattgagtctctaagtgagtttttgttgaactt* *60* F1FLANK1F_EcoRI

*ttgtgtagtgatgctttcacaatagttcttagatgtttggttacatatttttgtaggtga 120*

*tggcagcgatgagtccgatgaagaggataagaagaagaagaagaagaagcagatatcgaa 180*

*gaagaagagaaagcatgttggtcctactctggaatctggggaaaaacgtggaaggaaacc 240*

*ttccaagtataagggggaagagtttacaactgaggggaagcaaacgagaaagagacagaa 300*

*gaattaggactttgaaaattctttcttgcttatgtgtgtgtgtggactcttaggtgtctg 360*

*tcaaaataggactttctttttgtgtatttgggaatgttatgcgtagtttatgtgtagagg 420*

*acaaacttattttctgaaatggtttatgtgtgttaggattttattaaaatcaaaacgttt 480* ***FLANK1***

*ggctaagttgtcccggttggccggtcaataatacaagagtagctaagttgtcccagttgg 540*

*ccggtcaatacacataacttaaaatacaagagtagctaagttgtcccagttggccggtca 600*

*atacacaacttaaaatacaagagtagctaagttgacccagttagccgggacacccagaga 660*

*cagagtagctcgtcagctcttcaagctccttagctcgtcctcaagctcctcagttcgtcc 720*

*tcaaagctcctcagctcgtcttcaagctcttcccatcacagcgaccatctcctcgcctag 780*

*ctgtcagctagtcagctcgtccgactcctcgtctagctatcagctcatccctcaactaca 840*

*aaactcatctacctcctcgcctagcggcagttcgtccattgactgcatagtccacctatc* 900 F1FLANK13_SalI

*tcgtcgcccagctc*gtctataccttcagcagacaactcggtttgtccaattcgcctctct 960

accacggttcatccagctcgtccgagctcacttattctaatatgaa**CTA**CGTGTGGCTTG 1020

ATCCCATTTTTCGTACTTGTGTGATTCGTGCATCAACATTTTCGTACACATAACTTAAAA 1080

TACAAGAGTAGCTAAGTTGTCCCACTTGGCCGGTCAATACACAACTTAAAATACAAGAGT 1140

AACATATTTAGGTTCAAATGTCATACACCATCCTCAATAACATAAATCACATCATAATCA 1200

CCATGTAGAAAGTAATGCCCTAAGCCTATCAATCTCGACTTCTTTGGCGAGTAACCTTTC 1260

TTTAAGGGTTTCAATTTCGCCTTGCATGAGATGAATAGTTTCCCGGATGGACTCCAAAGC 1320

ATGATTGCATGCTAGTTGTCCATGTCTTAGTGCGGAATCAAGTTTGTGATTTACCTTCTC 1380

AAGCTCCTCAGTTACCGCCTGATCCCACCACTGAATAAGGTGGTTTCGGGACTCGTACTC 1440

TTCAGCCTGCGAAAGAGAGAAGACTTCATAAATATAATTCACTTGTACTTTGTTGTTCCA 1500

ATGAAATGAAAACACAATAACTTACTTTAAGCATTGGGCATTTGTAGACTCTGTCGCCTT 1560

GCCTGTAACCAGTTTTGCACACTTCAAGTTTTACAGGCTTACCACAAAAACATTTTTCTG 1620

GAACCCCCCAATTTGCATCGGCATTCGCCTCTCCACTATTCCAACTAGCTTCGGACGTGG 1680

ATGGTTGAGAGTAACTATAGGGCATCTACAGAACAGAGAAAGAAAGAGATATGAATCATG 1740

AACGCTATAAACCCTAGATCTACTAATTACGGCGGCTAGAAACCCGTAAGAAAGCCTAGA 1800

TCTACTAATTACGGCGGCTAGAAACCCGTAAGAAACCCTAGATCTACTAATTACGGCGGC 1860

TAGAAACCCTTTAGAAGAAGAGAGAAGAACGAGAGAGAGGAAGAACTCCAGTGCATGTAA 1920

TACCTTGGTTGTCGACAGCTAGGCGAGGAGATGGTCGCTGTGATGGGAAGAGCTTGAAGA 1980

CGAGCTGAGGAGCTTTGAGGACGATCTGAGGAGAGAAGTGAAGAAGAGAGAAGAACGAGA 2040

GAAGCGAAATAGAAGAGAGAGTGAGAATAAATAGGTAGAAACTTTGTAGTGTGAACGCAA 2100

TAATCGTAGTGTGAACGCGACTTCGTGGGGTCTATTTTTAAAAAAATTTATTTATTAGAA 2160

TATTCAGAATACACATAGTTGTATGTATATGTACAGTTGGCCGGAATCACAAACAGTTGA 2220

ACTATTCTAAATTTTGTGTTTTTGAAGTTGAATCTGAAAAACCAACTCATTTTTTTAGTT 2280

GGTCCCATAAGTTTTAACATATTAATGGATTTTAATAAGTTTTGATAGAATCATATTTAG 2340

TAAATATCTGTTTTCATACATAACGTATGTACATTATACAATCATGAACTATATGTTCCT 2400

TGTCATACATAGGTGATTTATTAGGAAACCAGTTTATGATCAAGTTTATTAATAACAATG 2460

TTGTAATATCATATTTTGGATTTATTCAATGTTTAGATGTTGATAATAGCTGAAAATATT 2520

GAGAATTATAATGCAAATAATATAGTACATCAGACTTACAAATAATATAATGCAAATACA 2580

AATGAACTTAGTTGTATTGGTTGAACCCTTTCTGCAGTTGGCCAGGTTGTTCCGGTTGAA 2640

CCCTTTCTGCATCAGACTTAATCTTCCTCAGGCTGCAGCCAAGCCTCCAAGTTTATTCCA 2700

GGTTGCGATTTTCTCCTCTTTCGCGCTTCACCAGCTGATGGAAACCGCTTCTCTTGATTT 2760

CGTCCTGGTCCTTTTGGTGTGTATTCCGGCGGAAAGGCAACTAACTCTTTAATATGGGCA 2820

GGAAGAACCCAAACCGACTCATGTGGGACTACATACAACGTCCGGTAATAGGCCAATGAC 2880

CAACTATCTATCAAGTAATACACAGAGCAGAGTCCGTAGATGGTTGTATCCGCATTTCTC 2940

CCTTCCTTGCGGGCGAGGTGCCTTGCTGCGGCCATAGCATGGACACACGGGATCTTGTCT 3000

ATATCAAAACATTTGCATGAACAACTTTTCATTTTCAAATCCACCAGAAAACTTACCGAG 3060

TTGAGATCAATCACGTTGTATTCTTGGTTGTAACTGTTAAGCTCAAACACAGTTAATTTG 3120

GCTGCAACTTGACATCTAATGTGCAATATGTTCTCCACAGTAGGCACAACTTGTGCTGTG 3180

ATTGGACAAGAACCTGAAGCTTGCCTATGCTCGTTGAACCATTCAGAAAACTTAGAAATC 3240

ATCACATCAATCATTGGTAACAAGTGGTATTTTCTCGCTTTTTTAAACACACCATTAATG 3300

GACTCCGCAGAATTACTAGTGTCAATGTTATACTTGTCTCCTTTAAAATGGCACCTCGAC 3360

CATTTCTCAAACCCTACTTTCACAAGAAAGTCAGCAGCTTTAGGCCATAAACTTGTAAAA 3420

TGACCAAACTCTTTCTCGAACTCACTCTCAGTATATGCATGAGCACAATCCCTGAACTTG 3480

ACAGCAGCTCCATCCTTGTCAATTGTCACACGATCTCTCATGTTTTGACACAAATGCCAT 3540

ATACAAGCTGCATGAAGCGCATTCGGGTACACCGTCTTGACAGCCTTCTTTATGCTTTGA 3600

TGCCTATCACTAATAAAGACCAATCCGGGAACATCCGAATATACAGTTTTCAGCTTTTCT 3660

AAGAACCAAATCCAACTTACATCCTTCTCGCTATCAATGATTCCAAACGCAAGTGGATAA 3720

TGATGATGATTAGGATCTTGTGCTGTTGCAATAACCAACATCCCACCATACACAGTTTTA 3780

AGATGAGTTGCATCCACAACTATCACTTTCCGCATTGCCCTAAAACCTTCAATGCAAGCG 3840

CCTAATGCAATGAAGAGGTACTTGAACTTTTTCTCTCCTTCCAACTCCACATAAGTTACT 3900

GTCCCTGGATTTACCTTCTCTAACATGTAAAGATAAGAAAATAACATCGTGTAGCTTCTT 3960

TCCGGAGTCCCTCGCACATCACTAACATGTAGCATTTTGCCTCTTAAGGCAGTGGAGTAT 4020

GAACAATGCACCCCAAGTCTTCCTCTTACAATCGACATAATGTTTTTTGGAGTTGGGGTC 4080

TCTAAATTTCCGGGATAATCACAATGTAAAACCGATGCTATTAATCTTGGTGTGCCTCGC 4140

TGTATACTGTTGCTTGTAGTCTCAACACTCCGAGAGCATGTATGCATCTTTCTATGCACT 4200

CTAACACTCCAAAAATGAGATTTTTTAGTTCTCGCAACTCTTAGATACCAAGTACACCCT 4260

TTAGAAGCTTGGCGGCATCGTAGCATAAGTCTCAACGGGTCTGACTTAATAGTATAGACT 4320

CCAAAAACTTCTTTCTTTGCAGCTCTGTTAACCAATTCCCACACAGCCTCCCTTGAACAA 4380

AATTCTTGACCAATTTCAATACCCGTACCATCATCCCATTCTATAACCTGCGGAGTAACT 4440

TCTACACTTCGTGGAATATCGTGAATATCATCATAATCAATCCTATCATCCTCATCTTGT 4500

TCCTCAACAGAATTCACCGGCCTTTCCATCGGCCTTTCGATAGCCACGATCTCCGTACCT 4560

ACTACATTCTCACAATCATTCACATCATTATCATATCCATCATGCACCATCTCACATACT 4620

TCATTTAAAACATCATCCTCTATTACATTTACACCAACTGAACTTCGACGCTCCGGTACA 4680

GAAATTTCCTCTCTCCTCTCAACTCCTATACCATTTAATTCCTCCACATGCAAAACAGGA 4740

ATCCGACTCTCTTCATCAGATTCTGTTAAAAATATGAAGACATCCTCATCATTTACAATA 4800

TATGTCTCTCTCCTAACTTTAGACAGATTGTAACTCAGTTTCAACTTGACAGAAGCTTCA 4860

TCAATCGCAACCTTCTTCATGATCTTGTCAACTAACATCGAATAGCTAATCTTCTCCAAA 4920

CTACTCGTCTTAAACGATATAACATATAGTGAATTTTTACCAATCCACTCATTCACTTCA 4980

GAATAGTATCCTCCAAAGTCAAAATGTACCTTAGTGTGGATCTTCTTCTT**CAT**cgctcct 5040 AT4G28970

gaaaatacaaccagttcaactccaatcaaacattttaatagttataccacatgtactctt 5100

atcatacgttgaaatcaatctctcgtcgcactcgtctctactgaacctaattcgttacct 5160

ttcaattgatttccaatgttaatacatacctattcgttaaactaattggttaatctaaac 5220

tttgcaaacacaaaccccccaaatctttaatatgaaatctcggaattcaaattcaactca 5280

cattcaacctacattcctttgctcatcacctcaaataacgtttcggcaacctctaatcga 5340

ttactctttactttgcaaaccaatcatcaaactatcaaattgaatcgagctgggaattat 5400

atagaattcaaaatcccaatctaaaatcacaatgcaccaatgtcaaaggaaatatgagaa 5460

gaaactgacctaattcgtggcttttacgccggatttcaagtcggcggcatgaggaaatcg 5520

atggtggccgacgaagaagatgaacgtcgaccaaagaagaagaacacgacgacgaagagg 5580

agaatctccgaactcgattttctttatttagcttttgaatttctttttcatttttttaat 5640

caactcattaagggtacaactggtatatcacattttcggtggcattcagattaacttatg 5700

gcagtccagatcagttttttcaaaaaagtggcataagtgataaaagtccccccctgcggt 5760

ttggtttcttgtccgctccaaaacttgtgagggcattttagtcattacaacatgggaact 5820

ggatttttaaactggtgaattcgttagcgtttatgtgaataatcatccatcat**caggaaa** 5880 CDKF;1F

**gATCCGAAGAAAAGCGAC**GCCGTTTTAAAGGAG**ATG**GATAAACAACCGGCGACCAGTTGG 5940 **CDKF;1**

AGTATCCATACCCGGCCGGAGATAATCGCCAAGTACGAGATCTTCGAGCGAGTCGGATCC 6000 At4g28980

GGAGCTTACGCCGACGTTTACAGAGCTCGCCGTCTCTCCGACGGTCTAATCGTCGCGCTC 6060

AAGGAGATTTTCGATTACCAATCTGCGTTCCGGGAGATTGACGCGCTCACCATCCTCAAT 6120

GGATCTCCTAATGTAGTTGTTATGCACGAATACTTCTGGCGTGAGGAAGAGAACGCTGTG 6180

CTTGTGCTTGAGTTTCTTAGGTCGGATCTCGCTGCGGTGATCCGAGATGGTAAGAGGAAG 6240

AAGAAGGTAGAAGGAGGGGATGGATTCTCGGTTGGGGAGATTAAGAGATGGATGATTCAG 6300

ATTTTAACCGGTGTTGATGCTTGTCATCGGAATTTGATTGTACATAGAGATTTGAAGCCT 6360

GGGAATATGTTGATTTCTGATGATGGAGTTCTTAAGCTTGCTGATTTTGGTCAGgtgaga 6420

ttatgattgttactgattgtgttagttatttcattgtttgatgcacaaatatgagaaacc 6480

tctgaatgaaaaatgcttggtttgcttgaaatttggaagagttagtgtaatcaagtggct 6540

agaccttatcatttagtgtaatctatgagtttctaaatgtgtgttggcttcttggttcat 6600

atacagGCTAGGATACTCATGGAGCATGATATTGTAGCTTCAGATGAAAACCAACAAGCT 6660

TACAAACTGGAAGACAAAGATGGAGAAACCAGCGAACCACCAGAAGTTATTCCTGATTAT 6720

GAGAACTCACCTAGGCAAGGGTCTGATGGTCAGGAACGGGAAGCGATGAGTAAAGACGAG 6780

TACTTCCGACAGGTGGAAGAGCTCAAGGCTAAACAGGTTGTTAGAGATGATACTGATAAG 6840

GACTCGAATGTGCATGATGGAGACATATCTTGCCTTGCAACGTGCACTGTTAGTGAAATG 6900

GATGATGATCTCGGTAGGAACTCGTTTTCTTACGATGCTGACGAAGCAGTGGATGATACA 6960

CAGGGTTTGATGACATCTTGTGTGGGAACCCGATGGTTTAGGCCACCAGAACTGCTCTAT 7020

GGATCTACGATGTATGGGTTAGAGGTCGATCTATGGTCGCTTGGTTGTGTGTTTGCAGAA 7080

CTCTTGTCTCTTGAGCCTTTGTTCCCAGGAATTTCAGATATTGATCAGATCAGCAGAGTG 7140

ACCAATGTGCTGGGAAATTTAAATGAAGAGGTCTGGCCTGGTTGTGTTGATCTTCCAGAC 7200

TACAAGTCAATCTCATTTGCTAAAGTAGAATCTCCTCTTG**G**TATTGAAGG**A**TGCCTTCCC 7260

/LB ----RB\ **GABI_315A10**

AACCATTCAGGGGATGTGATATCGCTGCTCAAGAAGCTTATCTGTTATGATCCAGCTAGT 7320

AGAGCTACTACTATGGAGATGCTGAATGACAAATATTTAAGTGAAGAGCCTCTTCCAGTT 7380

CCAGTTTCAGAGCTCTATGTGCCTCCGACAATGAGTGGACCAGATGAGGATTCTCCGAGA 7440 **F1seq5**

AAGTGGAATGATTACAGAGAAATGGATTCAGATTCAGATTTTGATGGCTTTGGACCCATG 7500 **F1_50ntF**

AATGTAAAGCCTACCAGTAGTGGATTTACAATAGAATTCCCC**ATG**GTGAGCAAGGGCGAG 7560

GAGCTGTTCACCGGGGTGGTGCCCATCCTGGTCGAGCTGGACGGCGACGTAAACGGCCAC 7620

AAGTTCAGCGTGTCCGGCGAGGGCGAGGGCGATGCCACCTACGGCAAGCTGACCCTGAAG 7680

TTCATCTGCACCACCGGCAAGCTGCCCGTGCCCTGGCCCACCCTCGTGACCACCCTGACC 7740

TACGGCGTGCAGTGCTTCAGCCGCTACCCCGACCACATGAAGCAGCACGACTTCTTCAAG 7800 GFP

TCCGCCATGCCCGAAGGCTACGTCCAGGAGCGCACCATCTTCTTCAAGGACGACGGCAAC 7860

TACAAGACCCGCGCCGAGGTGAAGTTCGAGGGCGACACCCTGGTGAACCGCATCGAGCTG 7920

AAGGGCATCGACTTCAAGGAGGACGGCAACATCCTGGGGCACAAGCTGGAGTACAACTAC 7980

AACAGCCACAACGTCTATATCATGGCCGACAAGCAGAAGAACGGCATCAAGGTGAACTTC 8040

AAGATCCGCCACAACATCGAGGACGGCAGCGTGCAGCTCGCCGACCACTACCAGCAGAAC 8100

ACCCCCATCGGCGACGGCCCCGTGCTGCTGCCCGACAACCACTACCTGAGCACCCAGTCC 8160

GCCCTGAGCAAAGACCCCAACGAGAAGCGCGATCACATGGTCCTGCTGGAGTTCGTGACC 8220 **PIPL:**

GCCGCCGGGATCACTCTCGGCATGGACGAGCTGTACAAGGTCGAC**ATG**GGTCATGATGAT 8280 CobW

CATCACCATGGTCATGACTGCCATGATCACCACAATGAGCATGAGCATGAGCATGAACAC 8340

GAGCATCACCATTCTCATGATCACACCCATGACTGGTCTCATCCTCAGTTCGAAAAAGGA 8400 StepII

GGTGGATCTGGTGGAGGTTCTGGAGGTGGATGGTCTCACCCACAATTTGAGAAGGGATCT 8460 HA

TATCCATACGATGTTCCAGATTATGCT**TGA**CCTTGGCATTTAAggtacgcagtcaaaagc 8520 **F1_50ntR**

tctggctcgcttaaacacataaatcagtctcttctctctctctctttcttcttcaaagag 8580

aatgggtatataccaaaacctgacatgtgtttctttaggtcttgattgatgcaacactct 8640

ttgttgtttgaagatcttgttacagaataccgcttagaaaattgttaacttttcgttagg 8700

acattgtttaggtatcaatggcggcaaaaacttttgaaattcgtttgctcattgatctca 8760

caacttttttttgcgctttgtttctctcatcgatctcagttttatgtgtttatctctcct 8820

tgg**cagAGTCCATGGTTGAGCATCTTGAT**CATCCCGACTTCCCCTGAATTCGCCTACTTT 8880 CDKF;1R/**F1seq3**

GTCAAAAATGTGTTGTGTGTGTGTGTGTGTTGTTCCTCTCTCTCTGCCTCCCTCGTATGA 8940

AAATGGTAACATTTACAATTGGAAAGTCGGTGTTAGCCTTTAGCTATTAGCTGAAGAGCT 9000

AGTTTTCTTGTTGCCCAGGCCATTCTTTACCCTATTTTTCATCTGAAGTAACAATGAAAC 9060

AAAATCTATATATACTCGAGTTTTGCTTtctaggatctgtatttcttaattgtttctata 9120

ttttgatcaattttgaacttttcatatcaagttatcaacatgtgtagtagcaacccttca 9180

ataatgagaccatggaataaaagcctatcctatatgtcatccagtttttgctaagataaa 9240

ttattttcagttacccaaattacataatcaaaactgatatcataaacaacaaaaatagca 9300

cacacaaaaatcataactgaatctgattcttccagaaatgtgaactgttgtttactataa 9360

aaagaagaaaaccagaaccacaatacatttgactccatgaatcagtcatactagtccaac 9420

atactaaatataaaacttaaggggcaatattgaaaattagacaaaagttgatagaccaaa 9480

agataaattaataacagttaccagtatctatcgccaagtctcccctagggttttttttCT 9540

CTGAAACGGACATCGAGAAATTCCAGGAGTGAGTTTTGACAATCGGAGAGAA**ATG**GGGTC 9600 AT4G28990

GACAGAAAAAGAGCAGACGACGCAACATCACCCGCCTCACATCAGCAGTCTCGTCGTACG 9660

TCCCTCCGGAAGCAACGACAGAGAAGACGGCCGCAACGCCGCCGGAGACTATGAGCCCGG 9720

AGAAGTCTCTCGTGACCGGCCTTCGTTTAATCGCTCGGATCGGTATAAGGGTGACAATGG 9780

TGgttcgtatctcttctaaacattccagtcctcttttctcctgagtttctcttctcgtta 9840

ttggtttcgatttctgaaattgggggaatgaagaacagaactaagtaggcatcacttgtt 9900

tctttcactacaaatgcttgtgattgtctacgtcttagtcaatgttttatctcctttgac 9960

ttgtgattccgatgaggggaattgtgtgtagttatctcgtgagcatttggtgctatctta 10020

aaggtttttgataatgctctcataactataacaatgctgtgtaaattgcaaatgagcatt 10080

gttcttgccttgaagtacttaggtcttgatgtttacataatgcattgttttcgtaaaaga 10140

tgcattatgtaatgtatctaaagagtatagtgactcttagtgcgattatacattgctaca 10200

gatgcatatcaacattccctgttcagttcttagtgaattgtttggtatatgctctgagaa 10260

tgctgacaccaacggacttgtaatttcacagtgtcagtgctccattttattggtctgcaa 10320

agaaaatgattttttgaactttgctgtctgctgatgtatatttgtattatactgtagttg 10380

acttagtcttgttctactggctactttttgtgtgtgtgttagGACATAGAACTCGGGCAA 10440

GTTCTAGCTCACCAGGCCGTCGTGGATATGAAGATCACAAGCATGGCTCTGATCTTAATC 10500

ATTCAGGCGTTCCACCTCGTGGCCGTGAGTTAAGCAGCAGAAGGGAAGCACCTGGGAGAC 10560

ACAGAGACTATTCCCCACCCTTAGCTAGAGGTGGAGCTGGTGCTCGTCCTTATCGAAGAG 10620

GTTTAGATGGGCCTGAACCTCCACATGGAAGGGATGGCATGAGCAGAAATAATATATCAA 10680

AGGTGCAACCAAGAGAAGGAGATTGGTACTGTCTTGATCCATtgtaagtatccctgctat 10740

tctaaatttctacaatctcaggttattctatatgaactctatagaatattggtatctcag 10800

aacctagagaagtttctaaatcccatattatgtttgttttgtcatgaatacagATGCAGA 10860

AACTTGAACTTTGCGAGACGCGAGTCCTGCTACAAATGCAAGAGGCATCGTTATGCACCG 10920

GCTAATAGTCCTCCACTGCCTCGCCTTCTTCCTCCTCCCATGAATCATTCCCCAAGAAGA 10980

GACTTTAATGGCTACAGATCTCCTCCGCGTGGCTGGCCCAGAGACTACCCGCCGCCAAGG 11040

CATGACCATCCCACATGGAGAGACAGAGAACGAGACCGTCCGCATTACTCAGACCATGAG 11100

TACCCTCCTAGCAGAAGGATAGCATCTGACTGGGCCCATACAGAGCCGCTCCCAAAACCT 11160

CATTATGACAGACGACCACCTCTCAGTCCACCTCGTGGTGGCAGATGGGGACGAGTTTTG 11220

AGAGAGAGGAGCAGATCCCCGCCATTGAGAGATGTTCCACCACCGCCATTGAGAGATGGT 11280

CCACCACCGTCATTGAGAGGAGGCGGTCCACCGCTGCACAGAGATTACCGCCGTGACAGT 11340

CACTT*TGACAGAGAAAGACGAGACGATGGGCGTGGCGGCAGAGGCAGAATGGGAAACTCT* 11400 F1FLANK2F_SalI

*TAC****TGA****TGCAAAAACTGTGTATGTCTTGGGATTTTGGTTTAGACAGTTCAAATTTTCGCG 11460*

*GAATGTGACCTTGTATTACACCTTAGATTCTTTGTTCTTGTTAGCAAAACTCTTGTGCCA 11520*

*TTAGTGCAAAAACCAAACAAATGAGAAGCAATTTTGGTGCTCtttcgtaattaaagaaac 11580*

*atttagggccgaaacaaaaaatatacttcttcttaatattccccttccgtttcggcttcc 11640*

*gcttgagaaggtgcgtactatgagttcacgagtggtctatctacgtgtcagttttttatt 11700*

*gggtgatagctaaatacacgcatattaaaagggctttttggatatttgcttcaccgtaga 11760*

*tcggacggtggaaagagacaaaacctaaagcatttatgaattgacatcgttggatcatcg 11820*

*tatttaacaagaaccgtacgatgagattgccacgcaaatctaaccgtcgaatataatcca 11880* ***FLANK2***

*gaaaattgaaatttcaaaacatgttagagagagggaggaggagagatggtgtgagagaag 11940*

*aagaagaacaaaaccgaaagtatttttttgtgcttttaatttttgtttttaattttacca 12000*

*aattcctctgagggcttcttcttcttcttctccgtcttcttcttcttcttcttcttcgtt 12060*

*tagagctaaaacccaatagaaccctaattctcatctcccttaaagcttatcccttttctc 12120*

*tctctatatatatacgcggatcaaattcaaagtctcagtttttgagctttcttaacagcc 12180*

*gccgaatcgatttgtgattttcccgctccggagagattcggatctggatttttcgagctg 12240*

*atttgtaggaatttctcgttttctcgcggtgaattatatcaactgtggaaaaaaggaaaa 12300*

*tttctgggtttttttggttttggagattggaaaatctttgagagatgggtgaagacggcg 12360*

*gaggaggcgaattcccgccaaaaaaggacggtgttgaagagggttttccgacgaagaagc 12420* F1FLANK1_BamHI

*c***GGATCC** 12427

**BamHI**

***CDKF;1*:*CmR-araC-ccdB* cassette insertion between the gene specific primers F1seq5 and F1seq3:**

GTTTCAGAGCTCTATGTGCCTCCGACAATGAGTGGACCAGATGAGGATTCTCCGAGAAAG 60 F1seq5

TGGAATGATTACAGAGAAATGGATTCAGATTCAGATTTTGATGGCTTTGGACCCATGAAT 120 F1_50ntF

GTAAAGCCTACCAGTAGTGGATTTACAATAGAATTCCCCCtgtgacggaagatcacttcg 180 CmR5F

cagaataaataaatcctggtgtccctgttgataccgggaagccctgggccaacttttggc 240

gaaaatgagacgttgatcggcacgtaagaggttccaactttcaccataatgaaataagat 300

cactaccgggcgtattttttgagttatcgagattttcaggagctaaggaagctaaa**ATG**G 360

AGAAAAAAATCACTGGATATACCACCGTTGATATATCCCAATGGCATCGTAAAGAACATT 420

TTGAGGCATTTCAGTCAGTTGCTCAATGTACCTATAACCAGACCGTTCAGCTGGATATTA 480

CGGCCTTTTTAAAGACCGTAAAGAAAAATAAGCACAAGTTTTATCCGGCCTTTATTCACA 540 CmR

TTCTTGCCCGCCTGATGAATGCTCATCCGGAATTCCGTATGGCAATGAAAGACGGTGAGC 600

TGGTGATATGGGATAGTGTTCACCCTTGTTACACCGTTTTCCATGAGCAAACTGAAACGT 660

TTTCATCGCTCTGGAGTGAATACCACGACGATTTCCGGCAGTTTCTACACATATATTCGC 720

AAGATGTGGCGTGTTACGGTGAAAACCTGGCCTATTTCCCTAAAGGGTTTATTGAGAATA 780

TGTTTTTCGTCTCAGCCAATCCCTGGGTGAGTTTCACCAGTTTTGATTTAAACGTGGCCA 840

ATATGGACAACTTCTTCGCCCCCGTTTTCACCATGGGCAAATATTATACGCAAGGCGACA 900

AGGTGCTGATGCCGCTGGCGATTCAGGTTCATCATGCCGTTTGTGATGGCTTCCATGTCG 960

GCAGAATGCTTAATGAATTACAACAGTACTGCGATGAGTGGCAGGGCGGGGCG**TAA**tttt 1020

tttaaggcagttattggtgcccttaaacgcctggttgctacgcctgaataagtgataata 1080

agcggatgaatggcagaaattcgaaaCTATATTACCCTGTTATCCCTAGCGTAACTgccc 1140 I-SceI

atggcggccgcgggaattcgatatcactagagccgtcaattgtctgattcgttaccaa**TT** 1200

**A**TGACAACTTGACGGCTACATCATTCACTTTTTCTTCACAACCGGCACGAAACTCGCTCG 1260

GGCTGGCCCCGGTGCATTTTTTAAATACTCGCGAGAAATAGAGTTGATCGTCAAAACCAA 1320

CATTGCGACCGACGGTGGCGATAGGCATCCGGGTAGTGCTCAAAAGCAGCTTCGCCTGAC 1380

TAATGCGTTGGTCCTCGCGCCAGCTTAAGACGCTAATCCCTAACTGCTGGCGGAAAAGAT 1440

GTGACAGACGCGACGGCGACAAGCAAACATGCTGTGCGACGCTGGCGATATCAAAATTGC 1500 araC

TGTCTGCCAGGTGATCGCTGATGTACTGACAAGCCTCGCGTACCCGATTATCCATCGGTG 1560

GATGGAGCGACTCGTTAATCGCTTCCATGCGCCGCAGTAACAATTGCTCAAGCAGATTTA 1620

TCGCCAGCAGCTCCGAATAGCGCCCTTCCCCTTGCCCGGCGTTAATGATTTGCCCAAACA 1680

GGTCGCTGAAATGCGGCTGGTGCGCTTCATCCGGGCGAAAGAAACCCGTATTGGCAAATA 1740

TTGACGGCCAGTTAAGCCATTCATGCCAGTAGGCGCGCGGACGAAAGTAAACCCACTGGT 1800

GATACCATTCGCGAGCCTCCGGATGACGACCGTAGTGATGAATCTCTCCTGGCGGGAACA 1860

GCAAAATATCACCCGGTCGGCAGACAAATTCTCGTCCCTGATTTTTCACCACCCCCTGAC 1920

CGCGAATGGTGAGATTGAGAATATAACCTTTCATTCCCAGCGGTCGGTCGATAAAAAAAT 1980

CGAGATAACCGTTGGCCTCAATCGGCGTTAAACCCGCCACCAGATGGGCGTTAAACGAGT 2040

ATCCCGGCAGCAGGGGATCATTTTGCGCTTCAGC**CAT**acttttcatactcccaccattca 2100

gagaagaaaccaattgtccatattgcatcagacattgccgtcactgcgtcttttactggc 2160

tcttctcgctaacccaaccggtaaccccgcttattaaaagcattctgtaacaaagcggga 2220

ccaaagccatgacaaaaacgcgtaacaaaagtgtctataatcacggcagaaaagtccaca 2280

ttgattatttgcacggcgtcacactttgctatgccatagcatttttatccataagattag 2340

cGGATCCtacctgacgctttttatcgcaactctctactgtttctccatacccgttttttt 2400

ggatggagtgaaacg**ATG**CAGTTTAAGGTTTACACCTATAAAAGAGAGAGCCGTTATCGT 2460

CTGTTTGTGGATGTACAGAGTGATATTATTGACACGCCCGGGCGACGGATGGTGATCCCC 2520

CTGGCCAGTGCACGTCTGCTGTCAGATAAAGTCTCCCGTGAACTTTACCCGGTGGTGCAT 2580 ***ccdB***

ATCGGGGATGAAAGCTGGCGCATGATGACCACCGATATGGCCAGTGTGCCGGTCTCCGTT 2640

ATCGGGGAAGAAGTGGCTGATCTCAGCCACCGCGAAAATGACATCAAAAACGCCATTAAC 2700 ccdBR

CTGATGTTCTGGGGAATA**TAA**GAGCTCCCTTGGCATTTAAggtacgcagtcaaaagctct 2760 F1_50ntR

ggctcgcttaaacacataaatcagtctcttctctctctctctttcttcttcaaagagaat 2820

gggtatataccaaaacctgacatgtgtttctttaggtcttgattgatgcaacactctttg 2880

ttgtttgaagatcttgttacagaataccgcttagaaaattgttaacttttcgttaggaca 2940

ttgtttaggtatcaatggcggcaaaaacttttgaaattcgtttgctcattgatctcacaa 3000

cttttttttgcgctttgtttctctcatcgatctcagttttatgtgtttatctctccttgg 3060

cagAGTCCATGGTTGAGCATCTTGATCATCCCGACTTCCCCTGAATTCGCCTACTTTGTC 3120 F1seq3

***CDKF;1:GFP* construct between the gene specific primers F1seq5 and F1seq3:**

GTTTCAGAGCTCTATGTGCCTCCGACAATGAGTGGACCAGATGAGGATTCTCCGAGAAAG 60 F1seq5

TGGAATGATTACAGAGAAATGGATTCAGATTCAGATTTTGATGGCTTTGGACCCATGAAT 120 F1_50ntF

GTAAAGCCTACCAGTAGTGGATTTACAATAGAATTCCCC**ATG**GTGAGCAAGGGCGAGGAG 180

CTGTTCACCGGGGTGGTGCCCATCCTGGTCGAGCTGGACGGCGACGTAAACGGCCACAAG 240

TTCAGCGTGTCCGGCGAGGGCGAGGGCGATGCCACCTACGGCAAGCTGACCCTGAAGTTC 300

ATCTGCACCACCGGCAAGCTGCCCGTGCCCTGGCCCACCCTCGTGACCACCCTGACCTAC 360

GGCGTGCAGTGCTTCAGCCGCTACCCCGACCACATGAAGCAGCACGACTTCTTCAAGTCC 420 GFP

GCCATGCCCGAAGGCTACGTCCAGGAGCGCACCATCTTCTTCAAGGACGACGGCAACTAC 480

AAGACCCGCGCCGAGGTGAAGTTCGAGGGCGACACCCTGGTGAACCGCATCGAGCTGAAG 540

GGCATCGACTTCAAGGAGGACGGCAACATCCTGGGGCACAAGCTGGAGTACAACTACAAC 600

AGCCACAACGTCTATATCATGGCCGACAAGCAGAAGAACGGCATCAAGGTGAACTTCAAG 660

ATCCGCCACAACATCGAGGACGGCAGCGTGCAGCTCGCCGACCACTACCAGCAGAACACC 720

CCCATCGGCGACGGCCCCGTGCTGCTGCCCGACAACCACTACCTGAGCACCCAGTCCGCC 780

CTGAGCAAAGACCCCAACGAGAAGCGCGATCACATGGTCCTGCTGGAGTTCGTGACCGCC 840

GCCGGGATCACTCTCGGCATGGACGAGCTGTACAAG**TAA**CCTTGGCATTTAAggtacgca 900 F1_50ntR

gtcaaaagctctggctcgcttaaacacataaatcagtctcttctctctctctctttcttc 960

ttcaaagagaatgggtatataccaaaacctgacatgtgtttctttaggtcttgattgatg 1020

caacactctttgttgtttgaagatcttgttacagaataccgcttagaaaattgttaactt 1080

ttcgttaggacattgtttaggtatcaatggcggcaaaaacttttgaaattcgtttgctca 1140

ttgatctcacaacttttttttgcgctttgtttctctcatcgatctcagttttatgtgttt 1200

atctctccttggcagAGTCCATGGTTGAGCATCTTGATCATCCCGACTTCCCCTGAATTC 1260 F1seq3

GCCTACTTTGTC 1272

***CDKF;1:PIPL* construct between the gene specific primers F1seq5 and F1seq3:**

GTTTCAGAGCTCTATGTGCCTCCGACAATGAGTGGACCAGATGAGGATTCTCCGAGAAAG 60 F1seq5

TGGAATGATTACAGAGAAATGGATTCAGATTCAGATTTTGATGGCTTTGGACCCATGAAT 120 F1_50ntF

GTAAAGCCTACCAGTAGTGGATTTACAATAGAATTCCCC**ATG**GGTCATGATGATCATCAC 180 **PIPL:**

CATGGTCATGACTGCCATGATCACCACAATGAGCATGAGCATGAGCATGAACACGAGCAT 240 CobW

CACCATTCTCATGATCACACCCATGACTGGTCTCATCCTCAGTTCGAAAAAGGAGGTGGA 300 StrepII

TCTGGTGGAGGTTCTGGAGGTGGATGGTCTCACCCACAATTTGAGAAGGGATCTTATCCA 360 **HA**

TACGATGTTCCAGATTATGCT**TGA**CCTTGGCATTTAAggtacgcagtcaaaagctctggc 420 F1_50ntR

tcgcttaaacacataaatcagtctcttctctctctctctttcttcttcaaagagaatggg 480

tatataccaaaacctgacatgtgtttctttaggtcttgattgatgcaacactctttgttg 540

tttgaagatcttgttacagaataccgcttagaaaattgttaacttttcgttaggacattg 600

tttaggtatcaatggcggcaaaaacttttgaaattcgtttgctcattgatctcacaactt 660

ttttttgcgctttgtttctctcatcgatctcagttttatgtgtttatctctccttggcag 720

AGTCCATGGTTGAGCATCTTGATCATCCCGACTTCCCCTGAATTCGCCTACTTTGTC 777 F1seq3

**CDKF;1-GFPPIPL (89.3 kDa)**

MDKQPATSWSIHTRPEIIAKYEIFERVGSGAYADVYRARRLSDGLIVALKEIFDYQSAFR 60

EIDALTILNGSPNVVVMHEYFWREEENAVLVLEFLRSDLAAVIRDGKRKKKVEGGDGFSV 120

GEIKRWMIQILTGVDACHRNLIVHRDLKPGNMLISDDGVLKLADFGQARILMEHDIVASD 180

ENQQAYKLEDKDGETSEPPEVIPDYENSPRQGSDGQEREAMSKDEYFRQVEELKAKQVVR 240

DDTDKDSNVHDGDISCLATCTVSEMDDDLGRNSFSYDADEAVDDTQGLMTSCVGTRWFRP 300

PELLYGSTMYGLEVDLWSLGCVFAELLSLEPLFPGISDIDQISRVTNVLGNLNEEVWPGC 360

VDLPDYKSISFAKVESPLGIEGCLPNHSGDVISLLKKLICYDPASRATTMEMLNDKYLSE 420

EPLPVPVSELYVPPTMSGPDEDSPRKWNDYREMDSDSDFDGFGPMNVKPTSSGFTIEFPM 480

VSKGEELFTGVVPILVELDGDVNGHKFSVSGEGEGDATYGKLTLKFICTTGKLPVPWPTL 540 GFP

VTTLTYGVQCFSRYPDHMKQHDFFKSAMPEGYVQERTIFFKDDGNYKTRAEVKFEGDTLV 600

NRIELKGIDFKEDGNILGHKLEYNYNSHNVYIMADKQKNGIKVNFKIRHNIEDGSVQLAD 660 CobW

HYQQNTPIGDGPVLLPDNHYLSTQSALSKDPNEKRDHMVLLEFVTAAGITLGMDELYKVD 720 StrepII

MGHDDHHHGHDCHDHHNEHEHEHEHEHHHSHDHTHDWSHPQFEKGGGSGGGSGGGWSHPQ 780 HA

FEKGSYPYDVPDYA. 795

**CDKF;1-GFP (80.67 kDa)**

MDKQPATSWSIHTRPEIIAKYEIFERVGSGAYADVYRARRLSDGLIVALKEIFDYQSAFR 60

EIDALTILNGSPNVVVMHEYFWREEENAVLVLEFLRSDLAAVIRDGKRKKKVEGGDGFSV 120

GEIKRWMIQILTGVDACHRNLIVHRDLKPGNMLISDDGVLKLADFGQARILMEHDIVASD 180

ENQQAYKLEDKDGETSEPPEVIPDYENSPRQGSDGQEREAMSKDEYFRQVEELKAKQVVR 240

DDTDKDSNVHDGDISCLATCTVSEMDDDLGRNSFSYDADEAVDDTQGLMTSCVGTRWFRP 300

PELLYGSTMYGLEVDLWSLGCVFAELLSLEPLFPGISDIDQISRVTNVLGNLNEEVWPGC 360

VDLPDYKSISFAKVESPLGIEGCLPNHSGDVISLLKKLICYDPASRATTMEMLNDKYLSE 420

EPLPVPVSELYVPPTMSGPDEDSPRKWNDYREMDSDSDFDGFGPMNVKPTSSGFTIEFPM 480

VSKGEELFTGVVPILVELDGDVNGHKFSVSGEGEGDATYGKLTLKFICTTGKLPVPWPTL 540

VTTLTYGVQCFSRYPDHMKQHDFFKSAMPEGYVQERTIFFKDDGNYKTRAEVKFEGDTLV 600 GFP

NRIELKGIDFKEDGNILGHKLEYNYNSHNVYIMADKQKNGIKVNFKIRHNIEDGSVQLAD 660

HYQQNTPIGDGPVLLPDNHYLSTQSALSKDPNEKRDHMVLLEFVTAAGITLGMDELYK. 719

**CDKF;1-PIPL (62.2 kDa)**

MDKQPATSWSIHTRPEIIAKYEIFERVGSGAYADVYRARRLSDGLIVALKEIFDYQSAFR 60

EIDALTILNGSPNVVVMHEYFWREEENAVLVLEFLRSDLAAVIRDGKRKKKVEGGDGFSV 120

GEIKRWMIQILTGVDACHRNLIVHRDLKPGNMLISDDGVLKLADFGQARILMEHDIVASD 180

ENQQAYKLEDKDGETSEPPEVIPDYENSPRQGSDGQEREAMSKDEYFRQVEELKAKQVVR 240

DDTDKDSNVHDGDISCLATCTVSEMDDDLGRNSFSYDADEAVDDTQGLMTSCVGTRWFRP 300

PELLYGSTMYGLEVDLWSLGCVFAELLSLEPLFPGISDIDQISRVTNVLGNLNEEVWPGC 360

VDLPDYKSISFAKVESPLGIEGCLPNHSGDVISLLKKLICYDPASRATTMEMLNDKYLSE 420

EPLPVPVSELYVPPTMSGPDEDSPRKWNDYREMDSDSDFDGFGPMNVKPTSSGFTIEFPM 480 CobW

GHDDHHHGHDCHDHHNEHEHEHEHEHHHSHDHTHDWSHPQFEKGGGSGGGSGGGWSHPQF 540 StrepII

EKGSYPYDVPDYA. 554 HA

**(c)**

***CDKD;1:GFPPIPL* insertion in the EcoRI-BamHI sites of pGAPKm and pGAPHyg vectors**

**EcoRI**

**GAATTC***acgaaggcaagaccagccaaataagtttctacacatacaacaccaagagacctc 60* D1Flank1-EcoRI

*atgtttgaagacatactatgtgtctacaagattatcttattgtgctgaaaccatagactg 120*

*aaagtctgaaacatcgaaggctggttgatagttcttatgaaaacttttggaccggatcga 180*

*catggtgatctaagttttcggaattttgtttttgggatcagtttacctgtgtttttcgga 240*

*tatttttgagtaagtttactctttaaactagcacataactgtatctcgatttcttttggg 300*

*ttggagactcaaagttcgagactcacaactgtagcacatgtaacttgtttatggaagtat 360*

*aacaatattaaatttaaaacaaacaaaaatcataatctttttagtaaactaaaaattgtc 420*

*gaattatatgtttaaaacatcatttttttaagtgaaatttggtttacatgatgactccct 480* ***Flank1***

*taaaagaaattgtttagctagggttcagtttcaattattctgcctaaaaccatttcttta 540*

*tcaatcaaactataataacaaaatttgctgtagtttacgttcgatttcaaattatatggc 600*

*caactcctacaggtgatccgaaaagacattcaatttattgatatgtgttatttttatatc 660*

*ttaatcttaacttttattaacttttaatgttcttcaagtttataatcttcattgtgtaaa 720*

*gacaggaatcaagtactcgagttgacaaatgaatcgtataagttagtgaatatctttggt 780*

*cctttaaatccgatccgatcatcattttacatatatgacagaaaactattttcgcaatta 840*

*actaaaggactgtgattgtagaacaggatttagttgttagattagatatgagaataatca 900*

*tacaaagatagtcagtgcatatggcagcaaagattgcaaaatttaaaagtctccaaagaa 960*

*ccaaggatcttctcatctacccgaggcagttgcataaaccctagttttttacaagaaaaa 1020*

*caaataacacgctaaacattgcttcggaatc*aaaataataaccatataattctcaaatta 1080 D1FLank1_SalI

tagcgaataatccttaccaaaaagaaattataacggaaaattaaagatgaatccgaataa 1140

ataattgttgagtagaaataaagaaaaataaaaaatagagaaggtgaaagaagatgtaga 1200

gctcccttcaatccaaagaagagtaaaagccattaaagggcaagttaaagctcctgagat 1260

atgcaatccatataaccaaatccttttttactttacaaggattagggcttatgggatcca 1320

tagcttagcagcttttctttaccctgctcaactcatgtttgaactttaaggagctctact 1380

tccatcgtcagatcaagatctatcgaaaaacaaagtatcttgtcaaatatattatGGATC 1440

TGACATAAGCAAACTGTAAAGACCTTTAACTCTTCATAAAACAATGAGAAAGTTTTGATG 1500 AT1G73687

AGAACGTGGATTAACAAAAACACCAAAAGAGGTAAATCTCTTACATTAGAGCTATGAAGG 1560 **miR159a**

AGATATAAAGACAGAACAAAAGAGAGGAGGAACCCTAATTATTAACCCTAAACATACTT**A** 1620

**GAGAGAAGGAAAGAGAGAGAGAGAAGAG**GCCACGTCATGTTTtggaagaaatgatagata 1680

gagagtcgtaattatagtggtttagatattcgtgatagaagaataaaaagaaaagaaaaa 1740

aaggaacaggtgggtgcatctgaaaagacatgggaagggtctgacattgtttgtgttaga 1800

ttccctacgcgtcacatgcgtccttctcggcttttgtgcttcactctttttttttttggg 1860

taaagataataaaaataacaattattatcgttcctgccaactactagtccactacatttt 1920

taagatgtaactttgaaaatgaatttggccgaccattctttatttcttttagaaaataat 1980

gtacaagtgtttccgtttggtgacctaattctaaatcatttgttagccctaacaactaga 2040

aatttcaacacgaaatagtgaaataggttgatgtacggtcaggattataagtctgatgat 2100

gtgggcacgaaagcaggtaattatttctctgtctgagcagaatctattgctagatttatt 2160

acaacgaacttgtcttattaccctcacgagtctcgtctctttcacaattcaatggcttat 2220

ttttaagtgtgtttttttatccatcgtgtaagtttttttttgagttgtttaaataaaaat 2280

ttgtatgatgaaaaatgttcactagcaaaatacttgaccctgaagtatcctaaatttttc 2340

tggttcatactcaaatgttaggcttactttgttttctttggtcacctaaaaaaaagtgga 2400

ctgatcagcctaattatttgtgcaaactttaatgattgggctgcatctctttcttatgag 2460

cccattatatgggcccagaagttaacagttgcttgtctaaccttctcggaatagttattt 2520

gggccttatatttgggcttgattccacgcttcatgaaacggaagttaatccggtagtgtt 2580

ctcctggtccatgctgcggaaaatatgagcaaggttaatttggcaacttgttgatgcagc 2640

tttttattttcttggattttttttttaatttctaagtatgtttaggcaatgttccaatat 2700

tcatacagctaaaaaaatgttaatattcacaatcatcttttgtttaaccactaacttata 2760

acattttaaaccgaagaataattgacgagtcaatgttgagtaaaccggaacaattttgct 2820

tttcatttataaacctttaaacgacgtcgttacgatagacgcttccagcttcgttttatt 2880

tctagctttagagcgtctctttcctttttcGTTTACTTTTCAATATTTTCCCCTAAAAAT 2940

GTCGATTCCTTCTGTAATTGCTCTAACGGCTACTTTTTCTCCAGAGCATCATAAGATACC 3000

TCAACGTTAACGATAACAAATTAATTAAGATCAGTTTCGATCAATTGCTTGTGATCCGAT 3060 **CDKD;1**

TAGAGAGTTCCGGAG**ATG**GAACAGCCGAAGAAAGTTGCTGATAGGTATCTAAAGCGAGAG 3120 AT1G73690

GTTCTTGGTCAAGGTACTTATGGAGTCGTCTTCAAAGCTACTGATACAAAGgtgaagatg 3180

attcctctctcaattcttcatattttttgtcgatcccatcgaaactagggttccaattct 3240

gtcgtgtgtcttttgacgtgtctcttcctgggaacaatttggttaaatctgatagctctt 3300

aggttttaaatcagctaaagtttcgatctttacactaatatgtgttcttagaagtcaaag 3360

tttgcatttttatgttcttaatggatttaaagtgtgtagtttttgtttaagtttgtataa 3420

ggaagctgatttgttttgttgtggcaatttgtagAATGGAGAAACTGTAGCGATCAAGAA 3480

AATAAGACTTGGTAAAGAGAAAGAAGGTGTGAATGTAACAGCTCTTAGAGAAATCAAATT 3540

ACTTAAAGAGCTTAAGCATCCACATATAATTGAGTTGATTGATGCGTTTCCTCACAAGGA 3600

GAATTTGCACATCGTGTTTGAGTTCATGGAGACTGATCTCGAAGCAGTTATCCGAGATCG 3660

TAATCTCTATCTTTCGCCTGGTGATGTCAAATCTTACCTCCAAATGATATTGAAAGGTCT 3720

TGAATATTGCCATGGCAAATGGGTTCTGCACAGgtactatcattggaacttagaagcaga 3780

acttgttctatctctaataaggtctgttttgatttaaacttatggatgatcattcgtatt 3840

tgtttcagAGATATGAAGCCAAACAACTTGTTGATAGGACCCAATGGACAGCTGAAACTT 3900

GCAGATTTTGGGTTAGCACGTATATTTGGTAGCCCAGGTCGTAAGTTTACCCACCAGGTa 3960

tgctttgtttgttgtgacaatgctatttatgtagtagttctgctatctttcgtatcttga 4020

tgtcctttagcaattttcataggtGTTTGCTAGATGGTATAGAGCACCTGAACTTTTGTT 4080

TGGTGCAAAACAATATGATGGTGCAGTTGATGTTTGGGCTGCTGGCTGCATTTTTGCTGA 4140

ACTTCTATTACGCAGACCATTTCTTCAGgtgaacctccattttgattgtgtagtaatcca 4200

gtgcatctgtctacttgtttatctaacttacttattccattgcagGGAAACAGTGATATT 4260

GATCAATTAAGCAAAATCTTTGCTGCCTTTGGGACTCCAAAAGCAGATCAGTGGCCTGAC 4320

ATGATCTGCCTTCCTGATTATGTAGAGTATCAATTTGTCCCTGCTCCTTCTTTACGTTCT 4380

TTACTCCCAACGGTTAGTGAGGATGCTTTAGATTTGTTGTCAAAGATGTTCACCTATGAC 4440

CCCAAGTCTAGAATATCGATTCAGCAGGCTCTAAAACACAGGTACctaaaaatctcccct 4500

atggataatgcataagaacaggttatttagttttgttctcaaaccttgatttctttttaa 4560

aggtacTTCACATCTGCACCTTCACCTACTGACCCTTTAAAGCTCCCAAGACCAGTTTCC 4620

AAGCAAGATGCTAAGTCATCTGATAGTAAACTTGAAGCCATTAAAGTGCTGTCACCAGCA 4680

CATAAGTTTAGAAGAGTGATGCCTGACCGAGGAAAGTCTGGTAATGGTTTCAAGGACCAG 4740

AGTGTTGATGTCATGAGACAAGCTAGCCATGATGGACAAGCACCAATGTCTTTAGATTTC 4800

ACCATCTTAGCTGAGCGGCCACCAAACCGACCAACCATCACCAGgtaaaaacaattggtt 4860 D1seq5

ttattacagtcagttcttggtcagcttaaaaaatgaaaacttttgatgaattcttgattt 4920

ttggatttgcagTGCAGATAGATCTCATCTGAAGAGGAAACTTGATCTCGAGTTCCTA**AT** 4980

**G**GTGAGCAAGGGCGAGGAGCTGTTCACCGGGGTGGTGCCCATCCTGGTCGAGCTGGACGG 5040

CGACGTAAACGGCCACAAGTTCAGCGTGTCCGGCGAGGGCGAGGGCGATGCCACCTACGG 5100

CAAGCTGACCCTGAAGTTCATCTGCACCACCGGCAAGCTGCCCGTGCCCTGGCCCACCCT 5160

CGTGACCACCCTGACCTACGGCGTGCAGTGCTTCAGCCGCTACCCCGACCACATGAAGCA 5220

GCACGACTTCTTCAAGTCCGCCATGCCCGAAGGCTACGTCCAGGAGCGCACCATCTTCTT 5280 GFP

CAAGGACGACGGCAACTACAAGACCCGCGCCGAGGTGAAGTTCGAGGGCGACACCCTGGT 5340

GAACCGCATCGAGCTGAAGGGCATCGACTTCAAGGAGGACGGCAACATCCTGGGGCACAA 5400

GCTGGAGTACAACTACAACAGCCACAACGTCTATATCATGGCCGACAAGCAGAAGAACGG 5460

CATCAAGGTGAACTTCAAGATCCGCCACAACATCGAGGACGGCAGCGTGCAGCTCGCCGA 5520

CCACTACCAGCAGAACACCCCCATCGGCGACGGCCCCGTGCTGCTGCCCGACAACCACTA 5580

CCTGAGCACCCAGTCCGCCCTGAGCAAAGACCCCAACGAGAAGCGCGATCACATGGTCCT 5640

GCTGGAGTTCGTGACCGCCGCCGGGATCACTCTCGGCATGGACGAGCTGTACAAGGTCGA 5700 PIPL:

C**ATG**GGTCATGATGATCATCACCATGGTCATGACTGCCATGATCACCACAATGAGCATGA 5760 CobW

GCATGAGCATGAACACGAGCATCACCATTCTCATGATCACACCCATGACTGGTCTCATCC 5820 StrepII

TCAGTTCGAAAAAGGAGGTGGATCTGGTGGAGGTTCTGGAGGTGGATGGTCTCACCCACA 5880 HA

ATTTGAGAAGGGATCTTATCCATACGATGTTCCAGATTATGCT**TGA**GATATCGCGTAACA 5940

GGCTTCTTCTTGACGTCGTTCTTCAGGTTCCTATAGCCTATAGGATCTCTCTTACTGGTT 6000 D1_50ntR

GGTTCCGTTCAGTAATTCCTGAAATTTTTCATATCACAACTAGTGATGACGAATAGTTTG 6060 D1seq3

AATCATTCTTTCTGTCTAAAATAAAGAATATAAAGCACAGATGATTAACTAATCATCATG 6120

TACACCCTTCATAGAAAAATCTCTGTTTTTGGCCTATGTTTAGAAGAGAGCTCTGATCCC 6180

AAACTCATTcttcgttcatttccaatttaaagtccagaatttttagtatcgaacacgttg 6240

gtgtattacatatatgaatttttgatctcattacactaatgctcgatGAGAATGGAAACA 6300

CATCCAAGGAATTTCAATAACTATGTAACAATGGTAAAGTTTTCTAAAGACCAATGTACG 6360

GCCACATCAATTAGAAATTCCCAATAAGTTCATATGGGCATACAAGAATAAAGCACTCAT 6420

TAAGCTCATTTGCAACTGCTTTTTCCTCAGACGTTTATTGA**TCA**TTGGACGTCACCATCT 6480 AT1G73700

TTGTCATCTGAAGATCCGACTCTGTTCGTAGCTTTCTTTGCCTaatcatgtaaaacaaga 6540

aacagaatcacattatggcagagttattatacatataaaactgagccgatcagttaaaag 6600

gttttacctCTTTATCCCAGTTTGTGAATATAGTGACTAAGGAAAGACACAACACTTGAA 6660

CAGACAATGCTGTTACGATTCCCAGCCAAAGCCCCtacaatatgaaaataccacaaaata 6720

aaagtttctataaaactagtcttgtgtttgaacttgagaatgagaaggagagtttaagat 6780

tattaccCGACCACCAATGTGGAAATGGAAACCAAGTAACAATCCTAATGGAACTCCAAC 6840

AAGATAATATGATCCAAGATTCACACATGCTCCAATTTTCTGCCATCCACATCCTCTAGC 6900

AACCCCTGcaaccataaatcaaaactatttgaccaggattcgaaccggacatatataaac 6960

aaggaaccaaagccaatcttctatcattcaaaacctgAGAGTACGCATTGTAAGCCATCA 7020

AGGAAGTTTCCACATGCAACAATCGGTATCATTGATGCGGCATATGCGATGATTTTCGGG 7080

TCGCTGCTAAAAGCATGACCTAAGATCTTCCGAATCGACAACAAAACTGTTACAACCACA 7140

ATTCCCTCGGCTACTGCTATGCCTACAATGACATATACAGCCAGTTTTGCCACTTGTGGA 7200

TTCCCTGCTCCTAACTCGTTTGAGACCCTTATGCTgcaagttaacagaaacacacaaagt 7260

gagataggttggtttgatgagatttttctttttatgagctcttggatacattttgttact 7320

gacctTGCAGCGCCACCAAGACCAACCGAAATCTGCCAGATTGTTAGGGAAGTATTTAGA 7380

Ctgaaaagttgcagaaatgaggttgatcaaatataaagaagaagtacacaaactctgagt 7440

accataagacaaagataaccgaaatctaccaGATTGAAAGCACCGAAGTTTCTAAAACCG 7500

GGTTAGGAAGAAGGCCTGAGGCAAGAACCAGAAGCTCGAAGGACCAAAGCTCCAAACtgt 7560

ttcaaacagaatttgattaaaatgcagatactggaacctgaacaatgcagcattttatgt 7620

ttgatgacttaccAGACCATGACCGCTGAAGGAAAAGCAATTTTAGAGAAATCATAAAGT 7680

TCTTGAAAAGCCTCTTTCGAAAACCCGGTCCAACTATGCGAGCAAGAAGGCGAAAACTTG 7740

ACATAACACGAGAGGAGAATGACGTTGAACCAGTAAGAGACCGAAATAGCAAGAGCAGCT 7800

CCTCTGTATCCTAAACCAGTCTTCAACACAAACAACCAACAGAGAAGCAAATGAAGACAT 7860

GTAGTGATACCAGAGCAGACAAAGACAGGGAACACATTGTTCTGCGCTTGCAAGAACCTG 7920

TTGATACATTGAAGCAGTCCGTAGGCGAAGAGGCTCGGGATCATGTATTTAGCGTAGGAG 7980

CCAGCAACACTTGCAATGGACTTGTCTTGATGGACCAATACGAGAATCTGTTCTGTGTTA 8040

GCCCAAATGATTGAGAGAGGAACAGAGAGTATAAGAAGTACAAACATTGCTCTCTGCATT 8100

TGAATTCCCAATTTTCCATATAGTTTTGCTCCGTAAGCTTGGCCACATAGTGTCTCCAAT 8160

GCACTTGCTGTTCCCAActgtttcacatacacacaaactttgaacaaagttacaaaacag 8220

aggataactaaggttatgttattgaatgcactctttttttttttttgactggatcgtgag 8280

agatgggacgaagaaacgtacGAGGAAGGTGAAGCCGGTAACGGA*GGCAAAGGAGGTGGC 8340* D1Flank2_SalI

*GATAGAGGCGGCAGAGAGAGGAAGAGAGCCAAGATGGCCAACGAACATGACGGAGATGAC 8400*

*TTGGAGAGAGTATTGAAGAAGGCTAACACCAATGAGTGGAGCAGAAAGCCATAGCTGCTT 8460*

*CTTCACCTCCTCCTTCACACGAATCATCGTCGTGTCTTTCTCTGTGATCAGAAGCGGAGG 8520*

*CGTCACACCATCTTC****CAT****TTTCTCTCTGTTTCTCACaaaaaaatatgtcaaaaaaaaact 8580*

*aagagaaatagaataacaacaaactatctctctctttgttcctagtctttagggtttgtg 8640*

*gagccgtcgttgatatttatgcattttttaaggaatgaaatcttttaattatgtggtaaa 8700*

*tagaaattaatttcttttctacttccccctgaggcagattatattttgttttattaaatg 8760* ***Flank2***

*acaacaacaattgaaagatgctcacaattcacaaccacgcatttttttttatattcaaag 8820*

*cttccgattatatagacaatgacgtgagtggctcctagttgctcttctcttattctattc 8880*

*tccatcgagtcaggagaccacctcagactctgtctccggtattactagcaaaactctcct 8940*

*ttagctttcttcaccaaaagttgttatgattacataacctatgaggagattggaaactat 9000*

*gaggagaggcaccattattgctacatattacagagattaaggcaatttccgagaaagtat 9060*

*tgaggattttagtactaatgtacagaaagttacaaaagagtgtgtttaccaagtggggaa 9120*

*aactgaagtttccaaaacaggattttggaagtaaagcctgagactgagaggaagaccatt 9180*

*ttcttgtatgaccaaatcgttatgattccatcaagatatgatcaacattaaagatatgat 9240*

*atatacaagaagacatgaatcatatatgtgtaccaccagtctaaccaaaccatgaatg***GG** 9300 D1Flank2_BamHI

**ATCC**

**BamHI**

***CDKD;1:GFP* construct between the gene specific primers F1seq5 and F1seq3:**

GCCACCAAACCGACCAACCATCACCAGgtaaaaacaattggttttattacagtcagttct 60 D1seq5

tggtcagcttaaaaaatgaaaacttttgatgaattcttgatttttggatttgcagTGCAG 120 D1_50ntF

ATAGATCTCATCTGAAGAGGAAACTTGATCTCGAGTTCCTA**ATG**GTGAGCAAGGGCGAGG 180

AGCTGTTCACCGGGGTGGTGCCCATCCTGGTCGAGCTGGACGGCGACGTAAACGGCCACA 240

AGTTCAGCGTGTCCGGCGAGGGCGAGGGCGATGCCACCTACGGCAAGCTGACCCTGAAGT 300

tCATCTGCACCACCGGCAAGCTGCCCGTGCCCTGGCCCACCCTCGTGACCACCcTGACcT 360

ACGGCGTGCAGTGCTTCAGCCGCTACCCCGACCACATGAAGCAGCACGACTTCTTCAAGT 420 GFP

CCGCCATGCCCGAAGGCTACGTCCAGGAGCGCACCATCTTCTTCAAGGACGACGGCAACT 480

ACAAGACCCGCGCCGAGGTGAAGTTCGAGGGCGACACCCTGGTGAACCGCATCGAGCTGA 540

AGGGCATCGACTTCAAGGAGGACGGCAACATCCTGGGGCACAAGCTGGAGTACAACTACA 600

ACAGCCACAACGTCTATATCATGGCCGACAAGCAGAAGAACGGCATCAAGGTGAACTTCA 660

AGATCCGCCACAACATCGAGGACGGCAGCGTGCAGCTCGCCGACCACTACCAGCAGAACA 720

CCCCCATCGGCGACGGCCCCGTGCTGCTGCCCGACAACCACTACCTGAGCACCCAGTCCG 780

CCCTGAGCAAAGACCCCAACGAGAAGCGCGATCACATGGTCCTGCTGGAGTTCGTGACCG 840

CCGCCGGGATCACTCTCGGCATGGACGAGCTGTACAAG**TAA**GATATCGCGTAACAGGCTT 900 D1_50ntR

CTTCTTGACGTCGTTCTTCAGGTTCCTATAGCCTATAGGATCTCTCTTACTGGTTGGTTC 960 D1seq3

CGTTCAGTA 969

**CDKD;1:GFPPIPL (80.72 kDa)**

MEQPKKVADRYLKREVLGQGTYGVVFKATDTKNGETVAIKKIRLGKEKEGVNVTALREIK 60

LLKELKHPHIIELIDAFPHKENLHIVFEFMETDLEAVIRDRNLYLSPGDVKSYLQMILKG 120

LEYCHGKWVLHRDMKPNNLLIGPNGQLKLADFGLARIFGSPGRKFTHQVFARWYRAPELL 180

FGAKQYDGAVDVWAAGCIFAELLLRRPFLQGNSDIDQLSKIFAAFGTPKADQWPDMICLP 240

DYVEYQFVPAPSLRSLLPTVSEDALDLLSKMFTYDPKSRISIQQALKHRYFTSAPSPTDP 300

LKLPRPVSKQDAKSSDSKLEAIKVLSPAHKFRRVMPDRGKSGNGFKDQSVDVMRQASHDG 360

QAPMSLDFTILAERPPNRPTITSADRSHLKRKLDLEFLMVSKGEELFTGVVPILVELDGD 420

VNGHKFSVSGEGEGDATYGKLTLKFICTTGKLPVPWPTLVTTLTYGVQCFSRYPDHMKQH 480

DFFKSAMPEGYVQERTIFFKDDGNYKTRAEVKFEGDTLVNRIELKGIDFKEDGNILGHKL 540

EYNYNSHNVYIMADKQKNGIKVNFKIRHNIEDGSVQLADHYQQNTPIGDGPVLLPDNHYL 600

STQSALSKDPNEKRDHMVLLEFVTAAGITLGMDELYKVDMGHDDHHHGHDCHDHHNEHEH 660

EHEHEHHHSHDHTHDWSHPQFEKGGGSGGGSGGGWSHPQFEKGSYPYDVPDYA. 714

**CDKD;1:GFP (72.06 kDa)**

MEQPKKVADRYLKREVLGQGTYGVVFKATDTKNGETVAIKKIRLGKEKEGVNVTALREIK 60

LLKELKHPHIIELIDAFPHKENLHIVFEFMETDLEAVIRDRNLYLSPGDVKSYLQMILKG 120

LEYCHGKWVLHRDMKPNNLLIGPNGQLKLADFGLARIFGSPGRKFTHQVFARWYRAPELL 180

FGAKQYDGAVDVWAAGCIFAELLLRRPFLQGNSDIDQLSKIFAAFGTPKADQWPDMICLP 240

DYVEYQFVPAPSLRSLLPTVSEDALDLLSKMFTYDPKSRISIQQALKHRYFTSAPSPTDP 300

LKLPRPVSKQDAKSSDSKLEAIKVLSPAHKFRRVMPDRGKSGNGFKDQSVDVMRQASHDG 360

QAPMSLDFTILAERPPNRPTITSADRSHLKRKLDLEFLMVSKGEELFTGVVPILVELDGD 420

VNGHKFSVSGEGEGDATYGKLTLKFICTTGKLPVPWPTLVTTLTYGVQCFSRYPDHMKQH 480 GFP

DFFKSAMPEGYVQERTIFFKDDGNYKTRAEVKFEGDTLVNRIELKGIDFKEDGNILGHKL 540

EYNYNSHNVYIMADKQKNGIKVNFKIRHNIEDGSVQLADHYQQNTPIGDGPVLLPDNHYL 600

STQSALSKDPNEKRDHMVLLEFVTAAGITLGMDELYK. 638

**(d)**

***CDKD;2:GFPPIPL* insertion in the EcoRI-BamHI sites of pGAPKm and pGAPHyg vectors**

**EcoRI**

**GAATTC***gcacaggaaaacgagggaagggacctttttccggttttcaaattcaacctgtaa* 60 D2Flank1-EcoRI

*ttgctcatcataaattctcttggtcatacgtggtgtatggtctttttatggttttaggtg 120*

*gttgccactgatagaataaatggaaacaaactcatgtcattccaagagttatcaacaaga 180*

*gagagagggagcaaagatgctttgataactacagaaagtattaaggtattgaacacaata 240*

*tctatacaacccaattgaatgttagtatggaagttccacatcgttttacaatgagattta 300*

*tttttctcagtatattcgacgaaagttttccttttcatcctattgtatcctttgaataca 360*

*aaattcttagtccattgtcatttttgtagttttcaatatgttcttctagatgtatttctt 420*

*ttcttctaatggctttctgcaggttgaagtctgcgtcttcgtgtttgatattatgtttgt 480*

*caatggagaacagtatgagtcttttactcactatttatattttcatttgcatatatccaa 540* ***Flank1***

*atacttatatgttgagttgtaatttacttagcgtatcaatgcaggctgttggctcttccc 600*

*ctccgtgagagacgaagacgtgattcccatcattttcttttttccttaacttgatgcatt 660*

*cattttctctaccaggttcatatatgtccactcttatcttacttggttttgcttgaactt 720*

*acaggtctaaaggaggtcttccctgagactaggccaggttatcttgagtatgctaaggaa 780*

*ataactgtaagtgctacggtagtttctcacctagaagttataggctatagccttcttctg 840*

*tatctaagtgttttggtattttcattgaactgatcattgtttgatttttataggtgggag 900*

*cagaagaagcgtctctgaataatcatgacacattgagtaggataaatgcttttcttgaag 960*

*aagcatttcagtcatcttgtgaaggaatcatggtcaagtctttagatgtaaatgctggat 1020*

*actgccccacaaagcgctctgattcatg*gctcaaggtctctttgctgtaaattatcttac 1080 D2FLank1_SalI

taaaacataatgtgatggaaatggattaaataatgcttttcttttctggctcaggttaag 1140

cgagattatgtagatggattgggcgatacattggatttagttcctattggtgcttggtat 1200

ggcaatgggagaaaagcaggatggtagtaaaaacatattttccaactttctctttttttt 1260

tttctgaaaagcagaattgtaatcataagtttactggtaatttctcaaatgcatcttgat 1320

gaattctgatgttttttatatattttgtgtgtaggtatagtccattccttatggcctgct 1380

tcaaccctgagactgaagaattccagagtgtctgccgtgtcatgtctggtttttctgatg 1440

ccttttacatcgaggtaatgaaaagctcaactctaacgttgttgtttaatgatgaaacag 1500

atggggtagtaaatacgttgggtttggtctatacttggactcctaattgtcacgtgagaa 1560

ccatttcaagcttgattctactttcttattttgcttatcttgcagatgaaagaattctac 1620

tcggaggataagatccttgcgaaaaagccgccttactacagaacaggggagacacctgac 1680

atgtggttttccgcagaggtagtttgggaaatcagaggtgctgatttcacagtctcgcct 1740

gttcacagtgccagtttaggtctcgtccatccatcgcgaggcatctctgttaggtttccg 1800

agatttatcagtaaagtgacagatagaaatcctgaagaatgtagtacggctacagatatc 1860

gcagagatgtttcatgctcaaaccagaaaaatgaacatcacttctcaacattaacacgct 1920

tcctatctgcttttggttcgtttagacttttttttttttttttttttttgctatcaagca 1980

cttgcaaagttgcaagggtgtttaaataggaacctaactcacaaaatattttttgtggtt 2040

tttaatctgtaggtagaatgtgtaaatatttctacctagaactatgtgaatatttaaccc 2100

atctgtatatattatctatgtgcgtcactttttcttttttgatgtcgtaaacacaaatgg 2160

taacactggtaacaaaatattgaaagatagaaaaaaaaaaaagtatataggatatgattg 2220

gtaatctcttatttgtgtcactaatcgattgaattctatatttttttcgtatttatgtgt 2280

tggacactaaaattatcaaatatttagcaaatttccctgctgcatttcgtgagttaaagt 2340

attcctcccgccaattcaacttaaccggtagtagccggtcaccattccaacgacaaccgg 2400

ttataagaaagccatccccaaaattgaaaaCATTTTCATCGTCTTCTTAATCAAAAAAAA 2460

AAAAAAAAAAAAACTCTGAAGCTTCTTCTTTGATTAATTCTCTCCTGGGGAAAAAAAACC 2520

CTAGCTCTTCCTTCTTCTCTCTTCTCTTGAATTATCTGCTTTCGAATTTTTTGAAAAGGG 2580

AGAAACTTTTTCATCTGGGTCTCTCTCTCTCCCGAGTTTGGATGAAGTTTATTGAAACCT 2640

AGGGTTTTTCTCCGACTTGGTTGTTGATTAGAGATA**ATG**AGTGCAATCAAAATCACCAAC 2700 AT1G66740

GTCGCTGTATTGCATAATCCTGCTCCTTTTGTTAGCCCTTTTCAGTTCGAGATTTCTTAC 2760

GAGTGTTTGAATTCTCTCAAAGACggtaaaaatctcctctttctccttcccctgaattgt 2820

aattcatctcgactttatttaggttttagtctctgctgattcttatggattgttttcaac 2880

accaatttgttgctgctgattgtaattggaacatattgttacctaattgctttgtcagag 2940

tcatgctctaatggtttgttttctaattgataatctagagtcttgttgtctattagttgg 3000

atttatctatgcacaagttcttggttaacattgttatagggtgaggataacttagataga 3060

cccgtgcttttgcttgtggtacaGATTTGGAATGGAAGCTTATCTATGTAGGCTCAGCAG 3120

AAGATGAGACTTATGATCAACTTCTAGAGAGTGTGCTTGTAGGGCCTGTTAATGTTGGCA 3180

ACTACCGCTTTGTATTTCAGgtaacatatgcactactacctcttccaagcaccaacaaag 3240

tcttggtctaactctctattactactgttacagGCTGATCCTCCGGATCCATCAAAGATT 3300

CAGGAGGAAGACATCATCGGTGTTACTGTGCTATTGTTGACATGTTCTTACATGGGTCAA 3360

GAGTTCTTGAGAGTTGGATATTACGTGAACAATGATTATGAGGATGAGCAACTCAAGGAA 3420

GAGCCTCCAACTAAGGTTTTGATTGATAAAGTTCAGAGGAACATACTTTCCGACAAACCT 3480

AGAGTTACTAAATTTCCTATAGATTTTCATCCAGAAGAAGAGCAGACTGCTGCTACTGCC 3540

GCTCCTCCTGAACAATCTGATGAACAACAACCTAATGTCAATGGTGAAGCTCAGGTTTTA 3600

CCTGATCAGTCAGTAGAACCAAAACCTGAGGAATCA**TGA**TCCTTATACCAAGTCTAGTTG 3660

AAGAAAAGTGGATGAGAATTGAGAACTATTGTCTCCACAGATGTTGCGCTTTGCTTTTCT 3720

GTCTTTCAAAAGCATTATATGCTGTTTGTTGTACTTAATTCTAGAGGCTTTAGGGAAGTG 3780

ATTCTTGACATTTTTGTATGTTTATGTTTTGGGCAAAGGTTTTTTAAATCGAAGCAAAGC 3840

CAACAGTTGCCAAACAAgattctgagttctgatggttggaatttggtaataggtgtagaa 3900

taacttccctagattttctcaaggtacaaagcgacatttgatcttcatcatttgttgaaa 3960

aaagtgccgttaaatggtcatagttagcattgatgatgatgattatcatcatcatcaacc 4020

ataattctttcaccaaaaaaacatgagtccgtgatttttttaggaccgtttcacagttgt 4080

tctgccaaaaatatgagtccgcatttattcaccaaaaaaaaaaaaaaaaaaaaagtagta 4140

cacattacaatttctcgcctaacgattttactctgtggtaaaccgaaagatagttaaaaa 4200

cggctccatttcgattactttgaaaaaaaaaaaatgtttgttttgttaaaagaaaatatc 4260

ttttaaaatattaaaaaatattttattcaaacttttattaatacagatctaattactaaa 4320

ctcaataacattataaaagccgtgttcggctaagcccagtaagccctatatgttttgaat 4380

aaatacgatattttataataaacagattgttgtttttaaatacctaataatacataatat 4440

attcctatacttgacaataacgaataagaattatattacctattattttggatttcgtaa 4500

aattattttatattttttttaggtaaatggtaaatttgtaaaaaatagctactttgatat 4560

ttatattgtgtattattttctactcgataagattaatcatcaatagttcgaggtggaggg 4620

ttaaattatctagggtctgatgatcatgtagaatcacttcctgcaaacaaatcacgagtg 4680

ttagcatatttatgctatccacagtatcatatggctgtttctgattatttcataaatata 4740

aggttcgtttcataaaacacaaaatcttacgggtataaaattgaaaccaaagaattcaat 4800

ttcttaAAAGTGTTTCACTAGGAGCCCTCGTACTTCCTCGAAGTGTACCGGAGAATCTAA 4860

ACCCA**ATG**TCGAAATCCGGCGATAATCAACCAGTTGATAGATACCTCCGCCGTCAAATCC 4920 **CDKD;2**

TCGGAGAAGGAACATACGGTGTCGTTTACAAAGCCACCGACACAAAggtaaaatcaaaaa 4980 **At1g66750**

cccttcccgatttcgttttcaatttcatcaagattttcaaactcaacaatcttcgatttt 5040

gtgatttccttaGACTGGGAAAACTGTAGCAGTGAAGAAGATAAGATTAGGAAACCAAAA 5100

GGAAGGAGTAAATTTCACAGCTTTAAGAGAAATCAAGCTACTAAAAGAGCTGAATCATCC 5160

ACATATCGTTGAACTCATTGATGCTTTCCCTCACGATGGAAGTTTGCATCTAGTTTTTGA 5220

GTATATGCAAACAGATTTAGAAGCAGTGATTCGTGATCGTAATATCTTTTTATCTCCTGG 5280

TGATATTAAGTCTTATATGTTGATGACTTTGAAGGGTTTAGCTTATTGTCATAAGAAATG 5340

GGTTCTTCACAGgtaagaagttatgcttaggtttataaagatttgattttgattgtgttg 5400

atggtttgtttttggtgtggttgttagGGATATGAAGCCGAATAATTTGTTGATTGGAGA 5460

GAATGGTTTGTTGAAGTTGGCTGATTTTGGTTTGGCGAGGTTGTTTGGGAGTCCGAATCG 5520

GAGGTTTACTCATCAGGTATTgatgctatgattgatacggttttgagcttggtagctagg 5580

ttagtgtagttcttgatgatcttattggatttattggttaggtatttatatagttattga 5640

tatttatggttgcattggtagctagattagtctggagtttgtagtaaatatcttggtact 5700

tcttcttgatgatctattttgaattttatgctcaaaatgaagagtcggtacagagtattg 5760

agaagatatcatactttatgtgtgtttggttttggtgaggtgaaagattgtattgacatt 5820

tttttaccctagaagattctgagattgattagttatgaagaaatctcttctattttttgt 5880

ttgaattgacaggtagattagtcttgttcttgatgatctattggtttttttttgctggga 5940

caaactaagagtatatatagtgcattgagaagacatactatgtttttaattcgaatgctt 6000

gcatacctatgttattggtttcggttggtgattacaagtttaagtttccggatagcattt 6060

tgttgattacggtcatattaacattttgactcaagaagattctgcaattgattagttctg 6120

tagaagtctctttttgctggatatggaaaaattgatacagagtattaagaagacatcata 6180

cggttttaattttgattcttgcatacctgtgtttttcgattttggtgagatgagagattg 6240

atggttcaaagtcaccagatacagttttgttacttacggatgtattaacattgttacttt 6300

agaaagattctgagatttattggttgtgtaacttgtgtagatatctcatttggttagcta 6360

tttgtatctgcttgtggaagattcgttaagtttaaattggttatacttattcacacaggt 6420

attTGCTACATGGTACAGAGCGCCTGAGTTACTGTTTGGGAGTCGACAGTATGGAGCAGG 6480

AGTTGATGTTTGGGCTGCAGGCTGTATTTTTGCTGAGTTACTACTTCGTAGACCATTTCT 6540

TCCGgtaatgctctatacccttcgtacttcatgtttaaaaagtctgtagttctctaaaca 6600

tttcctatactttcttcagGGTTCTACTGAGATTGATCAACTTGGAAAGATCTTTCAAGC 6660

GTTTGGTACTCCAGTACCTTCCCAATGGTCTGACATGATCTATCTCCCAGACTACATGGA 6720

ATTCTCCTATACACCCGCTCCACCATTACGTACCATTTTCCCTATGGCAAGTGATGACGC 6780

TTTGGATCTTCTAGCAAAAATGTTCATCTACGACCCACGTCAAAGGATCACAATACAACA 6840

AGCTTTAGACCACAGGTATactcttgaaaaagaaacccacgggacaaaaattctgtttca 6900

tcactctgattatcattggttttttcatcaggtatTTCTCGTCTTCTCCATCACCAACTG 6960

AGCCAGGGAAGCTTCAGATTCCAGCTTCCAAAGGAGACGCACTCGAACCAAAGGCCTCCG 7020 D2seq5

AGCAAAACCAACATGGAAATAGCCCAGCGGTACTGTCTCCTCCTGGTAAAATGAGGAGAG 7080 D2_50ntF

TGATGGGTCCTGAAGGATTCACT**ATG**GTGAGCAAGGGCGAGGAGCTGTTCACCGGGGTGG 7140

TGCCCATCCTGGTCGAGCTGGACGGCGACGTAAACGGCCACAAGTTCAGCGTGTCCGGCG 7200

AGGGCGAGGGCGATGCCACCTACGGCAAGCTGACCCTGAAGTTCATCTGCACCACCGGCA 7260

AGCTGCCCGTGCCCTGGCCCACCCTCGTGACCACCCTGACCTACGGCGTGCAGTGCTTCA 7320 GFP

GCCGCTACCCCGACCACATGAAGCAGCACGACTTCTTCAAGTCCGCCATGCCCGAAGGCT 7380

ACGTCCAGGAGCGCACCATCTTCTTCAAGGACGACGGCAACTACAAGACCCGCGCCGAGG 7440

TGAAGTTCGAGGGCGACACCCTGGTGAACCGCATCGAGCTGAAGGGCATCGACTTCAAGG 7500

AGGACGGCAACATCCTGGGGCACAAGCTGGAGTACAACTACAACAGCCACAACGTCTATA 7560

TCATGGCCGACAAGCAGAAGAACGGCATCAAGGTGAACTTCAAGATCCGCCACAACATCG 7620

AGGACGGCAGCGTGCAGCTCGCCGACCACTACCAGCAGAACACCCCCATCGGCGACGGCC 7680

CCGTGCTGCTGCCCGACAACCACTACCTGAGCACCCAGTCCGCCCTGAGCAAAGACCCCA 7740

ACGAGAAGCGCGATCACATGGTCCTGCTGGAGTTCGTGACCGCCGCCGGGATCACTCTCG 7800

GCATGGACGAGCTGTACAAGGTCGAC**ATG**GGTCATGATGATCATCACCATGGTCATGACT 7860 PIPL:

GCCATGATCACCACAATGAGCATGAGCATGAGCATGAACACGAGCATCACCATTCTCATG 7920 CobW

ATCACACCCATGACTGGTCTCATCCTCAGTTCGAAAAAGGAGGTGGATCTGGTGGAGGTT 7980 StrepII

CTGGAGGTGGATGGTCTCACCCACAATTTGAGAAGGGATCTTATCCATACGATGTTCCAG 8040 **HA**

ATTATGCT**TGA**GAAGATGATGTTTCATTTCTTTTGGAAAATATCAATGGATATGCCCAGA 8100 D2_50ntR

AGATTTCAAAATTAGATTTTTACCATTGATAATTCAATATTAGTTAACTATCAAAGAGAA 8160

AAAATTTAAAGCCATAAATGTCCTTCTTAGTTCTTACTTTGTTTAATTTACCAAATATGT 8220

CATGTATCAACTATTGTTATAATTTCACTATATATCAACTATCTTACcatattaatataa 8280

atattattcagatatttaatatgttagcacgttatattcagtataacatgttattataaa 8340

gttaaattgattatttcatattaatttgtatgatatggtaaatggtttaatacgatatat 8400

aaaaatttatcgtgatactatactagttaacatattaatcaaatagttatatatatttag 8460

cacgatactatactagttaacatgttaatcaaatagttatccacataaatcataaatatt 8520

ttatgcaatccaatgataaatacgacagtgtcgtaaatttcggatgaatagtgtcttcag 8580

gcagttgatgaacagtatcatgtctaaaatatccggtaataaatagtgttttgagacaat 8640

gacatatttagtaaattaaacaaaatgaaaaaggaaatttatgattttaatttttttctc 8700

tttcatacttacctaatattaaactatctataatagatatctaattttcaagtcttccag 8760

gcatatacattaatatagctctttccttttttcctttccttgactataatcactaatgga 8820

gaggtatatacagtaatgactttaatttctagaattgcgtttcgtaatgcttttctacaa 8880

agtagttaccataaaaaaatatcaaacgagaagccatcttttgacctcatccatgtctaa 8940

tatctattgacgttaaattccttcgaaccttaaactgataaaaaaaaattatcttcaata 9000

aacttaaatttatgatataacactgaccgaagaaaaaactaatttttctaaagaaaacac 9060

atttaatgtttaattataattgcactaaattctcactctaaacatttttccatgactatg 9120

aaccaataataattcaaatatatgtacacgtgagtgtcttaatcagtggttgatcatatt 9180

catattgtcaatttgtgattaatattcattgggaagcagtgatcttgttgtatagggact 9240

aggctttcacaatatctgcaatttccactcctattggcgatccatacatatttcattggc 9300

ccactaaaaaaataaattccaaaacaattggcattactacaaatacttactctttcggtt 9360

tcataaagaatgtttttctataattttatatttgttcataaagacttctaaaactaatca 9420

cttattaaatatggactcatgtatatatatatattttttttaatttgtatgaaatgtgtc 9480

aaaatgatattttttcaagccgatgaagtactaaacaatctctatttggtgcggtttggt 9540

ttttgaagttatgattcttttgactcactcttacgatcaggaactcacagtttggacttt 9600

ggacatagaccgtgaccggcccatgagagacacgagttaaactttgaatttagaagaaaa 9660

gagtattaaaaaaatgacctttgtggttaccccactgcaaaacgacctcactcagatgag 9720

ggctaacaaattggaattggaggcagcaattatgccacttggctaagtagttgaggcagt 9780

gacgcggcgatattatccatgtatataatgtgtgaagccaaaaacgattgtaacattttt 9840

ctctgatcttaggtattaatacgagaattcaattttttccgacaatttgttgaaagcttt 9900

ctctgtcaacaataaaatgaacgtgcattaaataccaaggttggctctatctaagaagcc 9960

aattcggttaaccaaagtatttctagagggttacctaaattaaccaaaagaaatccgggt 10020

tcggttttggttagtgtaaaactatttgtttttgtacattttctcatgaaattgcttatt 10080

agtaccatatctccgtaccaatttttttttttattattactttaagagtctcactaagca 10140

attacccatgagataacatggtgtaccgcaataatacaacgttacctatgaaacttcgag 10200

tagttagaaagttttgacaacacctttaatttgatttgaagcatgcaatcaccaaatctc 10260

tagtaactactgttcaaaggttgcttatagtgtggccgagaggtatgccatcatcgactt 10320

cactattcttcgcggaaacaactcttttgcgatagatctcaacttcaccaatcggatctc 10380

tttcatcgattagattgataaagttattccatgtcaaaggaaaaaaacaaagagatagtt 10440

ggattatcaagatgcatgaagtatgtggagtgcttttttaaaccaagtgaacgtaaatat 10500

gaatttgtatacataatacatgtgattagagttagtttaacataagtaaaaaagaaagtt 10560

gaaactatcaaaatgctctgtgtaagtattaattatttcttccaacacacgatgtcagaa 10620

gtcagaaccgattattaatacttttctcaacaacttctgtttctttatttatttttgata 10680

acttcgacgaggataagtaagaaaatggcttcctctttttcagtgtgtataatgcagttc 10740

ttgactggactactctcaattggatatcacggattgtgaatacatattctcggttataac 10800

ttttaactttataagttatacttttatttagtttcacaataagtgtggtttaattcttac 10860

aagtttgtatcaaaataggtattgttttatattttgaatgtagttacagtatttggtgaa 10920

attgcaattctattcttactattttaatgtattgaccaataaaaaaaagttgtataatat 10980

attaataagagataaacaaaaaaaatgtttttactatatgtgaagaacctgtaaatgaca 11040

ttgtgaatacatatattctcggttataacttttaactttataagttatacatttctttct 11100

tttcacaataagtgtcgttttagtgttaaaattttatttcaaaataagtattgttattat 11160

ttttagtgtagatatttggtaaatattcaattttaatgtattgaccaattataaaaaatt 11220

gtataatatattaataagcgatagacaaaaaaatctaattgtttttttactatatgtgaa 11280

gaacctgtaaatgacatttatactaaaacgtaaaaagtatgtatgatatattgaatcaaa 11340

aataatactagtaatcagtagttaagttacatctattttgttaacgaaatagtaaaaaaa 11400

aatgtcattaagagacggatttattattgatatcaagtagagcaaaatttgtttgtttag 11460

aaacacaaacaaaatcttaactttggcggtttagtctgactaatttcaaatttatgttgt 11520

ggagtatataaagtcgatagtactttttaacaatgattttgaaaactaaaactttatgtt 11580

attggagtattatcatggattggagtttttgtcaatgaattaacatggagatgtggatta 11640

ggttaggttcatcgagatatgtatttaaagttgaaacatgtgttttattattattctttt 11700

catttatttatttttttctttctgtcggtcagtattgaaacatgtgtttagtttcatttg 11760

tcctctcaggcctcagcatgtctagtctaggagtattctttatgtttcaaaatataagag 11820

gttttagttaaatatgcagattaagaaatatccatttttaaaaaaattcaaccattcata 11880

aaaatatcatataatataaataattataaaatattaaattaatctatagttatgttacac 11940

aatttacataaatcacgctgtcatgagtagtctttttcatatatcatctattgacaaaat 12000

aacaatttatcttgttgttcttttgaaaactagctattactacatatattatacattttt 12060

tttctcataagtggtttaacgctgacaaaaaaaatctattgtaagatggtaaccactgaa 12120

agccatcttaggttcaatacttaaataaatgatatgatattaaaaatattgatcatggac 12180

cgatgtttatcgatttgagccaatgcaatcaatacgaagagagacttttattccttattc 12240

tttagaaatgtatatctcattttatgactaattaaataataactgatgatcagtatgtgg 12300

ttctatacatttaaaaatcttctaattcagttttttttttttttttggtatcgatgttaa 12360

gtatttataccgttttcaagtttgagacagaatacagagtaaacaatagaagcaaaaaaa 12420

aaaaaaaaaaaaaaaaaagggagaaagactacgactaaaggaactacgaatacaagagat 12480

aacacgataagaaacacaaagcgaagcaactagtaaaggcttgaagaggaaacaccgatt 12540

ctaattcagttttcatatctgtttgatactcttaaggtattatattgttctgtgttccag 12600

ttctatctcatgctaatcaaaaatattgttaataccactcttgcctttttcaagaaccga 12660

catgatttttaagttttaaccacaaatgtatttgattggtaaaaggttctaaaaagtttc 12720

aacaactcacaaagccttaaaactgaaccattcatgttttacttttctttttaaggctat 12780

acaaccaaaacaaactttatgttgtctaattaatttctctggcataaaaaaaaaaaaaga 12840

ctccacaactaaaatcttttaagtacaaactacaaataggaaccaattattagttactag 12900

ttaggatgcatcttcttcttacacgtgctgaattctatcttttgacttaaatgagtgaga 12960

tgataaaagtcgaaaacgatttcgcttttatacgcggaagcggaaacgtgtttaTTTTCT 13020

TTTCTTCGAGTCGCGACGTCGCATATATTATTTATCAAAATACAAATTTTATTATATCAA 13080

AATCATAAAGCTAAATAAATACGACACAACTTCCAACATTTTCTCTAACCAGAAAGAAAA 13140

AAAAA**ATG**AAGAAGAGTATCGAAACTCCGTTATTGTTGAACACCAAACAATCACAAGACG 13200 AT1G66760

AAGATAAAGAAAAAATAAGATGGGAGAAGATGAAGAAAGTTGCTTCAATGGCTGCTCCAA 13260

TGGTC*GCCGTGAACATGTCTCAATACCTTCTTCAAGCAACTTCTACAATGATCGTCGGTC 13320* D2Flank2_SalI

*ACCGGAGCGAACTCGCTCTCGCCGGAATCGCTCTAGGAAGCTCCTTTGCTAATGTTACCG 13380*

*GCTTTGGTGTTCTTgtaagcaagtatatgaacctttatatatatatatgtattggattaa 13440*

*tttgtttctaattattgttgtatactatatagTTTGGACTTTCAGGTTCATTGGAAACAC 13500*

*TATGTGGTCAAGCATATGGAGCAAAACAATATCACAAGCTTGGATCTTACACTTTCACTT 13560*

*CAATAGTTTTCCTTTTGATTATCTCTGTTCCGATTTCGATTCTTTGGATGTTCATGAACC 13620*

*AGATCTTGCTGTTGCTTCATCAAGATCCTCAAATAGCAGAGTTAGCTGGTGTGTACTGTC 13680* ***Flank2***

*TCTGGCTGGTACCAGCTTTATTCGGTTACTCTGTTCTCGAGTCGTTGGTTCGATATTTTC 13740*

*AGTCACAAAGCTTGATTTATCCAATGGTCTTGAGCTCTCTAGCTGCTCTGTCTTTCCATG 13800*

*TTCCTCTCTGTTGGCTAATGGTTCATAAATTTGATTTTGGAGCCAAAGGAGCGGCTGCGT 13860*

*CTATCGGTATCTCTTACTGGCTCAACGCGGTTTTTCTTTGGGTTTATATGAAACGCTCTA 13920*

*GTCGTTGTGTCGAAACGCGGATTTATATGTCCAAGGATGTTTTTGTTCATACAAATATCT 13980*

*TCTTTCAATTTGCCATTCCTTCTGCAATGATGTGTTggtaagtatgtacaactttattaa 14040*

*aaatccatatagaattaaccaagtgatggtagtctagtggtggtcattgttttgtggata 14100*

*ggtgaaagatttaaaatcttaggttacataaataaatgtgtaatggattttttttccctt 14160*

*cactaatattctattgacacttattttcttattgctcttgcttccttcaataGCCTTGAG 14220* D2Flank2_BamHI

*TGGTTAGCTTTCGAGGTCATTACTTTG***GGATCC** 14253

**BamHI**

***CDKD;2:GFP* construct between the gene specific primers D2seq5 and D2seq3:**

CCAGCTTCCAAAGGAGACGCACTCGAACCAAAGGCCTCCGAGCAAAACCAACATGGAAAT 60 D2seq5

AGCCCAGCGGTACTGTCTCCTCCTGGTAAAATGAGGAGAGTGATGGGTCCTGAAGGATTC 120

ACT**ATG**GTGAGCAAGGGCGAGGAGCTGTTCACCGGGGTGGTGCCCATCCTGGTCGAGCTG 180 D2_50ntF

GACGGCGACGTAAACGGCCACAAGTTCAGCGTGTCCGGCGAGGGCGAGGGCGATGCCACC 240

TACGGCAAGCTGACCCTGAAGTTCATCTGCACCACCGGCAAGCTGCCCGTGCCCTGGCCC 300

ACCCTCGTGACCACCCTGACCTACGGCGTGCAGTGCTTCAGCCGCTACCCCGACCACATG 360

AAGCAGCACGACTTCTTCAAGTCCGCCATGCCCGAAGGCTACGTCCAGGAGCGCACCATC 420

TTCTTCAAGGACGACGGCAACTACAAGACCCGCGCCGAGGTGAAGTTCGAGGGCGACACC 480 GFP

CTGGTGAACCGCATCGAGCTGAAGGGCATCGACTTCAAGGAGGACGGCAACATCCTGGGG 540

CACAAGCTGGAGTACAACTACAACAGCCACAACGTCTATATCATGGCCGACAAGCAGAAG 600

AACGGCATCAAGGTGAACTTCAAGATCCGCCACAACATCGAGGACGGCAGCGTGCAGCTC 660

GCCGACCACTACCAGCAGAACACCCCCATCGGCGACGGCCCCGTGCTGCTGCCCGACAAC 720

CACTACCTGAGCACCCAGTCCGCCCTGAGCAAAGACCCCAACGAGAAGCGCGATCACATG 780

GTCCTGCTGGAGTTCGTGACCGCCGCCGGGATCACTCTCGGCATGGACGAGCTGTACAAG 840

**TAA**GAAGATGATGTTTCATTTCTTTTGGAAAATATCAATGGATATGCCCAGAAGATTTCA 900 D2_50ntR

AAATTAGATTTTTACCATTGATAATTCAATATTAGTTAACTATCAAAGAGAAAAAATTTA 960

AAGCCATAAATGTCCTTCTTAGTTCTTACTTTGTTTAATTTACCAAATATGTCATGTATC 1020

AACTATTGTTATAATTTCACTATATATCAACTATCTTACcatattaatataaatattatt 1080

cagatatttaatatgttagcacgttatattcagtataacatgttattataaagttaaatt 1140

gattatttcatattaatttgtatgatatggtaaatggtttaatacgatatataaaaattt 1200

atcgtgatactatactagttaacatattaatcaaatagttatatatatttagcacgatac 1260

tatactagttaacatgttaatcaaatagttatccacataaatcataaatattttatgcaa 1320

tccaatgataaatacgacagtgtcgtaaatttcggatgaatagtgtcttcaggcag 1376 D2seq3

**CDKD;2:GFPPIPL (74.79 kDa)**

MSKSGDNQPVDRYLRRQILGEGTYGVVYKATDTKTGKTVAVKKIRLGNQKEGVNFTALRE 60

IKLLKELNHPHIVELIDAFPHDGSLHLVFEYMQTDLEAVIRDRNIFLSPGDIKSYMLMTL 120

KGLAYCHKKWVLHRDMKPNNLLIGENGLLKLADFGLARLFGSPNRRFTHQVFATWYRAPE 180

LLFGSRQYGAGVDVWAAGCIFAELLLRRPFLPGSTEIDQLGKIFQAFGTPVPSQWSDMIY 240

LPDYMEFSYTPAPPLRTIFPMASDDALDLLAKMFIYDPRQRITIQQALDHRYFSSSPSPT 300

EPGKLQIPASKGDALEPKASEQNQHGNSPAVLSPPGKMRRVMGPEGFTMVSKGEELFTGV 360

VPILVELDGDVNGHKFSVSGEGEGDATYGKLTLKFICTTGKLPVPWPTLVTTLTYGVQCF 420 GFP

SRYPDHMKQHDFFKSAMPEGYVQERTIFFKDDGNYKTRAEVKFEGDTLVNRIELKGIDFK 480

EDGNILGHKLEYNYNSHNVYIMADKQKNGIKVNFKIRHNIEDGSVQLADHYQQNTPIGDG 540 PIPL:

PVLLPDNHYLSTQSALSKDPNEKRDHMVLLEFVTAAGITLGMDELYKVDMGHDDHHHGHD 600 CobW

CHDHHNEHEHEHEHEHHHSHDHTHDWSHPQFEKGGGSGGGSGGGWSHPQFEKGSYPYDVP 660 StrepII

DYA. 664 HA

**CDKD;2:GFP (66.12 kDa)**

MSKSGDNQPVDRYLRRQILGEGTYGVVYKATDTKTGKTVAVKKIRLGNQKEGVNFTALRE 60

IKLLKELNHPHIVELIDAFPHDGSLHLVFEYMQTDLEAVIRDRNIFLSPGDIKSYMLMTL 120

KGLAYCHKKWVLHRDMKPNNLLIGENGLLKLADFGLARLFGSPNRRFTHQVFATWYRAPE 180

LLFGSRQYGAGVDVWAAGCIFAELLLRRPFLPGSTEIDQLGKIFQAFGTPVPSQWSDMIY 240

LPDYMEFSYTPAPPLRTIFPMASDDALDLLAKMFIYDPRQRITIQQALDHRYFSSSPSPT 300

EPGKLQIPASKGDALEPKASEQNQHGNSPAVLSPPGKMRRVMGPEGFTMVSKGEELFTGV 360

VPILVELDGDVNGHKFSVSGEGEGDATYGKLTLKFICTTGKLPVPWPTLVTTLTYGVQCF 420 GFP

SRYPDHMKQHDFFKSAMPEGYVQERTIFFKDDGNYKTRAEVKFEGDTLVNRIELKGIDFK 480

EDGNILGHKLEYNYNSHNVYIMADKQKNGIKVNFKIRHNIEDGSVQLADHYQQNTPIGDG 540

PVLLPDNHYLSTQSALSKDPNEKRDHMVLLEFVTAAGITLGMDELYK. 588

**(e)**

***CDKD;3:GFPPIPL* insertion in the EcoRI-BamHI sites of pGAPKm and pGAPHyg vectors**

**EcoRI**

**GAATTC***gctaagattgatccatccactttccttgaaacgcttggtggtcccgagtctcct 60* D3Flank1-EcoRI

*ggacgaacatggatgcttatcttcaccgccgaggtaccattagatattcaattagccttt 120*

*acatgtttttataaatattgcagtcttgattgatccattcaaatcattgacttgggatct 180*

*tatataccataaatacagaagaaactgacgaaaggtcgttatttccctctaactgctgtt 240*

*cagagatttgatgctgcggtaagtctctaaactggttctagtactgctttagggtctgat 300*

*tggtaaaagtgttgaggcactttaaaatcctgaaagccgatattttggaagtaacaaaac 360*

*aattcttgtgcttgatttttcaaccagggaaaaagaatagagaatggggtgtatcttggt 420*

*ccatttggagcattaacattcgaaggaaggttttcatggaagaatcggatactagctttt 480* ***Flank1***

*gtcttcgaacagatccgcataaagattggaccattagatcctctagagttcagcttgggg 540*

*aagaaagacgctgtggaagagcctagtaataaagaccctttcttcatttggttctacatc 600*

*gatgaagaaatagccgttgctcgaggtagaagtggcggtacagctttctggtgtcgttgt 660*

*cgtcgcattgcttcctaatgactctcttctacttctcagaagctgcctctccttgtaaat 720*

*gatatttgaagatattgagcctatttgtaatgttcggaaaaaaggtctatttccccatgg 780*

*gaactatcgcatctggctagatgaagcccgatctttgacctcttgctatttacccatgaa 840*

*atgaaagttaaacgtctatttccccaccaattaaaagttcaagagcttaatgtccccgaa 900*

*ataaaacttcgagaacttaatgtccatgaactggattaagtcggtttactgtatgcgaaa 960*

*ccgatgacttaaccaactaaacctatttagaaccagaataaacctaatttaaaccaaaat 1020*

*tatgacccgtaacccgactctcgaac*ctaaacaaaaaaatccccaaattctctcccgacg 1080 D3FLank1_SalI

aaccctaatttaacctgacgaatcctcaaatccagtttttccaaaccctaaaactttgat 1140

cgatcgatgtcggg**ATG**TCGTTAATCCATCACGACCCCGATCTTGATGGTGATGTCGATC 1200 AT1G18050

CCGAAGCTGAAGACAGAGATAACTTTCACCTGGAAGAAATGCTAAACGAGTGGACCGACG 1260

AACCACCAATTCGCCACGACGTGTACCCGGAGAGCGATGGTGAAGGAGCCGACGACAGTT 1320

CCGACATACATGTGAGGCGTGGTGATGGGTTTTTGTACCAAAAGCAGAGTTTCTTCAGTG 1380

GAGTTGCGTTCAAAGAGGCGGTTATAGATTATGCTCTGAGAACTGGACATAATCCAACAT 1440

CTCTTTGGCACTCCAACCACTCTTCCTCGACCACGAggtcctgtagatcccgacgaactt 1500

gatgacagattgctcatctttcaactggaaataaaaaaattgggaatcgatttagggttc 1560

ttgagttttgagtttggggttttagattttggatttgggattcgacaatggatcgggttt 1620

taaaaacgaatccgggtttttttggtttggttttgaccgataatatggtttcgtctcggg 1680

tttacgtatcaaaccgaagtaaccacgattcggggacattaagctcttgaacttttaatt 1740

ggtggggaaatagacgtttaactttcatttcatgggtaaatagcaagaggtcaaagatcg 1800

ggcttcatctagccagatgcgatagttcccatggggaaatagaccgtttttccgtaatgt 1860

tctgatgttaaacagaatggttttataacataacagttcatgtatgtatgatttggttct 1920

taccggttaagtagataatgtatggtgaaaatggaaaacgacgtctctcacaacaaaaaa 1980

caactaaaacccaaaatctcgacccaaaaccgaatttaacttcaaattaagtaaatatgg 2040

cccgaagttataagcccgaataacagaggcccattataaaacctggagcacaagtttctc 2100

gtctataagtgctaggctacgcatagggctccgtggatttgttctaagaggatattctcc 2160

gttgatttgcaattgtcaatttccaaGCTTCAGAACTAGAGTTCGTTCGTTCACGACGTC 2220

ACTGTGGCTCTCGCGGCGTGGCTGTTACTTTCCGGTGggtgaggtacggtacttgttatc 2280

tctctccgaaaccctaagtagtccttggatatgtatctttgccttttcttatatttacgt 2340

ttaggaagataattcgttttcttggtagtttaccccttgtataaatatagtcatatagag 2400

ctcagggtcaagtgataatcaacacattcaatcgatctctttgcatctcttgtcatcaag 2460

ggttaactttatctattattttgtgtttctctttgtataGAAAAGAGATGTCGAGTGATT 2520

TGCAATCGAACTCCGGCAGATGTACCTCCGAGGACTAGATATTATGCGGATAGCTCAGCC 2580

CGTTTGGTTTTCATGGAGGGGCCTGAGATGGAGAGAAAGATGATGACAAGCTATGCGGGA 2640

AACCCAAAGTACTCCTTCTTCTGGAGCTCAGATCGTTATCATGCTTATTACCAGAAAAAG 2700

CTTGCTGGATACCGTGCGCAGAATTACATGAGGGACCTGAGATCAAACTTAGTGATTATG 2760

GGATACCTGATGTTCCATTGAGGGTGAGGGTAAGGGAACCACCGCTTCAGATTCCAAAAC 2820

CTCCTCCGGGCTTTGCTAGTGACCAGAAGCCGACAATGAAGTATCCGCCTCCACCACCTC 2880

GCAAGTATGCAGCTATACTCCCTGAATGGATTACTGGCAAAGAGCTTGAAACTATCAAGG 2940

AAGGGGATATGGACGAGAAACAGCAACGA**TAG**ctcttaagaaatcagctaaactctagca 3000

aaaacacgtggattgctaatatgctttataggttataggttaaatttgttgaggtataaa 3060

acagtcatttcaaaaaatcaaaaattcacgattaatttagctattgtttaaaagtttaga 3120

tattaaataatatttttctcttttataaatcgaaaatgtttggatattaaatgtataaaa 3180

aactagaaaagtaaataattgggcgtttctctttatttgttgtttccctcgattagccga 3240

ttgCGTCTGCAAACGCGCCAACGGCTACTTTTTCTCCGGAGCCGGAGCTCTCAAAGCTGA 3300

AACCCTAAATTTTCCCGGTATCTTCCTCATCGTCGATTTTACTCAATTTAGAATAAAAAT 3360

CGGAAAGCAATCGAATTCTAGCGACGTGAATTAAGAAGAAAGAGAGAGGCGCGATTATTG 3420

AGTGGGTTCTGAAAAACCCGAAA**ATG**CCGGAGCAGCCAAAGAAAGTTGCTGATAGGTATC 3480 **CDKD;3**

TCAAGCAAGAGGTTCTTGGGCAAGGTACCTATGGAGTCGTCTTCAAAGCCACTGATACTA 3540 AT1G18040

Aggtcatatctttgctgatttttgtttgcaaaattagggtttaggatctgtcggcaattc 3600

ctaatttctccttcttttttggttccggattttgattgaagtttttgttggatcaagggg 3660

aagtaattgtggggtgtaattttgcaGACGGAACAAACTGTAGCAATTAAGAAGATAAGG 3720

CTTGGTAAACAAAGAGAAGGTGTAAATATTACAGCTCTCAGAGAAATCAAAATGCTCAAA 3780

GAGCTAAAGCATCCTCATATTATTCTGCTCATTGATGCATTTCCTCACAAGGAAAACTTG 3840

CATCTTGTCTTTGAGTTCATGGAGACTGACCTCGAAGCAGTTATTCGCGATTCTAACATT 3900

TTCCTCTCACCCGCCGACATTAAATCCTACCTCCTAATGACATTTAAAGGACTTGCTTAT 3960

TGCCACGATAAATGGGTTTTGCACAGgtacatttgtttatccttaacaacttgcttattg 4020

tctctgtaatgttgcttggtttgggcactgattatgtgtggcttgagttcagGGATATGA 4080

AGCCAAATAACTTGTTAATAGGAGTTGATGGACAGCTTAAGCTTGCAGATTTTGGCTTAG 4140

CACGTATATTTGGTAGTCCAAATCGTAAGTTTACCCACCAGGTatgccttttgccgcatt 4200

gccattttatctattgctgtaatataaccaaattgatcgactaactcatatgaaatattg 4260

ataagaaagttttaagtagttcttatcagtagttatgtctagggatgaggaagattagat 4320

gtctcatgtcttttcatttctctagttgtatactaaatgtaacatgtattaaacagtctc 4380

aactcatattaaatgatggtgcagtagctatctcatatctgtactaaaagttttgctgtt 4440

aaccggatttttgacctgagctatttctgccttctttgtttccctttgaacacccaaaat 4500

tacaatagaatccatgtgagctctgtttccaaatgcgcaatgcaacttattttctcttgt 4560

ttctagttgttatattatcttcatatcttgatatatctgataatgcacaggtGTTTGCTA 4620

GATGGTACAGAGCACCAGAACTTTTGTTTGGTGCAAAACAATATGGTGCTGCAGTTGATG 4680

TTTGGGCCGTTGCCTGCATATTCGCCGAACTTCTACTACGCAGACCATTTCTTCAGgtta 4740

aataatcttactcaaatctaatggctaccattaaaactttagtccaattgtcatctcttt 4800

ggatgttatactcactctcagactacataattacatattcattatcaattttctcggtat 4860

gtttacttattctatttttgccgttgcagGGAAACAGTGATATTGATCAATTAAGCAAAA 4920

TATTTGCTGCTTTTGGGACACCAAAAGCAGATCAGTGGCCGGATTTAACAAAGCTTCCAG 4980

ATTATGTAGAGTATCAATTTGTCCCTGCACCGTCTCTACGTTCTTTATTCCCAGCAGTTA 5040

GTGATGATGCTCTCGACTTGTTGTCCAAAATGTTTACCTATGACCCAAAGGCTAGAATTT 5100

CCATTAAGCAGGCTCTAGAACACAGGTgcctaatgttcaccatctgtttataggcaaagt 5160

atatctagcgttctgctctcacacctttgttttcattatttcaggtACTTCACTTCTGCA 5220

CCTGCTCCTACTGACCCGGCTAAGCTCCCAAAGCCCGTTCCTAAGCAGGATGGTAAATCA 5280

TCTTACGGTAAACATGAGGCCATTACAGTGCAATCACCACCACGTAAGCTTAGAAGAGTG 5340

ATGCCTGAGCGTGGGAGGGTTGATAGTTTGAAGTCTCATGTTGACAAGGATCAACAAGCA 5400

CCCATGTCATTAGATTTTACCATCCTCGCTGAGCGACCTCCAAACAGGCCAACAATCACC 5460 D3seq5

Aggtaaaaaacaagacagtttctccctcactgcatattaatctcatttgtcattacagcc 5520

tcaagattgattgtgttttaatgacttcttcttgttcaatttgtaGTGCTGATAGATCTC 5580 D3_50ntF

ATCTGAAGAGGAAACTCGATCTTGAGTTCCAG**ATG**GTGAGCAAGGGCGAGGAGCTGTTCA 5640

CCGGGGTGGTGCCCATCCTGGTCGAGCTGGACGGCGACGTAAACGGCCACAAGTTCAGCG 5700

TGTCCGGCGAGGGCGAGGGCGATGCCACCTACGGCAAGCTGACCCTGAAGTTCATCTGCA 5760

CCACCGGCAAGCTGCCCGTGCCCTGGCCCACCCTCGTGACCACCCTGACCTACGGCGTGC 5820

AGTGCTTCAGCCGCTACCCCGACCACATGAAGCAGCACGACTTCTTCAAGTCCGCCATGC 5880

CCGAAGGCTACGTCCAGGAGCGCACCATCTTCTTCAAGGACGACGGCAACTACAAGACCC 5940 GFP

GCGCCGAGGTGAAGTTCGAGGGCGACACCCTGGTGAACCGCATCGAGCTGAAGGGCATCG 6000

ACTTCAAGGAGGACGGCAACATCCTGGGGCACAAGCTGGAGTACAACTACAACAGCCACA 6060

ACGTCTATATCATGGCCGACAAGCAGAAGAACGGCATCAAGGTGAACTTCAAGATCCGCC 6120

ACAACATCGAGGACGGCAGCGTGCAGCTCGCCGACCACTACCAGCAGAACACCCCCATCG 6180

GCGACGGCCCCGTGCTGCTGCCCGACAACCACTACCTGAGCACCCAGTCCGCCCTGAGCA 6240

AAGACCCCAACGAGAAGCGCGATCACATGGTCCTGCTGGAGTTCGTGACCGCCGCCGGGA 6300

TCACTCTCGGCATGGACGAGCTGTACAAGGTCGAC**ATG**GGTCATGATGATCATCACCATG 6360 PIPL:

GTCATGACTGCCATGATCACCACAATGAGCATGAGCATGAGCATGAACACGAGCATCACC 6420 CobW

ATTCTCATGATCACACCCATGACTGGTCTCATCCTCAGTTCGAAAAAGGAGGTGGATCTG 6480 StrepII

GTGGAGGTTCTGGAGGTGGATGGTCTCACCCACAATTTGAGAAGGGATCTTATCCATACG 6540 **HA**

ATGTTCCAGATTATGCT**TGA**GACTGTTGGTTATGCACTCTTCTCTTAGCTTCTCCATTTT 6600 D3_50ntR

CATGTTCTGGTGAAACCACACCATTGTTAAGTTGTCAATTTACTGCTGCTTGTGTAATCT 6660 D3seq3

GGCGATTTATTCTTTTCCATATTTAGCTTGATTACAAACCAGAGGGACCTTAGATTCACA 6720

ATGATAATATTTTGTTGTCCCATAGGCTTTTTATTCATTGTAAACTGATCATACTGTACT 6780

ATGAAGTCTATGATTATAAAGCTTTCTACTTTTTGCTTCTAGAATTCTTTGCGGTACAAA 6840 natural antisense

GAGAGCTCTGTTTAATCTTCAAACTCATTTTTCTTTCGATGGTGGCCCCAGGAATTTAAT 6900 natsi with **AT1G18030**

TGATCCATTGACAGCTCAAAAAGATTTTACCAAATACCAACAACAGGTTTATAAAACACA 6960

TACGAGCCAGAAAAACAGATGCCCACATGTCAGTCAGGCTCTTCTTTAG**TCA**TACCCGCT 7020

TGAAGACTATCACGATTGCTGTGCAGTTGTCTTTGCAACGACGTTCTTTCACAGCTTCCT 7080

TCACAAGACGGCGACTTACCGTGCTTACATGCAAGCCCTCCTacaaaatcatagttctta 7140

gcctcttagtttctcacatttaatttcccttgacactctttacttgtgtgcacaaggttc 7200

taacaagccattttgctcacatggctaccgaaaaacatctgttttttgagcttctttcta 7260

tgtagttaaagagagtgtgaaatggaggacaaagtgattttgacaactgtaggtatttac 7320

ctTCAAAAGTTTCTGGACAAATCCAACAGCATCACTTGGTCCAAACACctgaaaacggtt 7380

gcattgtgtatgagcagcaaactttgatatataaagacaaaatgaaatgttcttatagag 7440

cactgcaaaaccacaaacgaagagcttacttacTTCCCACAATCCATCGCAACCAAGAAT 7500

CATGAAGTTTTCTCTCTCAGTTAATTCAAAAGCATGAATGTCTGGAGTTGCACTGACACC 7560

AAACTagattcaataacttgcgttagatgcgaataaaccataagattttaggataagtta 7620

cagattctaggcatttgaagttgcaaaatgatatggagcaaggttcaagaacatgtcgat 7680

agaatattgcacgcgagagctaaaatgtcacagataatgggctcaatataaagtgggaag 7740

ccaccggatcattacctTCTTAAAATGACGATCACCAAAAGCCCTAGAAACCTCAAGACG 7800

CCCTTGTAACCGTCCATTTGAGCTTATAACACCACCTGACTgtgaaactcatagcaagtt 7860

atcaacagagcatggtgtgctcacatttacttggaagtctctgtctggctcatcaaaaac 7920

taacctTTTGAATGCGAGAGCGCTCCTGTGGATAAATTGCTTTGTGCTCTCTCGTCAAAA 7980

CAATTGCTTTGAGTGGATTACCTGCTTCTGTATGATTCCCCAATTCGTTGGTAGTAGAGG 8040

ATCGTGCCAAAACAGCCTTAGCATCACCGATATTGGCAACAAAAACCTataaagcatgag 8100

agtttatgggtgtgaatactggagaaaaggtcgagttattttatgaaatttatctagaga 8160

gtaaaaatattgaattccagagaccccatcacagtcatacctTTTGATCAAGTATCCAGA 8220

CACAGACTGCTGTGGCTCCATCTTGCCATCCTCCTGtacaagacacaggtcagagagaag 8280

cataaatatgaatgaactaagagattctatggagaagatataggaaagcaatcaacagcc 8340

gagataaaagctcagacattcgaaacatagatgcagaatatagccaagacaaacacacac 8400

atacacaaactacctgAAACACTTTTTTGCAGGAGCAACTCATCAGTTTTCCGGAAACCT 8460

gaaagtaaaattgctttaacagttgagaacctgtggatctgaaaagccataatgaaatga 8520

ctaaaaagttttaagagacttatgaagtaaacttatatatgtacctTCAAGAATGGCCTT 8580

TTTAGCAACTTTGACATCCAGCtgaaacagcaatttgacagagaagaaggtgaagtgaga 8640

ccgagtgaatgacatgaaaacagttgtaaagacataattaatatgaaccaagtaagacag 8700

accAACTCACGTGGTAACCCAGCTGAAAGAACGTTAAGGTGAAGATGCTTCTTAGCAAAC 8760

TCTGCAGCTAAACGACCTCCATGCCCATCATAAATTGCAAAATGCGCACACCTacatacc 8820

aatacagaaaagcctacctagctttataaagaaacctaaaaaaatatcattttgcacaat 8880

aagattcgtatcttcctaattcttctaagtcccacaaacagaatgtgtccaattactaaa 8940

cttgattcaactaagagcttgtatttgttacctTAGTGTCCCTGGGAAATCCAAAGAAGC 9000

GTCAGGCAAAACCACCCAAACATCTTCCATAGTATGTCTAGCTCCTTTGTCTTCAGCAAC 9060

ATCAGCTTCGACCAGAAACTCCTTCTTTTCTTCACTTACAAAGCTAGGCTTATCTTCTTC 9120

CGCCTCTCTATTACCAACGGCGGCTACAGCTTCTCCTCCGCCGGAAACCT*CCTCTGACTT 9180* D3Flank2_SalI

*CTTTGCCTTCTTCACCGGTGAGACCAAATCCTCGCTGGAAAACGAATCAGCCGCGTGCTT 9240*

*GTTATTTGGTTTCTCCATTGCAGTCAAATCAGATTCCTTCTCCAA****CAT****TCGTTGAGAATC 9300* AT1G18030

*TTACGCTTAATCTTCTCGGTCTGTTTCCCGGCAATAATCTTGTCGggtcaccgtcgcaaa 9360*

*gaatgagatagtaaagtaaacacaaacaagttgcaagaatatataatttagtacccaaag 9420*

*tttctgtatattttattttaggccccacacttttaaatttacctgaagtaccttgaattt 9480*

*taattcgtatacggtattattacactacacataaatgattcggtttcttaaccatgatat 9540*

*tcgtatatttgtgcaagtttatcaatcggcgtaagttttgcgaaagcatttgtgactttg 9600*

*tataaccaattttagttttgttgaagcatttgtgtaagcaattttggaacttatagtttg 9660*

*ttgcggacgatgatgattttatataggtggtacgttgtagtggaaatgatgaaacttgga 9720*

*gagttggacactgccgaagcttgtttgaggcaagctttttgccttatgtgattgaaaaat 9780* ***Flank 2***

*atgttttgctaataatgcatctgaataaaccccttaagcagttgttgtatctgtggtctc 9840*

*gaggaaagggtagtctgtatagcccactacaggatctgaagtgtagaacgttgatctatc 9900*

*gtacctattcaacggctcattgagttcgaatctcctcgtcagatccggattcgccaagaa 9960*

*cggccgtccataagccacaagatcggttcttccctcttccaccgccttgttcccgtcttc 10020*

*tctagaatatcctcctgctactatgaacgtacctttgaaggcttttcgcatgggcgtaag 10080*

*cgattccgtgcattcgaagattccttcaagggttttcattctaggttcaaccatgtgacc 10140*

*gtagacgactccatgccatgcttgttcatagcttgcaccaggtagagccctaatgcttct 10200*

*ggattcgagtctcctgactccatgtaatctgcaaatggcgagagtctgattccaacacga 10260*

*tctgaaccgatctcgttcaccactgcttcgattacttcaagagcaaatctacagcggttc 10320*

*tctaatgacccaccatattggtcacttctgtcattcactttgtctttcaggaactgatcg 10380*

*atcaggtaaccatgtgcgccgtgaacctccactccatcgaagcc***GGATCC** 10430 D3Flank2_BamHI

**BamHI**

***CDKD;3*:*KmR-araC-ccdB* cassette insertion between the gene specific primers F1seq5 and F1seq3:**

ATCCTCGCTGAGCGACCTCCAAACAGGCCAACAATCACCAggtaaaaaacaagacagttt 60 D3seq5

ctccctcactgcatattaatctcatttgtcattacagcctcaagattgattgtgttttaa 120

tgacttcttcttgttcaatttgtaGTGCTGATAGATCTCATCTGAAGAGGAAACTCGATC 180

TTGAGTTCCAGgccgccatgacatgggccacgttgtgtctcaaaatctctgatgttacat 240 D3_50ntF

tgcacaagataaaaatatatcatcatgaacaataaaactgtctgcttacataaacagtaa 300

tacaaggggtgtt**ATG**AGCCATATTCAACGGGAAACGTCTTGCTCGAGGCCGCGATTAAA 360

TTCCAACATGGATGCTGATTTATATGGGTATAAATGGGCTCGCGATAATGTCGGGCAATC 420

AGGTGCGACAATCTATCGATTGTATGGGAAGCCCGATGCGCCAGAGTTGTTTCTGAAACA 480

TGGCAAAGGTAGCGTTGCCAATGATGTTACAGATGAGATGGTCAGACTAAACTGGCTGAC 540

GGAATTTATGCCTCTTCCGACCATCAAGCATTTTATCCGTACTCCTGATGATGCATGGTT 600

ACTCACCACTGCGATCCCCGGGAAAACAGCATTCCAGGTATTAGAAGAATATCCTGATTC 660 **KmR**

AGGTGAAAATATTGTTGATGCGCTGGCAGTGTTCCTGCGCCGGTTGCATTCGATTCCTGT 720

TTGTAATTGTCCTTTTAACAGCGATCGCGTATTTCGTCTCGCTCAGGCGCAATCACGAAT 780

GAATAACGGTTTGGTTGATGCGAGTGATTTTGATGACGAGCGTAATGGCTGGCCTGTTGA 840

ACAAGTCTGGAAAGAAATGCATAAGCTTTTGCCATTCTCACCGGATTCAGTCGTCACTCA 900

TGGTGATTTCTCACTTGATAACCTTATTTTTGACGAGGGGAAATTAATAGGTTGTATTGA 960

TGTTGGACGAGTCGGAATCGCAGACCGATACCAGGATCTTGCCATCCTATGGAACTGCCT 1020

CGGTGAGTTTTCTCCTTCATTACAGAAACGGCTTTTTCAAAAATATGGTATTGATAATCC 1080

TGATATGAATAAATTGCAGTTTCATTTGATGCTCGATGAGTTTTTC**TAA**tcagaattggt 1140

taattggttgtaacactggcagagcattacgctgacttgacgggacgg**CTATATTACCCT** 1200 **I-SceI**

**GTTATCCCTAGCGTAACTC**catggcggccgcgggaattcgatatcactagagccgtcaat 1260

tgtctgattcgttaccaa**TTA**TGACAACTTGACGGCTACATCATTCACTTTTTCTTCACA 1320

ACCGGCACGAAACTCGCTCGGGCTGGCCCCGGTGCATTTTTTAAATACTCGCGAGAAATA 1380

GAGTTGATCGTCAAAACCAACATTGCGACCGACGGTGGCGATAGGCATCCGGGTAGTGCT 1440

CAAAAGCAGCTTCGCCTGACTAATGCGTTGGTCCTCGCGCCAGCTTAAGACGCTAATCCC 1500

TAACTGCTGGCGGAAAAGATGTGACAGACGCGACGGCGACAAGCAAACATGCTGTGCGAC 1560

GCTGGCGATATCAAAATTGCTGTCTGCCAGGTGATCGCTGATGTACTGACAAGCCTCGCG 1620

TACCCGATTATCCATCGGTGGATGGAGCGACTCGTTAATCGCTTCCATGCGCCGCAGTAA 1680 **araC**

CAATTGCTCAAGCAGATTTATCGCCAGCAGCTCCGAATAGCGCCCTTCCCCTTGCCCGGC 1740

GTTAATGATTTGCCCAAACAGGTCGCTGAAATGCGGCTGGTGCGCTTCATCCGGGCGAAA 1800

GAAACCCGTATTGGCAAATATTGACGGCCAGTTAAGCCATTCATGCCAGTAGGCGCGCGG 1860

ACGAAAGTAAACCCACTGGTGATACCATTCGCGAGCCTCCGGATGACGACCGTAGTGATG 1920

AATCTCTCCTGGCGGGAACAGCAAAATATCACCCGGTCGGCAGACAAATTCTCGTCCCTG 1980

ATTTTTCACCACCCCCTGACCGCGAATGGTGAGATTGAGAATATAACCTTTCATTCCCAG 2040

CGGTCGGTCGATAAAAAAATCGAGATAACCGTTGGCCTCAATCGGCGTTAAACCCGCCAC 2100

CAGATGGGCGTTAAACGAGTATCCCGGCAGCAGGGGATCATTTTGCGCTTCAGC**CAT**act 2160

tttcatactcccaccattcagagaagaaaccaattgtccatattgcatcagacattgccg 2220

tcactgcgtcttttactggctcttctcgctaacccaaccggtaaccccgcttattaaaag 2280

cattctgtaacaaagcgggaccaaagccatgacaaaaacgcgtaacaaaagtgtctataa 2340

tcacggcagaaaagtccacattgattatttgcacggcgtcacactttgctatgccatagc 2400

atttttatccataagattagcggatcctacctgacgctttttatcgcaactctctactgt 2460

ttctccatacccgtttttttggatggagtgaaacg**ATG**CAGTTTAAGGTTTACACCTATA 2520

AAAGAGAGAGCCGTTATCGTCTGTTTGTGGATGTACAGAGTGATccdBATTATTGACACG 2580

CCCGGGCGACGGATGGTGATCCCCCTGGCCAGTGCACGTCTGCTGTCAGATAAAGTCTCC 2640 **ccdB**

CGTGAACTTTACCCGGTGGTGCATATCGGGGATGAAAGCTGGCGCATGATGACCACCGAT 2700

ATGGCCAGTGTGCCGGTCTCCGTTATCGGGGAAGAAGTGGCTGATCTCAGCCACCGCGAA 2760

AATGACATCAAAAACGCCATTAACCTGATGTTCTGGGGAATA**TAA**gagctcGACTGTTGG 2820 D3_50ntR

TTATGCACTCTTCTCTTAGCTTCTCCATTTTCATGTTCTGGTGAAACCACACCATTGTTA 2880

AGTTGTCAATTTACTGCTGCTTGTGTAATCTGGCGATTTATTC 2923 D3seq3

***CDKD;3:GFP* construct between the gene specific primers D3seq5 and D3seq3:**

ATCCTCGCTGAGCGACCTCCAAACAGGCCAACAATCACCAggtaaaaaacaagacagttt 60 D3seq5

ctccctcactgcatattaatctcatttgtcattacagcctcaagattgattgtgttttaa 120

tgacttcttcttgttcaatttgtaGTGCTGATAGATCTCATCTGAAGAGGAAACTCGATC 180 D3_50ntF

TTGAGTTCCAG**ATG**GTGAGCAAGGGCGAGGAGCTGTTCACCGGGGTGGTGCCCATCCTGG 240

TCGAGCTGGACGGCGACGTAAACGGCCACAAGTTCAGCGTGTCCGGCGAGGGCGAGGGCG 300

ATGCCACCTACGGCAAGCTGACCCTGAAGTTCATCTGCACCACCGGCAAGCTGCCCGTGC 360

CCTGGCCCACCCTCGTGACCACCCTGACCTACGGCGTGCAGTGCTTCAGCCGCTACCCCG 420

ACCACATGAAGCAGCACGACTTCTTCAAGTCCGCCATGCCCGAAGGCTACGTCCAGGAGC 480

GCACCATCTTCTTCAAGGACGACGGCAACTACAAGACCCGCGCCGAGGTGAAGTTCGAGG 540 GFP

GCGACACCCTGGTGAACCGCATCGAGCTGAAGGGCATCGACTTCAAGGAGGACGGCAACA 600

TCCTGGGGCACAAGCTGGAGTACAACTACAACAGCCACAACGTCTATATCATGGCCGACA 660

AGCAGAAGAACGGCATCAAGGTGAACTTCAAGATCCGCCACAACATCGAGGACGGCAGCG 720

TGCAGCTCGCCGACCACTACCAGCAGAACACCCCCATCGGCGACGGCCCCGTGCTGCTGC 780

CCGACAACCACTACCTGAGCACCCAGTCCGCCCTGAGCAAAGACCCCAACGAGAAGCGCG 840

ATCACATGGTCCTGCTGGAGTTCGTGACCGCCGCCGGGATCACTCTCGGCATGGACGAGC 900

TGTACAAG**TAA**GACTGTTGGTTATGCACTCTTCTCTTAGCTTCTCCATTTTCATGTTCTG 960 D3_50ntR

GTGAAACCACACCATTGTTAAGTTGTCAATTTACTGCTGCTTGTGTAATCTGGCGATTTA 1020

TTC 1023 D3seq3

**CDKD;3-GFPPIPL (80.2 kDa)**

MPEQPKKVADRYLKQEVLGQGTYGVVFKATDTKTEQTVAIKKIRLGKQREGVNITALREI 60

KMLKELKHPHIILLIDAFPHKENLHLVFEFMETDLEAVIRDSNIFLSPADIKSYLLMTFK 120

GLAYCHDKWVLHRDMKPNNLLIGVDGQLKLADFGLARIFGSPNRKFTHQVFARWYRAPEL 180

LFGAKQYGAAVDVWAVACIFAELLLRRPFLQGNSDIDQLSKIFAAFGTPKADQWPDLTKL 240

PDYVEYQFVPAPSLRSLFPAVSDDALDLLSKMFTYDPKARISIKQALEHRYFTSAPAPTD 300

PAKLPKPVPKQDGKSSYGKHEAITVQSPPRKLRRVMPERGRVDSLKSHVDKDQQAPMSLD 360

FTILAERPPNRPTITSSADRSHLKRKLDLEFQMVSKGEELFTGVVPILVELDGDVNGHKF 420

SVSGEGEGDATYGKLTLKFICTTGKLPVPWPTLVTTLTYGVQCFSRYPDHMKQHDFFKSA 480 GFP

MPEGYVQERTIFFKDDGNYKTRAEVKFEGDTLVNRIELKGIDFKEDGNILGHKLEYNYNS 540 PIPL:

HNVYIMADKQKNGIKVNFKIRHNIEDGSVQLADHYQQNTPIGDGPVLLPDNHYLSTQSAL 600 CobW

SKDPNEKRDHMVLLEFVTAAGITLGMDELYKVDMGHDDHHHGHDCHDHHNEHEHEHEHEH 660 StrepII

HHSHDHTHDWSHPQFEKGGGSGGGSGGGWSHPQFEKGSYPYDVPDYA. 708 HA

**CDKD;3-GFPPIPL (71.54 kDa)**

MPEQPKKVADRYLKQEVLGQGTYGVVFKATDTKTEQTVAIKKIRLGKQREGVNITALREI 60

KMLKELKHPHIILLIDAFPHKENLHLVFEFMETDLEAVIRDSNIFLSPADIKSYLLMTFK 120

GLAYCHDKWVLHRDMKPNNLLIGVDGQLKLADFGLARIFGSPNRKFTHQVFARWYRAPEL 180

LFGAKQYGAAVDVWAVACIFAELLLRRPFLQGNSDIDQLSKIFAAFGTPKADQWPDLTKL 240

PDYVEYQFVPAPSLRSLFPAVSDDALDLLSKMFTYDPKARISIKQALEHRYFTSAPAPTD 300

PAKLPKPVPKQDGKSSYGKHEAITVQSPPRKLRRVMPERGRVDSLKSHVDKDQQAPMSLD 360

FTILAERPPNRPTITSSADRSHLKRKLDLEFQMVSKGEELFTGVVPILVELDGDVNGHKF 420

SVSGEGEGDATYGKLTLKFICTTGKLPVPWPTLVTTLTYGVQCFSRYPDHMKQHDFFKSA 480 GFP

MPEGYVQERTIFFKDDGNYKTRAEVKFEGDTLVNRIELKGIDFKEDGNILGHKLEYNYNS 540

HNVYIMADKQKNGIKVNFKIRHNIEDGSVQLADHYQQNTPIGDGPVLLPDNHYLSTQSAL 600

SKDPNEKRDHMVLLEFVTAAGITLGMDELYK. 632

**(f)**

***CYCH:mCherry* insertion in the SpeI-BamHI sites of pGAPBRKm and pGAPBRHyg vectors**

**SpeI**

**ACTAGT**CCCGTCCTTTATCAGTCGGGAATAGAGTGATTGCCATCCATCCTAAAACACGGG 60 CYCH_Flank1

AGATTCGTGATGGCAAGATTCTTACTGTGGATCATAACAAGTGCAACGTTCTCTTTGATG 120 (AT5G27610)

AATTGGGTGTCGAGTTAGTTATGgtaaatcacaaactatatcaattccgttgatagtttg 180

tccattttttgtatatctcagatgatctaataaatccttttttctgacagGACATTGACT 240

GCATGCCTTTAAATCCATTGGAATACATGCCAGAGGGTCTAAGGAGGCAAATTGATAAGT 300

GCTTGGCTATATGCAAAGAAGCAAGGCTTAACAGACACCCAAGCTCTGATGCATCTGTTC 360

TGTTCTCTCCTTCTGTGCTTGAAAATGTCAACTTTTCCATGAATCCTCCTCCTGCGAAAC 420

AGgtaaacaaagattgacgtttgactcgtgtgtcaaagctttgatcttcgtctttctttc 480

catacattttttaatctctgtggataattccataattctctgcagGATGATATCAGAGAG 540

CCAGTTTTGTATGGTAAAGTAATAGCAACCAACACTACTGATCAATCTATCGTAATCAAT 600

AGCAAAGTAACAGGAACAGAAATTCAACGGACTCTGGCACTGCAGCATACTTCCGATGCG 660

CAGgtaactaaaaagtctatgcctgtggaaactttgtctttagattgctcatgttcgaat 720

aagtatagacttattttgtgtactctttttgctaatagtaacagctacagaaattctttt 780

ctcatgattctgctttccagGAAATGGAGCCAGAAATGATTGAAATTGTCATTGAATCAA 840

AGTCAATAGCGCAAGCAATGGTGGATGCAGCTATAAAGgtatttataatatatctagaga 900

cttcagagtcttctaaatacagagaagccaaattcatacaactctaacatcttttgttta 960

aatcgttgtcaaccgttttagGCTGCATCGTCGGGGAAGAACAATGAAGACTCAGAGAAT 1020

ATGGTTCACCAAGCTTTAAGCTCCATTGGCGAACATCAGCCATTAGACAACTCTATAGTG 1080

CCTGGTATCAAGCATCAAGAGTATACCAATGGCAGCTTGGATCATCATTCCTTAAACACA 1140

GCAGAGCCGATGAGTAACGGTTTCATCTCACAGGAAGGATCAGGAAAAAACAAAACACCA 1200

ATGCCTTCAGAGCTTATCACCTCTTGTGTTGCATCTTGGCTCATGATGCAggtaaaggaa 1260

ataattttgtcttggactaacgaaagtgtaatctaaatcaagaataccaaagttttcact 1320

tgatgcggttttgattagaactctttgtgtgttatttacctgcaGATGATCTCAAAGAAG 1380

CAGTACCCACCAGCAGATGTGGCTCAGCTAATGGATACAGTAGTGAATGACTTGCAGCCA 1440

CGGTGTCCACAGAATATGCCGATATACAGAGAAATTCAAACTTGTATGGGATTGATCAAG 1500

ACTCAAATAATGGCTCTTGTAAGAACGTCA**TGA**AACATTTGTGTCCTTTGTAATTAACCT 1560

GACATCGAAGATAACACTTGGAGATCAGATTTAATTGTTTGGATTGTAGAGGTTAGTTTA 1620

GTTTATAGTTAGTTAATAATTTGAACAGCTCCAAAGGCTGTTGATAATTAGTTACCATTG 1680

ATTCTGTAACTTTTTTTTGTCATCATTCATTGTAACTTTGGTTACCTTTTactttggagc 1740

tgttttttttttctttctttctcagtgattattttcttgggtcattttggatcctaatgg 1800

ctgaaataatttattcccaccttaatacaaaatctcaaatatatactattccttaactaa 1860

aattctagaacaatgccaaatggatatgtttgaaatacctttaataacaactaggattta 1920

acctgtggtgtaccacggacaaatgttttattcatatataataatattaaattttacggt 1980

ttttaatttagtatttaagttttataattttatattaatgttataactaaaacaaatggt 2040

gatgataaaataataaaataaatatttaacatgtgctatatcaatccaaactcgtcccac 2100

tatatttcatatcaatccaaacccgtcccgctatatttctcatctcatgatattttttta 2160

taagtttaaataataataatgttttataaagttaaaatatatctataatatttaaaaata 2220

ataattattttttacttaagttggaaagatttttggaaacgtttgttgagcgcaatataa 2280

agtatttaaattttcaaatctgaataattaataccaattatatatcttgaaagatctttg 2340

gcaactattgttgaatataatatcttgtatttaattatcgacaggaaatatttaatgtca 2400

atggcatgacattgtaaatacaagtacaaaatttatttgctaaaatggctgcaaaaatgt 2460

atatgtagatttacttctaattttaatgtttcaaaggacaaaactaggcatgaccataaa 2520

atccgaagcccgaaaaacctagttcgctttggttcgagtggatctaaagtgtttattttg 2580

gggttttggtttggttcgtgtaggatctgaaacctgaacagatacctgaaatatcgaaac 2640

tcaaacggatacatgtaactcgaaataatacgtatacataatttgtcggtaaattcaggt 2700

tcaatatcaggtttcaattaaatttgagaaccagcccgaacccgaatagacctcaaccga 2760

tattttcagttcctaaatgtattttcaatctctttaccaaagtaacccaaaaacccaatc 2820

taaacagacccgaaaatccgaatgtccagacctaactaaaatcgtgagtcaaactcaaaa 2880

aatgattgattaacactatattatacttttacttagattctataaattggacacatgtat 2940

gggtgttattaagggttggaaacataaatttaagccatgaccttatttttggttggaaaa 3000

ttcttgttcttttttttgttagtaaagggctgttaataagggttttaatgggttagcgaa 3060

cgacaccgtttataataaatatgtttgctaaacgacatcgtttgaccttagggtttattg 3120

tttaacatgaaagaacgtcgtttcaccaacgtttcttcttcctcgcaagtctcaaagcca 3180

ggaacgatctcgccgtatgagcaacttccgacattaacactgtattgctctgccgtactc 3240

tcctctttttcccttcatactcgtcttcacatctgaattccgacaagatattgcgtctgc 3300

tctcctctcttccctcattctcgtcttctcattaccaaaaacatcttcttctcgtagcta 3360

actcaatccaattccaatctccattcaaatcgagcaccagataattttttctcctaaatt 3420

ctaatataagccctaaaattcaaaattgaaatatctatagaatctccttccgattcagac 3480

ggataatcgccatcaacagcgtgtgaattcgatcaaaaatctgaactttccacaaagaaa 3540

ttcgaaagctcagatttttcgatttcgaaaattttgaaaactcagtta**ATG**GCGGATTTT 3600 **CYCH**

CAGACATCAACACAACGGGCCAAGTGGATTTTCACTCCCCAGAAACTGgtaatccccttc 3660 At5g27620

tccttctgatcactacaatgcggctttataattatattttgttcatctttggattttctt 3720

tgctttgattgcttcatagagagtcggattaatcgagcatttggaactctttgttactta 3780

gttatgtgtgttgttgttgttattatcttacttgttgtgtagagatattggtattgcaac 3840

taagctgtaatgttgtaattgtagGCAGAGAGATATAAAGCTGCTAACCAGAGGGCAGTG 3900

CAAATGCTGGAGAAggtttgttagaataattgttgatatggagctttagtcctgtattca 3960

tgtttagattttaacttattgttattcataGTGTGGAACAACTCAAGTTGAAGTAGATGC 4020

TAGTGGATCACTAACATATCCTAAAGATAAAGTTGGTTCAGGAGATCAAggtaaaaattt 4080

tggttccttcaatatctacttcatacgtatcatagttttgtttgtcgaagtttaaataat 4140

gttactttttgctttgttactctcaGCTGATAAGAAGCTTAAGCCTTTGAGTGCTGATGA 4200

AGAAAGGTTCATGAGAGCATTTTATGAGGCAAAGGTCCAAGAAGTGTGCAGTGCCTTTGC 4260

ATTTCCTCACAAGATTCAGgtgcatttctactcgtcgagatagaagtttatgatgaagtt 4320

aaaaataaaatacctgatttttgtttgctctctgaatttatgtttcttccttctaatatt 4380

ttgcagGCAACAGCCCTCCAATACTTTAAGAGATTTTATCTGCAATGGTCTGTTATGCAA 4440

CATCATCCAAAAGAGATAATGTatagttcttttaaatcctcaaaatggtcaaaagctttg 4500

cttttttcaagagaatagttttgaaattgtttaccaaatggagctaagcattttgtattt 4560

ctgagctctgagaggctttttaagtcagtcataaatggtccattgttgtaatttggacta 4620

attgttacctttcattgaacaggtTAACCTGTGTGTATGCAGCTTGTAAAATAGAGGAGA 4680

ATCATGTATCTGCTGAGGAAATTGGGAAAGGGATTAACCAAGATCACCGAATAATTCTCA 4740

AGTACGAGATGGCTGTTCTTCAggtatgatatgagtcaattaatttgtaccaacttccct 4800

atgtttatttttccctggtgttcttttctctctctattcttttgttcatattatattaac 4860

tctatgtgcaGAGTTTGGAATTTGATCTGATTGTTTATGCACCGTATCGTGCAATCGAAG 4920

GTTTTGTCAACAACATGGAGgtaaatcagtcctgtctaaatttcttagtaggttttctta 4980

tttcaactggcttttctctgtttacatcttatagtgatgagactctttttcagtttattg 5040

gcaattgttttgttgcaaacaaaattctgaatgttaacttaacgccaagaacttggtgaa 5100

ccataataggatataccaagattttcaatacttttttcggtgtgttatttttcatccttg 5160

ttgaacttcacgctatcttcaaaagtaacctcatcttttcatcctatagGAATTTCTTCA 5220

AGCTAGAGATGATGAAATCCAAAAACTAGAggtagatctatcttcagatatttcctttat 5280

cttcactagattttaagcatgtggtaactgaatgtgggatcaaaatgtaaattttagtgt 5340

aagtttgtgatcatagctgtaagtctatttacttaacaggctttattgaaatattatttt 5400

ctcatactggcataacttttcaagtattctgcaGAGTTTGCTCAAAGGGGCGACAGCAGA 5460

AGCCGATAAAGTTATGCTCACAGATGCTCCACTCCTCTTTCCTCCTGGCCAggtgggcat 5520

gctagtttattttgtctccctagcctcttattatcagattaaagatactgaataatatcc 5580

atcaaagaatctggttaaactcattgtttgggatatttcagtaaagctttaaatatgcta 5640

ctcaagacataaatttaggatgaaagttggtgctttgttttctctttgactgatatttaa 5700

gtgaaattaatgaatgtttatgatatagtagtatagcttatcgatatatactcaccttgt 5760

tccttctttcaggatttttcgtttttctttagcgtatatgtaacaaaagaagtatcacaa 5820

attgttgtactgtctctcaactcttgaacgtgtgcccttgtaGTTGGCATTGGCGTCGTT 5880

ACGTATTGCAAATGGGGTTCTTGGAGTGATTGACTTTGATAGGTtatttctcttattcta 5940

tatccatcccgattatttacccttttaatctttccatgatattaatctcgtggtcttgaa 6000

tttctgtatatgcaaagctttttactggttgtattttcaactaaactgcatttggtgcag 6060

gtACCTAGAGAACATTGTTTCTCAACCGAACTCTGAGCACACGACTTCAGAGCTTACAAA 6120

GTTACTTGATAACATCGAATATTTGGTAtttactaacttccataattatcttaacaatga 6180

aacttgcatatctaaaaggaagttttactagatgctctctcttatctcaggtaAAGAACT 6240 CYCHseq5

ACAAGTGCCCAAGTGAAAAGGACATGAAGCATATCAACCGGAAGCTAAAATCTTGTCTAG 6300

GACATAGTTCTTCACATGACGAgtacactaacaacctctcttctactaattcttttaacc 6360

actatatatatttcataagctttgcttctgcaattgactcccttctctatttatcttcat 6420

cagGAGTAAGAAACGGGAGAAGAGATCAAAACACAAGTCCCATAGGAGCTCCAATGATAC 6480 CYCH_50ntF

ACCAAACGGGGCACCGCCACCCATAGGT**ATG**GTGAGCAAGGGCGAGGAGGATAACATGGC 6540

CATCATCAAGGAGTTCATGCGCTTCAAGGTGCACATGGAGGGCTCCGTGAACGGCCACGA 6600

GTTCGAGATCGAGGGCGAGGGCGAGGGCCGCCCCTACGAGGGCACCCAGACCGCCAAGCT 6660

GAAGGTGACCAAGGGTGGCCCCCTGCCCTTCGCCTGGGACATCCTGTCCCCTCAGTTCAT 6720

GTACGGCTCCAAGGCCTACGTGAAGCACCCCGCCGACATCCCCGACTACTTGAAGCTGTC 6780

CTTCCCCGAGGGCTTCAAGTGGGAGCGCGTGATGAACTTCGAGGACGGCGGCGTGGTGAC 6840

CGTGACCCAGGACTCCTCCCTGCAGGACGGCGAGTTCATCTACAAGGTGAAGCTGCGCGG 6900

CACCAACTTCCCCTCCGACGGCCCCGTAATGCAGAAGAAGACCATGGGCTGGGAGGCCTC 6960

CTCCGAGCGGATGTACCCCGAGGACGGCGCCCTGAAGGGCGAGATCAAGCAGAGGCTGAA 7020

GCTGAAGGACGGCGGCCACTACGACGCTGAGGTCAAGACCACCTACAAGGCCAAGAAGCC 7080

CGTGCAGCTGCCCGGCGCCTACAACGTCAACATCAAGTTGGACATCACCTCCCACAACGA 7140

GGACTACACCATCGTGGAACAGTACGAACGCGCCGAGGGCCGCCACTCCACCGGCGGCAT 7200

GGACGAGCTGTACAAG**TGACTATATTACCCTGTTATCCCTAGCGTA**gtttctcgtactgc 7260 CYCH_50ntR

cattagcaattcttcctttgcactttggtttagttttaatcttattgaatgagaaattcg 7320

tcttttatagtttcttccccgttgttgttcttactagtaaatttgagcagattatgtata 7380 CYCHseq3

acaatgatattggccataagttttgtttattctgaagatgttaatgaatttataacaaaa 7440

cttccatttattttaagttttggtgtacataacaatcgtcaaaaatggtttgtttgcgat 7500

aatgatcaactttttacatgatcaaaagtttttccagacattttgatactttctagttac 7560

taccacatctgttcttacacagaaacaaatggatatgactagtgtagcggtaactatcaa 7620

atcctaataataacaatgaaaatttgtattaaataaccaaaaaccatcgaaaataaaatt 7680

tggattgtgaatgaaacttgtaaagatgcttgattggttgtgctaaatgtcttttacagc 7740

cataatatatttgttaaagactaaattgttgccaattatgttatagtttttttctttaac 7800

ttagagccccaaaaaaaatctttttgttttgactgtaaatctttaattttctacatccta 7860

ttttgtgttgtagatttttttttttttgtagatttttttttttttttctctcctctaact 7920

ttttgtcacgattattttcaactaataatctaaaatgtagttatatgaataacagataaa 7980

gtattttatacatttagacaaataatttataaatttatatagattccatttacctagatg 8040

tgcagaattctttttgtgtttttattataaaattgttattaaataatttatatatattcc 8100

atctacctagatgtgcataattcaatttgtgtttttattttataattgctattaaataat 8160

tttagtaattatgatatacaattttctcattttgaaaaaaggtaatttataaatataaaa 8220

ttcaaatatttcttaatcataatttttagttacaactaaaatatttattgccaaaatcct 8280

atatttttcaaatctacaaaaccaagaggttaccgtcaaaattctacagttagagcttat 8340

acacttattgccaatattatacataaaatattttaccttatgcaaatgcctatgctttta 8400

tgggtcatagacggacctggctactgtgtaactaatcacccacataatttatcagtcaaa 8460

cataacaaattctttttgtgccaaaaagtcaaaaacgttagaaactggattttggaagtt 8520

gatgcatgcaaatagcaacttgtcgtgccgcgctatataaacgctgcatcaagttttcgt 8580

aattaaCTAAAACCAAATTAGTATTAATCCGAAAACTTGAAACTGTGGCTTCCTCCTCAA 8640

ATCTCCGATCAACGGAAACAAA**ATG**GCTCACATGGTGAGAGCGAGCTCTGGCCTTTCTTA 8700 At5g27630

CCCGGAGAGATTCTACGCCGCCGCATCGTACGTCGGCCTCGACGGATCTCAATCGTCGGT 8760

CAAACAACTC**GGATCC** 8776 CYCH_Flank2

**BamHI**

**C-mCherrystop-SpR cassette insertion between the gene specific primers CYCHseq5 and CYCGseq3**

GAACTACAAGTGCCCAAGTGAAAAGGACATGAAGCATATCAACCGGAAGCTAAAATCTTG 60 CYCHseq5

TCTAGGACATAGTTCTTCACATGACGAgtacactaacaacctctcttctactaattcttt 120

taaccactatatatatttcataagctttgcttctgcaattgactcccttctctatttatc 180

ttcatcagGAGTAAGAAACGGGAGAAGAGATCAAAACACAAGTCCCATAGGAGCTCCAAT 240 CYCH_50ntF

GATACACCAAACGGGGCACCGCCACCCATAGGT**ATG**GTGAGCAAGGGCGAGGAGGATAAC 300 CmCherrystopF

ATGGCCATCATCAAGGAGTTCATGCGCTTCAAGGTGCACATGGAGGGCTCCGTGAACGGC 360

CACGAGTTCGAGATCGAGGGCGAGGGCGAGGGCCGCCCCTACGAGGGCACCCAGACCGCC 420

AAGCTGAAGGTGACCAAGGGTGGCCCCCTGCCCTTCGCCTGGGACATCCTGTCCCCTCAG 480

TTCATGTACGGCTCCAAGGCCTACGTGAAGCACCCCGCCGACATCCCCGACTACTTGAAG 540

CTGTCCTTCCCCGAGGGCTTCAAGTGGGAGCGCGTGATGAACTTCGAGGACGGCGGCGTG 600 mCherry

GTGACCGTGACCCAGGACTCCTCCCTGCAGGACGGCGAGTTCATCTACAAGGTGAAGCTG 660

CGCGGCACCAACTTCCCCTCCGACGGCCCCGTAATGCAGAAGAAGACCATGGGCTGGGAG 720

GCCTCCTCCGAGCGGATGTACCCCGAGGACGGCGCCCTGAAGGGCGAGATCAAGCAGAGG 780

CTGAAGCTGAAGGACGGCGGCCACTACGACGCTGAGGTCAAGACCACCTACAAGGCCAAG 840

AAGCCCGTGCAGCTGCCCGGCGCCTACAACGTCAACATCAAGTTGGACATCACCTCCCAC 900

AACGAGGACTACACCATCGTGGAACAGTACGAACGCGCCGAGGGCCGCCACTCCACCGGC 960

GGCATGGACGAGCTGTACAAG**TGACTATATTACCCTGTTATCCCTAGCGTA**actagtctc 1020 **I-SceI**

atgttaccgatgctattcggaagaacggcaactaagctgccgggtttgaaacacggatga 1080

tctcgcggagggtagcatgttgattgtaacgatgacagagcgttgctgcctgtgatcaat 1140

tcgggcacgaacccagtggacataagcctcgttcggttcgtaagctgtaatgcaagtagc 1200

gtaactgccgtcacgcaactggtccagaaccttgaccgaacgcagcggtggtaacggcgc 1260

agtggcggttttcatggcttcttgtt**ATG**ACATGTTTTTTTGGGGTACAGTCTATGCCTC 1320

GGGCATCCAAGCAGCAAGCGCGTTACGCCGTGGGTCGATGTTTGATGTTATGGAGCAGCA 1380

ACGATGTTACGCAGCAGGGCAGTCGCCCTAAAACAAAGTTAAACATCATGGGGGAAGCGG 1440

TGATCGCCGAAGTATCGACTCAACTATCAGAGGTAGTTGGCGTCATCGAGCGCCATCTCG 1500

AACCGACGTTGCTGGCCGTACATTTGTACGGCTCCGCAGTGGATGGCGGCCTGAAGCCAC 1560

ACAGTGATATTGATTTGCTGGTTACGGTGACCGTAAGGCTTGATGAAACAACGCGGCGAG 1620

CTTTGATCAACGACCTTTTGGAAACTTCGGCTTCCCCTGGAGAGAGCGAGATTCTCCGCG 1680

CTGTAGAAGTCACCATTGTTGTGCACGACGACATCATTCCGTGGCGTTATCCAGCTAAGC 1740

GCGAACTGCAATTTGGAGAATGGCAGCGCAATGACATTCTTGCAGGTATCTTCGAGCCAG 1800

CCACGATCGACATTGATCTGGCTATCTTGCTGACAAAAGCAAGAGAACATAGCGTTGCCT 1860

TGGTAGGTCCAGCGGCGGAGGAACTCTTTGATCCGGTTCCTGAACAGGATCTATTTGAGG 1920

CGCTAAATGAAACCTTAACGCTATGGAACTCGCCGCCCGACTGGGCTGGCGATGAGCGAA 1980

ATGTAGTGCTTACGTTGTCCCGCATTTGGTACAGCGCAGTAACCGGCAAAATCGCGCCGA 2040

AGGATGTCGCTGCCGACTGGGCAATGGAGCGCCTGCCGGCCCAGTATCAGCCCGTCATAC 2100

TTGAAGCTAGACAGGCTTATCTTGGACAAGAAGAAGATCGCTTGGCCTCGCGCGCAGATC 2160

AGTTGGAAGAATTTGTCCACTACGTGAAAGGCGAGATCACCAAGGTAGTCGGCAAA**TAA**t 2220

gtctagctagaaattcgttcaagccgacgccgcttcgccGAAGTG**CTATATTACCCTGTT** 2280 I-SceISpR

**ATCCCTAGCGTA**gtttctcgtactgccattagcaattcttcctttgcactttggtttagt 2340 CYCH_50ntR

tttaatcttattgaatgagaaattcgtcttttatagtttcttccccgttgttgttcttac 2400 CYCHseq3

tagt 2404

**C-GFPstop-SpR cassette insertion between the gene specific primers CYCHseq5 and CYCGseq3**

GAACTACAAGTGCCCAAGTGAAAAGGACATGAAGCATATCAACCGGAAGCTAAAATCTTG 60 CYCHseq5

TCTAGGACATAGTTCTTCACATGACGAgtacactaacaacctctcttctactaattcttt 120

taaccactatatatatttcataagctttgcttctgcaattgactcccttctctatttatc 180

ttcatcagGAGTAAGAAACGGGAGAAGAGATCAAAACACAAGTCCCATAGGAGCTCCAAT 240 CYCH_50ntF

GATACACCAAACGGGGCACCGCCACCCATAGGT**ATG**GTGAGCAAGGGCGAGGAGCTGTTC 300 **CGFPstopF**

ACCGGGGTGGTGCCCATCCTGGTCGAGCTGGACGGCGACGTAAACGGCCACAAGTTCAGC 360

GTGTCCGGCGAGGGCGAGGGCGATGCCACCTACGGCAAGCTGACCCTGAAGTTCATCTGC 420

ACCACCGGCAAGCTGCCCGTGCCCTGGCCCACCCTCGTGACCACCCTGACCTACGGCGTG 480

CAGTGCTTCAGCCGCTACCCCGACCACATGAAGCAGCACGACTTCTTCAAGTCCGCCATG 540 GFP

CCCGAAGGCTACGTCCAGGAGCGCACCATCTTCTTCAAGGACGACGGCAACTACAAGACC 600

CGCGCCGAGGTGAAGTTCGAGGGCGACACCCTGGTGAACCGCATCGAGCTGAAGGGCATC 660

GACTTCAAGGAGGACGGCAACATCCTGGGGCACAAGCTGGAGTACAACTACAACAGCCAC 720

AACGTCTATATCATGGCCGACAAGCAGAAGAACGGCATCAAGGTGAACTTCAAGATCCGC 780

CACAACATCGAGGACGGCAGCGTGCAGCTCGCCGACCACTACCAGCAGAACACCCCCATC 840

GGCGACGGCCCCGTGCTGCTGCCCGACAACCACTACCTGAGCACCCAGTCCGCCCTGAGC 900

AAAGACCCCAACGAGAAGCGCGATCACATGGTCCTGCTGGAGTTCGTGACCGCCGCCGGG 960

ATCACTCTCGGCATGGACGAGCTGTACAAG**TAACTATATTACCCTGTTATCCCTAGCGTA** 1020 **I-SceI**

actagtctcatgttaccgatgctattcggaagaacggcaactaagctgccgggtttgaaa 1080

cacggatgatctcgcggagggtagcatgttgattgtaacgatgacagagcgttgctgcct 1140

gtgatcaattcgggcacgaacccagtggacataagcctcgttcggttcgtaagctgtaat 1200

gcaagtagcgtaactgccgtcacgcaactggtccagaaccttgaccgaacgcagcggtgg 1260

taacggcgcagtggcggttttcatggcttcttgtt**ATG**ACATGTTTTTTTGGGGTACAGT 1320

CTATGCCTCGGGCATCCAAGCAGCAAGCGCGTTACGCCGTGGGTCGATGTTTGATGTTAT 1380

GGAGCAGCAACGATGTTACGCAGCAGGGCAGTCGCCCTAAAACAAAGTTAAACATCATGG 1440

GGGAAGCGGTGATCGCCGAAGTATCGACTCAACTATCAGAGGTAGTTGGCGTCATCGAGC 1500

GCCATCTCGAACCGACGTTGCTGGCCGTACATTTGTACGGCTCCGCAGTGGATGGCGGCC 1560

TGAAGCCACACAGTGATATTGATTTGCTGGTTACGGTGACCGTAAGGCTTGATGAAACAA 1620

CGCGGCGAGCTTTGATCAACGACCTTTTGGAAACTTCGGCTTCCCCTGGAGAGAGCGAGA 1680

TTCTCCGCGCTGTAGAAGTCACCATTGTTGTGCACGACGACATCATTCCGTGGCGTTATC 1740

CAGCTAAGCGCGAACTGCAATTTGGAGAATGGCAGCGCAATGACATTCTTGCAGGTATCT 1800

TCGAGCCAGCCACGATCGACATTGATCTGGCTATCTTGCTGACAAAAGCAAGAGAACATA 1860

GCGTTGCCTTGGTAGGTCCAGCGGCGGAGGAACTCTTTGATCCGGTTCCTGAACAGGATC 1920

TATTTGAGGCGCTAAATGAAACCTTAACGCTATGGAACTCGCCGCCCGACTGGGCTGGCG 1980

ATGAGCGAAATGTAGTGCTTACGTTGTCCCGCATTTGGTACAGCGCAGTAACCGGCAAAA 2040

TCGCGCCGAAGGATGTCGCTGCCGACTGGGCAATGGAGCGCCTGCCGGCCCAGTATCAGC 2100

CCGTCATACTTGAAGCTAGACAGGCTTATCTTGGACAAGAAGAAGATCGCTTGGCCTCGC 2160

GCGCAGATCAGTTGGAAGAATTTGTCCACTACGTGAAAGGCGAGATCACCAAGGTAGTCG 2220

GCAAA**TAA**tgtctagctagaaattcgttcaagccgacgccgcttcgcc**GAAGTGCTATAT** 2280 I-SceISpR

**TACCCTGTTATCCCTAGCGTA**gtttctcgtactgccattagcaattcttcctttgcactt 2340 CYCH_50ntR

tggtttagttttaatcttattgaatgagaaattcgtcttttatagtttcttccccgttgt 2400 CYCHseq3

tgttcttactagt 2413

***CYH:GFP* construct between the gene specific primers CYCHseq5 and CYCHseq3**

GAACTACAAGTGCCCAAGTGAAAAGGACATGAAGCATATCAACCGGAAGCTAAAATCTTG 60 CYCHseq5

TCTAGGACATAGTTCTTCACATGACGAgtacactaacaacctctcttctactaattcttt 120

taaccactatatatatttcataagctttgcttctgcaattgactcccttctctatttatc 180

ttcatcagGAGTAAGAAACGGGAGAAGAGATCAAAACACAAGTCCCATAGGAGCTCCAAT 240 CYCH_50ntF

GATACACCAAACGGGGCACCGCCACCCATAGGT**ATG**GTGAGCAAGGGCGAGGAGCTGTTC 300

ACCGGGGTGGTGCCCATCCTGGTCGAGCTGGACGGCGACGTAAACGGCCACAAGTTCAGC 360

GTGTCCGGCGAGGGCGAGGGCGATGCCACCTACGGCAAGCTGACCCTGAAGTtCATCTGC 420

ACCACCGGCAAGCTGCCCGTGCCCTGGCCCACCCTCGTGACCACCcTGACcTACGGCGTG 480

CAGTGCTTCAGCCGCTACCCCGACCACATGAAGCAGCACGACTTCTTCAAGTCCGCCATG 540

CCCGAAGGCTACGTCCAGGAGCGCACCATCTTCTTCAAGGACGACGGCAACTACAAGACC 600

CGCGCCGAGGTGAAGTTCGAGGGCGACACCCTGGTGAACCGCATCGAGCTGAAGGGCATC 660

GACTTCAAGGAGGACGGCAACATCCTGGGGCACAAGCTGGAGTACAACTACAACAGCCAC 720

AACGTCTATATCATGGCCGACAAGCAGAAGAACGGCATCAAGGTGAACTTCAAGATCCGC 780

CACAACATCGAGGACGGCAGCGTGCAGCTCGCCGACCACTACCAGCAGAACACCCCCATC 840

GGCGACGGCCCCGTGCTGCTGCCCGACAACCACTACCTGAGCACCCAGTCCGCCCTGAGC 900

AAAGACCCCAACGAGAAGCGCGATCACATGGTCCTGCTGGAGTTCGTGACCGCCGCCGGG 960

ATCACTCTCGGCATGGACGAGCTGTACAAG**TAACTATATTACCCTGTTATCCCTAGCGTA** 1020 **I-SceI**

gtttctcgtactgccattagcaattcttcctttgcactttggtttagttttaatcttatt 1080 CYCH_50ntR

gaatgagaaattcgtcttttatagtttcttccccgttgttgttcttactagt 1132 CYCHseq3

**CYCH-mCherry (64.83 kDa)**

MADFQTSTQRAKWIFTPQKLAERYKAANQRAVQMLEKCGTTQVEVDASGSLTYPKDKVGS 60

GDQADKKLKPLSADEERFMRAFYEAKVQEVCSAFAFPHKIQATALQYFKRFYLQWSVMQH 120

HPKEIMLTCVYAACKIEENHVSAEEIGKGINQDHRIILKYEMAVLQSLEFDLIVYAPYRA 180

IEGFVNNMEEFLQARDDEIQKLESLLKGATAEADKVMLTDAPLLFPPGQLALASLRIANG 240

VLGVIDFDRYLENIVSQPNSEHTTSELTKLLDNIEYLVKNYKCPSEKDMKHINRKLKSCL 300

GHSSSHDESKKREKRSKHKSHRSSNDTPNGAPPPIGMVSKGEEDNMAIIKEFMRFKVHME 360

GSVNGHEFEIEGEGEGRPYEGTQTAKLKVTKGGPLPFAWDILSPQFMYGSKAYVKHPADI 420 mCherry

PDYLKLSFPEGFKWERVMNFEDGGVVTVTQDSSLQDGEFIYKVKLRGTNFPSDGPVMQKK 480

TMGWEASSERMYPEDGALKGEIKQRLKLKDGGHYDAEVKTTYKAKKPVQLPGAYNVNIKL 540

DITSHNEDYTIVEQYERAEGRHSTGGMDELYK. 573

**CYCH-GFP(65.05 kDa)**

MADFQTSTQRAKWIFTPQKLAERYKAANQRAVQMLEKCGTTQVEVDASGSLTYPKDKVGS 60

GDQADKKLKPLSADEERFMRAFYEAKVQEVCSAFAFPHKIQATALQYFKRFYLQWSVMQH 120

HPKEIMLTCVYAACKIEENHVSAEEIGKGINQDHRIILKYEMAVLQSLEFDLIVYAPYRA 180

IEGFVNNMEEFLQARDDEIQKLESLLKGATAEADKVMLTDAPLLFPPGQLALASLRIANG 240

VLGVIDFDRYLENIVSQPNSEHTTSELTKLLDNIEYLVKNYKCPSEKDMKHINRKLKSCL 300

GHSSSHDESKKREKRSKHKSHRSSNDTPNGAPPPIGMVSKGEELFTGVVPILVELDGDVN 360

GHKFSVSGEGEGDATYGKLTLKFICTTGKLPVPWPTLVTTLTYGVQCFSRYPDHMKQHDF 420 GFP

FKSAMPEGYVQERTIFFKDDGNYKTRAEVKFEGDTLVNRIELKGIDFKEDGNILGHKLEY 480

NYNSHNVYIMADKQKNGIKVNFKIRHNIEDGSVQLADHYQQNTPIGDGPVLLPDNHYLST 540

QSALSKDPNEKRDHMVLLEFVTAAGITLGMDELYK. 576

**(g)**

***HISTONE H3.1:mCherry* insertion in the SpeI-BamHI sites of pGAPBRKm and pGAPBRHyg vectors**

**SpeI**

**ACTAGT**CAAATACTTGCTTCGCCAAGAAAACTCGCTCAAACCCTCCTTGCAGTTTTCAAG 60 H3.1_Flank1

CTTCTCCAAAACCGGAACCAACTCGTTAGGTTTAGCTACTCTTACGTTCAATTCTTCAAA 120

TCTTATCCTCATCAATCTTATGGCGGAGAAGTAGTCTTGTACCATTAGTTCTGTCATTTC 180

AATAACGATTTTGTTCAGCTTCGACTGTGGAGCTTTTGGCCTATCACTTATATGTTCGAA 240

TAGTTCTACTAGGAAATCAATTTGTTTGAGAATGCAATCCATTGGGTAAGACGAGACTCG 300

CTGAGTCTCCAAACGCTTATCTTCGTTTTTTTCTTTTATGTTTGGAGTAATACCTAAAAC 360

CTCCGCAATAGATAAGTAACAATCTAGATATTGTTTGTACctattcaaaaccaaattttg 420

atgaatcataaaaatttcgattaaattgtgatttaacgttaaatcaaaaacaaaaatata 480

taataaagaataatcgacttatcgattgtgctcatctatatcctaatcaatccaacagta 540

tatagaaatcttacCATTGAACCCATGGAGTCAACTCTCTAGTAAAACGAGATGAATTTA 600

CATTAAGATCATTCAATTTAAGATTACTCCCTCCTTGAGTATAAATCAAAGTACGATGAT 660

TAATATTGTTCCGAAGACTATCTTCTCCATTATACCCACTTTCTGATTTAACCATTTTAT 720

GGAGAAGAATGAGACATTTAGCAGCCACACATACGTCCGTCGTCACACGCAGCCTATGCA 780

AGATCGCATCAACAGTATCAGGACCGTAACACGTGTCAATAGTTGATTGGAGAAAAGTGA 840

CGTATTTGTCTGAAGGCGGGTTGTTTGAGGTATGAGACGTGGCTTTCAAGAGAGCTAAAT 900

CAATGGTTTTGGCATTCACGGAGCTACACAAATGAACCACATTGAGTTTCATCTGTGAGG 960

CTTCGTCTTTCAATATGCCATTCAAGGTCGCTAATTTTCC**CAT**ttgaaatatagttgttc 1020 AT5G65370

tattccaatgttatgaaactttagcaatgtggctttgctttaataatgtttataggtttc 1080

acaatgtgtttttggtttgatttgattgtaaggtatcaaatatatatgttgtataatagt 1140

catctattgaacatgtgtattagatggaattttcatgccaggatttaattagatattcag 1200

ttttgaaaattaaatacacatggattttattgcaataactatttaatttattattatttt 1260

atctatttataaactaaatttaatgttattttcttaaagtttcataaatgaatactaaag 1320

gtttaatttacctctatctagagtactttgattatgatatatttttgataaaaagttcat 1380

taagaaaatctgacaacatttacatacgtatatggataagatttctattaattgacttat 1440

ctctcaatttattttaagtgaaaattgaactaaaaaaacttttaaaatgacgttttctca 1500

acacattttgtaagtattcacattatacaaaattacagacactttaatttgtgagtataa 1560

cacgtattattaaataaacagaaatgtaagaaatataaaacattattgcttcagtaatca 1620

atttcaaatcaatcaactaactcaactctcaatctttgtatctagatttaaagcctgatt 1680

ggctgattttacaccactcacacgttaataatagttaacgtgcgtcaatgatattattat 1740

ttctcgacttatgtattctacatgggccacatcagcagaaactaagattataatttcgtg 1800

gactgatacacaacatattaacgggcttaacctagactctacaacatgggcttttatttc 1860

atagaggccttcactgtcaggttccaaaatttagcgaaaaggtttaaaagacgcgcctaa 1920

ataaaaaaactaaaaaattgtgaaggaagctaaaaaacttgggcgctaaagaaaactaac 1980

ctgattggctgctttagacgtgacgcggatcactaaaattaaaacaatcacaaccgttga 2040

ttaaataaaagatctaacgacgcaaaacaaatacttcccttgtatacgcttcgctataaa 2100

aaccaatccagtatccctttagtctcaacATCTTCTTCTTCTTCATCAAATCTCACAAAT 2160

CTTCAACACTTAATCACAAATCTCAAAGCTTCGGATACCAA**ATG**GCTCGTACCAAGCAAA 2220 **Histone H3.1**

CCGCAAGGAAATCCACCGGAGGAAAAGCCCCAAGGAAACAACTCGCAACAAAGGCGGCGA 2280 AT5G65360

GGAAATCAGCTCCGGCGACCGGAGGAGTAAAGAAGCCACACAGATTCCGTCCTGGAACTG 2340

TTGCCCTAAGAGAAATCAGGAAGTATCAGAAGAGCACTGAGCTTCTGATCCGCAAGCTTC 2400

CGTTCCAGCGTTTGGTTCGTGAGATCGCTCAGGATTTCAAAACAGATCTGCGTTTCCAGA 2460

GCAGCGCCGTCGCAGCACTTCAGGAAGCGGCTGAAGCATACCTCGTTGGATTGTTTGAAG 2520 H3.1seq5

ACACCAATCTTTGCGCGATTCATGCTAAGAGAGTCACTATCATGCCTAAGGATATTCAAT 2580 H3.1_50ntF

TGGCGAGGAGAATTAGAGGCGAGAGGGCT**ATG**GTGAGCAAGGGCGAGGAGGATAACATGG 2640

CCATCATCAAGGAGTTCATGCGCTTCAAGGTGCACATGGAGGGCTCCGTGAACGGCCACG 2700

AGTTCGAGATCGAGGGCGAGGGCGAGGGCCGCCCCTACGAGGGCACCCAGACCGCCAAGC 2760

TGAAGGTGACCAAGGGTGGCCCCCTGCCCTTCGCCTGGGACATCCTGTCCCCTCAGTTCA 2820

TGTACGGCTCCAAGGCCTACGTGAAGCACCCCGCCGACATCCCCGACTACTTGAAGCTGT 2880 mCherry

CCTTCCCCGAGGGCTTCAAGTGGGAGCGCGTGATGAACTTCGAGGACGGCGGCGTGGTGA 2940

CCGTGACCCAGGACTCCTCCCTGCAGGACGGCGAGTTCATCTACAAGGTGAAGCTGCGCG 3000

GCACCAACTTCCCCTCCGACGGCCCCGTAATGCAGAAGAAGACCATGGGCTGGGAGGCCT 3060

CCTCCGAGCGGATGTACCCCGAGGACGGCGCCCTGAAGGGCGAGATCAAGCAGAGGCTGA 3120

AGCTGAAGGACGGCGGCCACTACGACGCTGAGGTCAAGACCACCTACAAGGCCAAGAAGC 3180

CCGTGCAGCTGCCCGGCGCCTACAACGTCAACATCAAGTTGGACATCACCTCCCACAACG 3240

AGGACTACACCATCGTGGAACAGTACGAACGCGCCGAGGGCCGCCACTCCACCGGCGGCA 3300

TGGACGAGCTGTACAAG**TGACTATATTACCCTGTTATCCCTAGCGTA**GAAGGAGATTGAA 3360 **I-SceI**

GTACTCTAGACTGTGATCGTTATGCTTATGTATATCTTTCGTTTTCCCTAATTTCGTGTT 3420 H3.1_50ntR

TTAGGGTTGGATTAGGTTTTGCGTTTATGTTGTTCGATATCTAACGGATCAAAATCTCTC 3480 H3.1seq3

CTTCCTTAGCAAAGTTTGAAAACTCCCTCCACATTTTCATCTCCTTTTCCACATAACCAA 3540

CTCAACACTTTTCAATTTCTATAACAATTTCGAGAAATCAAAACCAGaattaataaaatt 3600

aaccagttgattctttctat**ATG**GCTCGTACCAAGCAGACTGCTAGAATATCCACCGGAG 3660 AT5G65350

GAAAAGCTCCGAGGAAGCAATTAGCGCCAAAAGCGGCGAGACAATCTGCACCAGCAACGG 3720

GAGGAGTGAAGAAGCCACACAGATTCCGTCCAGGAACTGTTGCGTTGAGAGATATTAGGA 3780

AATACCAAAAGAGTACCGAGATTTTGATCAGAAAGCTTCCGTTTCAGCGACTTGTTCGTG 3840

AGATCGCTCAAGATTTCAAAACGGATCTGAGGTTTCAGAGCAGTGCCGTCGCTGCTCTCC 3900

AGGAAGCTGCTGAGGCTTACCTTGTTGGGTTATTCGAAGATACAAACCTCTGTGCGATTC 3960

ACGCAAAGAGGGTGACGATCATGCCTAAAGAGATCCAATTAGCGAGAAGGATTAGAGGTG 4020

AGAGAGCTCGAGGAGAG**TGA**TGATTTTGTTGTTCTGCGAACTGGGATTTCATGGGTTTTT 4080

CGATTTACGGATTTGGGTTTATGTAAGTTTCGATCTTTGCTGCTTTGGAATGTCTTGTAG 4140

GTTGGTTAGTTAATGCTTTTATTTGCAGCTAAGGTGTTTGTGGAAATTCCCCAATGAAAC 4200

TTGTAATGTTCAATACAATCCTTACTTTGGTTCTCaaaagtctttagaaatctatggttc 4260

aagattttaccttataattctccaatgtcatgtgtaggatgtcttcttactctgtctccg 4320

actaaccagacagcatacacttgtgtgattacataaagacagttacacagatcaaccgga 4380

accaaacacaaacactctaaataagatgcaagacttatatattaacatcaaagaagacaa 4440

tgttgtggtttgaataattagatcatttgtgtccgatagaagattctattgacacttcat 4500

cgccgtggagttgtagattgaggatgaatgacatgattgctctggtcctaaccaaagcaa 4560

ttgatggctccaacattctcataaccaggccgctactatctcccaaagaaagactagcat 4620

tggtagcctgatgtcaatgtctgttagatcatcaatcactggtgcttctgttccattata 4680

aactattgagaattggttcagaatcatcatcatatatccatctcttctttcctaacata**G** 4740 H3.1_Flank2

**GATCC** 4745

**BamHI**

***HISTONE H3.1:GFP* construct between the gene specific primers H3.1seq5 and H3.1seq3**

GAAGCGGCTGAAGCATACCTCGTTGGATTGTTTGAAGACACCAATCTTTGCGCGATTCAT 60 H3.1seq5

GCTAAGAGAGTCACTATCATGCCTAAGGATATTCAATTGGCGAGGAGAATTAGAGGCGAG 120 H3.1_50ntF

AGGGCT**ATG**GTGAGCAAGGGCGAGGAGCTGTTCACCGGGGTGGTGCCCATCCTGGTCGAG 180

CTGGACGGCGACGTAAACGGCCACAAGTTCAGCGTGTCCGGCGAGGGCGAGGGCGATGCC 240

ACCTACGGCAAGCTGACCCTGAAGTTCATCTGCACCACCGGCAAGCTGCCCGTGCCCTGG 300

CCCACCCTCGTGACCACCCTGACCTACGGCGTGCAGTGCTTCAGCCGCTACCCCGACCAC 360

ATGAAGCAGCACGACTTCTTCAAGTCCGCCATGCCCGAAGGCTACGTCCAGGAGCGCACC 420

ATCTTCTTCAAGGACGACGGCAACTACAAGACCCGCGCCGAGGTGAAGTTCGAGGGCGAC 480

ACCCTGGTGAACCGCATCGAGCTGAAGGGCATCGACTTCAAGGAGGACGGCAACATCCTG 540

GGGCACAAGCTGGAGTACAACTACAACAGCCACAACGTCTATATCATGGCCGACAAGCAG 600

AAGAACGGCATCAAGGTGAACTTCAAGATCCGCCACAACATCGAGGACGGCAGCGTGCAG 660

CTCGCCGACCACTACCAGCAGAACACCCCCATCGGCGACGGCCCCGTGCTGCTGCCCGAC 720

AACCACTACCTGAGCACCCAGTCCGCCCTGAGCAAAGACCCCAACGAGAAGCGCGATCAC 780

ATGGTCCTGCTGGAGTTCGTGACCGCCGCCGGGATCACTCTCGGCATGGACGAGCTGTAC 840

AAG**TAACTATATTACCCTGTTATCCCTA**GCGTAGAAGGAGATTGAAGTACTCTAGACTGT 900 **I-SceI**

GATCGTTATGCTTATGTATATCTTTCGTTTTCCCTAATTTCGTGTTTTAGGGTTGGATTA 960 H3.1:50ntR

GGTTTTGCGTTTATGTTGTTCGATATCTAACGGATCAAAATCTCTCCTTCCTTAGCA 1017 H3.1seq3

**HISTONE H3.1:mCherry (41.97 kDa)**

MARTKQTARKSTGGKAPRKQLATKAARKSAPATGGVKKPHRFRPGTVALREIRKYQKSTE 60

LLIRKLPFQRLVREIAQDFKTDLRFQSSAVAALQEAAEAYLVGLFEDTNLCAIHAKRVTI 120

MPKDIQLARRIRGERAMVSKGEEDNMAIIKEFMRFKVHMEGSVNGHEFEIEGEGEGRPYE 180

GTQTAKLKVTKGGPLPFAWDILSPQFMYGSKAYVKHPADIPDYLKLSFPEGFKWERVMNF 240 mCherry

EDGGVVTVTQDSSLQDGEFIYKVKLRGTNFPSDGPVMQKKTMGWEASSERMYPEDGALKG 300

EIKQRLKLKDGGHYDAEVKTTYKAKKPVQLPGAYNVNIKLDITSHNEDYTIVEQYERAEG 360

RHSTGGMDELYK. 373

**HISTONE H3.1:mCherry (42.19 kDa)**

MARTKQTARKSTGGKAPRKQLATKAARKSAPATGGVKKPHRFRPGTVALREIRKYQKSTE 60

LLIRKLPFQRLVREIAQDFKTDLRFQSSAVAALQEAAEAYLVGLFEDTNLCAIHAKRVTI 120

MPKDIQLARRIRGERAMVSKGEELFTGVVPILVELDGDVNGHKFSVSGEGEGDATYGKLT 180

LKFICTTGKLPVPWPTLVTTLTYGVQCFSRYPDHMKQHDFFKSAMPEGYVQERTIFFKDD 240 GFP

GNYKTRAEVKFEGDTLVNRIELKGIDFKEDGNILGHKLEYNYNSHNVYIMADKQKNGIKV 300

NFKIRHNIEDGSVQLADHYQQNTPIGDGPVLLPDNHYLSTQSALSKDPNEKRDHMVLLEF 360

VTAAGITLGMDELYK. 376

**Figure S2.** Example templates for planning the recombineering experiments and sequences of modified plant genes in pGAP and pGAPBR vectors constructed in this work.

(a) Sequences of EcoRI-BamHI cleaved pGAPKm and pGAPHyg vectors (NCBI Accession No: EU933992.1 and EU933993.1) used for gap-repair cloning of *CDKF;1*, *CDKD;1*, *CDKD;2* and *CDKD;3* gene constructs. Abbreviations: OCSpA: 3’-UTR carrying polyadenylation sequences of pTiAch5 octopine synthase gene; KmR/aphII: kanamycin resistance/aminoglycoside phosphotransferase II gene; pNOS: promoter of pTiC58 nopaline synthase gene; T-DNA: transferred DNA; RB: right 25bp border sequence of pTiC58 T-DNA, black arrow indicates the direction of T-DNA transfer; RK2oriT: conjugational transfer origin of IncP-type RK2 plasmid, nicking site is indicated; RK2oriV: DNA replication origin of RK2 plasmid; LB: left 25bp border of pTiAch5 T-DNA; pBSK ori: DNA replication origin of plasmid pBSKII(-); AmpR/CbR: ampicillin/carbenicillin resistance gene; HygR/ aph(IV): hyromycin resistance/ aminoglycoside phosphotransferase IV gene.

(b) Sequence of genomic DNA carrying the *CDKF;1:GFPPIPL* gene in the EcoRI-BamHI sites of pGAPKm and pGAPHyg vectors, and the position of *cdkf;1* (GABI_315A10) T-DNA insertion mutation. Gene specific primers F1seq5 and F1seq3 flanking the stop codon (yellow) were used for verification of the presence of the *CDKF;1* gene in the BAC F25O24 (KmR) and monitoring insertion and replacement of the *CmR-araC-ccdB* insertion cassette. 50 nt flanks (F1_50ntF and F1_50ntR) upstream and downstream of the stop codon are marked in blue 5’ and 3’ of the GFPPIPL insertion (Figure S3a). Fragments representing the boundaries of genomic DNA designed for transfer into plant were PCR amplified with the primer pairs F1FLANK1F_EcoRI + F1FLANK13_SalI and F1FLANK2F_SalI+ F1FLANK1_BamHI, and then cloned into EcoRI-BamHI sites of pGAP binary vectors. Sequences of CDKF;1:CmR-araC-ccdB cassette insertion, *CDKF;1:GFP* and *CDKF;1:PIPL* constructs between the gene specific primers F1seq5 and F1seq3, as well as amino acid sequences of CDKF;1:GFPPIPL, CDKF;1-GFP and CDKF;1-PIPL proteins are shown below the sequence of *CDKF;1:GFPPIPL* recombineering construct. Abbreviations: CobW: segment of the Co^2+^/Ni^2+^-binding domain of Arabidopsis COBW-like protein (At1g15730) carrying 18 His residues; StrepII: Strep-Tactin binding peptide; HA: hemagglutinin epitope, GFP: green fluorescent protein.

(c) Sequence of genomic DNA region carrying the *CDKD;1:GFPPIPL* gene in the EcoRI-BamHI sites of pGAPKm and pGAPHyg vectors. The positions of D1seq5 and D1seq3 gene specific primers, D1_50ntF and D1_50ntR 50 nt flanks of stop codon, and D1FLANK1F_EcoRI + D1FLANK13_SalI and D1FLANK2F_SalI+ D1FLANK1_BamHI primer pairs used for cloning of flanking fragments of plant DNA segment transferred with the modified *CDKD;1* gene constructs into plants are marked as in (b). Note: the *CDKD;1* and *miR156a* genes are transcribed by a common bidirectional promoter, while the downstream gene AT1G73700 (MATE efflux family protein) has opposite polarity compared to *CDKD;1* and their 3’-UTRs do not overlap. Therefore, *miR159a* and a larger upstream region were included in the gene construct, whereas the 3’ flanking region was placed to the 5’ segment of downstream gene AT1G73700. Sequences of *CDKD;1:GFP* construct between the gene specific primers D1seq5 and D1seq3, as well as amino acid sequences of CDKD;1:GFPPIPL and CDKD;1:GFP proteins are shown below the sequence *CDKD;1:GFPPIPL* construct.

(d) Sequence of genomic DNA region carrying the *CDKD;2:GFPPIPL* gene in the EcoRI-BamHI sites of pGAPKm and pGAPHyg vectors. The positions of D2seq5 and D2seq3 gene specific primers, D250ntF and D250ntR 50 nt flanks of stop codon, and D2FLANK1F_EcoRI + D2FLANK13_SalI and D2FLANK2F_SalI+ D2FLANK1_BamHI primer pairs used for cloning of flanking fragments of plant DNA segment transferred with the modified *CDKD;2* gene constructs into plants are marked as in (b). Sequences of the *CDKD;2:GFP* construct between the gene specific primers D2seq5 and D2seq3, as well as amino acid sequences of CDKD;2:GFPPIPL and CDKD;2:GFP proteins are depicted below the *CDKD;2:GFPPIPL* construct.

(e) Sequence of genomic DNA carrying the *CDKD;3:GFPPIPL* gene in the EcoRI-BamHI sites of pGAPKm and pGAPHyg vectors. The positions of D3seq5 and D3seq3 gene specific primers, D3_50ntF and D3_50ntR 50 nt flanks of stop codon, and D3FLANK1F_EcoRI + D3FLANK13_SalI and D3FLANK2F_SalI+ D3FLANK1_BamHI primer pairs used for cloning of flanking fragments of plant DNA segment transferred with the modified *CDKD;3* gene constructs into plants are marked as in (b). Note: 3’-UTR of the *CDKD;3* gene overlaps with that of the downstream AT1G18030 PP2C8 protein phosphatase gene. Therefore, the downstream flank was designed such that it includes the AT1G18030 gene. The *CDKD;3* gene carried by the BAC T10F20 (CmR) was modified using the *KmR-araC-ccdB* cassette. Sequences of the CDKD;3:KmR-araC-ccdB cassette insertion and *CDKD;3:GFP* gene between the gene specific primers D3seq5 and D3seq3, as well as amino acid sequences of CDKD3:GFPPIPL and CDKD3:GFP proteins are shown below the *CDKD;2:GFPPIPL* construct.

(f) Sequence of genomic DNA carrying the *CYCH:mCherry* gene in the SpeI-BamHI sites of pGAPBRKm and pGAPBRHyg vectors. The positions of CYCHseq5 and CYCHseq3 gene specific primers, and CYCH_50ntF and CYCH_50ntR 50 nt flanks of the stop codon are indicated as in (b). The flanks of genomic DNA region transferred with *CYCH:mCherry* into plants are marked by the 50nt flanking primers CYC_Flank1 and CYCH-Flank2. Reverse complement of CYC-Flank1 is combined with that of the SpeIR primer sequence (Figure S5; Table S1), whereas CYCH-Flank2 is fused to 3’ sequences of the BamHIF primer of pGAPBRKm and pGAPBRHygR (Figure S5, Table S1) to PCR amplify the BamHI-linearized Agrobacterium binary vectors for gap repair. Note: Application of Q5 enhancer (NEB) is necessary for PCR amplification of the vectors. Sequences of the C-GFPstop-SpR cassette insertion and *CYCH:GFP* construct between the gene specific primers CYCHseq5 and CYCHseq3, as well as amino acid sequences of CYCH:mCherry and CYCH:GFP proteins are shown below the *CYCH:mCherry* gene.

(g) Sequence of genomic DNA carrying the *HISTONE H3.1:mCherry* gene in the SpeI-BamHI sites of pGAPBRKm and pGAPBRHyg vectors. The positions of H3.1seq5 and H3.1seq3 gene specific primers, H3.1_50ntF and H3.1_50ntR 50 nt flanks of stop codon, and H3.1_Flank1 and H3.1_Flank2 primers are indicated as in (f). Note: the HISTONE H3.1 gene (AT5G65360) and its downstream neighbor AT5G65350 encoding HISTONE 3-like 5 (H3.5) overlap, so it is not clear where the promoter region of the latter gene is located. Therefore, AT5G65350 was retained in the H3.1 gene constructs. Sequence of the *HISTONE H3.1:GFP* construct between the gene specific primers H3.1seq5 and H3.1seq3, as well as amino acid sequences of H3.1:mCherry and H3.1:GFP proteins are depicted below the *HISTONE H3.1:mCherry* construct.
