# Supplementary material for: Gene modification by fast‐track recombineering for cellular localization and isolation of components of plant protein complexes
Source: Plant J. 2019 Jul 26;100(2):411–29. doi: 10.1111/tpj.14450 (PMC6852550; doi:10.1111/tpj.14450)
Supplement: Supplementary file 3 — Figure S3. Nucleotide and amino acid sequences of GFP–PIPL and PIPL–GFP tags. [file TPJ-100-411-s003.docx]

**(a)**

**GFPPIPL cassette**

GAATTC**ATGGTGAGCAAGGGCGAGGAG**CTGTTCACCGGGGTGGTGCCCATCCTGGTCGAG 60 **GFPF**

CTGGACGGCGACGTAAACGGCCACAAGTTCAGCGTGTCCGGCGAGGGCGAGGGCGATGCC 120

ACCTACGGCAAGCTGACCCTGAAGTTCATCTGCACCACCGGCAAGCTGCCCGTGCCCTGG 180

CCCACCCTCGTGACCACCCTGACCTACGGCGTGCAGTGCTTCAGCCGCTACCCCGACCAC 240

ATGAAGCAGCACGACTTCTTCAAGTCCGCCATGCCCGAAGGCTACGTCCAGGAGCGCACC 300

ATCTTCTTCAAGGACGACGGCAACTACAAGACCCGCGCCGAGGTGAAGTTCGAGGGCGAC 360 GFP

ACCCTGGTGAACCGCATCGAGCTGAAGGGCATCGACTTCAAGGAGGACGGCAACATCCTG 420

GGGCACAAGCTGGAGTACAACTACAACAGCCACAACGTCTATATCATGGCCGACAAGCAG 480

AAGAACGGCATCAAGGTGAACTTCAAGATCCGCCACAACATCGAGGACGGCAGCGTGCAG 540

CTCGCCGACCACTACCAGCAGAACACCCCCATCGGCGACGGCCCCGTGCTGCTGCCCGAC 600

AACCACTACCTGAGCACCCAGTCCGCCCTGAGCAAAGACCCCAACGAGAAGCGCGATCAC 660 **GFPR**

ATGGTCCTGCTGGAGTTCGTGACCGCCGCCGGGATCACTCTCGGCATGGACGAGCTGTAC 720 PIPL:

AAGGTCGAC**ATGGGTCATGATGATCATCACCATGG**TCATGACTGCCATGATCACCACAAT 780 CobW, **PIPLF**

GAGCATGAGCATGAGCATGAACACGAGCATCACCATTCTCATGATCACACCCATGACTGG 840 StrepII

TCTCATCCTCAGTTCGAAAAAGGAGGTGGATCTGGTGGAGGTTCTGGAGGTGGATGGTCT 900 StrepII

CACCCACAATTTGAGAAGGGAT**CTTATCCATACGATGTTCCAGATTATGCTTGA**GAGCTC 960 HA,**PIPLR**

MVSKGEELFTGVVPILVELDGDVNGHKFSVSGEGEGDATYGKLTLKFICTTGKLPVPWPT 60

LVTTLTYGVQCFSRYPDHMKQHDFFKSAMPEGYVQERTIFFKDDGNYKTRAEVKFEGDTL 120

VNRIELKGIDFKEDGNILGHKLEYNYNSHNVYIMADKQKNGIKVNFKIRHNIEDGSVQLA 180

DHYQQNTPIGDGPVLLPDNHYLSTQSALSKDPNEKRDHMVLLEFVTAAGITLGMDELYKV 240

DMGHDDHHHGHDCHDHHNEHEHEHEHEHHHSHDHTHDWSHPQFEKGGGSGGGSGGGWSHP 300

QFEKGSYPYDVPDYA. 316

**(b)**

**PIPLGFP cassette**

GAATTC**ATGGGTCATGATGATCATCACCATGG**TCATGACTGCCATGATCACCACAATGAG 60 PIPL, **PIPLF**

CATGAGCATGAGCATGAACACGAGCATCACCATTCTCATGATCACACCCATGACTGGTCT 120 StrepII

CATCCTCAGTTCGAAAAAGGAGGTGGATCTGGTGGAGGTTCTGGAGGTGGATGGTCTCAC 180 StrepII

CCACAATTTGAGAAGGGAT**CTTATCCATACGATGTTCCAGATTATGCT**GTCGAC**ATG**GTG 240 HA,**PIPLR/GFPF**

AGCAAGGGCGAGGAGCTGTTCACCGGGGTGGTGCCCATCCTGGTCGAGCTGGACGGCGAC 300

GTAAACGGCCACAAGTTCAGCGTGTCCGGCGAGGGCGAGGGCGATGCCACCTACGGCAAG 360

CTGACCCTGAAGTTCATCTGCACCACCGGCAAGCTGCCCGTGCCCTGGCCCACCCTCGTG 420

ACCACCCTGACCTACGGCGTGCAGTGCTTCAGCCGCTACCCCGACCACATGAAGCAGCAC 480

GACTTCTTCAAGTCCGCCATGCCCGAAGGCTACGTCCAGGAGCGCACCATCTTCTTCAAG 540 GFP

GACGACGGCAACTACAAGACCCGCGCCGAGGTGAAGTTCGAGGGCGACACCCTGGTGAAC 600

CGCATCGAGCTGAAGGGCATCGACTTCAAGGAGGACGGCAACATCCTGGGGCACAAGCTG 660

GAGTACAACTACAACAGCCACAACGTCTATATCATGGCCGACAAGCAGAAGAACGGCATC 720

AAGGTGAACTTCAAGATCCGCCACAACATCGAGGACGGCAGCGTGCAGCTCGCCGACCAC 780

TACCAGCAGAACACCCCCATCGGCGACGGCCCCGTGCTGCTGCCCGACAACCACTACCTG 840

AGCACCCAGTCCGCCCTGAGCAAAGACCCCAACGAGAAGCGCGATCACATGGTCCTGCTG 900

GAGTTCGTGACCGCCGCCGGGATCACTC**TCGGCATGGACGAGCTGTACAAG**GAGCTC 957 **GFPR**

MGHDDHHHGHDCHDHHNEHEHEHEHEHHHSHDHTHDWSHPQFEKGGGSGGGSGGGWSHPQ 60

FEKGSYPYDVPDYAVDMVSKGEELFTGVVPILVELDGDVNGHKFSVSGEGEGDATYGKLT 120

LKFICTTGKLPVPWPTLVTTLTYGVQCFSRYPDHMKQHDFFKSAMPEGYVQERTIFFKDD 180

GNYKTRAEVKFEGDTLVNRIELKGIDFKEDGNILGHKLEYNYNSHNVYIMADKQKNGIKV 240

NFKIRHNIEDGSVQLADHYQQNTPIGDGPVLLPDNHYLSTQSALSKDPNEKRDHMVLLEF 300

VTAAGITLGMDELYK 315

**Figure S3.** Nucleotide and amino acid sequences of GFPPIPL and PIPLGFP tags.

(a) Sequence of the GFPPIPL tag and corresponding protein. For C-terminal fusion, the GFP-PIPL tag is PCR amplified with the GFPF and PIPLR primers. For N-terminal fusion of GFPPIPL tag, the stop codon must be removed from the PIPLR PCR primer, whereas GFP is amplified with the GFPF and GFPR primers.

(b) Sequence of the PIPLGFP tag and corresponding protein. For N-terminal fusion, the PIPLGFP tag is PCR amplified with the PIPLF and GFPR primers, and GFP is amplified with the GFPF and GFPR primers. For C-terminal fusions stop codons should be added to the PIPLR and GFPR PCR primers.
