# Supplementary material for: Gene modification by fast‐track recombineering for cellular localization and isolation of components of plant protein complexes
Source: Plant J. 2019 Jul 26;100(2):411–29. doi: 10.1111/tpj.14450 (PMC6852550; doi:10.1111/tpj.14450)
Supplement: Supplementary file 4 — Figure S4. Nucleotide sequences of N‐terminal and C‐terminal I‐SceI insertion cassettes. [file TPJ-100-411-s004.docx]

**N-terminal insertion cassettes**

**(a) N-KmR-GFP-PIPL / N-KmR-GFP**

BamHI I-SceI

ggatcc**CTATATTACCCTGTTATCCCTAGCGTA**gccgccatgaccgtcccgtcaagtcag 60 **N-KmRI-SceIF**

cgtaatgctctgccagtgttacaaccaattaaccaattctga**TTA**GAAAAACTCATCGAG 120

CATCAAATGAAACTGCAATTTATTCATATCAGGATTATCAATACCATATTTTTGAAAAAG 180

CCGTTTCTGTAATGAAGGAGAAAACTCACCGAGGCAGTTCCATAGGATGGCAAGATCCTG 240

GTATCGGTCTGCGATTCCGACTCGTCCAACATCAATACAACCTATTAATTTCCCCTCGTC 300

AAAAATAAGGTTATCAAGTGAGAAATCACCATGAGTGACGACTGAATCCGGTGAGAATGG 360

CAAAAGCTTATGCATTTCTTTCCAGACTTGTTCAACAGGCCAGCCATTACGCTCGTCATC 420

AAAATCACTCGCATCAACCAAACCGTTATTCATTCGTGATTGCGCCTGAGCGAGACGAAA 480

TACGCGATCGCTGTTAAAAGGACAATTACAAACAGGAATCGAATGCAACCGGCGCAGGAA 540 KmR

CACTGCCAGCGCATCAACAATATTTTCACCTGAATCAGGATATTCTTCTAATACCTGGAA 600

TGCTGTTTTCCCGGGGATCGCAGTGGTGAGTAACCATGCATCATCAGGAGTACGGATAAA 660

ATGCTTGATGGTCGGAAGAGGCATAAATTCCGTCAGCCAGTTTAGTCTGACCATCTCATC 720

TATAACATCATTGGCAACGCTACCTTTGCCATGTTTCAGAAACAACTCTGGCGCATCGGG 780

CTTCCCATACAATCGATAGATTGTCGCACCTGATTGCCCGACATTATCGCGAGCCCATTT 840

ATACCCATATAAATCAGCATCCATGTTGGAATTTAATCGCGGCCTCGAGCAAGACGTTTC 900

CCGTTGAATATGGCT**CAT**aacaccccttgtattactgtttatgtaagcagacagttttat 960

tgttgatgatgatatatttttatcttgtgcaatgtaacatcagagattttgagacacaac 1020

gtggctcatggcggccgcgggaattcgatatcactagagccgtcaattgtctgattcgtt 1080

acc**ACTAGTCTATATTACCCTGTTATCCCTAGCGTAATG**GTGAGCAAGGGCGAGGAGCTG 1140 **SpeI**/**I-SceI**

TTCACCGGGGTGGTGCCCATCCTGGTCGAGCTGGACGGCGACGTAAACGGCCACAAGTTC 1200

AGCGTGTCCGGCGAGGGCGAGGGCGATGCCACCTACGGCAAGCTGACCCTGAAGTtCATC 1260

TGCACCACCGGCAAGCTGCCCGTGCCCTGGCCCACCCTCGTGACCACCcTGACcTACGGC 1320

GTGCAGTGCTTCAGCCGCTACCCCGACCACATGAAGCAGCACGACTTCTTCAAGTCCGCC 1380

ATGCCCGAAGGCTACGTCCAGGAGCGCACCATCTTCTTCAAGGACGACGGCAACTACAAG 1440 GFP

ACCCGCGCCGAGGTGAAGTTCGAGGGCGACACCCTGGTGAACCGCATCGAGCTGAAGGGC 1500

ATCGACTTCAAGGAGGACGGCAACATCCTGGGGCACAAGCTGGAGTACAACTACAACAGC 1560

CACAACGTCTATATCATGGCCGACAAGCAGAAGAACGGCATCAAGGTGAACTTCAAGATC 1620

CGCCACAACATCGAGGACGGCAGCGTGCAGCTCGCCGACCACTACCAGCAGAACACCCCC 1680

ATCGGCGACGGCCCCGTGCTGCTGCCCGACAACCACTACCTGAGCACCCAGTCCGCCCTG 1740

AGCAAAGACCCCAACGAGAAGCGCGATCACATGGTCCTGCTGGAGTTCGTGACCGCCGCC 1800

GGGATCACTC**TCGGCATGGACGAGCTGTACAAG**GTCGAC**ATG**GGTCATGATGATCATCAC 1860 **GFPRnostop**

CATGGTCATGACTGCCATGATCACCACAATGAGCATGAGCATGAGCATGAACACGAGCAT 1920 CobW 18 His

CACCATTCTCATGATCACACCCATGACTGGTCTCATCCTCAGTTCGAAAAAGGAGGTGGA 1980 StrepII

TCTGGTGGAGGTTCTGGAGGTGGATGGTCTCACCCACAATTTGAGAAGGGA**TCTTATCCA** 2040 HA

**TACGATGTTCCAGATTATGCT**ggtacc 2067 **PIPLR**

KpnI

**(b) N-KmR-mCherry**

BamHI I-SceI

ggatcc**CTATATTACCCTGTTATCCCTAGCGTA**gccgccatgaccgtcccgtcaagtcag 60 **N-KmRI-SceIF**

cgtaatgctctgccagtgttacaaccaattaaccaattctga**TTA**GAAAAACTCATCGAG 120

CATCAAATGAAACTGCAATTTATTCATATCAGGATTATCAATACCATATTTTTGAAAAAG 180

CCGTTTCTGTAATGAAGGAGAAAACTCACCGAGGCAGTTCCATAGGATGGCAAGATCCTG 240

GTATCGGTCTGCGATTCCGACTCGTCCAACATCAATACAACCTATTAATTTCCCCTCGTC 300

AAAAATAAGGTTATCAAGTGAGAAATCACCATGAGTGACGACTGAATCCGGTGAGAATGG 360

CAAAAGCTTATGCATTTCTTTCCAGACTTGTTCAACAGGCCAGCCATTACGCTCGTCATC 420

AAAATCACTCGCATCAACCAAACCGTTATTCATTCGTGATTGCGCCTGAGCGAGACGAAA 480 KmR

TACGCGATCGCTGTTAAAAGGACAATTACAAACAGGAATCGAATGCAACCGGCGCAGGAA 540

CACTGCCAGCGCATCAACAATATTTTCACCTGAATCAGGATATTCTTCTAATACCTGGAA 600

TGCTGTTTTCCCGGGGATCGCAGTGGTGAGTAACCATGCATCATCAGGAGTACGGATAAA 660

ATGCTTGATGGTCGGAAGAGGCATAAATTCCGTCAGCCAGTTTAGTCTGACCATCTCATC 720

TGTAACATCATTGGCAACGCTACCTTTGCCATGTTTCAGAAACAACTCTGGCGCATCGGG 780

CTTCCCATACAATCGATAGATTGTCGCACCTGATTGCCCGACATTATCGCGAGCCCATTT 840

ATACCCATATAAATCAGCATCCATGTTGGAATTTAATCGCGGCCTCGAGCAAGACGTTTC 900

CCGTTGAATATGGCT**CAT**aacaccccttgtattactgtttatgtaagcagacagttttat 960

tgttcatggcggccgcgggaattcgatatcactagagccgtcaattgtctgattcgttac 1020

c**ACTAGTCTATATTACCCTGTTATCCCTAGCGTAATG**GTGAGCAAGGGCGAGGAGGATAA 1080 **SpeI**/**I-SceI**

CATGGCCATCATCAAGGAGTTCATGCGCTTCAAGGTGCACATGGAGGGCTCCGTGAACGG 1140

CCACGAGTTCGAGATCGAGGGCGAGGGCGAGGGCCGCCCCTACGAGGGCACCCAGACCGC 1200

CAAGCTGAAGGTGACCAAGGGTGGCCCCCTGCCCTTCGCCTGGGACATCCTGTCCCCTCA 1260

GTTCATGTACGGCTCCAAGGCCTACGTGAAGCACCCCGCCGACATCCCCGACTACTTGAA 1320

GCTGTCCTTCCCCGAGGGCTTCAAGTGGGAGCGCGTGATGAACTTCGAGGACGGCGGCGT 1380 mCherry

GGTGACCGTGACCCAGGACTCCTCCCTGCAGGACGGCGAGTTCATCTACAAGGTGAAGCT 1440

GCGCGGCACCAACTTCCCCTCCGACGGCCCCGTAATGCAGAAGAAGACCATGGGCTGGGA 1500

GGCCTCCTCCGAGCGGATGTACCCCGAGGACGGCGCCCTGAAGGGCGAGATCAAGCAGAG 1560

GCTGAAGCTGAAGGACGGCGGCCACTACGACGCTGAGGTCAAGACCACCTACAAGGCCAA 1620

GAAGCCCGTGCAGCTGCCCGGCGCCTACAACGTCAACATCAAGTTGGACATCACCTCCCA 1680

CAACGAGGACTACACCATCGTGGAACAGTACGAACGCGCCGAGGGCCGCCACTCCACCG**G** 1740

**CGGCATGGACGAGCTGTACAAG**ggtacc 1768 **mCherrynostop**

KpnI

**(c) N-SpR-GFP-PIPL / N-SpR-GFP**

BamHI I-SceI

ggatcc**CTATATTACCCTGTTATCCCTAGCGTACtgtgactcat**gttaccgatgctattc 60 **N-SpRI-SceIF** ggaagaacggcaactaagctgccgggtttgaaacacggatgatctcgcggagggtagcat 120

gttgattgtaacgatgacagagcgttgctgcctgtgatcaattcgggcacgaacccagtg 180

gacataagcctgttcggttcgtaagctgtaatgcaagtagcgtaactgccgtcacgcaac 240

tggtccagaaccttgaccgaacgcagcggtggtaacggcgcagtggcggttttcatggct 300

tgttATGACTGTTTTTTTGGGGTACAGTCT**ATG**CCTCGGGCATCCAAGCAGCAAGCGCGT 360

TACGCCGTGGGTCGATGTTTGATGTTATGGAGCAGCAACGATGTTACGCAGCAGGGCAGT 420

CGCCCTAAAACAAAGTTAAACATCATGGGGGAAGCGGTGATCGCCGAAGTATCGACTCAA 480

CTATCAGAGGTAGTTGGCGTCATCGAGCGCCATCTCGAACCGACGTTGCTGGCCGTACAT 540

TTGTACGGCTCCGCAGTGGATGGCGGCCTGAAGCCACACAGTGATATTGATTTGCTGGTT 600

ACGGTGACCGTAAGGCTTGATGAAACAACGCGGCGAGCTTTGATCAACGACCTTTTGGAA 660

ACTTCGGCTTCCCCTGGAGAGAGCGAGATTCTCCGCGCTGTAGAAGTCACCATTGTTGTG 720 SpR

CACGACGACATCATTCCGTGGCGTTATCCAGCTAAGCGCGAACTGCAATTTGGAGAATGG 780

CAGCGCAATGACATTCTTGCAGGTATCTTCGAGCCAGCCACGATCGACATTGATCTGGCT 840

ATCTTGCTGACAAAAGCAAGAGAACATAGCGTTGCCTTGGTAGGTCCAGCGGCGGAGGAA 900

CTCTTTGATCCGGTTCCTGAACAGGATCTATTTGAGGCGCTAAATGAAACCTTAACGCTA 960

TGGAACTCGCCGCCCGACTGGGCTGGCGATGAGCGAAATGTAGTGCTTACGTTGTCCCGC 1020

ATTTGGTACAGCGCAGTAACCGGCAAAATCGCGCCGAAGGATGTCGCTGCCGACTGGGCA 1080

ATGGAGCGCCTGCCGGCCCAGTATCAGCCCGTCATACTTGAAGCTAGACAGGCTTATCTT 1140

GGACAAGAAGAAGATCGCTTGGCCTCGCGCGCAGATCAGTTGGAAGAATTTGTCCACTAC 1200

GTGAAAGGCGAGATCACCAAGGTAGTCGGCAAA**TAA**tgtctagctagaaattcgttcaag 1260

ccgacgccgcttcgccgg**actagtCTATATTACCCTGTTATCCCTAGCGTAATG**GTGAGC 1320 **SpeI**/**I-SceI**

AAGGGCGAGGAGCTGTTCACCGGGGTGGTGCCCATCCTGGTCGAGCTGGACGGCGACGTA 1380

AACGGCCACAAGTTCAGCGTGTCCGGCGAGGGCGAGGGCGATGCCACCTACGGCAAGCTG 1440

ACCCTGAAGTtCATCTGCACCACCGGCAAGCTGCCCGTGCCCTGGCCCACCCTCGTGACC 1500 GFP

ACCcTGACcTACGGCGTGCAGTGCTTCAGCCGCTACCCCGACCACATGAAGCAGCACGAC 1560

TTCTTCAAGTCCGCCATGCCCGAAGGCTACGTCCAGGAGCGCACCATCTTCTTCAAGGAC 1620

GACGGCAACTACAAGACCCGCGCCGAGGTGAAGTTCGAGGGCGACACCCTGGTGAACCGC 1680

ATCGAGCTGAAGGGCATCGACTTCAAGGAGGACGGCAACATCCTGGGGCACAAGCTGGAG 1740

TACAACTACAACAGCCACAACGTCTATATCATGGCCGACAAGCAGAAGAACGGCATCAAG 1800

GTGAACTTCAAGATCCGCCACAACATCGAGGACGGCAGCGTGCAGCTCGCCGACCACTAC 1860

CAGCAGAACACCCCCATCGGCGACGGCCCCGTGCTGCTGCCCGACAACCACTACCTGAGC 1920

ACCCAGTCCGCCCTGAGCAAAGACCCCAACGAGAAGCGCGATCACATGGTCCTGCTGGAG 1980

TTCGTGACCGCCGCCGGGATCACTC**TCGGCATGGACGAGCTGTACAAG**GTCGAC**ATG**GGT 2040 **GFPRnostop**

CATGATGATCATCACCATGGTCATGACTGCCATGATCACCACAATGAGCATGAGCATGAG 2100 CobW 18 His

CATGAACACGAGCATCACCATTCTCATGATCACACCCATGACTGGTCTCATCCTCAGTTC 2160 StrepII

GAAAAAGGAGGTGGATCTGGTGGAGGTTCTGGAGGTGGATGGTCTCACCCACAATTTGAG 2220

AAGGGATCT**TATCCATACGATGTTCCAGATTATGCT**ggtacc 2262 HA, **PIPLR**

KpnI

**(d) N-SpR-mCherry**

BamHI I-SceI

**ggatccCTATATTACCCTGTTATCCCTAGCGTACtgtgactcat**gttaccgatgctattc 60 **N-SpRI-SceIF**

ggaagaacggcaactaagctgccgggtttgaaacacggatgatctcgcggagggtagcat 120

gttgattgtaacgatgacagagcgttgctgcctgtgatcaattcgggcacgaacccagtg 180

gacataagcctgttcggttcgtaagctgtaatgcaagtagcgtaactgccgtcacgcaac 240

tggtccagaaccttgaccgaacgcagcggtggtaacggcgcagtggcggttttcatggct 300

tgttATGACTGTTTTTTTGGGGTACAGTCT**ATG**CCTCGGGCATCCAAGCAGCAAGCGCGT 360

TACGCCGTGGGTCGATGTTTGATGTTATGGAGCAGCAACGATGTTACGCAGCAGGGCAGT 420

CGCCCTAAAACAAAGTTAAACATCATGGGGGAAGCGGTGATCGCCGAAGTATCGACTCAA 480

CTATCAGAGGTAGTTGGCGTCATCGAGCGCCATCTCGAACCGACGTTGCTGGCCGTACAT 540

TTGTACGGCTCCGCAGTGGATGGCGGCCTGAAGCCACACAGTGATATTGATTTGCTGGTT 600

ACGGTGACCGTAAGGCTTGATGAAACAACGCGGCGAGCTTTGATCAACGACCTTTTGGAA 660

ACTTCGGCTTCCCCTGGAGAGAGCGAGATTCTCCGCGCTGTAGAAGTCACCATTGTTGTG 720 SpR

CACGACGACATCATTCCGTGGCGTTATCCAGCTAAGCGCGAACTGCAATTTGGAGAATGG 780

CAGCGCAATGACATTCTTGCAGGTATCTTCGAGCCAGCCACGATCGACATTGATCTGGCT 840

ATCTTGCTGACAAAAGCAAGAGAACATAGCGTTGCCTTGGTAGGTCCAGCGGCGGAGGAA 900

CTCTTTGATCCGGTTCCTGAACAGGATCTATTTGAGGCGCTAAATGAAACCTTAACGCTA 960

TGGAACTCGCCGCCCGACTGGGCTGGCGATGAGCGAAATGTAGTGCTTACGTTGTCCCGC 1020

ATTTGGTACAGCGCAGTAACCGGCAAAATCGCGCCGAAGGATGTCGCTGCCGACTGGGCA 1080

ATGGAGCGCCTGCCGGCCCAGTATCAGCCCGTCATACTTGAAGCTAGACAGGCTTATCTT 1140

GGACAAGAAGAAGATCGCTTGGCCTCGCGCGCAGATCAGTTGGAAGAATTTGTCCACTAC 1200

GTGAAAGGCGAGATCACCAAGGTAGTCGGCAAA**TAA**tgtctagctagaaattcgttcaag 1260

ccgacgccgcttcgccgg**actagtCTATATTACCCTGTTATCCCTAGCGTAATG**GTGAGC 1320 **SpeI**/**I-SceI**

AAGGGCGAGGAGGATAACATGGCCATCATCAAGGAGTTCATGCGCTTCAAGGTGCACATG 1380

GAGGGCTCCGTGAACGGCCACGAGTTCGAGATCGAGGGCGAGGGCGAGGGCCGCCCCTAC 1440

GAGGGCACCCAGACCGCCAAGCTGAAGGTGACCAAGGGTGGCCCCCTGCCCTTCGCCTGG 1500

GACATCCTGTCCCCTCAGTTCATGTACGGCTCCAAGGCCTACGTGAAGCACCCCGCCGAC 1560

ATCCCCGACTACTTGAAGCTGTCCTTCCCCGAGGGCTTCAAGTGGGAGCGCGTGATGAAC 1620 mCherry

TTCGAGGACGGCGGCGTGGTGACCGTGACCCAGGACTCCTCCCTGCAGGACGGCGAGTTC 1680

ATCTACAAGGTGAAGCTGCGCGGCACCAACTTCCCCTCCGACGGCCCCGTAATGCAGAAG 1740

AAGACCATGGGCTGGGAGGCCTCCTCCGAGCGGATGTACCCCGAGGACGGCGCCCTGAAG 1800

GGCGAGATCAAGCAGAGGCTGAAGCTGAAGGACGGCGGCCACTACGACGCTGAGGTCAAG 1860

ACCACCTACAAGGCCAAGAAGCCCGTGCAGCTGCCCGGCGCCTACAACGTCAACATCAAG 1920

TTGGACATCACCTCCCACAACGAGGACTACACCATCGTGGAACAGTACGAACGCGCCGAG 1980

GGCCGCCACTCCACCG**GCGGCATGGACGAGCTGTACAAG**ggtacc 2025 **mCherrynostop**

KpnI

**C-terminal insertion cassettes**

**(e) C-GFPstop-KmR**

BamHI

GGATCC**ATGGTGAGCAAGGGCGAGGAG**CTGTTCACCGGGGTGGTGCCCATCCTGGTCGAG 60 **GFPF**

CTGGACGGCGACGTAAACGGCCACAAGTTCAGCGTGTCCGGCGAGGGCGAGGGCGATGCC 120

ACCTACGGCAAGCTGACCCTGAAGTTCATCTGCACCACCGGCAAGCTGCCCGTGCCCTGG 180

CCCACCCTCGTGACCACCCTGACCTACGGCGTGCAGTGCTTCAGCCGCTACCCCGACCAC 240

ATGAAGCAGCACGACTTCTTCAAGTCCGCCATGCCCGAAGGCTACGTCCAGGAGCGCACC 300

ATCTTCTTCAAGGACGACGGCAACTACAAGACCCGCGCCGAGGTGAAGTTCGAGGGCGAC 360 GFP

ACCCTGGTGAACCGCATCGAGCTGAAGGGCATCGACTTCAAGGAGGACGGCAACATCCTG 420

GGGCACAAGCTGGAGTACAACTACAACAGCCACAACGTCTATATCATGGCCGACAAGCAG 480

AAGAACGGCATCAAGGTGAACTTCAAGATCCGCCACAACATCGAGGACGGCAGCGTGCAG 540

CTCGCCGACCACTACCAGCAGAACACCCCCATCGGCGACGGCCCCGTGCTGCTGCCCGAC 600

AACCACTACCTGAGCACCCAGTCCGCCCTGAGCAAAGACCCCAACGAGAAGCGCGATCAC 660

ATGGTCCTGCTGGAGTTCGTGACCGCCGCCGGGATCACTCTCGGCATGGACGAGCTGTAC 720

AAG**TGACTATATTACCCTGTTATCCCTAGCGTAactagt**gccgccatgaccgtcccgtca 780 **I-SceI/SpeI**

agtcagcgtaatgctctgccagtgttacaaccaattaaccaattctga**TTA**GAAAAACTC 840

ATCGAGCATCAAATGAAACTGCAATTTATTCATATCAGGATTATCAATACCATATTTTTG 900

AAAAAGCCGTTTCTGTAATGAAGGAGAAAACTCACCGAGGCAGTTCCATAGGATGGCAAG 960

ATCCTGGTATCGGTCTGCGATTCCGACTCGTCCAACATCAATACAACCTATTAATTTCCC 1020

CTCGTCAAAAATAAGGTTATCAAGTGAGAAATCACCATGAGTGACGACTGAATCCGGTGA 1080

GAATGGCAAAAGCTTATGCATTTCTTTCCAGACTTGTTCAACAGGCCAGCCATTACGCTC 1140

GTCATCAAAATCACTCGCATCAACCAAACCGTTATTCATTCGTGATTGCGCCTGAGCGAG 1200

ACGAAATACGCGATCGCTGTTAAAAGGACAATTACAAACAGGAATCGAATGCAACCGGCG 1260 KmR

CAGGAACACTGCCAGCGCATCAACAATATTTTCACCTGAATCAGGATATTCTTCTAATAC 1320

CTGGAATGCTGTTTTCCCGGGGATCGCAGTGGTGAGTAACCATGCATCATCAGGAGTACG 1380

GATAAAATGCTTGATGGTCGGAAGAGGCATAAATTCCGTCAGCCAGTTTAGTCTGACCAT 1440

CTCATCT**A**TAACATCATTGGCAACGCTACCTTTGCCATGTTTCAGAAACAACTCTGGCGC 1500

ATCGGGCTTCCCATACAATCGATAGATTGTCGCACCTGATTGCCCGACATTATCGCGAGC 1560

CCATTTATACCCATATAAATCAGCATCCATGTTGGAATTTAATCGCGGCCTCGAGCAAGA 1620

CGTTTCCCGTTGAATATGGCT**CAT**aacaccccttgtattactgtttatgtaagcagacag 1680

ttttattgttgatgatgatatatttttatcttgtgcaatgtaacatcagagattttgaga 1740

cacaacgtggctcatggcggccgcgggaattcgatatcactagagccgtcaattgtctga 1800

ttc**GTTACCCTATATTACCCTGTTATCCCTAGCGTA**GGTACC 1842 **C-KmRI-SceIR**

I-SceI KpnI

**(f) C-GFP-PIPLstop-KmR / C-PIPLstop-KmR**

BamHI

GGATCC**ATGGTGAGCAAGGGCGAGGAG**CTGTTCACCGGGGTGGTGCCCATCCTGGTCGAG 60 **GFPF**

CTGGACGGCGACGTAAACGGCCACAAGTTCAGCGTGTCCGGCGAGGGCGAGGGCGATGCC 120

ACCTACGGCAAGCTGACCCTGAAGTTCATCTGCACCACCGGCAAGCTGCCCGTGCCCTGG 180

CCCACCCTCGTGACCACCCTGACCTACGGCGTGCAGTGCTTCAGCCGCTACCCCGACCAC 240

ATGAAGCAGCACGACTTCTTCAAGTCCGCCATGCCCGAAGGCTACGTCCAGGAGCGCACC 300

ATCTTCTTCAAGGACGACGGCAACTACAAGACCCGCGCCGAGGTGAAGTTCGAGGGCGAC 360 **GFP**

ACCCTGGTGAACCGCATCGAGCTGAAGGGCATCGACTTCAAGGAGGACGGCAACATCCTG 420

GGGCACAAGCTGGAGTACAACTACAACAGCCACAACGTCTATATCATGGCCGACAAGCAG 480

AAGAACGGCATCAAGGTGAACTTCAAGATCCGCCACAACATCGAGGACGGCAGCGTGCAG 540

CTCGCCGACCACTACCAGCAGAACACCCCCATCGGCGACGGCCCCGTGCTGCTGCCCGAC 600

AACCACTACCTGAGCACCCAGTCCGCCCTGAGCAAAGACCCCAACGAGAAGCGCGATCAC 660

ATGGTCCTGCTGGAGTTCGTGACCGCCGCCGGGATCACTCTCGGCATGGACGAGCTGTAC 720

AAGGTCGAC**ATGGGTCATGATGATCATCACCATGG**TCATGACTGCCATGATCACCACAAT 780 CobW 18 His, **PIPLF**

GAGCATGAGCATGAGCATGAACACGAGCATCACCATTCTCATGATCACACCCATGACTGG 840 **StrepII**

TCTCATCCTCAGTTCGAAAAAGGAGGTGGATCTGGTGGAGGTTCTGGAGGTGGATGGTCT 900

CACCCACAATTTGAGAAGGGATCTTATCCATACGATGTTCCAGATTATGCT**TGACTATAT** 960 **HA/I-SceI**

**TACCCTGTTATCCCTAGCGTA**ACTAGTGCCGCCATGACCGTCCCGTCAAGTCAGCGTAAT 1020

GCTCTGCCAGTGTTACAACCAATTAACCAATTCTGA**TTA**GAAAAACTCATCGAGCATCAA 1080

ATGAAACTGCAATTTATTCATATCAGGATTATCAATACCATATTTTTGAAAAAGCCGTTT 1140

CTGTAATGAAGGAGAAAACTCACCGAGGCAGTTCCATAGGATGGCAAGATCCTGGTATCG 1200

GTCTGCGATTCCGACTCGTCCAACATCAATACAACCTATTAATTTCCCCTCGTCAAAAAT 1260

AAGGTTATCAAGTGAGAAATCACCATGAGTGACGACTGAATCCGGTGAGAATGGCAAAAG 1320

CTTATGCATTTCTTTCCAGACTTGTTCAACAGGCCAGCCATTACGCTCGTCATCAAAATC 1380

ACTCGCATCAACCAAACCGTTATTCATTCGTGATTGCGCCTGAGCGAGACGAAATACGCG 1440 KmR

ATCGCTGTTAAAAGGACAATTACAAACAGGAATCGAATGCAACCGGCGCAGGAACACTGC 1500

CAGCGCATCAACAATATTTTCACCTGAATCAGGATATTCTTCTAATACCTGGAATGCTGT 1560

TTTCCCGGGGATCGCAGTGGTGAGTAACCATGCATCATCAGGAGTACGGATAAAATGCTT 1620

GATGGTCGGAAGAGGCATAAATTCCGTCAGCCAGTTTAGTCTGACCATCTCATCTATAAC 1680

ATCATTGGCAACGCTACCTTTGCCATGTTTCAGAAACAACTCTGGCGCATCGGGCTTCCC 1740

ATACAATCGATAGATTGTCGCACCTGATTGCCCGACATTATCGCGAGCCCATTTATACCC 1800

ATATAAATCAGCATCCATGTTGGAATTTAATCGCGGCCTCGAGCAAGACGTTTCCCGTTG 1860

AATATGGCT**CAT**aacaccccttgtattactgtttatgtaagcagacagttttattgttga 1920

tgatgatatatttttatcttgtgcaatgtaacatcagagattttgagacacaacgtggct 1980

catggcggccgcgggaattcgatatcactagagccgtcaattgtctgattc**GTTACCCTA** 2040 **TATTACCCTGTTATCCCTAGCGTA**GGTACC 2070 **C-KmRI-SceIR**

I-SceI KpnI

**(g) C-mCherrystop-KmR**

BamHI

ggatcc**ATGGTGAGCAAGGGCGAGGAG**GATAACATGGCCATCATCAAGGAGTTCATGCGC 60 **GFPF**

TTCAAGGTGCACATGGAGGGCTCCGTGAACGGCCACGAGTTCGAGATCGAGGGCGAGGGC 120

GAGGGCCGCCCCTACGAGGGCACCCAGACCGCCAAGCTGAAGGTGACCAAGGGTGGCCCC 180

CTGCCCTTCGCCTGGGACATCCTGTCCCCTCAGTTCATGTACGGCTCCAAGGCCTACGTG 240

AAGCACCCCGCCGACATCCCCGACTACTTGAAGCTGTCCTTCCCCGAGGGCTTCAAGTGG 300

GAGCGCGTGATGAACTTCGAGGACGGCGGCGTGGTGACCGTGACCCAGGACTCCTCCCTG 360 mCherry

CAGGACGGCGAGTTCATCTACAAGGTGAAGCTGCGCGGCACCAACTTCCCCTCCGACGGC 420

CCCGTAATGCAGAAGAAGACCATGGGCTGGGAGGCCTCCTCCGAGCGGATGTACCCCGAG 480

GACGGCGCCCTGAAGGGCGAGATCAAGCAGAGGCTGAAGCTGAAGGACGGCGGCCACTAC 540

GACGCTGAGGTCAAGACCACCTACAAGGCCAAGAAGCCCGTGCAGCTGCCCGGCGCCTAC 600

AACGTCAACATCAAGTTGGACATCACCTCCCACAACGAGGACTACACCATCGTGGAACAG 660

TACGAACGCGCCGAGGGCCGCCACTCCACCGGCGGCATGGACGAGCTGTACAAG**TGACTA 720**

**TATTACCCTGTTATCCCTAGCGTAactagt**gccgccatgaccgtcccgtcaagtcagcgt 780 **I-SceI**/**SpeI**

aatgctctgccagtgttacaaccaattaaccaattctga**TTA**GAAAAACTCATCGAGCAT 840

CAAATGAAACTGCAATTTATTCATATCAGGATTATCAATACCATATTTTTGAAAAAGCCG 900

TTTCTGTAATGAAGGAGAAAACTCACCGAGGCAGTTCCATAGGATGGCAAGATCCTGGTA 960

TCGGTCTGCGATTCCGACTCGTCCAACATCAATACAACCTATTAATTTCCCCTCGTCAAA 1020

AATAAGGTTATCAAGTGAGAAATCACCATGAGTGACGACTGAATCCGGTGAGAATGGCAA 1080

AAGCTTATGCATTTCTTTCCAGACTTGTTCAACAGGCCAGCCATTACGCTCGTCATCAAA 1140

ATCACTCGCATCAACCAAACCGTTATTCATTCGTGATTGCGCCTGAGCGAGACGAAATAC 1200 KmR

GCGATCGCTGTTAAAAGGACAATTACAAACAGGAATCGAATGCAACCGGCGCAGGAACAC 1260

TGCCAGCGCATCAACAATATTTTCACCTGAATCAGGATATTCTTCTAATACCTGGAATGC 1320

TGTTTTCCCGGGGATCGCAGTGGTGAGTAACCATGCATCATCAGGAGTACGGATAAAATG 1380

CTTGATGGTCGGAAGAGGCATAAATTCCGTCAGCCAGTTTAGTCTGACCATCTCATCTGT 1440

AACATCATTGGCAACGCTACCTTTGCCATGTTTCAGAAACAACTCTGGCGCATCGGGCTT 1500

CCCATACAATCGATAGATTGTCGCACCTGATTGCCCGACATTATCGCGAGCCCATTTATA 1560

CCCATATAAATCAGCATCCATGTTGGAATTTAATCGCGGCCTCGAGCAAGACGTTTCCCG 1620

TTGAATATGGCT**CAT**aacaccccttgtattactgtttatgtaagcagacagttttattgt 1680

tcatggcggccgcgggaattcgatatcactagagccgtcaattgtctgattc**GTTACCCT** 1740

**ATATTACCCTGTTATCCCTAGCGTA**ggtacc 1771 **C-KmRI-SceIR**

I-SceI KpnI

**(h) C-GFPstop-SpR**

BamHI

ggatcc**ATGGTGAGCAAGGGCGAGGAG**CTGTTCACCGGGGTGGTGCCCATCCTGGTCGAG 60 **GFPF**

CTGGACGGCGACGTAAACGGCCACAAGTTCAGCGTGTCCGGCGAGGGCGAGGGCGATGCC 120

ACCTACGGCAAGCTGACCCTGAAGTtCATCTGCACCACCGGCAAGCTGCCCGTGCCCTGG 180

CCCACCCTCGTGACCACCcTGACcTACGGCGTGCAGTGCTTCAGCCGCTACCCCGACCAC 240

ATGAAGCAGCACGACTTCTTCAAGTCCGCCATGCCCGAAGGCTACGTCCAGGAGCGCACC 300

ATCTTCTTCAAGGACGACGGCAACTACAAGACCCGCGCCGAGGTGAAGTTCGAGGGCGAC 360

ACCCTGGTGAACCGCATCGAGCTGAAGGGCATCGACTTCAAGGAGGACGGCAACATCCTG 420

GGGCACAAGCTGGAGTACAACTACAACAGCCACAACGTCTATATCATGGCCGACAAGCAG 480

AAGAACGGCATCAAGGTGAACTTCAAGATCCGCCACAACATCGAGGACGGCAGCGTGCAG 540

CTCGCCGACCACTACCAGCAGAACACCCCCATCGGCGACGGCCCCGTGCTGCTGCCCGAC 600

AACCACTACCTGAGCACCCAGTCCGCCCTGAGCAAAGACCCCAACGAGAAGCGCGATCAC 660

ATGGTCCTGCTGGAGTTCGTGACCGCCGCCGGGATCACTCTCGGCATGGACGAGCTGTAC 720

AAG**TAACTATATTACCCTGTTATCCCTAGCGTAactagt**ctcatgttaccgatgctattc 780 **I-SceI**/**SpeI**

ggaagaacggcaactaagctgccgggtttgaaacacggatgatctcgcggagggtagcat 840

gttgattgtaacgatgacagagcgttgctgcctgtgatcaattcgggcacgaacccagtg 900

gacataagcctcgttcggttcgtaagctgtaatgcaagtagcgtaactgccgtcacgcaa 960

ctggtccagaaccttgaccgaacgcagcggtggtaacggcgcagtggcggttttcatggc 1020

ttcttgtt**ATG**ACATGTTTTTTTGGGGTACAGTCTATGCCTCGGGCATCCAAGCAGCAAG 1080

CGCGTTACGCCGTGGGTCGATGTTTGATGTTATGGAGCAGCAACGATGTTACGCAGCAGG 1140

GCAGTCGCCCTAAAACAAAGTTAAACATCATGGGGGAAGCGGTGATCGCCGAAGTATCGA 1200

CTCAACTATCAGAGGTAGTTGGCGTCATCGAGCGCCATCTCGAACCGACGTTGCTGGCCG 1260

TACATTTGTACGGCTCCGCAGTGGATGGCGGCCTGAAGCCACACAGTGATATTGATTTGC 1320

TGGTTACGGTGACCGTAAGGCTTGATGAAACAACGCGGCGAGCTTTGATCAACGACCTTT 1380 SpR

TGGAAACTTCGGCTTCCCCTGGAGAGAGCGAGATTCTCCGCGCTGTAGAAGTCACCATTG 1440

TTGTGCACGACGACATCATTCCGTGGCGTTATCCAGCTAAGCGCGAACTGCAATTTGGAG 1500

AATGGCAGCGCAATGACATTCTTGCAGGTATCTTCGAGCCAGCCACGATCGACATTGATC 1560

TGGCTATCTTGCTGACAAAAGCAAGAGAACATAGCGTTGCCTTGGTAGGTCCAGCGGCGG 1620

AGGAACTCTTTGATCCGGTTCCTGAACAGGATCTATTTGAGGCGCTAAATGAAACCTTAA 1680

CGCTATGGAACTCGCCGCCCGACTGGGCTGGCGATGAGCGAAATGTAGTGCTTACGTTGT 1740

CCCGCATTTGGTACAGCGCAGTAACCGGCAAAATCGCGCCGAAGGATGTCGCTGCCGACT 1800

GGGCAATGGAGCGCCTGCCGGCCCAGTATCAGCCCGTCATACTTGAAGCTAGACAGGCTT 1860

ATCTTGGACAAGAAGAAGATCGCTTGGCCTCGCGCGCAGATCAGTTGGAAGAATTTGTCC 1920

ACTACGTGAAAGGCGAGATCACCAAGGTAGTCGGCAAA**TAA**tgtctagctagaaattcgt 1980

tcaagccgacgccgcttcgcc**GAAGTGCTATATTACCCTGTTATCCCTAGCGTA**ggtacc 2040 **C-SpRI-SceIR**

I-SceI KpnI

**(i) C-mCherrystop-SpR**

BamHI

ggatcc**ATGGTGAGCAAGGGCGAGGAG**GATAACATGGCCATCATCAAGGAGTTCATGCGC 60 **GFPF**

TTCAAGGTGCACATGGAGGGCTCCGTGAACGGCCACGAGTTCGAGATCGAGGGCGAGGGC 120

GAGGGCCGCCCCTACGAGGGCACCCAGACCGCCAAGCTGAAGGTGACCAAGGGTGGCCCC 180

CTGCCCTTCGCCTGGGACATCCTGTCCCCTCAGTTCATGTACGGCTCCAAGGCCTACGTG 240

AAGCACCCCGCCGACATCCCCGACTACTTGAAGCTGTCCTTCCCCGAGGGCTTCAAGTGG 300 mCherry

GAGCGCGTGATGAACTTCGAGGACGGCGGCGTGGTGACCGTGACCCAGGACTCCTCCCTG 360

CAGGACGGCGAGTTCATCTACAAGGTGAAGCTGCGCGGCACCAACTTCCCCTCCGACGGC 420

CCCGTAATGCAGAAGAAGACCATGGGCTGGGAGGCCTCCTCCGAGCGGATGTACCCCGAG 480

GACGGCGCCCTGAAGGGCGAGATCAAGCAGAGGCTGAAGCTGAAGGACGGCGGCCACTAC 540

GACGCTGAGGTCAAGACCACCTACAAGGCCAAGAAGCCCGTGCAGCTGCCCGGCGCCTAC 600

AACGTCAACATCAAGTTGGACATCACCTCCCACAACGAGGACTACACCATCGTGGAACAG 660

TACGAACGCGCCGAGGGCCGCCACTCCACCGGCGGCATGGACGAGCTGTACAAG**TGACTA** 720

**TATTACCCTGTTATCCCTAGCGTAactagt**ctcatgttaccgatgctattcggaagaacg 780 **I-SceI/SpeI**

gcaactaagctgccgggtttgaaacacggatgatctcgcggagggtagcatgttgattgt 840

aacgatgacagagcgttgctgcctgtgatcaattcgggcacgaacccagtggacataagc 900

ctcgttcggttcgtaagctgtaatgcaagtagcgtaactgccgtcacgcaactggtccag 960

aaccttgaccgaacgcagcggtggtaacggcgcagtggcggttttcatggcttcttgtt**A** 1020

**TG**ACATGTTTTTTTGGGGTACAGTCTATGCCTCGGGCATCCAAGCAGCAAGCGCGTTACG 1080

CCGTGGGTCGATGTTTGATGTTATGGAGCAGCAACGATGTTACGCAGCAGGGCAGTCGCC 1140

CTAAAACAAAGTTAAACATCATGGGGGAAGCGGTGATCGCCGAAGTATCGACTCAACTAT 1200

CAGAGGTAGTTGGCGTCATCGAGCGCCATCTCGAACCGACGTTGCTGGCCGTACATTTGT 1260

ACGGCTCCGCAGTGGATGGCGGCCTGAAGCCACACAGTGATATTGATTTGCTGGTTACGG 1320

TGACCGTAAGGCTTGATGAAACAACGCGGCGAGCTTTGATCAACGACCTTTTGGAAACTT 1380 SpR

CGGCTTCCCCTGGAGAGAGCGAGATTCTCCGCGCTGTAGAAGTCACCATTGTTGTGCACG 1440

ACGACATCATTCCGTGGCGTTATCCAGCTAAGCGCGAACTGCAATTTGGAGAATGGCAGC 1500

GCAATGACATTCTTGCAGGTATCTTCGAGCCAGCCACGATCGACATTGATCTGGCTATCT 1560

TGCTGACAAAAGCAAGAGAACATAGCGTTGCCTTGGTAGGTCCAGCGGCGGAGGAACTCT 1620

TTGATCCGGTTCCTGAACAGGATCTATTTGAGGCGCTAAATGAAACCTTAACGCTATGGA 1680

ACTCGCCGCCCGACTGGGCTGGCGATGAGCGAAATGTAGTGCTTACGTTGTCCCGCATTT 1740

GGTACAGCGCAGTAACCGGCAAAATCGCGCCGAAGGATGTCGCTGCCGACTGGGCAATGG 1800

AGCGCCTGCCGGCCCAGTATCAGCCCGTCATACTTGAAGCTAGACAGGCTTATCTTGGAC 1860

AAGAAGAAGATCGCTTGGCCTCGCGCGCAGATCAGTTGGAAGAATTTGTCCACTACGTGA 1920

AAGGCGAGATCACCAAGGTAGTCGGCAAA**TAA**tgtctagctagaaattcgttcaagccga 1980

cgccgcttcgcc**GAAGTGCTATATTACCCTGTTATCCCTAGCGTA**ggtacc 2031 **C-SpRI-SceIR**

I-SceI KpnI

**Figure S4.** Nucleotide sequences of N- and C-terminal I-SceI insertion cassettes.

(a) Sequence of the N-terminal N-KmR-GFP-PIPL/N-KmR-GFP cassettes. For N-terminal fusion, the N-KmR-GFP-PIPL casette is PCR amplified with the N-KmRI-SceIF and PIPLR primers, whereas the N-KmR-GFP cassette is amplified by the primers N-KmRI-SceIF and GFPRnostop. Abbreviations: CobW: segment of the Co^2+^/Ni^2+^-binding domain of Arabidopsis COBW-like protein (At1g15730) carrying 18 His residues; StrepII: Strep-Tactin binding peptide; HA: hemagglutinin epitope, GFP: green fluorescent protein.

(b) Sequence of the N-terminal N-KmR-mCherry cassette. For N-terminal fusion, the cassette is amplified by the N-KmRI-SceIF and mCherrynostop primers.

(c) Sequence of the N-terminal N-SpR-GFP-PIPL / N-SpR-GFP cassettes. For N-terminal fusion, the N-SpR-GFP-PIPL cassette is PCR amplified with the N-SpRI-SceIF and PIPLR primers, whereas the N-SpR-GFP cassette is amplified by the N-SpRI-SceIF and GFPRnostop primers.

(d) Sequence of the N-terminal N-SpR-mCherry cassette. For N-terminal fusion, the cassette is amplified with the N-SpRI-SceIF and mCherrynostop primers.

(e) Sequence of the C-terminal C-GFPstop-KmR cassette. For C-terminal fusion, the cassette is amplified with the GFPF and C-KmRI-SceIR primers.

(f) Sequence of the C-terminal C-GFP-PIPLstop-KmR / C-PIPLstop-KmR cassettes. For C-terminal fusion, the C-GFP-PIPLstop-KmR cassette is amplified with the GFPF and C-KmRI-SceIR primers, whereas the C-PIPLstop-KmR cassette is amplified with the PIPLF and C-KmRI-SceIR primers.

(g) Sequence of the C-terminal C-mCherrystop-KmR cassette. For C-terminal fusion, the cassette is amplified with the GFPF and C-KmRI-SceIR primers.

(h) Sequence of the C-terminal C-GFPstop-SpR cassette. For C-terminal fusion, the cassette is amplified with the GFPF and C-SpRI-SceIR primers.

(i) Sequence of the C-terminal C-mCherrystop-SpR cassette. For C-terminal fusion, the cassette is amplified with the GFPF and C-SpRI-SceIR primers.
