# Supplementary material for: Gene modification by fast‐track recombineering for cellular localization and isolation of components of plant protein complexes
Source: Plant J. 2019 Jul 26;100(2):411–29. doi: 10.1111/tpj.14450 (PMC6852550; doi:10.1111/tpj.14450)
Supplement: Supplementary file 5 — Figure S5. Gibson assembly and sequences of pGAPBRHyg and pGAPBRKm binary vectors. [file TPJ-100-411-s005.docx]

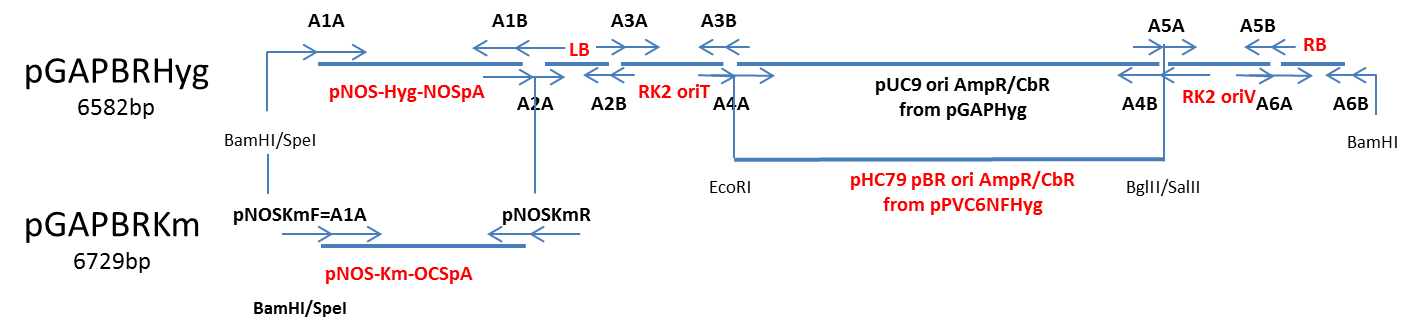
**(a)**

**(b)**

**pGAPBRHyg**

**BamHI**

**GGATCCattgtcgtttcccgcctt**cggtttaaactatcagtgtt**TGACAGGATATATTGG** 60 **BamHIF**

**CGGGTAAAC**ctaagagaaaagagcgtttattagaataatcggatatttaaaagggcgtga 120 **T-DNA RB**

aaaggtttatccgttcgtccatttgtatgtgcatgccaaccacagggttcccctcgggag 180

tgcttggcattccgtgcgataatgacttctgttcaaccacccaaacgtcggaaagcctga 240

cgacggagcagcattccaaaaagatcccttggctcgtctgggtcggctagaaggtcgagt 300

gggctgctgtggcttgatccctcaacgcggtcgcggacgtagcgcagcgccgaaaaatcc 360

tcgatcgcaaatccgaccggcacctggtcgctgaatgtcgatgccagcacctgcggcacg 420

tcaatgcttccgggcgtcgcgctcgggctgatcgcccatcccgttactgccccgatcccg 480

gcaatggcaaggactgccagcgctgccatttttggggtgaggccgttcgcggccgagggg 540

cgcagcccctggggggatgggaggcccgcgttagcgggccgggagggttcgagaaggggg 600

ggcaccccccttcggcgtgcgcggtcacgcgcacagggcgcagccctggttaaaaacaag 660

gtttataaatattggtttaaaagcaggttaaaagacaggttagcggtggccgaaaaacgg 720

gcggaaacccttgcaaatgctggattttctgcctgtggacagcccctcaaatgtcaatag 780 **RK2 ori_V_**

gtgcgcccctcatctgtcagcactctgcccctcaagtgtcaaggatcgcgcccctcatct 840

gtcagtagtcgcgcccctcaagtgtcaataccgcagggcacttatccccaggcttgtcca 900

catcatctgtgggaaactcgcgtaaaatcaggcgttttcgccgatttgcgaggctggcca 960

gctccacgtcgccggccgaaatcgagcctgcccctcatctatcaacgccgcgccgggtga 1020

gtcggcccctcaagtgtcaacgtccgcccctcatctgtcagtgagggccaagttttccgc 1080

gaggtatccacaacgccggcggccgcggtgtctcgcacacggcttcgacggcgtttctgg 1140

cgcgtttgcagggccatagacggccgccagcccagcggcgagggcaaccagcccggtgag 1200

cgtcggaaaggcgctggaagccccgtagcgacgcggagaggggcgagacaagccaagggc 1260

gcaggctcgatgcgcagcacgacatagccggttctcgcaaggacgagaatttccctgcgg 1320

tgcccctcaagtgtcaatgaaagtttccaacgcgagccattcgcgagagccttgagtcca 1380

cgctagatcctttttgtccggtgttgggttgaaggtgaagccgtcg**agatct**gcatcgca 1440 BglII

ggatgctgctggctaccctgtggaacacctacatctgtattaacgaagcgctggcattga 1500

ccctgagtgatttttctctggtcccgccgcatccataccgccagttgtttaccctcacaa 1560

cgttccagtaaccgggcatgttcatcatcagtaacccgtatcgtgagcatcctctctcgt 1620

ttcatcggtatcattacccccatgaacagaaatcccccttacacggaggcatcagtgacc 1680

aaacaggaaaaaaccgcccttaacatggcccgctttatcagaagccagacattaacgctt 1740 **pBR-ROP**

ctggagaaactcaacgagctggacgcggatgaacaggcagacatctgtgaatcgcttcac 1800

gaccacgctgatgagctttaccgcagctgcctcgcgcgtttcggtgatgacggtgaaaac 1860

ctctgacacatgcagctcccggagacggtcacagcttgtctgtaagcggatgccgggagc 1920

agacaagcccgtcagggcgcgtcagcgggtgttggcgggtgtcggggcgcagccatgacc 1980

cagtcacgtagcgatagcggagtgtatactggcttaactatgcggcatcagagcagattg 2040

tactgagagtgcaccatatgcggtgtgaaataccgcacagatgcgtaaggagaaaatacc 2100

gcatcaggcgctcttccgcttcctcgctcactgactcgctgcgctcggtcgttcggctgc 2160

ggcgagcggtatcagctcactcaaaggcggtaatacggttatccacagaatcaggggata 2220

acgcaggaaagaacatgtgagcaaaaggccagcaaaaggccaggaaccgtaaaaaggccg 2280

cgttgctggcgtttttccataggctccgcccccctgacgagcatcacaaaaatcgacgct 2340 **pBRori**

caagtcagaggtggcgaaacccgacaggactataaagataccaggcgtttccccctggaa 2400

gctccctcgtgcgctctcctgttccgaccctgccgcttaccggatacctgtccgcctttc 2460

tcccttcgggaagcgtggcgctttctcatagctcacgctgtaggtatctcagttcggtgt 2520

aggtcgttcgctccaagctgggctgtgtgcacgaaccccccgttcagcccgaccgctgcg 2580

ccttatccggtaactatcgtcttgagtccaacccggtaagacacgacttatcgccactgg 2640

cagcagccactggtaacaggattagcagagcgaggtatgtaggcggtgctacagagttct 2700

tgaagtggtggcctaactacggctacactagaaggacagtatttggtatctgcgctctgc 2760

tgaagccagttaccttcggaaaaagagttggtagctcttgatccggcaaacaaaccaccg 2820

ctggtagcggtggtttttttgtttgcaagcagcagattacgcgcagaaaaaaaggatctc 2880

aagaagatcctttgatcttttctacggggtctgacgctcagtggaacgaaaactcacgtt 2940

aagggattttggtcatgagattatcaaaaaggatcttcacctagatccttttaaattaaa 3000

aatgaagttttaaatcaatctaaagtatatatgagtaaacttggtctgacag**TTA**ccaat 3060

gcttaatcagtgaggcacctatctcagcgatctgtctatttcgttcatccatagttgcct 3120

gactccccgtcgtgtagataactacgatacgggagggcttaccatctggccccagtgctg 3180

caatgataccgcgagacccacgctcaccggctccagatttatcagcaataaaccagccag 3240

ccggaagggccgagcgcagaagtggtcctgcaactttatccgcctccatccagtctatta 3300

attgttgccgggaagctagagtaagtagttcgccagttaatagtttgcgcaacgttgttg 3360

ccattgctgcaggcatcgtggtgtcacgctcgtcgtttggtatggcttcattcagctccg 3420 **AmpR/CbR**

gttcccaacgatcaaggcgagttacatgatcccccatgttgtgcaaaaaagcggttagct 3480

ccttcggtcctccgatcgttgtcagaagtaagttggccgcagtgttatcactcatggtta 3540

tggcagcactgcataattctcttactgtcatgccatccgtaagatgcttttctgtgactg 3600

gtgagtactcaaccaagtcattctgagaatagtgtatgcggcgaccgagttgctcttgcc 3660

cggcgtcaacacgggataataccgcgccacatagcagaactttaaaagtgctcatcattg 3720

gaaaacgttcttcggggcgaaaactctcaaggatcttaccgctgttgagatccagttcga 3780

tgtaacccactcgtgcacccaactgatcttcagcatcttttactttcaccagcgtttctg 3840

ggtgagcaaaaacaggaaggcaaaatgccgcaaaaaagggaataagggcgacacggaaat 3900

gttgaatact**CAT**actcttcctttttcaatattattgaagcatttatcagggttattgtc 3960

tcatgagcggatacatatttgaatgtatttagaaaaataaacaaataggggttccgcgca 4020

catttccccgaaaagtgccacctgacgtctaagaaaccattattatcatgacattaacct 4080

ataaaaataggcgtatcacgaggccctttcgtcttcaa**gaattc**tcgtctctcgcctgtc 4140 EcoRI

ccctcagttcagtaatttcctgcatttgcctgtttccagtcggtagatattccacaaaac 4200

agcagggaagcagcgcttttccgctgcataaccctgcttcggggtcattatagcgatttt 4260

ttcggtatatccatcctttttcgcacgatatacaggattttgccaaagggttcgtgtaga 4320 **RK2 ori_T_**

ctttccttggtgtatccaacggcgtcagccgggcaggataggtgaagtaggcccacccgc 4380

gagcgggtgttccttcttcactgtcccttattcgcacctggcggtgctcaacgggaatcc 4440

tgctctgcgaggctggccggctaccgccggcgtaacagatgagggcaagcggatggctga 4500

tgaaaccaagccaaccaggaagggcagcccacctatcaaggtgtactgccttccagacga 4560

acgaagcatgtggatcactccgttgccccgtcgctcaccgtgttggggggaaggtgcaca 4620

tggctcagttctcaatggaaattatctgcctaaccggctcagttctgcgtagaaaccaac 4680

atgcaagctccaccgggtgcaaagcggcagcggc**GGCAGGATATATTCAATTGTAAAT**gg 4740 **T-DNA LB**

cttcatgtccgggaaatctacatggatcagcaatgagtatgatggtcaatatggagaaaa 4800

agaaagagtaattaccaattttttttcaattcaaaaatgtagatgtccgcagcgttatta 4860

taaaatgaaagtacattttgataaaacgacaaattacgatccgtcgtatttataggcgaa 4920

agca**agatct**gcttggcaattcccgatctagtaacatagatgacaccgcgcgcgataatt 4980 BglII

tatcctagtttgcgcgctatattttgttttctatcgcgtattaaatgtataattgcggga 5040

ctctaatcataaaaacccatctcataaataacgtcatgcattacatgttaattattacat 5100 **NOSpA**

gcttaacgtaattcaacagaaattatatgataatcatcgcaagaccggcaacaggattca 5160

atcttaagaaactttattgccaaatgtttgaacgatcggggatcgaagatcccggtcggc 5220

atctact**CTA**ttcctttgccctcggacgagtgctggggcgtcggtttccactatcggcga 5280

gtacttctacacagccatcggtccagacggccgcgcttctgcgggcgatttgtgtacgcc 5340

cgacagtcccggctccggatcggacgattgcgtcgcatcgaccctgcgcccaagctgcat 5400

catcgaaattgccgtcaaccaagctctgatagagttggtcaagaccaatgcggagcatat 5460

acgcccggagccgcggcgatcctgcaagctccggatgcctccgctcgaagtagcgcgtct 5520

gctgctccatacaagccaaccacggcctccagaagaagatgttggcgacctcgtattggg 5580

aatccccgaacatcgcctcgctccagtcaatgaccgctgttatgcggccattgtccgtca 5640

ggacattgttggagccgaaatccgcgtgcacgaggtgccggacttcggggcagtcctcgg 5700 **HygR**

cccaaagcatcagctcatcgagagcctgcgcgacggacgcactgacggtgtcgtccatca 5760

cagtttgccagtgatacacatggggatcagcaatcgcgcatatgaaatcacgccatgtag 5820

tgtattgaccgattccttgcggtccgaatgggccgaacccgctcgtctggctaagatcgg 5880

ccgcagcgatcgcatCCATGGcctccgcgaccggctgcagaacagcgggcagttcggttt 5940

caggcaggtcttgcaacgtgacaccctgtgcacggcgggagatgcaataggtcaggctct 6000

cgctgaatgccccaatgtcaagcacttccggaatcgggagcgcggccgatgcaaagtgcc 6060

gataaacataacgatctttgtagaaaccatcggcgcagctatttacccgcaggacatatc 6120

cacgccctcctacatcgaagctgaaagcacgagattcttcgccctccgagagctgcatca 6180

ggtcggagacgctgtcgaacttttcgatcagaaacttctcgacagacgtcgcggtgagtt 6240

caggcttttt**CAT**atcttattgccccccgatctggattgagagtgaatatgagactctaa 6300

ttggataccgaggggaatttatggaacgtcagtggagcatttttgacaagaaatatttgc 6360 **pNOS**

tagctgatagtgaccttaggcgacttttgaacgcgcaataatggtttctgacgtatgtgc 6420

ttagctcattaaactccagaaacccgcggctgagtggctccttcaacgttgcggttctgt 6480

cagttccaaacgtaaaacggcttgtcccgcgtcatcggcgggggtcataacgtgactccc 6540

ttaattctccgctcatgat**cttgatcccctgcgccaACTAGT** 6582 **SpeIR**

**SpeI**

**(c)**

**pGAPBRKm**

**BamHI**

**GGATCCattgtcgtttcccgcctt**cggtttaaactatcagtgtt**TGACAGGATATATTGG** 60 **BamHIF**

**CGGGTAAAC**ctaagagaaaagagcgtttattagaataatcggatatttaaaagggcgtga 120 **T-DNA RB**

aaaggtttatccgttcgtccatttgtatgtgcatgccaaccacagggttcccctcgggag 180

tgcttggcattccgtgcgataatgacttctgttcaaccacccaaacgtcggaaagcctga 240

cgacggagcagcattccaaaaagatcccttggctcgtctgggtcggctagaaggtcgagt 300

gggctgctgtggcttgatccctcaacgcggtcgcggacgtagcgcagcgccgaaaaatcc 360

tcgatcgcaaatccgaccggcacctggtcgctgaatgtcgatgccagcacctgcggcacg 420

tcaatgcttccgggcgtcgcgctcgggctgatcgcccatcccgttactgccccgatcccg 480

gcaatggcaaggactgccagcgctgccatttttggggtgaggccgttcgcggccgagggg 540

cgcagcccctggggggatgggaggcccgcgttagcgggccgggagggttcgagaaggggg 600

ggcaccccccttcggcgtgcgcggtcacgcgcacagggcgcagccctggttaaaaacaag 660

gtttataaatattggtttaaaagcaggttaaaagacaggttagcggtggccgaaaaacgg 720

gcggaaacccttgcaaatgctggattttctgcctgtggacagcccctcaaatgtcaatag 780 **RK2 ori_V_**

gtgcgcccctcatctgtcagcactctgcccctcaagtgtcaaggatcgcgcccctcatct 840

gtcagtagtcgcgcccctcaagtgtcaataccgcagggcacttatccccaggcttgtcca 900

catcatctgtgggaaactcgcgtaaaatcaggcgttttcgccgatttgcgaggctggcca 960

gctccacgtcgccggccgaaatcgagcctgcccctcatctatcaacgccgcgccgggtga 1020

gtcggcccctcaagtgtcaacgtccgcccctcatctgtcagtgagggccaagttttccgc 1080

gaggtatccacaacgccggcggccgcggtgtctcgcacacggcttcgacggcgtttctgg 1140

cgcgtttgcagggccatagacggccgccagcccagcggcgagggcaaccagcccggtgag 1200

cgtcggaaaggcgctggaagccccgtagcgacgcggagaggggcgagacaagccaagggc 1260

gcaggctcgatgcgcagcacgacatagccggttctcgcaaggacgagaatttccctgcgg 1320

tgcccctcaagtgtcaatgaaagtttccaacgcgagccattcgcgagagccttgagtcca 1380

cgctagatcctttttgtccggtgttgggttgaaggtgaagccgtcg**agatct**gcatcgca 1440 BglII

ggatgctgctggctaccctgtggaacacctacatctgtattaacgaagcgctggcattga 1500

ccctgagtgatttttctctggtcccgccgcatccataccgccagttgtttaccctcacaa 1560

cgttccagtaaccgggcatgttcatcatcagtaacccgtatcgtgagcatcctctctcgt 1620

ttcatcggtatcattacccccatgaacagaaatcccccttacacggaggcatcagtgacc 1680

aaacaggaaaaaaccgcccttaacatggcccgctttatcagaagccagacattaacgctt 1740 **pBR-ROP**

ctggagaaactcaacgagctggacgcggatgaacaggcagacatctgtgaatcgcttcac 1800

gaccacgctgatgagctttaccgcagctgcctcgcgcgtttcggtgatgacggtgaaaac 1860

ctctgacacatgcagctcccggagacggtcacagcttgtctgtaagcggatgccgggagc 1920

agacaagcccgtcagggcgcgtcagcgggtgttggcgggtgtcggggcgcagccatgacc 1980

cagtcacgtagcgatagcggagtgtatactggcttaactatgcggcatcagagcagattg 2040

tactgagagtgcaccatatgcggtgtgaaataccgcacagatgcgtaaggagaaaatacc 2100

gcatcaggcgctcttccgcttcctcgctcactgactcgctgcgctcggtcgttcggctgc 2160

ggcgagcggtatcagctcactcaaaggcggtaatacggttatccacagaatcaggggata 2220

acgcaggaaagaacatgtgagcaaaaggccagcaaaaggccaggaaccgtaaaaaggccg 2280

cgttgctggcgtttttccataggctccgcccccctgacgagcatcacaaaaatcgacgct 2340 **pBRori**

caagtcagaggtggcgaaacccgacaggactataaagataccaggcgtttccccctggaa 2400

gctccctcgtgcgctctcctgttccgaccctgccgcttaccggatacctgtccgcctttc 2460

tcccttcgggaagcgtggcgctttctcatagctcacgctgtaggtatctcagttcggtgt 2520

aggtcgttcgctccaagctgggctgtgtgcacgaaccccccgttcagcccgaccgctgcg 2580

ccttatccggtaactatcgtcttgagtccaacccggtaagacacgacttatcgccactgg 2640

cagcagccactggtaacaggattagcagagcgaggtatgtaggcggtgctacagagttct 2700

tgaagtggtggcctaactacggctacactagaaggacagtatttggtatctgcgctctgc 2760

tgaagccagttaccttcggaaaaagagttggtagctcttgatccggcaaacaaaccaccg 2820

ctggtagcggtggtttttttgtttgcaagcagcagattacgcgcagaaaaaaaggatctc 2880

aagaagatcctttgatcttttctacggggtctgacgctcagtggaacgaaaactcacgtt 2940

aagggattttggtcatgagattatcaaaaaggatcttcacctagatccttttaaattaaa 3000

aatgaagttttaaatcaatctaaagtatatatgagtaaacttggtctgacag**TTA**ccaat 3060

gcttaatcagtgaggcacctatctcagcgatctgtctatttcgttcatccatagttgcct 3120

gactccccgtcgtgtagataactacgatacgggagggcttaccatctggccccagtgctg 3180

caatgataccgcgagacccacgctcaccggctccagatttatcagcaataaaccagccag 3240

ccggaagggccgagcgcagaagtggtcctgcaactttatccgcctccatccagtctatta 3300

attgttgccgggaagctagagtaagtagttcgccagttaatagtttgcgcaacgttgttg 3360

ccattgctgcaggcatcgtggtgtcacgctcgtcgtttggtatggcttcattcagctccg 3420 **AmpR/CbR**

gttcccaacgatcaaggcgagttacatgatcccccatgttgtgcaaaaaagcggttagct 3480

ccttcggtcctccgatcgttgtcagaagtaagttggccgcagtgttatcactcatggtta 3540

tggcagcactgcataattctcttactgtcatgccatccgtaagatgcttttctgtgactg 3600

gtgagtactcaaccaagtcattctgagaatagtgtatgcggcgaccgagttgctcttgcc 3660

cggcgtcaacacgggataataccgcgccacatagcagaactttaaaagtgctcatcattg 3720

gaaaacgttcttcggggcgaaaactctcaaggatcttaccgctgttgagatccagttcga 3780

tgtaacccactcgtgcacccaactgatcttcagcatcttttactttcaccagcgtttctg 3840

ggtgagcaaaaacaggaaggcaaaatgccgcaaaaaagggaataagggcgacacggaaat 3900

gttgaatact**CAT**actcttcctttttcaatattattgaagcatttatcagggttattgtc 3960

tcatgagcggatacatatttgaatgtatttagaaaaataaacaaataggggttccgcgca 4020

catttccccgaaaagtgccacctgacgtctaagaaaccattattatcatgacattaacct 4080

ataaaaataggcgtatcacgaggccctttcgtcttcaa**gaattc**tcgtctctcgcctgtc 4140 EcoRI

ccctcagttcagtaatttcctgcatttgcctgtttccagtcggtagatattccacaaaac 4200

agcagggaagcagcgcttttccgctgcataaccctgcttcggggtcattatagcgatttt 4260

ttcggtatatccatcctttttcgcacgatatacaggattttgccaaagggttcgtgtaga 4320 **RK2 ori_T_**

ctttccttggtgtatccaacggcgtcagccgggcaggataggtgaagtaggcccacccgc 4380

gagcgggtgttccttcttcactgtcccttattcgcacctggcggtgctcaacgggaatcc 4440

tgctctgcgaggctggccggctaccgccggcgtaacagatgagggcaagcggatggctga 4500

tgaaaccaagccaaccaggaagggcagcccacctatcaaggtgtactgccttccagacga 4560

acgaagcatgtggatcactccgttgccccgtcgctcaccgtgttggggggaaggtgcaca 4620

tggctcagttctcaatggaaattatctgcctaaccggctcagttctgcgtagaaaccaac 4680

atgcaagctccaccgggtgcaaagcggcagcggc**GGCAGGATATATTCAATTGTAAAT**gg 4740 **T-DNA LB**

cttcatgtccgggaaatctacatggatcagcaatgagtatgatggtcaatatggagaaaa 4800

agaaagagtaattaccaattttttttcaattcaaaaatgtagatgtccgcagcgttatta 4860

taaaatgaaagtacattttgataaaacgacaaattacgatccgtcgtatttataggcgaa 4920

agca**agatct**ccaatactcaacttcaaggaatctcacccatgcgcgccggcggggaaccg 4980

gagttcccttcagtgagcgttattagttcgccgctcggtgtgtcgtagatactagcccct 5040

ggggccttttgaaatttgaataagatttatgtaatcagtcttttaggtttgaccggttct 5100

gccgctttttttaaaattggatttgtaataataaaacgcaattgtttgttattgtggcgc 5160 **OCSpA**

tctatcatagatgtcgctataaacctattcagcacaatatattgttttcattttaatatt 5220

gtacatataagtagtagggtacaatcagtaaattgaacggagaatattattcataaaaat 5280

acgatagtaacgggtgatatattcattagaatgaaccgaaaccggcggtaaggatctgag 5340

ctacacatgctcaggttttttacaacgtgcacaacagaattgaaagcaaatatcatgcga 5400

tcataggcgtctcgcatatctcattaaagcagggggtgggcgaagaactccagcatgaga 5460

tccccgcgctggaggatcatccagccggcgtcccggaaaacgattccgaagcccaacctt 5520

tcatagaaggcggcggtggaatcgaaatctcgtgatggcaggttgggcgtcgcttggtcg 5580

gtcatttcgaaccccagagtcccgc**TCA**gaagaactcgtcaagaaggcgatagaaggcga 5640

tgcgctgcgaatcgggagcggcgataccgtaaagcacgaggaagcggtcagcccattcgc 5700

cgccaagctcttcagcaatatcacgggtagccaacgctatgtcctgatagcggtccgcca 5760

cacccagccggccacagtcgatgaatccagaaaagcggccattttccaccatgatattcg 5820

gcaagcaggcatcgccatgggtcacgacgagatcctcgccgtcgggcatgcgcgccttga 5880

gcctggcgaacagttcggctggcgcgagcccctgatgctcttcgtccagatcatcctgat 5940 **KmR**

cgacaagaccggcttccatccgagtacgtgctcgctcgatgcgatgtttcgcttggtggt 6000

cgaatgggcaggtagccggatcaagcgtatgcagccgccgcattgcatcagccatgatgg 6060

atactttctcggcaggagcaaggtgagatgacaggagatcctgccccggcacttcgccca 6120

atagcagccagtcccttcccgcttcagtgacaacgtcgagcacagctgcgcaaggaacgc 6180

ccgtcgtggccagccacgatagccgcgctgcctcgtcctgcagttcattcagggcaccgg 6240

acaggtcggtcttgacaaaaagaaccgggcgcccctgcgctgacagccggaacacggcgg 6300

catcagagcagccgattgtctgttgtgcccagtcatagccgaatagcctctccacccaag 6360

cggccggagaacctgcgtgcaatccatcttgttcaatccacatgat**CAT**gggccggatct 6420

ggattgagagtgaatatgagactctaattggataccgaggggaatttatggaacgtcagt 6480

ggagcatttttgacaagaaatatttgctagctgatagtgaccttaggcgacttttgaacg 6540

cgcaataatggtttctgacgtatgtgcttagctcattaaactccagaaacccgcggctga 6600

gtggctccttcaacgttgcggttctgtcagttccaaacgtaaaacggcttgtcccgcgtc 6660

atcggcgggggtcataacgtgactcccttaattctccgctcatgat**cttgatcccctgcg** 6720

**ccaACTAGT** 6729 **SpeIR**

**SpeI**

**Figure S5.** Gibson assembly and sequences of pGAPBRHyg and pGAPBRKm binary vectors.

(a) The pGAPBRHyg and pGAPBRKm binary vectors were created by Gibson assembly from segments depicted in the figure, which were PCR amplified with the primers marked by arrows and listed in Table S1. Subsequently, the pUC9 replicon was replaced with an EcoRI-BglII fragment of the cosmid pHC79 from pPCV6NGHyg (Koncz et al., 1989). (b) and (c): Sequences of pGAPBRHyg and pGAPBRKm vectors between the BamHI and SpeI sites used for PCR amplification. The vectors are PCR amplified with the primers BamHIF and SpeIR. Reverse complement of 50 nt Flank1 primers is fused 3’ to reverse complement of SpeIR, whereas Flank2 is fused to BamHIF, and PCR amplification is performed with Q5 DNA polymerase using enhancer (NEB) as described in the Experimental Procedures. Abbreviations: T-DNA RB: right 25 bp border sequence of pTiC58 T-DNA, black arrow indicates the direction of T-DNA transfer; RK2oriV: DNA replication origin of RK2 plasmid; pBR-ROP: ROP gene, which regulates plasmid DNA replication by modulating the initiation of transcription of the primer RNA precursor; pBRori: pBR plasmid replication origin; AmpR/CbR: ampicillin/carbenicillin resistance gene; RK2oriT: conjugational transfer origin of RK2 plasmid; T-DNA LB: left 25 bp border of pTiAch5 T-DNA; NOSpA: 3’-UTR carrying polyadenylation sequences of pTiC58 nopaline synthase gene; HygR: hyromycin resistance/ aminoglycoside phosphotransferase IV gene; pNOS: promoter of pTiC58 nopaline synthase gene; KmR: kanamycin resistance/aminoglycoside phosphotransferase II gene. The positions of EcoRI-BglII sites used for swapping the pUC9 and pHC79 replicons is indicated.
