# Supplementary material for: Gene modification by fast‐track recombineering for cellular localization and isolation of components of plant protein complexes
Source: Plant J. 2019 Jul 26;100(2):411–29. doi: 10.1111/tpj.14450 (PMC6852550; doi:10.1111/tpj.14450)
Supplement: Supplementary file 6 — Figure S6. Localization of CDKF;1:GFP, CDKD;1:GFP, CDKD2:GFP–PIPL, CDKD;3:GFP, CYCH:GFP; CYCH:mCherry and HISTONE H3.1:mCherry proteins in roots, lateral roots, hypocotyls and primary leaves of 7‐day‐old seedlings by confocal microscopy. [file TPJ-100-411-s006.docx]

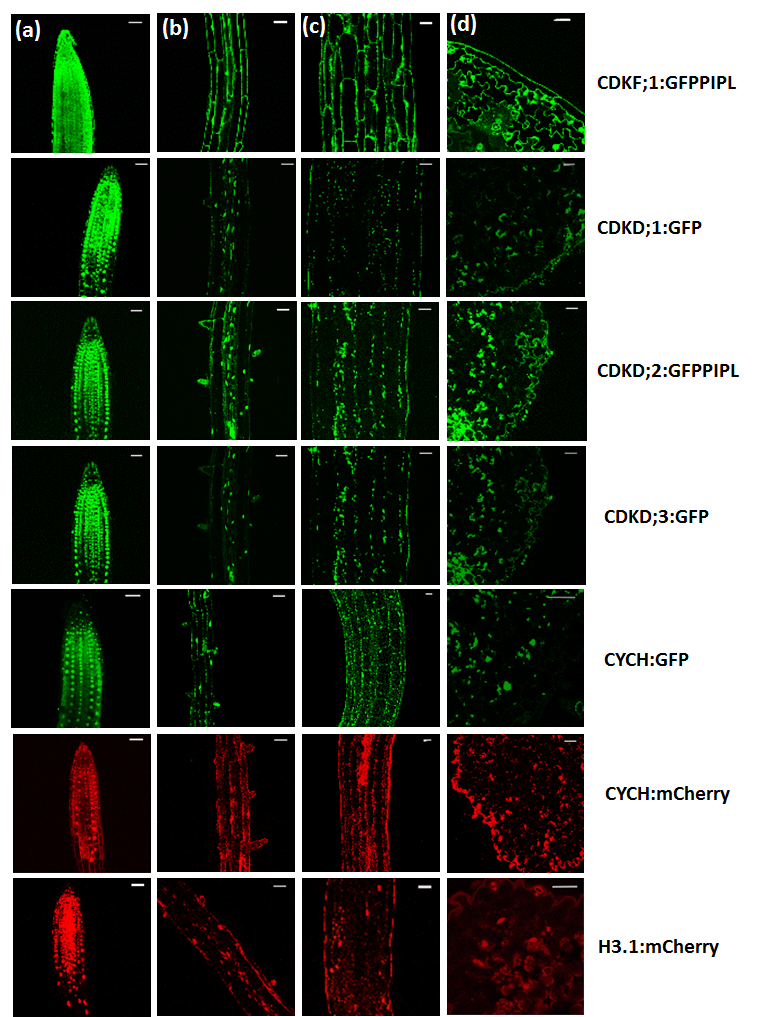


**Figure S6.** Localization of CDKF;1:GFP, CDKD;1:GFP, CDKD2:GFPPIPL, CDKD;3:GFP, CYCH:GFP; CYCH:mCherry and HISTONE H3.1:mCherry proteins in roots, lateral roots, hypocotyls and primary leaves of 7 days-old seedlings by confocal microscopy. (a) Primary roots, (b) lateral roots, (c) hypocotyls and (d) primary leaves. Bars: 30 μm.
