# Supplementary material for: Gene modification by fast‐track recombineering for cellular localization and isolation of components of plant protein complexes
Source: Plant J. 2019 Jul 26;100(2):411–29. doi: 10.1111/tpj.14450 (PMC6852550; doi:10.1111/tpj.14450)
Supplement: Supplementary file 7 — Figure S7. Localization of CDKF;1:GFP, CDKD;1:GFP, CDKD2:GFP–PIPL, CDKD;3:GFP, CYCH:GFP; CYCH:mCherry and HISTONE H3.1:mCherry proteins in pollens, pistil tissues and seed coat. [file TPJ-100-411-s007.docx]

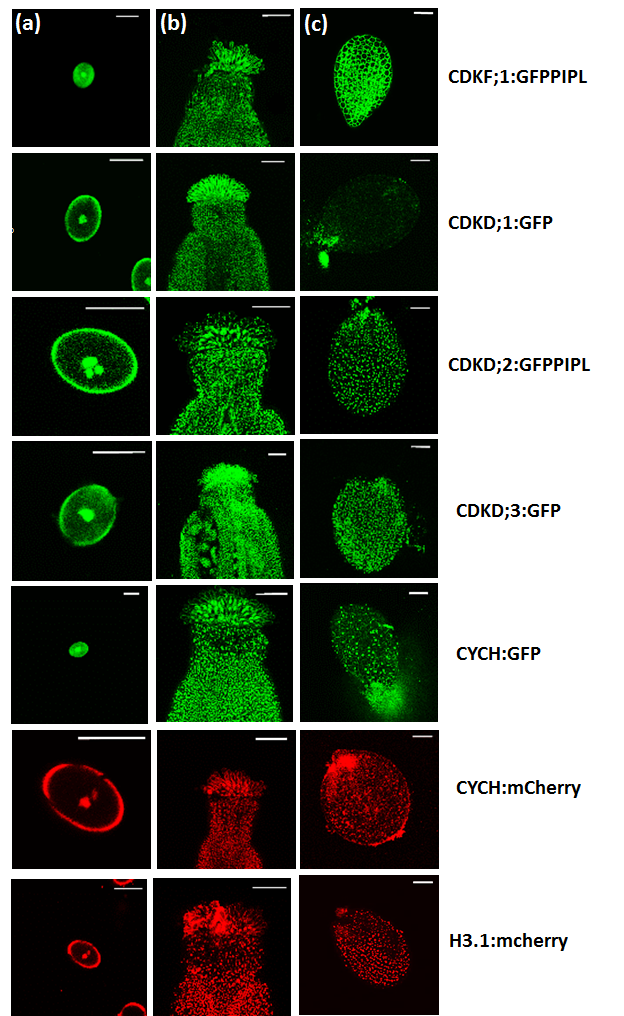


**Figure S7.** Localization of CDKF;1:GFP, CDKD;1:GFP, CDKD2:GFPPIPL, CDKD;3:GFP, CYCH:GFP; CYCH:mCherry and HISTONE H3.1:mCherry proteins in pollens, pistil tissues and seed coat. (a) pollens; (b) pistils and (c) seed coat. Bars: 20μ in (a) and 100 μm in (b) and (c).
